# Correction: *An electronic health records cohort study on heart failure following myocardial infarction in England: incidence and predictors*

**DOI:** 10.1136/bmjopen-2017-018331corr1

**Published:** 2018-03-22

Gho JMIH, Schmidt AF, Pasea L, *et al*. An electronic health records cohort study on heart failure following myocardial infarction in England: incidence and predictors. *BMJ Open* 2018;8:e018331. doi:10.1136/bmjopen-2017-018331.

Johannes M I H Gho and Amand F Schmidt are shared first authors of this paper.

