# Supplementary material for: An electronic health records cohort study on heart failure following myocardial infarction in England: incidence and predictors
Source: BMJ Open. 2018 Mar 3;8(3):e018331. doi: 10.1136/bmjopen-2017-018331 (PMC5855447; doi:10.1136/bmjopen-2017-018331)
Supplement: Supplementary file 1 [file bmjopen-2017-018331supp001.pdf]

Supplementary Data

An Electronic Health Records cohort study on Heart Failure Following Myocardial Infarction in England: Incidence and Predictors

Johannes M.I.H. Gho, Amand F. Schmidt, Laura Pasea, Stefan Koudstaal, Mar Pujades-Rodriguez, Spiros Denaxas, Anoop D. Shah, Riyaz S. Patel, Chris P. Gale, Arno W. Hoes, John G. Cleland, Harry Hemingway, Folkert W. Asselbergs

Phenotype coding lists

CALIBER myocardial infarction phenotype
Fatal or non-fatal myocardial infarction (MI) as recorded in any of the constituent CALIBER data sources. The type of myocardial infarction (ST elevation MI, STEMI; or non ST elevation MI, NSTEMI) is recorded only in MINAP and CPRD.

Read codes used to identify myocardial infarction in primary care records (Clinical Research Practice Datalink)

Category	Clinical code	Clinical term	Datasource lookup
**STEMI (3)**	G30X000	Acute ST segment elevation myocardial infarction	12229
**NSTEMI (4)**	G307100	Acute non-ST segment elevation myocardial infarction	10562
**Acute MI not further specified (5)**	323..00	ECG: myocardial infarction	7783
**Acute MI not further specified (5)**	3233.00	ECG: antero-septal infarct.	26975
**Acute MI not further specified (5)**	3234.00	ECG:posterior/inferior infarct	26972
**Acute MI not further specified (5)**	3235.00	ECG: subendocardial infarct	55401
**Acute MI not further specified (5)**	3236.00	ECG: lateral infarction	52705
**Acute MI not further specified (5)**	323Z.00	ECG: myocardial infarct NOS	59032
**Acute MI not further specified (5)**	889A.00	Diab mellit insulin-glucose infus acute myocardial infarct	61670
**Acute MI not further specified (5)**	G30..00	Acute myocardial infarction	241
**Acute MI not further specified (5)**	G30..12	Coronary thrombosis	2491
**Acute MI not further specified (5)**	G30..13	Cardiac rupture following myocardial infarction (MI)	30421
**Acute MI not further specified (5)**	G30..15	MI - acute myocardial infarction	1677
**Acute MI not further specified (5)**	G30..16	Thrombosis - coronary	13571
**Acute MI not further specified (5)**	G300.00	Acute anterolateral infarction	12139
**Acute MI not further specified (5)**	G301.00	Other specified anterior myocardial infarction	5387
**Acute MI not further specified (5)**	G301000	Acute anteroapical infarction	40429
**Acute MI not further specified (5)**	G301100	Acute anteroseptal infarction	17872
**Acute MI not further specified (5)**	G301z00	Anterior myocardial infarction NOS	14897
**Acute MI not further specified (5)**	G302.00	Acute inferolateral infarction	8935
**Acute MI not further specified (5)**	G303.00	Acute inferoposterior infarction	29643
**Acute MI not further specified (5)**	G304.00	Posterior myocardial infarction NOS	23892
**Acute MI not further specified (5)**	G305.00	Lateral myocardial infarction NOS	14898
**Acute MI not further specified (5)**	G306.00	True posterior myocardial infarction	63467
**Acute MI not further specified (5)**	G307.00	Acute subendocardial infarction	3704
**Acute MI not further specified (5)**	G307000	Acute non-Q wave infarction	9507

**Acute MI not further specified (5)**	G308.00	Inferior myocardial infarction NOS	1678
**Acute MI not further specified (5)**	G309.00	Acute Q-wave infarct	30330
**Acute MI not further specified (5)**	G30B.00	Acute posterolateral myocardial infarction	32854
**Acute MI not further specified (5)**	G30X.00	Acute transmural myocardial infarction of unspecif site	29758
**Acute MI not further specified (5)**	G30y.00	Other acute myocardial infarction	34803
**Acute MI not further specified (5)**	G30y000	Acute atrial infarction	28736
**Acute MI not further specified (5)**	G30y100	Acute papillary muscle infarction	62626
**Acute MI not further specified (5)**	G30y200	Acute septal infarction	41221
**Acute MI not further specified (5)**	G30yz00	Other acute myocardial infarction NOS	46017
**Acute MI not further specified (5)**	G30z.00	Acute myocardial infarction NOS	14658
**Acute MI not further specified (5)**	G31y100	Microinfarction of heart	68357
**Acute MI not further specified (5)**	G38..00	Postoperative myocardial infarction	32272
**Acute MI not further specified (5)**	G380.00	Postoperative transmural myocardial infarction anterior wall	46112
**Acute MI not further specified (5)**	G381.00	Postoperative transmural myocardial infarction inferior wall	46276
**Acute MI not further specified (5)**	G384.00	Postoperative subendocardial myocardial infarction	41835
**Acute MI not further specified (5)**	G38z.00	Postoperative myocardial infarction, unspecified	68748
**Acute MI not further specified (5)**	Gyu3400	[X]Acute transmural myocardial infarction of unspecif site	96838

Hospital Episode Statistics

**Category (code)**	ICD10 code	ICD10 term
**Acute MI not further specified (5)**	I21	Acute myocardial infarction

OPCS

**Category (code)**	OPCS 4	OPCS 4 term
**Transluminal coronary thrombolysis (2)**	K50.2	Percutaneous transluminal coronary thrombolysis using streptokinase
**Transluminal coronary thrombolysis (2)**	K50.3	Percutaneous transluminal injection of therapeutic substance into coronary artery NEC

Office for National Statistics (ONS)

**Category (code)**	ICD10 code	ICD10 term
**Death from MI (3)**	I21	Acute myocardial infarction
**Death from MI (3)**	I22	Subsequent myocardial infarction
**Death from MI (3)**	I23	Certain current complications following acute myocardial infarction

Office for National Statistics (ONS) prior to 2000 (ICD9)

**Category (code)**	ICD9 code	ICD9 term
**Death from myocardial infarction (3)**	410	Acute myocardial infarction
**Death from myocardial infarction (3)**	4110	Other acute and subacute forms of ischemic heart disease ; Postmyocardial infarction syndrome

**Death from myocardial infarction (3)**	4297	Ill-defined descriptions and complications of heart disease ; Certain sequelae of myocardial infarction, not elsewhere classified

Myocardial Ischaemia National Audit Project (MINAP)

1	Acute STEMI
2	Acute NSTEMI

Phenotype coding lists

CALIBER myocardial infarction phenotype
Fatal or non-fatal myocardial infarction (MI) as recorded in any of the constituent CALIBER data sources. The type of myocardial infarction (ST elevation MI, STEMI; or non ST elevation MI, NSTEMI) is recorded only in MINAP and CPRD.

Read codes used to identify myocardial infarction in primary care records (Clinical Research Practice Datalink)

Category	Clinical code	Clinical term	Datasource lookup
**STEMI (3)**	G30X000	Acute ST segment elevation myocardial infarction	12229
**NSTEMI (4)**	G307100	Acute non-ST segment elevation myocardial infarction	10562
**Acute MI not further specified (5)**	323..00	ECG: myocardial infarction	7783
**Acute MI not further specified (5)**	3233.00	ECG: antero-septal infarct.	26975
**Acute MI not further specified (5)**	3234.00	ECG:posterior/inferior infarct	26972
**Acute MI not further specified (5)**	3235.00	ECG: subendocardial infarct	55401
**Acute MI not further specified (5)**	3236.00	ECG: lateral infarction	52705
**Acute MI not further specified (5)**	323Z.00	ECG: myocardial infarct NOS	59032
**Acute MI not further specified (5)**	889A.00	Diab mellit insulin-glucose infus acute myocardial infarct	61670
**Acute MI not further specified (5)**	G30..00	Acute myocardial infarction	241
**Acute MI not further specified (5)**	G30..12	Coronary thrombosis	2491
**Acute MI not further specified (5)**	G30..13	Cardiac rupture following myocardial infarction (MI)	30421
**Acute MI not further specified (5)**	G30..15	MI - acute myocardial infarction	1677
**Acute MI not further specified (5)**	G30..16	Thrombosis - coronary	13571
**Acute MI not further specified (5)**	G300.00	Acute anterolateral infarction	12139
**Acute MI not further specified (5)**	G301.00	Other specified anterior myocardial infarction	5387
**Acute MI not further specified (5)**	G301000	Acute anteroapical infarction	40429
**Acute MI not further specified (5)**	G301100	Acute anteroseptal infarction	17872
**Acute MI not further specified (5)**	G301z00	Anterior myocardial infarction NOS	14897
**Acute MI not further specified (5)**	G302.00	Acute inferolateral infarction	8935
**Acute MI not further specified (5)**	G303.00	Acute inferoposterior infarction	29643
**Acute MI not further specified (5)**	G304.00	Posterior myocardial infarction NOS	23892
**Acute MI not further specified (5)**	G305.00	Lateral myocardial infarction NOS	14898

**Acute MI not further specified (5)**	G306.00	True posterior myocardial infarction	63467
**Acute MI not further specified (5)**	G307.00	Acute subendocardial infarction	3704
**Acute MI not further specified (5)**	G307000	Acute non-Q wave infarction	9507
**Acute MI not further specified (5)**	G308.00	Inferior myocardial infarction NOS	1678
**Acute MI not further specified (5)**	G309.00	Acute Q-wave infarct	30330
**Acute MI not further specified (5)**	G30B.00	Acute posterolateral myocardial infarction	32854
**Acute MI not further specified (5)**	G30X.00	Acute transmural myocardial infarction of unspecif site	29758
**Acute MI not further specified (5)**	G30y.00	Other acute myocardial infarction	34803
**Acute MI not further specified (5)**	G30y000	Acute atrial infarction	28736
**Acute MI not further specified (5)**	G30y100	Acute papillary muscle infarction	62626
**Acute MI not further specified (5)**	G30y200	Acute septal infarction	41221
**Acute MI not further specified (5)**	G30yz00	Other acute myocardial infarction NOS	46017
**Acute MI not further specified (5)**	G30z.00	Acute myocardial infarction NOS	14658
**Acute MI not further specified (5)**	G31y100	Microinfarction of heart	68357
**Acute MI not further specified (5)**	G38..00	Postoperative myocardial infarction	32272
**Acute MI not further specified (5)**	G380.00	Postoperative transmural myocardial infarction anterior wall	46112
**Acute MI not further specified (5)**	G381.00	Postoperative transmural myocardial infarction inferior wall	46276
**Acute MI not further specified (5)**	G384.00	Postoperative subendocardial myocardial infarction	41835
**Acute MI not further specified (5)**	G38z.00	Postoperative myocardial infarction, unspecified	68748
**Acute MI not further specified (5)**	Gyu3400	[X]Acute transmural myocardial infarction of unspecif site	96838

Hospital Episode Statistics

**Category (code)**	ICD10 code	ICD10 term
**Acute MI not further specified (5)**	I21	Acute myocardial infarction

OPCS

**Category (code)**	OPCS 4	OPCS 4 term
**Transluminal coronary thrombolysis (2)**	K50.2	Percutaneous transluminal coronary thrombolysis using streptokinase
**Transluminal coronary thrombolysis (2)**	K50.3	Percutaneous transluminal injection of therapeutic substance into coronary artery NEC

Office for National Statistics (ONS)

**Category (code)**	ICD10 code	ICD10 term
**Death from MI (3)**	I21	Acute myocardial infarction
**Death from MI (3)**	I22	Subsequent myocardial infarction
**Death from MI (3)**	I23	Certain current complications following acute myocardial infarction

Office for National Statistics (ONS) prior to 2000 (ICD9)

Category (code)	ICD9 code	ICD9 term
**Death from myocardial infarction (3)**	410	Acute myocardial infarction
**Death from myocardial infarction (3)**	4110	Other acute and subacute forms of ischemic heart disease ; Postmyocardial infarction syndrome
**Death from myocardial infarction (3)**	4297	Ill-defined descriptions and complications of heart disease ; Certain sequelae of myocardial infarction, not elsewhere classified

Myocardial Ischaemia National Audit Project (MINAP)

1	Acute STEMI
2	Acute NSTEMI

CALIBER heart failure phenotype

Primary care
1. Diagnosis of left ventricular dysfunction during echocardiogram during a consultation
2. Diagnosis of heart failure of valvular cause during a consultation
3. Diagnosis of heart failure due to hypertension during a consultation
4. Diagnosis of heart failure due to unspecified cause during a consultation
5. Diagnosis of heart failure not otherwise specified during a consultation

Secondary care
1. Diagnosis of heart failure due to hypertension as the primary diagnosis during a hospitalization
2. Diagnosis of heart failure not otherwise specified as the primary diagnosis during a hospitalization

Mortality
1. Hypertensive heart failure as the underlying cause of death
2. Heart failure not otherwise specified as the underlying cause of death

Read codes used to identify myocardial infarction in primary care records (Clinical Research Practice Datalink)

Category (code)	Clinical code	Clinical term	Datasource lookup
**Heart failure due to valvular disease (3)**	G580400	Congestive heart failure due to valvular disease	94870
**Heart failure due to hypertension (4)**	G210.00	Malignant hypertensive heart disease	50157
**Heart failure due to hypertension (4)**	G210000	Malignant hypertensive heart disease without CCF	95334
**Heart failure due to hypertension (4)**	G210100	Malignant hypertensive heart disease with CCF	72668
**Heart failure due to hypertension (4)**	G211100	Benign hypertensive heart disease with CCF	52127
**Heart failure due to hypertension (4)**	G21z100	Hypertensive heart disease NOS with CCF	62718
**Heart failure due to hypertension (4)**	G230.00	Malignant hypertensive heart and renal disease	67232
**Heart failure due to hypertension (4)**	G232.00	Hypertensive heart&renal dis wth (congestive) heart failure	21837
**Heart failure due to hypertension (4)**	G234.00	Hyperten heart&renal dis+both(congestv)heart and renal fail	57987
**Heart failure due to other cause (5)**	G1yz100	Rheumatic left ventricular failure	22262
**Heart failure NOS (6)**	1O1..00	Heart failure confirmed	9913
**Heart failure NOS (6)**	662W.00	Heart failure annual review	30779
**Heart failure NOS (6)**	662p.00	Heart failure 6 month review	83502
**Heart failure NOS (6)**	8B29.00	Cardiac failure therapy	24503
**Heart failure NOS (6)**	8H2S.00	Admit heart failure emergency	32898
**Heart failure NOS (6)**	9Or0.00	Heart failure review completed	19380
**Heart failure NOS (6)**	G400.00	Acute cor pulmonale	8464
**Heart failure NOS (6)**	G41z.11	Chronic cor pulmonale	5695
**Heart failure NOS (6)**	G554000	Congestive cardiomyopathy	5141
**Heart failure NOS (6)**	G554011	Congestive obstructive cardiomyopathy	68766
**Heart failure NOS (6)**	G58..00	Heart failure	2062
**Heart failure NOS (6)**	G58..11	Cardiac failure	1223
**Heart failure NOS (6)**	G580.00	Congestive heart failure	398

**Heart failure NOS (6)**	G580.11	Congestive cardiac failure	2906
**Heart failure NOS (6)**	G580.12	Right heart failure	10079
**Heart failure NOS (6)**	G580.13	Right ventricular failure	10154
**Heart failure NOS (6)**	G580.14	Biventricular failure	9524
**Heart failure NOS (6)**	G580000	Acute congestive heart failure	23707
**Heart failure NOS (6)**	G580100	Chronic congestive heart failure	32671
**Heart failure NOS (6)**	G580200	Decompensated cardiac failure	27884
**Heart failure NOS (6)**	G580300	Compensated cardiac failure	11424
**Heart failure NOS (6)**	G581.00	Left ventricular failure	884
**Heart failure NOS (6)**	G581.11	Asthma - cardiac	23481
**Heart failure NOS (6)**	G581.13	Impaired left ventricular function	5942
**Heart failure NOS (6)**	G581000	Acute left ventricular failure	5255
**Heart failure NOS (6)**	G582.00	Acute heart failure	27964
**Heart failure NOS (6)**	G58z.00	Heart failure NOS	4024
**Heart failure NOS (6)**	G58z.12	Cardiac failure NOS	17278
**Heart failure NOS (6)**	G5yy900	Left ventricular systolic dysfunction	8966
**Heart failure NOS (6)**	G5yyA00	Left ventricular diastolic dysfunction	12550
**Heart failure NOS (6)**	R2y1000	[D]Cardiorespiratory failure	20324
**Left ventricular dysfunction (3)**	585f.00	Echocardiogram shows left ventricular systolic dysfunction	11284
**Left ventricular dysfunction (3)**	585g.00	Echocardiogram shows left ventricular diastolic dysfunction	11351

Hospital Episode Statistics

**Category (code)**	**ICD10 code**	**ICD10 term**
**HF - hypertension cause (4)**	I110	Hypertensive heart disease with (congestive) heart failure
**HF - hypertension cause (4)**	I130	Hypertensive heart and renal disease with (congestive) heart failure
**HF - hypertension cause (4)**	I132	Hypertensive heart and renal disease with both (congestive) heart failure and renal failure
**HF - other unspecified cause (5)**	I260	Pulmonary embolism with mention of acute cor pulmonale
**HF - not otherwise specified (6)**	I50	Heart failure

Office for National Statistics (ONS)

**Category (code)**	**ICD10 code**	**ICD10 term**
**Hypertensive heart failure (4)**	I110	Hypertensive heart disease with (congestive) heart failure
**Hypertensive heart failure (4)**	I130	Hypertensive heart and renal disease with (congestive) heart failure
**Hypertensive heart failure (4)**	I132	Hypertensive heart and renal disease with both (congestive) heart failure and renal failure
**Heart failure with other specified cause (5)**	I260	Pulmonary embolism with mention of acute cor pulmonale
**Heart failure with unspecified cause (6)**	I50	Heart failure

Office for National Statistics (ONS) prior to 2000 (ICD9)

**Category (code)**	**ICD9 code**	**ICD9 term**
**Heart failure with other specified cause (5)**	4151	Acute pulmonary heart disease ; Pulmonary embolism and infarction
**Heart failure with unspecified cause (6)**	428	Heart failure

Myocardial Ischaemia National Audit Project (MINAP)

1	CCF

CALIBER baseline definitions
See also: https://www.caliberresearch.org

Definitions used for smoking at baseline
CPRD:
smoking_cessation_gprd
Prescription of smoking cessation medication in primary care. Currently lookup is performed from using BNF chapter 4.10.2.
smoking_status_gprd
An individual record of smoking status in primary care. A patient may have multiple records of smoking status.
HES:
smoking_status_hes
Smoking status recorded in HES. Currently there are only ICD-10 codes for smoking, no codes for ex-smoking or non-smoking.

Definitions used for hypertension at baseline
CPRD:
ht_gprd
Diagnosis of hypertension
HES:
ht_hes
Hypertension diagnosis recorded in secondary care.

Definitions used for diabetes at baseline
CPRD:
dm_gprd
Type of diabetes diagnosis at a particular point in time.
diabdiag_gprd
GP diagnosis of diabetes mellitus using a specific type I or type II diagnosis in the Read diagnosis chapter (i.e. codes starting with C08, C09, C10E, C10F). Vague terms such as `juvenile onset diabetes` or `insulin dependent diabetes` are not included.
HES:
dm_hes
Diagnosis of diabetes in secondary care (HES).

Supplementary Methods

Ethical approval
CALIBER was approved by the Lewisham Local Research Ethics Committee (ref: 09/H0810/16 date: 08/04/2009) and the Ethics and Confidentiality Committee (ECC) (ref: ECC 2-06(b)/2009 CALIBER dataset). CALIBER has been registered with the University College London Data Protection Officer (ref: Z6364106/2009/2/26). CALIBER EHR data are anonymised; individual informed consent was not sought from study participants.

Multiple imputation algorithm
Multiple imputation was implemented using the 'mice' package for R, to replace missing values in prognostic covariates (body mass index, smoking, systolic and diastolic blood pressure).[1] Imputation models included general practice and 3-year periods from 1998 to 2010 of index myocardial infarction (MI) and:

1. All the baseline covariates as used in the multivariable analysis (age, sex, index of multiple deprivation, hypertension, diabetes, type of MI, body mass index, smoking, systolic blood pressure, diastolic blood pressure);
2. Baseline measurements of covariates not considered in the multivariable analysis (alcohol intake, haemoglobin, white blood cell count, creatinine, alanine aminotransferase (ALAT), total cholesterol, HDL cholesterol);
3. Baseline medications prior to index MI (antiplatelet, oral anticoagulant, statin, ACE inhibitor, angiotensin receptor blocker (ARB), beta-blocker, calcium channel blocker, loop diuretic, aldosterone antagonist, digoxin);
4. Coexisting medical conditions (history of atrial fibrillation, peripheral arterial disease, depression, cancer, chronic obstructive pulmonary disease);
5. The Nelson-Aalen hazard and the event status for the outcomes analysed.

Missing data was multiply imputed and across 20 datasets a (stratified) multivariable Cox regression model was fit and results were combined using Rubin's rules.[2]

References

1. van Buuren S. Multiple imputation of discrete and continuous data by fully conditional specification. Stat Methods Med Res 2007;16:219-42.
2. Carpenter JR, Kenward MG. *Multiple Imputation and its Application*: Wiley; 2013.

Table S1 - Hazard ratios for heart failure in patients following a first myocardial infarction using the competing risks regression based on Fine and Gray's proportional subhazards model to account for the competing risk of all cause mortality

	Model 1	Model 2	Model 3	Model 4
	HR (95% CI)	HR (95% CI)	HR (95% CI)	HR (95% CI)
Age, per 10 years increase	1.41 (1.38 – 1.44)	1.39 (1.36 – 1.42)	1.36 (1.33 – 1.39)	1.35 (1.32 – 1.38)
Men	1.04 (0.99 – 1.10)	1.07 (1.01 – 1.13)	1.06 (1.00 – 1.12)	1.05 (1.00 – 1.11)
Index of multiple deprivation		overall p = <0.001	overall p = <0.001	overall p = <0.001
Q1		Reference	Reference	Reference
Q2		1.11 (1.03 – 1.21)	1.11 (1.02 – 1.20)	1.10 (1.01 – 1.20)
Q3		1.21 (1.12 – 1.32)	1.20 (1.11 – 1.31)	1.19 (1.09 – 1.29)
Q4		1.24 (1.14 – 1.35)	1.24 (1.14 – 1.34)	1.22 (1.12 – 1.32)
Q5		1.36 (1.25 – 1.47)	1.35 (1.25 – 1.47)	1.32 (1.21 – 1.43)
History of hypertension		1.15 (1.09 – 1.22)	1.13 (1.07 – 1.19)	1.14 (1.08 – 1.21)
History of diabetes		1.39 (1.29 – 1.49)	1.38 (1.29 – 1.48)	1.36 (1.27 – 1.47)
History of atrial fibrillation			1.50 (1.40 – 1.61)	1.49 (1.39 – 1.60)
Type of MI				
STEMI			1.10 (1.03 – 1.18)	1.11 (1.03 – 1.18)
History of peripheral arterial disease				1.29 (1.19 – 1.40)
History of COPD				1.18 (1.09 – 1.29)
ACE inhibitor use before MI				1.01 (0.95 – 1.08)
Angiotensin receptor blocker (ARB) use before MI				0.94 (0.85 – 1.05)
Beta-blocker use before MI				0.94 (0.88 – 1.00)

Complete case analysis with the addition of clinically relevant variables per model (n = 24,479, n events = 5,775). Overall p-values are shown for categorical covariates. ACE=Angiotensin-converting enzyme; CI=confidence interval; COPD=chronic obstructive pulmonary disease; MI=myocardial infarction; NSTEMI=non-ST-segment-elevation myocardial infarction; STEMI=ST-segment-elevation myocardial infarction; Q=quintile; HR=ratio of the subdistribution hazards.

Table S2 - Hazard ratios for heart failure in patients following a first myocardial infarction in sensitivity analyses

	Model 5<br>Cox regression<br>complete case without multiple imputation<br>n = 4,898<br>n events = 1,159	Model 5<br>Cox regression<br>with multiple imputation<br>n = 24,479<br>n events = 5,775	Model 5<br>Competing risk analysis<br>with complete case<br>n = 4,898<br>n events = 1,159
	HR (95% CI)	HR (95% CI)	HR (95% CI)
Age, per 10 years increase	1.48 (1.38 – 1.60)	1.47 (1.43 – 1.51)	1.44 (1.35 – 1.54)
Men	1.03 (0.89 – 1.19)	1.07 (1.01 – 1.13)	1.07 (0.95 – 1.22)
Body mass index (BMI in kg/m^2)	p = 0.005	p = <0.001	p = < 0.001
Underweight (18.5 <)	0.78 (0.48 – 1.32)	0.76 (0.58 – 0.99)	0.71 (0.45 – 1.12)
Normal (18.5; 25)	Reference	Reference	Reference
Overweight (25, 30)	0.84 (0.71 – 0.99)	0.84 (0.76 – 0.92)	0.84 (0.73 – 0.97)
Obese (>30)	1.12 (0.94 – 1.34)	0.98 (0.90 – 1.08)	1.17 (1.00 – 1.36)
Index of multiple deprivation	p = 0.040	p < 0.001	p = < 0.001
Q1	Reference	Reference	Reference
Q2	1.22 (0.94 – 1.58)	1.08 (0.98 – 1.19)	1.35 (1.10 – 1.66)
Q3	1.34 (1.03 – 1.75)	1.17 (1.06 – 1.30)	1.40 (1.14 – 1.71)
Q4	1.23 (0.93 – 1.63)	1.16 (1.05 – 1.29)	1.31 (1.07 – 1.61)
Q5	1.45 (1.09 – 1.92)	1.25 (1.12 – 1.40)	1.61 (1.33 – 1.96)
Smoking status	p = 0.792	p = 0.012	p = 0.952
Non-smoker	Reference	Reference	Reference
Current smoker	0.95 (0.77 – 1.14)	1.14 (1.04 – 1.25)	1.02 (0.86 – 1. 20)
Ex-smoker	0.98 (0.83 – 1.15)	1.03 (0.94 – 1.12)	0.99 (0.87 – 1.14)
History of hypertension	1.14 (0.96 – 1.35)	1.16 (1.09 – 1.24)	1.18 (1.02 – 1.37)
History of diabetes	1.28 (1.09 – 1.49)	1.46 (1.36 – 1.58)	1.23 (1.08 – 1.39)
History of atrial fibrillation	1.49 (1.25 – 1.76)	1.63 (1.51 – 1.75)	1.37 (1.18 – 1.59)
Type of MI			
STEMI	1.15 (0.97 – 1.37)	1.18 (1.10 – 1.27)	1.10 (0.95 – 1.28)
History of peripheral arterial disease	1.53 (1.25 – 1.87)	1.37 (1.25 – 1.50)	1.33 (1.13 – 1.57)
History of COPD	1.21 (0.99 – 1.48)	1.24 (1.15 – 1.38)	1.22 (1.03 – 1.44)
Systolic blood pressure, per 10 mmHg increase	0.99 (0.95 – 1.03)	1.00 (0.98 – 1.02)	1.01 (0.97 – 1.04)
Diastolic blood pressure, per 10 mmHg increase	1.01 (0.94 – 1.09)	0.99 (0.96 – 1.03)	0.99 (0.93 – 1.06)
ACE inhibitor use before MI	1.01 (0.87 – 1.17)	1.06 (0.99 – 1.14)	1.07 (0.94 – 1.21)
Angiotensin receptor blocker (ARB) use before MI	0.99 (0.95 – 1.03)	1.00 (0.90 – 1.12)	0.93 (0.78 – 1.12)
Beta-blocker use before MI	1.01 (0.94 – 1.09)	0.94 (0.88 – 1.00)	0.90 (0.79 – 1.02)

Using multivariable Cox regression without and with multiple imputation and the competing risks regression based on Fine and Gray's proportional subhazards model to account for the competing risk of all cause mortality (n = 4898, n events = 1159). Additional prognostic covariates: body mass index, smoking, systolic and diastolic blood pressure. Overall p-values are shown for categorical covariates. HR=hazard ratio; CI=confidence interval; MI=myocardial infarction; NSTEMI=non-ST-segment-elevation myocardial infarction; STEMI=ST-segment-elevation myocardial infarction; COPD=chronic obstructive pulmonary disease; ACE=Angiotensin-converting enzyme; Q=quintile.

Table S3 - Hazard ratios for heart failure in patients following a first myocardial infarction stratified on having a previous cancer diagnosis or not.

	Model 4<br>**Subjects without a history of cancer**<br>n = 21,719<br>n events = 5,029	Model 4<br>**Subjects with a history of cancer**<br>n = 2,760<br>n events = 746
	HR (95% CI)	HR (95% CI)
Age, per 10 years increase	1.49 (1.45 – 1.53)	1.45 (1.30 – 1.61)
Men	1.08 (1.01 – 1.15)	1.13 (0.93 – 1.37)
Index of multiple deprivation	overall p = <0.001	overall p = <0.001
Q1	Reference	Reference
Q2	1.08 (0.97 – 1.20)	1.07 (0.77 – 1.48)
Q3	1.17 (1.05 – 1.31)	1.27 (0.91 – 1.78)
Q4	1.17 (1.04 – 1.31)	1.14 (0.79 – 1.66)
Q5	1.25 (1.10 – 1.41)	1.43 (0.97 – 2.11)
History of hypertension	1.11 (1.03 – 1.18)	1.45 (1.16 – 1.81)
History of diabetes	1.53 (1.41 – 1.66)	1.19 (0.91 – 1.55)
History of atrial fibrillation	1.62 (1.49 – 1.76)	1.67 (1.33 – 2.09)
Type of MI		
STEMI	1.18 (1.09 – 1.28)	1.01 (0.77 – 1.31)
History of peripheral arterial disease	1.39 (1.26 – 1.54)	1.19 (0.88 – 1.62)
History of COPD	1.30 (1.18 – 1.43)	1.36 (1.03 – 1.81)
ACE inhibitor use before MI	1.11 (1.03 – 1.20)	1.00 (0.79 – 1.26)
Angiotensin receptor blocker (ARB) use before MI	1.06 (0.93 – 1.20)	0.92 (0.65 – 1.31)
Beta-blocker use before MI	0.97 (0.90 – 1.04)	0.99 (0.79 – 1.24)

All analyses are based on a complete case analysis. Overall p-values are shown for categorical covariates. ACE=Angiotensin-converting enzyme; CI=confidence interval; COPD=chronic obstructive pulmonary disease; MI=myocardial infarction; NSTEMI=non-ST-segment-elevation myocardial infarction; STEMI=ST-segment-elevation myocardial infarction; Q=quintile.

Table S4 - Hazard ratios for heart failure in patients following a first myocardial infarction stratified on a history of revascularization.

	Model 4 **Subjects without a history of revascularization** n = 21,160 n events = 5,222	Model 4 **Subjects with a history of revascularization** n = 3.319 n events = 553
	HR (95% CI)	HR (95% CI)
Age, per 10 years increase	1.48 (1.44 – 1.52)	1.30 (1.17 – 1.44)
Men	1.07 (1.01 – 1.14)	0.96 (0.75 – 1.23)
Index of multiple deprivation	overall p = <0.001	overall p = <0.001
Q1	Reference	Reference
Q2	1.06 (0.95 – 1.17)	1.13 (0.80 – 1.61)
Q3	1.14 (1.02 – 1.27)	1.39 (0.95 – 2.03)
Q4	1.15 (1.02 – 1.28)	1.34 (0.85 – 2.02)
Q5	1.22 (1.08 – 1.38)	1.29 (0.84 – 1.99)
History of hypertension	1.14 (1.07 – 1.22	1.18 (0.92 – 1.50)
History of diabetes	1.49 (1.37 – 1.61)	1.32 (1.00 – 1.75)
History of atrial fibrillation	1.61 (1.48 – 1.74)	1.90 (1.39 – 2.58)
Type of MI		
STEMI	1.14 (1.05 – 1.23)	1.39 (1.07 – 1.79)
History of peripheral arterial disease	1.40 (1.27 – 1.55)	1.22 (0.88 – 1.62)
History of COPD	1.30 (1.18 – 1.43)	1.36 (0.92 – 2.03)
ACE inhibitor use before MI	1.11 (1.03 – 1.19)	1.15 (0.89 – 1.49)
Angiotensin receptor blocker (ARB) use before MI	1.06 (0.94 – 1.19)	0.79 (0.54 – 1.17)
Beta-blocker use before MI	1.00 (0.94 – 1.07)	0.84 (0.65 – 1.08)

All analyses are based on a complete case analysis. Overall p-values are shown for categorical covariates. ACE=Angiotensin-converting enzyme; CI=confidence interval; COPD=chronic obstructive pulmonary disease; MI=myocardial infarction; NSTEMI=non-ST-segment-elevation myocardial infarction; STEMI=ST-segment-elevation myocardial infarction; Q=quintile.

Supplementary Figure 1 – Cumulative incidence curves for HF accounting for mortality as first event after index MI

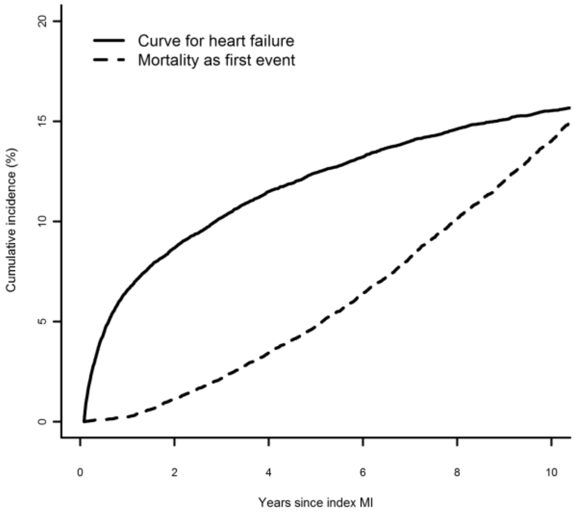

N at risk	21663	14293	9809	6236	3489	1504
Cum HF events	0	1902	602	351	267	155
Cum ACM events	0	292	635	814	990	971

Cumulative incidence curves adjusted for competing risk of mortality as first event. 10-year follow-up in patients who survived the first 30 days and did not develop HF during the first 30 days (30 days event free).
