# Supplementary material for: An electronic health records cohort study on heart failure following myocardial infarction in England: incidence and predictors
Source: BMJ Open. 2018 Mar 3;8(3):e018331. doi: 10.1136/bmjopen-2017-018331 (PMC5855447; doi:10.1136/bmjopen-2017-018331)
Supplement: Author's manuscript [file bmjopen-2017-018331.draft_revisions.pdf]

BMJ Open is committed to open peer review. As part of this commitment we make the peer review history of every article we publish publicly available.

When an article is published we post the peer reviewers' comments and the authors' responses online. We also post the versions of the paper that were used during peer review. These are the versions that the peer review comments apply to.

The versions of the paper that follow are the versions that were submitted during the peer review process. They are not the versions of record or the final published versions. They should not be cited or distributed as the published version of this manuscript.

BMJ Open is an open access journal and the full, final, typeset and author-corrected version of record of the manuscript is available on our site with no access controls, subscription charges or pay-per-view fees (http://bmjopen.bmj.com).

If you have any questions on BMJ Open's open peer review process please email editorial.bmjopen@bmj.com

BMJ Open

# An Electronic Health Records study on Heart Failure Following Myocardial Infarction: Incidence and Prognostic Factors


| | |
|---|---|
| Journal: | *BMJ Open* |
| Manuscript ID | bmjopen-2017-018331 |
| Article Type: | Research |
| Date Submitted by the Author: | 21-Jun-2017 |
| Complete List of Authors: | Gho, Johannes; Universitair Medisch Centrum Utrecht, Cardiology<br>Schmidt, Amand; Faculty of Science and Engineering, Groningen Research Institute of Pharmacy<br>Pasea, Laura; Farr Institute<br>Koudstaal, Stefan; Farr Institute<br>Pujades Rodriguez, Mar; Farr Institute<br>Denaxas, S; University College London, Clinical Epidemiology Group<br>Shah, Anoop; UCL, Farr Institute of Health Informatics research<br>Patel, Riyaz; Farr Institute of Health Informatics Research, University College London, Epidemiology and Public Health; Bart's Heart Centre, Cardiology<br>Gale, Chris; University of Leeds, Division of Epidemiology and Biostatistics<br>Hoes, Arno; Julius Center for Health Sciences and Primary Care, University Medical Center, Utrecht<br>Cleland, J; National Heart and Lung Institute,<br>Hemingway, Harry; UCL, Epidemiology and Public Health<br>Asselbergs, Folkert; University Medical Centre Utrecht, Department of Cardiology; ICIN-Netherlands Heart Institute, Durrer Center for Cardiogenetic Research |
| <b>Primary Subject Heading</b>: | General practice / Family practice |
| Secondary Subject Heading: | Cardiovascular medicine, Epidemiology, General practice / Family practice, Health informatics, Public health |
| Keywords: | Heart failure < CARDIOLOGY, Myocardial infarction < CARDIOLOGY, electronic health records |
| | |


SCHOLARONE™
Manuscripts

# An Electronic Health Records study on Heart Failure Following Myocardial Infarction: Incidence and Prognostic Factors

Johannes M.I.H. Gho, MD, PhD,[1,2,*] Amand F. Schmidt, PhD,[2,3,4*] Laura Pasea, MSc,[1] Stefan Koudstaal, MD, MSc, PhD,[1,2] Mar Pujades-Rodriguez, PhD,[1,5] Spiros Denaxas, PhD,[1] Anoop D. Shah, MRCP,[1] Riyaz S. Patel, MD, PhD,[1,3] Chris P. Gale, PhD, FRCP,[6] Arno W. Hoes, MD, PhD,[7] John G. Cleland, MD, FRCP,[8] Harry Hemingway, FFPH, FRCP,[1] Folkert W. Asselbergs, MD, PhD,[1,2,3,9]

[1]Farr Institute of Health Informatics Research, UCL Institute of Health Informatics, University College London, London, UK
[2]Department of Cardiology, Division Heart and Lungs, University Medical Center Utrecht, Utrecht, the Netherlands
[3]Institute of Cardiovascular Science, Faculty of Population Health Sciences, University College London, London, UK
[4]Groningen Research Institute of Pharmacy, University of Groningen, Groningen, the Netherlands
[5]Medical Research Council Bioinformatics Centre, Leeds Institute of Biomedical and Clinical Sciences, University of Leeds, Leeds, UK
[6]Medical Research Council Bioinformatics Centre, Leeds Institute for Cardiovascular and Metabolic Medicine, University of Leeds, Leeds, UK
[7]Julius Centre for Health Sciences and Primary Care, University Medical Center Utrecht, Utrecht, the Netherlands
[8]National Heart & Lung Institute, Royal Brompton & Harefield Hospitals, Imperial College London, London, UK
[9]Durrer Center for Cardiogenetic Research, ICIN-Netherlands Heart Institute, Utrecht, the Netherlands
* Shared first author

**Corresponding author:**
A Floriaan Schmidt
Institute of Cardiovascular Science, University College London
222 Euston Road, London NW1 2DA, United Kingdom
E-mail: amand.schmidt@ucl.ac.uk; a.f.schmidt@rug.nl
Tel: 0044 (0)20 3549 5625

**Word count text**: 2560
**Word count abstract**: 226
**Number of references**: 24
**Number of tables**: 2
**Number of figures**: 3
**(Web)appendix**: 1

For peer review only

**Abstract**

**Objectives** To investigate the incidence and prognostic factors of heart failure (HF) following a myocardial infarction (MI).

**Methods** Data was used from the CALIBER programme, linking electronic health records in England from primary care, hospital admissions, an MI registry and mortality data. Subjects were eligible if they were 18 years or older, did not have a history of HF, and survived a first MI. Factors associated with time to first HF were examined using Cox proportional hazard models.

**Results** Of the 24,479 MI patients 5,775 (23.6%) developed HF during a median follow-up of 3.7 years (incidence rate per 1000 person-years 63.8 (95% CI: 62.2 – 65.5). Baseline characteristics significantly associated with HF were: atrial fibrillation [HR 1.62, 95% CI 1.51 – 1.75], age [per 10 years increase: 1.45, 1.41 – 1.49], diabetes [1.45, 1.35 – 1.56], peripheral arterial disease [1.38, 1.26 – 1.51], COPD [1.28, 1.17 – 1.40], greater socioeconomic deprivation [5$^{th}$ vs. 1$^{st}$ quintile 1.27, 1.13 – 1.41], ST-segment elevation MI at presentation [1.19, 1.11 – 1.27], hypertension [1.16, 1.09 – 1.23], currently smoking [1.14, 1.04 – 1.25] and BMI [underweight 0.76, 0.58 – 0.99, overweight 0.84, 0.76 – 0.92 both vs normal weight]. Results were robust to various sensitivity analyses such as competing risk analyses and multiple imputation.

**Conclusion** In England one in four survivors of a first MI developed HF within four years, which was related to baseline patient characteristics at time of first MI.

**Keywords:** heart failure; myocardial infarction; incidence; risk factors; electronic health records

For peer review only

**Strengths and limitations of this study**

- ❖ The current study provides a contemporary description of the burden of heart failure in a representative sample of first time myocardial infarction patient included in the CALIBER, an UK based electronic health care database. Multivariable time to event models were used to identify independent prognostic variables for the occurrence of heart failure following a first myocardial infraction event
- ❖ Robust methods were used to account for potential calendar effect and competing risks due to all-cause mortality.
- ❖ While most patient characteristics (e.g., disease history and drug prescriptions) were completely recorded, information on BMI, smoking and blood pressure was missing for 70% to 35% of the patients. Despite multiply imputing missing data, results for these variables should be carefully interpreted.
- ❖ Further potential limitations include the reliance on ICD codes for disease classification instead of clinical criteria. Finally, a small proportion of subjects may have had an earlier myocardial infraction or heart failure event which was not recorded in the CALIBER due to left truncation of follow-up time.

**Abbreviations list:** heart failure (HF); myocardial infarction (MI); ST-segment elevation MI (STEMI); hazard ratio (HR); confidence interval (CI); electronic health records (EHRs); chronic obstructive pulmonary disease (COPD); body mass index (BMI).

For peer review only


For peer review only - http://bmjopen.bmj.com/site/about/guidelines.xhtml

## Introduction

Research describing the incidence of heart failure (HF) following myocardial infarction (MI) is limited, mainly originating from the thrombolytic era, often using small sample sizes and contradicting, making it difficult to provide evidence based medicine. For example, a previous UK study among almost 900 patients hospitalised with MI in 1998, found that one fifth developed HF during their hospital stay and a further third following hospital discharge(1). More recently, a Swedish study found an incidence of HF during the index MI hospitalization of 28% in 2008(2). Finally, a Danish nationwide cohort study reported an incidence of HF at 90-days following MI of 19.6% in 2009-10(3). Differences between these studies could be related to a number of factors, for example calendar time related change in treatment or national policies, all of which potentially limits generalizability of results. In the current paper we used a large and representative sample based on electronic health records (EHRs) from primary and hospital care to 1) describe the current incidence of HF after MI and 2) explore prognostic patient characteristics of post MI HF(4,5).

## Methods

Study design

Data was used from the CALIBER (CArdiovascular disease research using LInked Bespoke studies and Electronic health Records) programme dataset, which included linked data from (i) primary care EHRs, with diagnoses coded using the Read system (Clinical Practice Research Datalink, CPRD) from 244 consenting general practices(6), (ii) hospital care administrative records, information coded using the International Classification of Diseases (ICD) and the Office of Population Censuses and Surveys Classification of Interventions and Procedures (OPCS-4), Hospital Episode Statistics (HES), the Myocardial Ischaemia National Audit Project (MINAP) and (iii) the mortality register (from UK Office for National Statistics, ONS) (7).

Setting and participants



For peer review only - http://bmjopen.bmj.com/site/about/guidelines.xhtml

Patients were eligible if ≥18 years old, registered in CPRD practices in England consenting to data linkage, with at least one year of up-to-standard pre-study follow-up (meeting CPRD practice quality standards), and experiencing a first MI between 1st January 1998 to 25th March 2010 (4). The first record of MI across the linked data sources was considered as the index event and subsequent MI records within 30 days from the same or other data sources were considered as representing the same event. Patients with a fatal index MI or a history of HF before their index MI were excluded. Patients were censored if they ceased to be registered at a participating general practice, the date of death or the administrative censoring of the dataset (25th March 2010).

Variables and data sources

Study variables were derived from Read, ICD-9 or ICD-10, MINAP, Multilex drug or procedure codes, and the EHR phenotyping algorithms can be found online (https://caliberresearch.org/portal)(7). For continuous variables, the most recent measurement recorded in CPRD in the year before study entry was used as the baseline value. Data before study entry were used to determine prognostic factors, including age (years, using the date of birth), sex, ethnicity, social deprivation (Index of Multiple Deprivation, IMD score as recorded in CPRD in quintiles), smoking, alcohol use, history of cardiovascular disease, previous coronary revascularization, history of diabetes, history of thyroid disease, history of chronic obstructive pulmonary disease (COPD), history of depression and history of non-metastatic cancer. Further collected data in the year before study entry were body mass index (BMI), vital signs before hospitalisation, biomarkers (including haemoglobin, white blood cell count, platelets, erythrocyte sedimentation rate, creatinine, alanine aminotransferase (ALAT), glucose concentration and cholesterol levels) and medications prescribed before index MI. The indexing MI event was characterized as ST-segment-elevation myocardial infarction (STEMI), or non-STEMI (NSTEMI), site of infarction, mode and timing of reperfusion and peak cardiac biomarkers (troponin I, troponin T and CK). The primary study outcome was the first HF event following MI.

Statistical methods

For peer review only



For peer review only - http://bmjopen.bmj.com/site/about/guidelines.xhtml

Incidence rates (cases per 1000 person-years) and Kaplan-Meier cumulative incidence of HF were estimated, with Kaplan-Meier curves stratified by age (<50, 50-65, ≥65 years) and type of MI.

The associations of clinically relevant baseline variables with the onset of HF following MI were explored using Cox proportional hazard models. The proportional hazards assumption was checked based on the Schoenfeld residuals and by nonparametric correlation coefficients between survival time and the Schoenfeld residuals. All models were stratified for general practice and calendar year periods between 1998 to 2010 (1998-2001, 2001-2004, 2004-2007 and 2007-2010). Stratified Cox models were used to correct for clustering of observations by calendar period and practice. Stratified models were used instead of frailty models because the former does not make any distributional assumption. Models were sequentially adjusted for: 1) age and sex, 2) cardiovascular risk factors, 3) type of MI, and 4) co-morbidities and prescribed medication. Associations are presented as hazard ratio's (HRs) with 95% confidence intervals (CI). Analyses were performed using R (version 3.3.2)(8).

Sensitivity analyses

Missing data was most predominant for smoking, BMI, and systolic and diastolic blood pressure measurements (see Table 1) which were multiply imputed using the mice package and additionally included to the multivariable regression models as a sensitive analysis. Multiple imputation was implemented using 20 imputed datasets which were pooled using Rubin's rules (Supplementary)(9). To account for the fact that patients may have died before the onset of HF (e.g., competing risk by mortality), analyses were repeated using Fine and Gray models(10,11).

Ethical approval

CALIBER has received ethics approval (Supplementary). This study is in compliance with the Declaration of Helsinki and was approved by the ISAC (Independent Scientific Advisory Committee) for MHRA Database Research (protocol no. 14_198R), the MINAP Academic Group and is registered with ClinicalTrials.gov (NCT02384213).

For peer review only



For peer review only - http://bmjopen.bmj.com/site/about/guidelines.xhtml

**Results**

Subjects and baseline characteristics

52,770 patients experienced a first MI during the study period (Figure 1). Excluding patients with a fatal index MI (n = 15,104), a prior history of HF (n = 3,876), missing information on the type of MI (n = 97) and subjects with less than 1 year of follow-up prior to the indexing MI event (n = 9,214), resulted in a final set of 24,701. This cohort comprised of 4,657 STEMI patients, 19,822 NSTEMI subjects, 15,969 (65.2%) were men, 4,538 (42.1%) currently smoked, 12,258 (50.1%) had hypertension and 3,014 (12.3%) had a history of diabetes at baseline. In total, 6,129 (25.0%) were prescribed beta-blockers and 5,039 (20.6%) angiotensin converting enzyme (ACE) inhibitors prior to their indexing MI (Table 1).

Incidence

Patients contributed 90,482 person years of follow-up. During an median follow-up time of 3.7 years (IQR: 1.5; 6.7), 5,775patients (23.6%) developed HF. The crude incidence rate of HF following a first MI was 63.8 (95% CI: 62.2 – 65.5) per 1000 person-years. Within the first 30 days post MI follow-up 2,438 (10.0%) patients developed HF (Figure 2). From day 30 onwards 3,337 (15.8%) of MI patients (event free during the first 30 days) developed HF (Figure 2). The incidence of HF during the first 30-days of follow-up was 4.3% (102) in patients younger than 50 years, 6.0% (459) in 50-65 year olds and 12.9% (1877) in those 65 years and older, with HF incidence increasing proportionally as time progressed towards 5 years (Figure 3 top row). The 30-day incidence of HF was 9.5% (1892 events) for NSTEMI subjects and 11.7% (546 events) for STEMI subjects. Excluding patients who experienced an HF event within the first 30 days showed that STEMI patients had a lower incidence of HF than NSTEMI subjects (Figure 3 lower panels).

During the first 30 days of follow-up only 1 patient died, limiting the potential for competing risk by all-cause mortality. The Appendix Figure shows that the influences of all-cause mortality as a competing risk was similarly minor during the post 30-days follow-up. (Appendix Figure truncated at 5 years).

Prognostic factors after multivariable adjustments



For peer review only - http://bmjopen.bmj.com/site/about/guidelines.xhtml

Table 2 describes the results of the previously described nested Cox's models. Focusing on the final model, the hazard of HF increased with atrial fibrillation [HR 1.62, 95%CI 1.51 – 1.75], age [per 10 years increase: 1.45, 1.41 – 1.49], diabetes [1.45, 1.35 – 1.56], peripheral arterial disease [1.38, 1.26 – 1.51], COPD [1.28, 1.17 – 1.40], hypertension [1.16, 1.09 – 1.23], higher socioeconomic deprivation (p-value for the 5 groups < 0.001), and STEMI at presentation [1.19, 1.11 – 1.27)]. As described the proportional hazards assumption was evaluated both graphically and quantified using correlation coefficient, showing no violations after multivariable correction. Similar to the Kaplan-Meier analyses, accounting for competing risk by all-cause mortality had little impact on the presented results (Appendix Table S1). After multiply imputing the data models were extend to include smoking, BMI, and systolic and diastolic blood pressure variables which showed BMI, male gender and smoking to be prognostic factors. Importantly so the association of the previously described prognostic factor remained (Supplementary Table S2).

## Discussion

In this large population-based longitudinal study using linked EHRs, 23.6% of patients who survived a first MI developed HF during a median follow-up of 3.7 years, resulting in an incidence rate of 64 cases per 1000 person-years. Incident HF was associated with increasing age, higher socioeconomic deprivation, a history of diabetes, atrial fibrillation, peripheral arterial disease, COPD, STEMI at presentation, BMI and smoking.

### Prognostic factors

Similar to the current study, Torabi et al(14) reported that HF after MI increased steeply with age as well. A previous CALIBER study with linked EHRs among almost 90,000 patients with all-cause HF showed that HF predominantly affects the elderly and nearly two thirds of HF patients were uniquely captured in a single source, mainly in primary care, stressing the importance of using multiple linked data sources to obtain more accurate estimates of the prognostic HF burden(15). Socioeconomic deprivation has previously been shown to be an independent predictor of HF development and associated with an increased incidence of HF in MI-free subjects (16,17). Socioeconomic deprivation has also been


For peer review only - http://bmjopen.bmj.com/site/about/guidelines.xhtml

associated with more frequent hospital admission and higher mortality in patients with HF(18–20). The current study extends these observations to HF incidence after an MI. Furthermore, we showed that a history of diabetes, atrial fibrillation, peripheral arterial disease, COPD, STEMI at presentation, BMI and smoking are all independently prognostic of HF after MI. Interestingly, despite the size of the collected sample, gender only became significantly associated with HF after accounting for differences in BMI and smoking. Furthermore, the impact of male gender (HR of 1.07 95%CI 1.01 – 1.13) was modest indicating relative gender equality. It is of note that blood pressure was not significantly associated with HF. However, blood pressure measurements were frequently missing which (even after multiple imputation) may be the cause of the observed lack of association, hence this deserves independent exploration.

Strengths and limitations

The linkage of multiple EHR sources from primary and hospital care allowed for the collection of a representative sample(21). This enabled us to determine the prognostic value of routinely collected data in primary care records and to detect non-hospitalised HF cases, and estimates presented here can be considered representative of the intended patient population. A potential limitation is that the ascertainment of cardiovascular outcomes was not based on clinical criteria (e.g., validated questionnaires and properly conducted physical examinations), practices of medical coding will have changed over time and there could be subgroups of patients with left ventricular dysfunction without clinical symptoms. Calendar dependent changes over time were accounted for by using time period stratified Cox models. Similarly, based on the Kaplan-Meier plots there was evidence of violations of the proportional hazard assumption for subtype of MI, however these plots assume that the predictors are unrelated to other prognostic factors, which is known to be false. Using multivariable Cox models (again stratified for calendar period) and based on the, for correlation adjusted, Schoenfeld residuals we did not observe violations of the proportional hazard assumption. Residual confounding due to medication use might be a potential source of bias, however our intention was not to perform a causal analysis between drug prescriptions and our endpoint of incident HF. As such is relevant to note that despite the large sample size we did not observe a significant association of ACE, ARB or beta-blocker prescribed prior to the indexing MI event. We adhered to CPRD recommendations to obtain up-to-standard baseline data by



For peer review only - http://bmjopen.bmj.com/site/about/guidelines.xhtml

excluding patients with less than 1 year at CPRD practice prior to index MI. Assuming that duration of the CPRD follow-up is independent of the relations assessed here, excluding such patients should not hamper generalizability of results. Using the first MI recorded in the database without a prior history of HF, might have introduced bias due to left truncation (e.g., some subjects may already have experienced an MI before enrollment)(22). However CALIBER holds longitudinal records from primary and hospital care, making it unlikely a large part of the patients were misclassified as MI free and the one year of follow-up prior to entry further decreases the likelihood of misclassifying patients with prior MI(23). Due to our interest in HF occurrence after first MI selection bias (e.g., index event bias) was introduced(24). This index event bias does not impact the descriptive or prognostic value of the association presented, and is mostly relevant if one wants to develop an intervention based on the associations presented; this was not the aim of this study. Additionally, we note that we reduced the influence of selection bias by accounting for dependencies between predictors.

A final limitation is that there is a possible delay between primary and hospital care records, as we know from previous research that CPRD tends to record MI after HES or MINAP admission dates(4). The lower 30-day HF incidence in unclassified MI patients primarily derived from primary care could be partly explained because of a delay in coding. Therefore, we showed cumulative incidence rates in patients (alive and HF event free within the first 30 days) from 30 days after index MI to account for a delay in recording of MI in primary care. We were unable to differentiate between HF with preserved ejection fraction (HFpEF) and HF with reduced ejection fraction (HFrEF) as we had no access to detailed (echocardiographic) parameters to assess diastolic dysfunction, but it is likely that the majority of our HF patients had developed systolic dysfunction after MI.

Conclusion

In this large cohort study using linked EHRs in England from primary and hospital care about one in four people developed HF within a median of four years after surviving a first MI. Increasing age, higher socioeconomic deprivation, a history of hypertension, diabetes, atrial fibrillation, peripheral arterial

For peer review only

disease, COPD, smoking and STEMI at presentation were associated with new onset HF following MI, while the hazard of post MI HF was lower for overweight and underweight patients.

## Authors' contributions

JMIHG, AFS, LP, SK, MPR, SD, FWA, HM, JGC, AWH, CPG, RSP, ADS contributed to the idea and design of the study. JMIHG extracted and prepared the data for analysis. AFS and JMIHG performed the analysis. JMIHG drafted the manuscript, with revisions by AFS, LP, SK, MPR, SD, FWA, HM, JGC, AWH, CPG, RSP, ADS. JMIHG guaranties the quality and accuracy of the results presented.

## Competing interests

We have read and understood BMJ policy on declaration of interests and declare that we have no competing interests

## Data Sharing Statement

Data access can be requested via the CALIBER portal: https://www.ucl.ac.uk/health-informatics/caliber.

## Funding

This research received no specific grant from any funding agency in the public, commercial or not-for-profit sectors

For peer review only

## References

1. Torabi A., Cleland JGF., Khan NK., et al. The timing of development and subsequent clinical course of heart failure after a myocardial infarction. Eur Heart J 2008;29(7):859–70. Doi: 10.1093/eurheartj/ehn096.
2. Desta L., Jernberg T., Lofman I., et al. Incidence, temporal trends, and prognostic impact of heart failure complicating acute myocardial infarction. The SWEDEHEART Registry (Swedish Web-System for Enhancement and Development of Evidence-Based Care in Heart Disease Evaluated According to Recommended Therapies): a study of 199,851 patients admitted with index acute myocardial infarctions, 1996 to 2008. JACC Heart Fail 2015;3(3):234–42. Doi: 10.1016/j.jchf.2014.10.007.
3. Gjesing A., Gislason GH., Kober L., et al. Nationwide trends in development of heart failure and mortality after first-time myocardial infarction 1997-2010: A Danish cohort study. Eur J Intern Med 2014;25(8):731–8. Doi: 10.1016/j.ejim.2014.08.009.
4. Herrett E., Shah AD., Boggon R., et al. Completeness and diagnostic validity of recording acute myocardial infarction events in primary care, hospital care, disease registry, and national mortality records: cohort study. BMJ 2013;346:f2350.
5. Hripcsak G., Albers DJ. Next-generation phenotyping of electronic health records. J Am Med Inform Assoc 2013;20(1):117–21. Doi: 10.1136/amiajnl-2012-001145.
6. Herrett E., Gallagher AM., Bhaskaran K., et al. Data Resource Profile: Clinical Practice Research Datalink (CPRD). Int J Epidemiol 2015;44(3):827–36. Doi: 10.1093/ije/dyv098.
7. Denaxas SC., George J., Herrett E., et al. Data resource profile: Cardiovascular disease research using linked bespoke studies and electronic health records (CALIBER). Int J Epidemiol 2012;41(6):1625–38. Doi: 10.1093/ije/dys188.
8. Team RC. R: A Language and Environment for Statistical Computing. Vienna, Austria: R Foundation for Statistical Computing. www. R-project. org. A Lang Environ Stat Comput 2013.
9. Marshall A., Altman DG., Holder RL., Royston P. Combining estimates of interest in prognostic modelling studies after multiple imputation: current practice and guidelines. BMC Med Res Methodol 2009:57-.

For peer review only

10. Satagopan JM., Ben-Porat L., Berwick M., Robson M., Kutler D., Auerbach AD. A note on competing risks in survival data analysis. Br J Cancer 2004;91(7):1229–35. Doi: 10.1038/sj.bjc.6602102.

11. Gray B. cmprsk: Subdistribution Analysis of Competing Risks 2014.

12. Laut KG., Gale CP., Lash TL., Kristensen SD. Determinants and patterns of utilization of primary percutaneous coronary intervention across 12 European countries: 2003-2008. Int J Cardiol 2013;168(3):2745–53. Doi: 10.1016/j.ijcard.2013.03.085.

13. Chung SC., Sundstrom J., Gale CP., et al. Comparison of hospital variation in acute myocardial infarction care and outcome between Sweden and United Kingdom: population based cohort study using nationwide clinical registries. BMJ Br Med J 2015:h3913-.

14. Torabi A., Cleland JG., Rigby AS., Sherwi N. Development and course of heart failure after a myocardial infarction in younger and older people. J Geriatr Cardiol 2014;11(December 2005):1–12. Doi: 10.3969/j.issn.1671-5411.2014.01.002.

15. Koudstaal S., Pujades-Rodriguez M., Denaxas S., et al. Prognostic burden of heart failure recorded in primary care, acute hospital admissions, or both: A population-based linked electronic health record cohort study in 2.1 million people. Eur J Heart Fail 2016. Doi: 10.1002/ejhf.709.

16. McAlister FA., Murphy NF., Simpson CR., et al. Influence of socioeconomic deprivation on the primary care burden and treatment of patients with a diagnosis of heart failure in general practice in Scotland: population based study. BMJ 2004;328(7448):1110. Doi: 10.1136/bmj.38043.414074.EE.

17. Hawkins NM., Jhund PS., McMurray JJ V., Capewell S. Heart failure and socioeconomic status: Accumulating evidence of inequality. Eur J Heart Fail 2012;14(2):138–46. Doi: 10.1093/eurjhf/hfr168.

18. Philbin EF., Dec GW., Jenkins PL., DiSalvo TG. Socioeconomic status as an independent risk factor for hospital readmission for heart failure. Am J Cardiol 2001;87(12):1367–71.

19. Struthers AD., Anderson G., Donnan PT., MacDonald T. Social deprivation increases cardiac hospitalisations in chronic heart failure independent of disease severity and diuretic non-adherence. Heart 2000;83(1):12–6.


For peer review only



For peer review only - http://bmjopen.bmj.com/site/about/guidelines.xhtml

351 20. Pujades-Rodriguez M., Timmis A., Stogiannis D., et al. Socioeconomic deprivation and the incidence of 12 cardiovascular diseases in 1.9 million women and men: implications for risk prediction and prevention. PLoS One 2014;9(8):e104671. Doi: 10.1371/journal.pone.0104671.

354 21. Mann RD., Andrews EB. Pharmacovigilance: Second Edition. 2007.

355 22. Hazelbag CM., Klungel OH., van Staa TP., de Boer A., Groenwold RHH. Left truncation results in substantial bias of the relation between time-dependent exposures and adverse events. Ann Epidemiol 2015;25(8):590–6. Doi: 10.1016/j.annepidem.2015.03.019.

358 23. Lewis JD., Bilker WB., Weinstein RB., Strom BL. The relationship between time since registration and measured incidence rates in the General Practice Research Database. Pharmacoepidemiol Drug Saf 2005;14(7):443–51. Doi: 10.1002/pds.1115.

361 24. Dahabreh IJ., Kent DM. Index event bias as an explanation for the paradoxes of recurrence risk research. Jama 2011:822–3. Doi: 10.1001/jama.2011.163.

For peer review only

**Figure titles and legends**

**Figure 1 – Flowchart of study population**

CALIBER=Cardiovascular disease research using Linked Bespoke studies and Electronic health Records; CPRD=Clinical Practice Research Datalink; HF=heart failure; MI=myocardial infarction.

**Figure 2 – Incidence of HF after index MI**

Kaplan-Meier curves for crude incidence of HF after MI. **Left panel** 30-day follow-up after index MI. **Right panel** 5 year follow-up in patients who survived the first 30 days and did not develop HF during the first 30 days (30-days event free). HF=heart failure; MI=myocardial infarction.

**Figure 3 – Incidence of HF after index MI by group**

Kaplan-Meier curves for crude incidence of HF after MI stratified by group. **Top left panel:** 30-day follow-up after index MI stratified by age group. **Top right panel:** 5-year follow-up in 30-day event free patients stratified by age group. Log-rank test of ≥65 year old patients compared to patients aged 50-65 years ($p<0.001$). **Bottom left panel:** 30-day follow-up after index MI stratified by MI type. **Bottom right panel:** 5-year follow-up in patients who survived the first 30 days and did not develop HF during the first 30 days stratified by MI type. Log-rank test of NSTEMI compared to STEMI ($p<0.001$). HF=heart failure; MI=myocardial infarction.

For peer review only



For peer review only - http://bmjopen.bmj.com/site/about/guidelines.xhtml

Table 1 - Baseline characteristics at index myocardial infarction

| | STEMI patients n = 4,657 | NSTEMI patients n = 19,822 | Total MI patients n = 24,479 | Unknown (%) |
|---|---|---|---|---|
| Follow-up time (years), median (IQR) | 3.3 (1.4–5.7) | 3.9 (1.5–7.1) | 3.7 (1.5–6.7) | 0% |
| Mean age, years (SD) | 65.7 (13.2) | 68.7 (13.2) | 68.1 (13.2) | 0% |
| Male sex | 3311 (71.1%) | 12658 (63.9%) | 15969 (65.2%) | 0% |
| Ethnicity | | | | |
| White | 3466 (76.4%) | 14288 (76.1%) | 17754 (76.2%) | 4.8% |
| Asian | 83 (1.8%) | 327 (1.7%) | 410 (1.8%) | |
| Black | 20 (0.4%) | 63 (0.3%) | 83 (0.4%) | |
| Other | 968 (21.3%) | 4085 (21.7%) | 5053 (21.7%) | |
| Body mass index (BMI) | | | | |
| Underweight | 22 (1.7%) | 120 (2.0%) | 142 (1.9%) | 70.0% |
| Normal | 335 (25.5%) | 1661 (27.4%) | 1996 (27.2%) | |
| Overweight | 557 (43.0%) | 2431 (40.2%) | 2988 (40.7%) | |
| Obese | 381 (29.4%) | 1842 (30.4%) | 2223 (30.2%) | |
| Index of multiple deprivation | | | | |
| Most deprived quintile | 892 (19.2%) | 3985 (20.2%) | 4877 (20%) | 0.4% |
| | | | | |
| **Risk factors before index MI** | | | | |
| Current smoker | 1235 (51.1%) | 3303 (39.5%) | 4538 (42.1%) | 55.9% |
| Excess alcohol consumption | 55 (9.0%) | 219 (8.1%) | 274 (8.3%) | 86.4% |
| History of atrial fibrillation | 358 (7.7%) | 2210 (11.1%) | 2568 (10.5%) | 0% |
| History of hypertension | 2117 (45.5%) | 10141 (51.2%) | 12258 (50.1%) | 0% |
| History of peripheral arterial disease | 236 (5.1%) | 1475 (7.4%) | 1711 (7%) | 0% |
| Previous revascularization | | | | |
| PCI | 700 (15.0%) | 2044 (10.3%) | 2744 (11.2%) | 0% |
| CABG | 126 (2.7%) | 843 (4.3%) | 969 (4.0%) | 0% |
| Previous TIA | 154 (3.3%) | 998 (5.0%) | 1152 (4.7%) | 0% |
| Previous stroke | 80 (1.7%) | 410 (2.1%) | 490 (2.0%) | 0% |
| History of diabetes | 538 (11.6%) | 2476 (12.6%) | 3014 (12.3%) | 0% |
| History of thyroid disease | 237 (5.1%) | 1374 (6.9%) | 1661 (6.6%) | 0% |
| History of COPD | 304 (6.5%) | 1634 (8.2%) | 1938 (7.9%) | 0% |
| History of non-metastatic cancer | 478 (10.3%) | 2282 (11.5%) | 2760 (11.3%) | 0% |
| | | | | |
| **Vital signs before admission** median (IQR) | | | | |
| Systolic blood pressure, mmHg | 140 (130–153) | 140 (130–154) | 140 (130–154) | 34.2% |
| Diastolic blood pressure, mmHg | 80 (74–88) | 80 (71–88) | 80 (72–88) | 34.2% |
| | | | | |
| **Biomarkers,** median (IQR) | | | | |
| Troponin I (maximum) | 18.4 (3.6–50.0) | 2.2 (0.3–10.0) | 3.88 (0.62–21.4) | 88.1% |
| Troponin T (maximum) | 1.8 (0.60–4.8) | 0.4 (0.14–1.1) | 0.65 (0.18–2) | 90.6% |
| CK (maximum) | 721 (222–1631) | 219 (97–648) | 331 (123–1068) | 79.6% |
| | | | | |
| **Biomarkers before index MI** mean (SD) | | | | |
| Haemoglobin, g/dL, | 14.1 (1.72) | 13.6 (1.84) | 13.7 (1.83) | 67.3% |
| White blood cell count | 8.0 (2.94) | 7.9 (2.92) | 7.9 (2.92) | 69.3% |
| Neutrophil count | 5.0 (2.12) | 5.0 (2.40) | 5.0 (2.35) | 72.1% |
| Platelets | 270 (85.2) | 266 (88.0) | 267 (87.5) | 69.3% |
| Erythrocyte sedimentation rate | 19.5 (18.8) | 22.2 (21.6) | 21.8 (21.2) | 89.5% |
| Creatinine, µmol/L | 98.5 (39.1) | 102.5 (47.3) | 102 (46.0) | 59.0% |
| eGFR - CKD-EPI | 70.4 (19.9) | 66.2 (19.9) | 67.0 (20.0) | 60.7% |
| Random glucose concentration, mmol/L | 7.09 (3.53) | 7.26 (3.89) | 7.23 (3.82) | 76.7% |
| Total cholesterol | 5.39 (1.45) | 5.24 (1.28) | 5.27 (1.31) | 63.7% |
| LDL cholesterol | 3.24 (1.12) | 3.08 (1.10) | 3.11 (1.11) | 81.4% |
| | | | | |
| **STEMI** | | | | |
| Site of infarction | | | | |
| Anterior | 393 (40.2%) | | | 92.6% |
| Primary PCI | 258 (19.3%) | | | 85.3% |
| Prehospital fibrinolysis | 324 (7.0%) | | | 0% |
| Median (IQR) delay from symptom to reperfusion (min) | 150 (97 – 280) | | | 37.5% |
| | | | | |



For peer review only - http://bmjopen.bmj.com/site/about/guidelines.xhtml

| **Prescribed medication before index MI** | | | | |
|---|---|---|---|---|
| Antiplatelet | 867 (18.6%) | 6979 (35.2%) | 7846 (32.1%) | 0% |
| Oral anticoagulant | 67 (1.4%) | 624 (3.1%) | 691 (2.8%) | 0% |
| Statin | 825 (17.7%) | 5297 (26.7%) | 6122 (25.0%) | 0% |
| ACE inhibitor | 676 (14.5%) | 4363 (22.0%) | 5039 (20.6%) | 0% |
| Angiotensin receptor blocker (ARB) | 220 (4.7%) | 1208 (6.1%) | 1428 (5.8%) | 0% |
| Beta-blocker | 766 (16.4%) | 5363 (27.1%) | 6129 (25.0%) | 0% |
| Calcium channel blocker | 804 (17.3%) | 4621 (23.3%) | 5425 (22.2%) | 0% |
| Loop diuretic | 255 (5.5%) | 2188 (11.0%) | 2443 (10.0%) | 0% |
| Aldosterone antagonist | 24 (0.5%) | 198 (1.0%) | 222 (0.9%) | 0% |
| Digoxin | 53 (1.1%) | 515 (2.6%) | 568 (2.3%) | 0% |

IQR=inter-quartile range; SD=standard deviation; MI=myocardial infarction; STEMI=ST-segment-elevation myocardial infarction; PCI=Percutaneous Coronary Intervention; CABG=Coronary Artery Bypass Grafting; TIA=Transient Ischaemic Attack; COPD=Chronic Obstructive Pulmonary Disease; CK=Creatine kinase; ACE=Angiotensin-converting-enzyme. Prehospital and in-hospital fibrinolysis are not mutually exclusive.

For peer review only

**Table 2 - Hazard ratio for heart failure in patients following a first myocardial infarction using multivariable Cox regression**

| | Model 1<br>n = 24,479<br>n events = 5,775 | Model 2<br>n = 24,479<br>n events = 5,775 | Model 3<br>n = 24,479<br>n events = 5,775 | Model 4<br>n = 24,479<br>n events = 5,775 |
|---|---|---|---|---|
| | HR (95% CI) | HR (95% CI) | HR (95% CI) | HR (95% CI) |
| Age, per 10 years | 1.51 (1.48 – 1.55) | 1.49 (1.46 – 1.53) | 1.46 (1.43 – 1.50) | 1.45 (1.41 – 1.49) |
| Men | 1.06 (1.00 – 1.12) | 1.08 (1.02 – 1.14) | 1.07 (1.01 – 1.13) | 1.06 (1.00 – 1.12) |
| Index of multiple deprivation<br>Q1 (least deprived)<br>Q2<br>Q3<br>Q4<br>Q5 (most deprived) | | overall p = <0.001<br>Reference<br>1.09 (0.99 – 1.20)<br>1.20 (1.09 – 1.33)<br>1.20 (1.08 – 1.33)<br>1.30 (1.16 – 1.45) | overall p = <0.001<br>Reference<br>1.09 (0.99 – 1.20)<br>1.20 (1.08 – 1.32)<br>1.20 (1.08 – 1.33)<br>1.30 (1.16 – 1.45) | overall p = <0.001<br>Reference<br>1.08 (0.99 – 1.19)<br>1.18 (1.07 – 1.31)<br>1.17 (1.06 – 1.30)<br>1.27 (1.13 – 1.41) |
| History of hypertension | | 1.19 (1.12 – 1.26) | 1.17 (1.10 – 1.24) | 1.16 (1.09 – 1.23) |
| History of diabetes | | 1.48 (1.38 – 1.60) | 1.49 (1.38 – 1.60) | 1.45 (1.35 – 1.56) |
| History of atrial fibrillation | | | 1.65 (1.53 – 1.77) | 1.62 (1.51 – 1.75) |
| Type of MI<br>STEMI | | | 1.17 (1.10 – 1.26) | 1.19 (1.11 – 1.27) |
| History of peripheral arterial disease | | | | 1.38 (1.26 – 1.51) |
| History of COPD | | | | 1.28 (1.17 – 1.40) |
| Prescribed ACE inhibitor before MI | | | | 1.06 (0.99 – 1.13) |
| Prescribed Angiotensin receptor blocker (ARB) before MI | | | | 1.00 (0.89 – 1.11) |
| Prescribed Beta-blocker before MI | | | | 0.93 (0.88 – 1.00) |

Complete case analysis with the addition of clinically relevant variables per model. HR=hazard ratio; CI=confidence interval; Q=quintile; MI=myocardial infarction; NSTEMI=non-ST-segment-elevation myocardial infarction; STEMI=ST-segment-elevation myocardial infarction; COPD=chronic obstructive pulmonary disease; ACE=Angiotensin-converting enzyme.

For peer review only

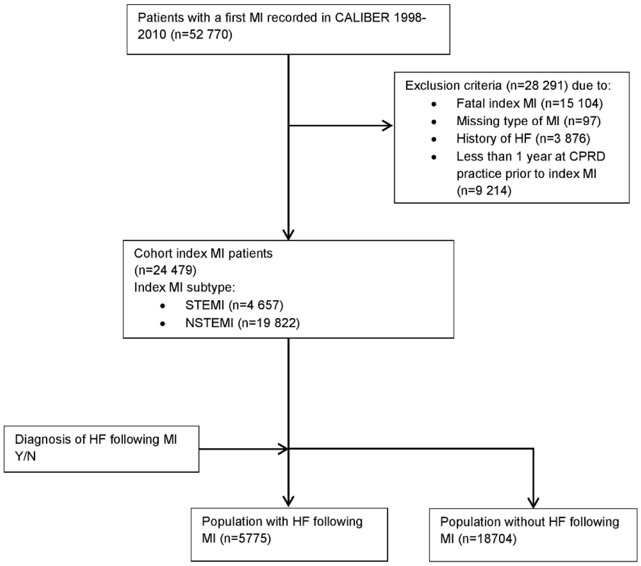


209x297mm (300 x 300 DPI)

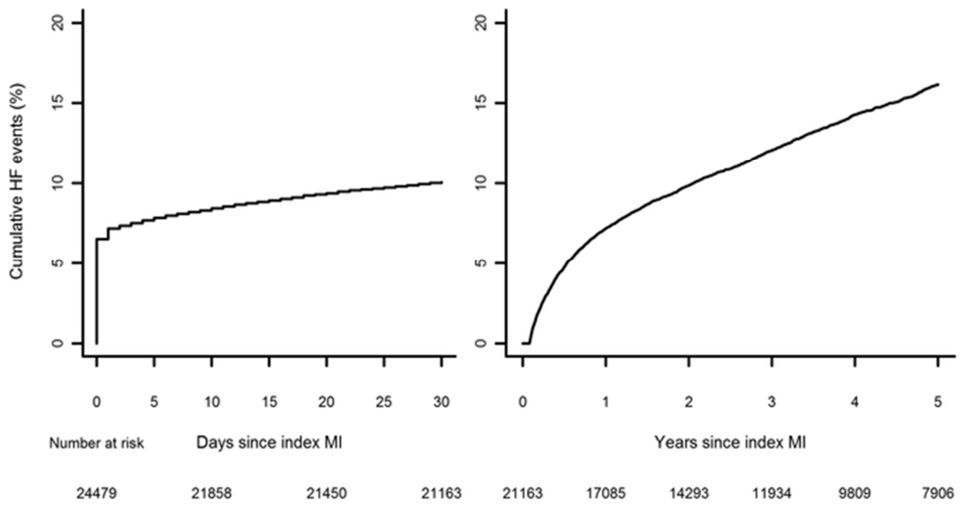


59x35mm (300 x 300 DPI)

review only

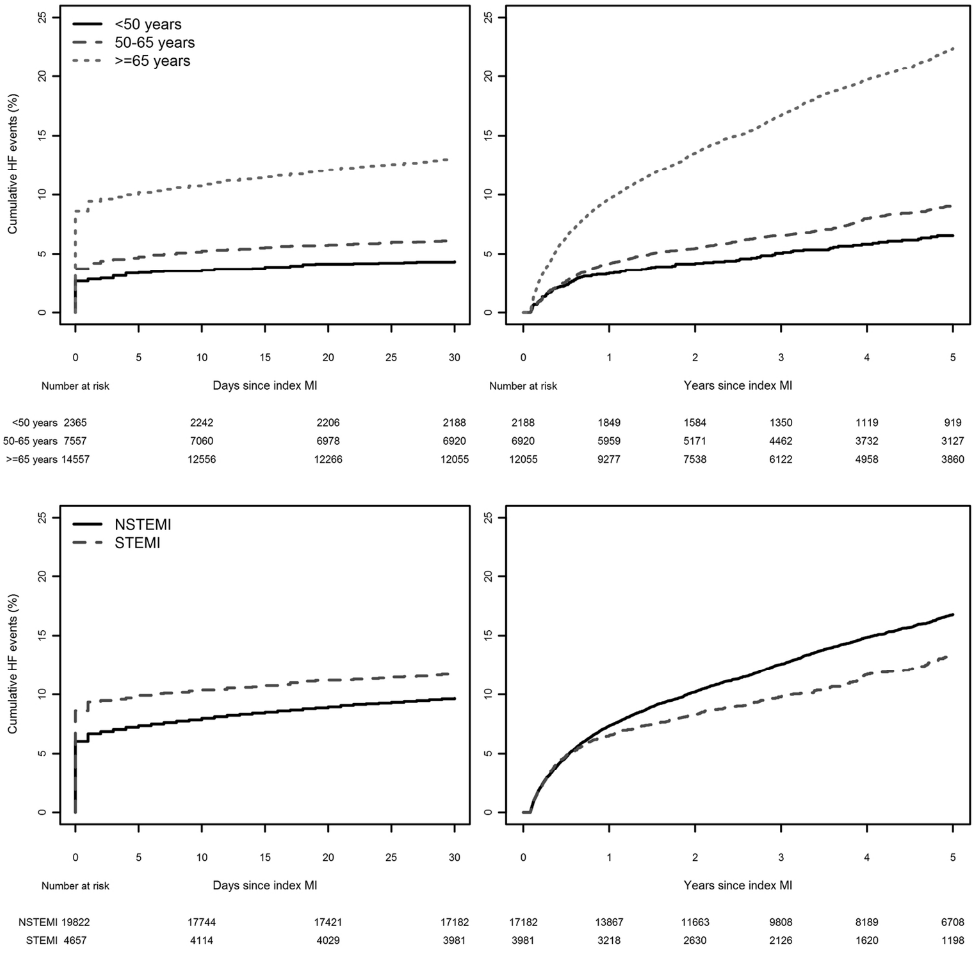


139x139mm (300 x 300 DPI)

**Supplementary Data**

## An Electronic Health Records study on Heart Failure Following Myocardial Infarction: Incidence and Prognostic Factors

Johannes M.I.H. Gho, Amand F. Schmidt, Laura Pasea, Stefan Koudstaal, Mar Pujades-Rodriguez, Spiros Denaxas, Anoop D. Shah, Riyaz S. Patel, Chris P. Gale, Arno W. Hoes, John G. Cleland, Harry Hemingway, Folkert W. Asselbergs

For peer review only

Supplementary Methods

**Ethical approval**
CALIBER was approved by the Lewisham Local Research Ethics Committee (ref: 09/H0810/16 date: 08/04/2009) and the Ethics and Confidentiality Committee (ECC) (ref: ECC 2-06(b)/2009 CALIBER dataset). CALIBER has been registered with the University College London Data Protection Officer (ref: Z6364106/2009/2/26). CALIBER EHR data are anonymised; individual informed consent was not sought from study participants.

**Multiple imputation algorithm**
Multiple imputation was implemented using the ‘mice’ package for R, to replace missing values in prognostic covariates (body mass index, smoking, systolic and diastolic blood pressure).[1] Imputation models included general practice and 3-year periods from 1998 to 2010 of index myocardial infarction (MI) and:

1. All the baseline covariates as used in the multivariable analysis (age, sex, index of multiple deprivation, hypertension, diabetes, type of MI, body mass index, smoking, systolic blood pressure, diastolic blood pressure);
2. Baseline measurements of covariates not considered in the multivariable analysis (alcohol intake, haemoglobin, white blood cell count, creatinine, alanine aminotransferase (ALAT), total cholesterol, HDL cholesterol);
3. Baseline medications prior to index MI (antiplatelet, oral anticoagulant, statin, ACE inhibitor, angiotensin receptor blocker (ARB), beta-blocker, calcium channel blocker, loop diuretic, aldosterone antagonist, digoxin);
4. Coexisting medical conditions (history of atrial fibrillation, peripheral arterial disease, depression, cancer, chronic obstructive pulmonary disease);
5. The Nelson-Aalen hazard and the event status for the outcomes analysed.

Missing data was multiply imputed and across 20 datasets a (stratified) multivariable Cox regression model was fit and results were combined using Rubin’s rules.[2]

References

1. van Buuren S. Multiple imputation of discrete and continuous data by fully conditional specification. Stat Methods Med Res 2007;16:219-42.
2. Carpenter JR, Kenward MG. *Multiple Imputation and its Application*: Wiley; 2013.

For peer review only

**Table S1 - Hazard ratio for heart failure in patients following a first myocardial infarction using the competing risks regression based on Fine and Gray's proportional subhazards model to account for the competing risk of all cause mortality**

| | Model 1 | Model 2 | Model 3 | Model 4 |
|---|---|---|---|---|
| | HR (95% CI) | HR (95% CI) | HR (95% CI) | HR (95% CI) |
| Age, per 10 years increase | 1.41 (1.38 – 1.44) | 1.39 (1.36 – 1.42) | 1.36 (1.33 – 1.39) | 1.35 (1.32 – 1.38) |
| Men | 1.04 (0.99 – 1.10) | 1.07 (1.01 – 1.13) | 1.06 (1.00 – 1.12) | 1.05 (1.00 – 1.11) |
| Index of multiple deprivation | | overall p = <0.001 | overall p = <0.001 | overall p = <0.001 |
| Q1 | | Reference | Reference | Reference |
| Q2 | | 1.11 (1.03 – 1.21) | 1.11 (1.02 – 1.20) | 1.10 (1.01 – 1.20) |
| Q3 | | 1.21 (1.12 – 1.32) | 1.20 (1.11 – 1.31) | 1.19 (1.09 – 1.29) |
| Q4 | | 1.24 (1.14 – 1.35) | 1.24 (1.14 – 1.34) | 1.22 (1.12 – 1.32) |
| Q5 | | 1.36 (1.25 – 1.47) | 1.35 (1.25 – 1.47) | 1.32 (1.21 – 1.43) |
| History of hypertension | | 1.15 (1.09 – 1.22) | 1.13 (1.07 – 1.19) | 1.14 (1.08 – 1.21) |
| History of diabetes | | 1.39 (1.29 – 1.49) | 1.38 (1.29 – 1.48) | 1.36 (1.27 – 1.47) |
| History of atrial fibrillation | | | 1.50 (1.40 – 1.61) | 1.49 (1.39 – 1.60) |
| Type of MI | | | | |
| STEMI | | | 1.10 (1.03 – 1.18) | 1.11 (1.03 – 1.18) |
| History of peripheral arterial disease | | | | 1.29 (1.19 – 1.40) |
| History of COPD | | | | 1.18 (1.09 – 1.29) |
| ACE inhibitor use before MI | | | | 1.01 (0.95 – 1.08) |
| Angiotensin receptor blocker (ARB) use before MI | | | | 0.94 (0.85 – 1.05) |
| Beta-blocker use before MI | | | | 0.94 (0.88 – 1.00) |

Complete case analysis with the addition of clinically relevant variables per model (n = 24,479, n events = 5,775). Overall p-values are shown for categorical covariates. ACE=Angiotensin-converting enzyme; CI=confidence interval; COPD=chronic obstructive pulmonary disease; MI=myocardial infarction; NSTEMI=non-ST-segment-elevation myocardial infarction; STEMI=ST-segment-elevation myocardial infarction; Q=quintile; HR=ratio of the subdistribution hazards.

For peer review only

**Table S2 - Hazard ratio for heart failure in patients following a first myocardial infarction in sensitivity analyses**

| | Model 5<br>Cox regression<br>complete case without multiple imputation<br>n = 4,898<br>n events = 1,159 | Model 5<br>Cox regression<br>with multiple imputation<br>n = 24,479<br>n events = 5,775 | Model 5<br>Competing risk analysis<br>with complete case<br>n = 4,898<br>n events = 1,159 |
|---|---|---|---|
| | HR (95% CI) | HR (95% CI) | HR (95% CI) |
| Age, per 10 years increase | 1.48 (1.38 – 1.60) | 1.47 (1.43 – 1.51) | 1.44 (1.35 – 1.54) |
| Men | 1.03 (0.89 – 1.19) | 1.07 (1.01 – 1.13) | 1.07 (0.95 – 1.22) |
| Body mass index (BMI) | p = 0.005 | p = <0.001 | p = < 0.001 |
| Underweight | 0.78 (0.48 – 1.32) | 0.76 (0.58 – 0.99) | 0.71 (0.45 – 1.12) |
| Normal | Reference | Reference | Reference |
| Overweight | 0.84 (0.71 – 0.99) | 0.84 (0.76 – 0.92) | 0.84 (0.73 – 0.97) |
| Obese | 1.12 (0.94 – 1.34) | 0.98 (0.90 – 1.08) | 1.17 (1.00 – 1.36) |
| Index of multiple deprivation | p = 0.040 | p < 0.001 | p = < 0.001 |
| Q1 | Reference | Reference | Reference |
| Q2 | 1.22 (0.94 – 1.58) | 1.08 (0.98 – 1.19) | 1.35 (1.10 – 1.66) |
| Q3 | 1.34 (1.03 – 1.75) | 1.17 (1.06 – 1.30) | 1.40 (1.14 – 1.71) |
| Q4 | 1.23 (0.93 – 1.63) | 1.16 (1.05 – 1.29) | 1.31 (1.07 – 1.61) |
| Q5 | 1.45 (1.09 – 1.92) | 1.25 (1.12 – 1.40) | 1.61 (1.33 – 1.96) |
| Smoking status | p = 0.792 | p = 0.012 | p = 0.952 |
| Non-smoker | Reference | Reference | Reference |
| Current smoker | 0.95 (0.77 – 1.14) | 1.14 (1.04 – 1.25) | 1.02 (0.86 – 1. 20) |
| Ex-smoker | 0.98 (0.83 – 1.15) | 1.03 (0.94 – 1.12) | 0.99 (0.87 – 1.14) |
| History of hypertension | 1.14 (0.96 – 1.35) | 1.16 (1.09 – 1.24) | 1.18 (1.02 – 1.37) |
| History of diabetes | 1.28 (1.09 – 1.49) | 1.46 (1.36 – 1.58) | 1.23 (1.08 – 1.39) |
| History of atrial fibrillation | 1.49 (1.25 – 1.76) | 1.63 (1.51 – 1.75) | 1.37 (1.18 – 1.59) |
| Type of MI | | | |
| STEMI | 1.15 (0.97 – 1.37) | 1.18 (1.10 – 1.27) | 1.10 (0.95 – 1.28) |
| History of peripheral arterial disease | 1.53 (1.25 – 1.87) | 1.37 (1.25 – 1.50) | 1.33 (1.13 – 1.57) |
| History of COPD | 1.21 (0.99 – 1.48) | 1.24 (1.15 – 1.38) | 1.22 (1.03 – 1.44) |
| Systolic blood pressure, per 10 mmHg increase | 0.99 (0.95 – 1.03) | 1.00 (0.98 – 1.02) | 1.01 (0.97 – 1.04) |
| Diastolic blood pressure, per 10 mmHg increase | 1.01 (0.94 – 1.09) | 0.99 (0.96 – 1.03) | 0.99 (0.93 – 1.06) |
| ACE inhibitor use before MI | 1.01 (0.87 – 1.17) | 1.06 (0.99 – 1.14) | 1.07 (0.94 – 1.21) |
| Angiotensin receptor blocker (ARB) use before MI | 0.99 (0.95 – 1.03) | 1.00 (0.90 – 1.12) | 0.93 (0.78 – 1.12) |
| Beta-blocker use before MI | 1.01 (0.94 – 1.09) | 0.94 (0.88 – 1.00) | 0.90 (0.79 – 1.02) |

Using multivariable Cox regression without and with multiple imputation and the competing risks regression based on Fine and Gray's proportional subhazards model to account for the competing risk of all cause mortality (n = 4898, n events = 1159). Additional prognostic covariates: body mass index, smoking, systolic and diastolic blood pressure. Overall p-values are shown for categorical covariates. HR=hazard ratio; CI=confidence interval; MI=myocardial infarction; NSTEMI=non-ST-segment-elevation myocardial infarction; STEMI=ST-segment-elevation myocardial infarction; COPD=chronic obstructive pulmonary disease; ACE=Angiotensin-converting enzyme; Q=quintile.

For peer review only

**Supplementary Figure 1 – Cumulative incidence curves for HF accounting for mortality as first event after index MI**

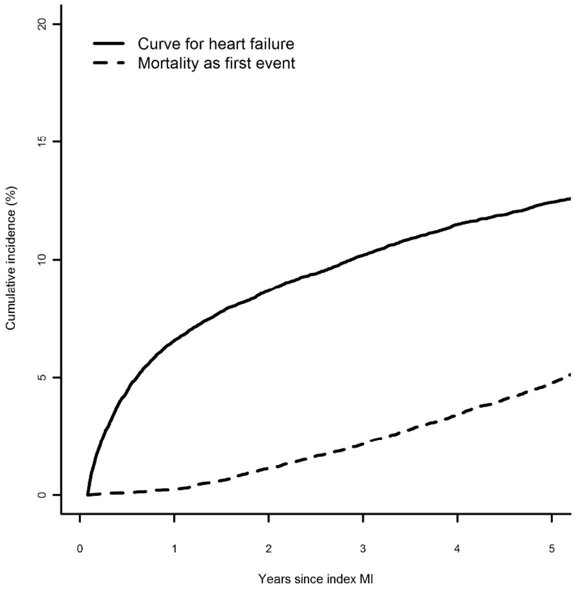


Cumulative incidence curves adjusted for competing risk of mortality as first event. 5-year follow-up in patients who survived the first 30 days and did not develop HF during the first 30 days (30 days event free).

For peer review only

STROBE Statement—checklist of items that should be included in reports of observational studies

| | Item No | Recommendation |
|---|---|---|
| **Title and abstract** | 1 ✓ | (*a*) ✓*page 2* - Indicate the study's design with a commonly used term in the title or the abstract |
| | | (*b*) ✓*page 2* - Provide in the abstract an informative and balanced summary of what was done and what was found |
| **Introduction** | | |
| Background/rationale | 2✓ | ✓*page 4* - Explain the scientific background and rationale for the investigation being reported |
| Objectives | 3✓ | ✓ *page 4* - State specific objectives, including any prespecified hypotheses |
| **Methods** | | |
| Study design | 4✓ | ✓*pages 4 through 6* - Present key elements of study design early in the paper |
| Setting | 5✓ | Describe the setting, locations, and relevant dates, including periods of recruitment, exposure, follow-up, and data collection |
| Participants | 6✓ | ✓*page 5* - (*a*) *Cohort study*—Give the eligibility criteria, and the sources and methods of selection of participants. Describe methods of follow-up<br>*Case-control study*—Give the eligibility criteria, and the sources and methods of case ascertainment and control selection. Give the rationale for the choice of cases and controls<br>*Cross-sectional study*—Give the eligibility criteria, and the sources and methods of selection of participants |
| | | (*b*) *Cohort study*—For matched studies, give matching criteria and number of exposed and unexposed<br>*Case-control study*—For matched studies, give matching criteria and the number of controls per case |
| Variables | 7✓ | ✓*page 5* - Clearly define all outcomes, exposures, predictors, potential confounders, and effect modifiers. Give diagnostic criteria, if applicable |
| Data sources/ measurement | 8✓* | ✓*page 5* - For each variable of interest, give sources of data and details of methods of assessment (measurement). Describe comparability of assessment methods if there is more than one group |
| Bias | 9✓ | ✓*page 6* - Describe any efforts to address potential sources of bias |
| Study size | 10✓ | ✓*page 7 and figure 1*- Explain how the study size was arrived at |
| Quantitative variables | 11✓ | ✓*page 6* - Explain how quantitative variables were handled in the analyses. If applicable, describe which groupings were chosen and why |
| Statistical methods | 12✓ | (*a*) *NA* Describe all statistical methods, including those used to control for confounding |
| | | (*b*) *NA* Describe any methods used to examine subgroups and interactions |
| | | (*c*) ✓*page 6* - Explain how missing data were addressed |
| | | (*d*) ✓*page 6* - *Cohort study*—If applicable, explain how loss to follow-up was addressed<br>*Case-control study*—If applicable, explain how matching of cases and controls was addressed<br>*Cross-sectional study*—If applicable, describe analytical methods taking account of sampling strategy |
| | | (*e*) ✓Describe any sensitivity analyses |

For peer review only

Continued on next page

For peer review only

| | | |
|---|---|---|
| **Results** | | |
| Participants | 13* | ✓*page 6* - (a) Report numbers of individuals at each stage of study—eg numbers potentially eligible, examined for eligibility, confirmed eligible, included in the study, completing follow-up, and analysed |
| | | ✓*page 6 and figure 1* - (b) Give reasons for non-participation at each stage |
| | | ✓*figure 1* - (c) Consider use of a flow diagram |
| Descriptive data | 14*✓ | (a) ✓*table 1* - Give characteristics of study participants (eg demographic, clinical, social) and information on exposures and potential confounders |
| | | (b) ✓*table 1* - Indicate number of participants with missing data for each variable of interest |
| | | (c) ✓*table 1*- *Cohort study*—Summarise follow-up time (eg, average and total amount) |
| Outcome data | 15* | ✓*table 1, figures 2-3 and pages 7-8 Cohort study*—Report numbers of outcome events or summary measures over time |
| | | *Case-control study*—Report numbers in each exposure category, or summary measures of exposure |
| | | *Cross-sectional study*—Report numbers of outcome events or summary measures |
| Main results | 16 | ✓*table 2, appendix tables* and pages 7-8 - (*a*) Give unadjusted estimates and, if applicable, confounder-adjusted estimates and their precision (eg, 95% confidence interval). Make clear which confounders were adjusted for and why they were included |
| | | *NA* (*b*) Report category boundaries when continuous variables were categorized |
| | | ✓ absolute rates reported in pages 7-8 - (*c*) If relevant, consider translating estimates of relative risk into absolute risk for a meaningful time period |
| Other analyses | 17 | ✓*pages 7-8* - Report other analyses done—eg analyses of subgroups and interactions, and sensitivity analyses |
| **Discussion** | | |
| Key results | 18 | ✓*page 8* Summarise key results with reference to study objectives |
| Limitations | 19 | ✓*pages 9-10* - Discuss limitations of the study, taking into account sources of potential bias or imprecision. Discuss both direction and magnitude of any potential bias |
| Interpretation | 20 | ✓*pages 8-10* Give a cautious overall interpretation of results considering objectives, limitations, multiplicity of analyses, results from similar studies, and other relevant evidence |
| Generalisability | 21 | ✓ *page 9* Discuss the generalisability (external validity) of the study results |
| **Other information** | | |
| Funding | 22 | ✓*page 11* Give the source of funding and the role of the funders for the present study and, if applicable, for the original study on which the present article is based |

*Give information separately for cases and controls in case-control studies and, if applicable, for exposed and unexposed groups in cohort and cross-sectional studies.

**Note:** An Explanation and Elaboration article discusses each checklist item and gives methodological background and published examples of transparent reporting. The STROBE checklist is best used in conjunction with this article (freely available on the Web sites of PLoS Medicine at http://www.plosmedicine.org/, Annals of Internal Medicine at http://www.annals.org/, and Epidemiology at http://www.epidem.com/). Information on the STROBE Initiative is available at www.strobe-statement.org.

For peer review only

BMJ Open

# An Electronic Health Records cohort study on Heart Failure Following Myocardial Infarction in England: Incidence and Prognostic Factors

| | |
|---|---|
| Journal: | *BMJ Open* |
| Manuscript ID | bmjopen-2017-018331.R1 |
| Article Type: | Research |
| Date Submitted by the Author: | 23-Aug-2017 |
| Complete List of Authors: | Gho, Johannes; Universitair Medisch Centrum Utrecht, Cardiology<br>Schmidt, Amand; Faculty of Science and Engineering, Groningen Research Institute of Pharmacy<br>Pasea, Laura; Farr Institute<br>Koudstaal, Stefan; Farr Institute<br>Pujades Rodriguez, Mar; Farr Institute<br>Denaxas, S; University College London, Clinical Epidemiology Group<br>Shah, Anoop; UCL, Farr Institute of Health Informatics research<br>Patel, Riyaz; Farr Institute of Health Informatics Research, University College London, Epidemiology and Public Health; Bart's Heart Centre, Cardiology<br>Gale, Chris; University of Leeds, Division of Epidemiology and Biostatistics<br>Hoes, Arno; Julius Center for Health Sciences and Primary Care, University Medical Center, Utrecht<br>Cleland, J; National Heart and Lung Institute,<br>Hemingway, Harry; UCL, Epidemiology and Public Health<br>Asselbergs, Folkert; University Medical Centre Utrecht, Department of Cardiology; ICIN-Netherlands Heart Institute, Durrer Center for Cardiogenetic Research |
| <b>Primary Subject Heading</b>: | General practice / Family practice |
| Secondary Subject Heading: | Cardiovascular medicine, Epidemiology, General practice / Family practice, Health informatics, Public health |
| Keywords: | Heart failure < CARDIOLOGY, Myocardial infarction < CARDIOLOGY, electronic health records |
| | |

SCHOLARONE™
Manuscripts

For peer review only - http://bmjopen.bmj.com/site/about/guidelines.xhtml

# An Electronic Health Records cohort study on Heart Failure Following Myocardial Infarction in England: Incidence and Prognostic Factors

Johannes M.I.H. Gho, MD, PhD,[1,2,*] Amand F. Schmidt, PhD,[2,3,4*] Laura Pasea, MSc,[1] Stefan Koudstaal, MD, MSc, PhD,[1,2] Mar Pujades-Rodriguez, PhD,[1,5] Spiros Denaxas, PhD,[1] Anoop D. Shah, MRCP,[1] Riyaz S. Patel, MD, PhD,[1,3] Chris P. Gale, PhD, FRCP,[6] Arno W. Hoes, MD, PhD,[7] John G. Cleland, MD, FRCP,[8] Harry Hemingway, FFPH, FRCP,[1] Folkert W. Asselbergs, MD, PhD,[1,2,3,9]

[1]Farr Institute of Health Informatics Research, UCL Institute of Health Informatics, University College London, London, UK
[2]Department of Cardiology, Division Heart and Lungs, University Medical Center Utrecht, Utrecht, the Netherlands
[3]Institute of Cardiovascular Science, Faculty of Population Health Sciences, University College London, London, UK
[4]Groningen Research Institute of Pharmacy, University of Groningen, Groningen, the Netherlands
[5]Medical Research Council Bioinformatics Centre, Leeds Institute of Biomedical and Clinical Sciences, University of Leeds, Leeds, UK
[6]Medical Research Council Bioinformatics Centre, Leeds Institute for Cardiovascular and Metabolic Medicine, University of Leeds, Leeds, UK
[7]Julius Centre for Health Sciences and Primary Care, University Medical Center Utrecht, Utrecht, the Netherlands
[8]National Heart & Lung Institute, Royal Brompton & Harefield Hospitals, Imperial College London, London, UK
[9]Durrer Center for Cardiogenetic Research, ICIN-Netherlands Heart Institute, Utrecht, the Netherlands
* Shared first author

**Corresponding author:**
A Floriaan Schmidt
Institute of Cardiovascular Science, University College London
222 Euston Road, London NW1 2DA, United Kingdom
E-mail: amand.schmidt@ucl.ac.uk; a.f.schmidt@rug.nl
Tel: 0044 (0)20 3549 5625

**Word count text**: 3427
**Word count abstract**: 265
**Number of references**: 32
**Number of tables**: 2
**Number of figures**: 3
**(Web)appendix**: 1

For peer review only

**Abstract**

**Objectives** To investigate the incidence and prognostic factors of heart failure (HF) following a myocardial infarction (MI) in a contemporary cohort of MI patients using routinely collected primary and hospital care data.

**Methods** Data were used from the CALIBER programme, linking electronic health records in England from primary care, hospital admissions, an MI registry and mortality data. Subjects were eligible if they were 18 years or older, did not have a history of HF, and survived a first MI. Factors associated with time to HF were examined using Cox proportional hazard models.

**Results** Of the 24,479 MI patients 5,775 (23.6%) developed HF during a median follow-up of 3.7 years (incidence rate per 1000 person-years 63.8 (95% CI: 62.2 – 65.5). Baseline characteristics significantly associated with HF were: atrial fibrillation [HR 1.62, 95% CI 1.51 – 1.75], age [per 10 years increase: 1.45, 1.41 – 1.49], diabetes [1.45, 1.35 – 1.56], peripheral arterial disease [1.38, 1.26 – 1.51], COPD [1.28, 1.17 – 1.40], greater socioeconomic deprivation [$5^{th}$ vs. $1^{st}$ quintile 1.27, 1.13 – 1.41], ST-segment elevation MI at presentation [1.19, 1.11 – 1.27], hypertension [1.16, 1.09 – 1.23], currently smoking [1.14, 1.04 – 1.25] and BMI [underweight 0.76, 0.58 – 0.99, overweight 0.84, 0.76 – 0.92 vs. normal weight]. Results were robust to various sensitivity analyses such as competing risk analysis and multiple imputation.

**Conclusion** In England, one in four survivors of a first MI developed HF during a median follow-up of four years. This contemporary study demonstrates MI patients are at lower, but still considerable, risk of HF. Baseline patient characteristics associated with time till HF were identified, which may fruitfully be used in future trial design.

**Keywords:** heart failure; myocardial infarction; incidence; risk factors; electronic health records

For peer review only



For peer review only - http://bmjopen.bmj.com/site/about/guidelines.xhtml

**Strengths and limitations of this study**

- ❖ The current study provides a contemporary description of the burden of heart failure in a representative sample of first time myocardial infarction patient included in the CALIBER, an UK based electronic health care database. Contrary to previous electronic health care studies, who exclusively focussed on hospital records, the current study includes both primary (GP) and hospital records increasing generalizability.
- ❖ Robust methods were used to account for potential calendar and centre effects, as well as competing risks.
- ❖ While most patient characteristics (e.g., disease history and drug prescriptions) were completely recorded, information regarding baseline BMI, smoking and blood pressure was missing for up to 70% of patients. Analyses based on multiple imputed data were similar to those from complete case analyses. Despite these reassuring findings, results for variables with a high number of missing observations should be carefully interpreted.
- ❖ Further potential limitations include the reliance on ICD and Read codes for disease classification.
- ❖ A small proportion of subjects may have had an earlier myocardial infarction or heart failure event, which was not recorded in CALIBER due to left truncation of follow-up time.

**Abbreviations list:** heart failure (HF); myocardial infarction (MI); ST-segment elevation MI (STEMI); hazard ratio (HR); confidence interval (CI); chronic obstructive pulmonary disease (COPD); body mass index (BMI).

For peer review only


For peer review only - http://bmjopen.bmj.com/site/about/guidelines.xhtml

## Introduction

Research describing the incidence of heart failure (HF) following myocardial infarction (MI) is limited, mainly originating from the thrombolytic era, often using small sample sizes with contradictory findings, making it difficult to provide evidence based medicine. For example, a previous UK study among almost 900 patients hospitalised with MI in 1998, found that one fifth developed HF during their hospital stay and a further third following hospital discharge[1]. More recently, a Swedish study found a 5-year cumulative risk of HF after MI of 21.8% in the calendar period 2004-2013[2]. Further, a Danish nationwide cohort study reported an incidence of HF at 90-days following MI of 19.6% in 2009-10[3]. Differences between these studies could be related to a number of factors, for example change in treatment or national policies, all of which potentially limits generalizability of results. We used a large contemporary and representative sample of patients with MI based on electronic health records to 1) describe the current incidence of HF after MI in England, using both primary and hospital care data, and 2) explore prognostic patient characteristics of post MI HF[4,5].

## Methods

Study design

Data were used from the CALIBER programme dataset, which included linked data from (i) primary care electronic health records, with diagnoses coded using the Read terminology (Clinical Practice Research Datalink, CPRD) from 226 consenting general practices[6], (ii) hospital care and audit records (Hospital Episode Statistics, HES) and audit records (Myocardial Ischaemia National Audit Project, MINAP), the Office of Population Censuses and Surveys Classification of Interventions and Procedures (OPCS-4), and (iii) the mortality register (from UK Office for National Statistics, ONS) [7].

Setting and participants

Patients were eligible if they were aged ≥18 years, registered in CPRD practices in England consenting to data linkage, with at least one year of up-to-standard pre-study follow-up (meeting CPRD practice quality



For peer review only - http://bmjopen.bmj.com/site/about/guidelines.xhtml

standards), and experiencing a first MI between 1st January 1998 and 25th March 2010. The first record of MI across the linked data sources was considered as the index event and subsequent MI records within 30 days from the same or other sources were considered as representing the same event [4]. Patients with a fatal index MI or a history of HF before their index MI were excluded. Patients were censored if they ceased to be registered at a participating general practice, at the date of death or the administrative censoring of the dataset (25th March 2010).

Variables and data sources

Study variables were derived from Read, ICD-9 or ICD-10, MINAP, drug prodcodes or procedure codes with the electronic health record phenotyping algorithms published at https://caliberresearch.org/portal[7]. For continuous variables, the most recent measurement recorded in CPRD in the year before study entry was used as the baseline value. Data before study entry were used to determine the prognostic potential of age (years, using the date of birth), sex, ethnicity, social deprivation (Index of Multiple Deprivation, IMD score as recorded in ONS in quintiles), smoking, alcohol use, history of cardiovascular disease, previous coronary revascularization, history of diabetes, history of thyroid disease, history of chronic obstructive pulmonary disease (COPD), history of depression and history of non-metastatic cancer. (see the CALIBER portal https://caliberresearch.org/portal for details). Diabetes and hypertension diagnosis were based on Read codes from primary care or ICD10 codes from Hospital Episode Statistics, both of which were classified using primary care consultation records and hospitals diagnosis records. Socioeconomic status was based on the Index of Multiple Deprivation which includes seven domains of deprivation (income deprivation, employment deprivation, health deprivation and disability, education, skills and training deprivation, barriers to housing and services, living environment deprivation and crime) [8]. The index MI event was characterized using ST-segment-elevation myocardial infarction (STEMI), or non-STEMI (NSTEMI), site of infarction, mode and timing of reperfusion and peak cardiac biomarkers (troponin I, troponin T and CK). MI was defined by linking all of the CALIBER data sources (e.g., Hospital Episode Statistics and CPRD), with the type of MI recorded in MINAP and CPRD, see for more detail https://www.caliberresearch.org and the appendix. Characteristics regarding the index MI (e.g., type of revascularisation and delay from symptom onset to delivery of reperfusion therapy) were derived from



For peer review only - http://bmjopen.bmj.com/site/about/guidelines.xhtml

MINAP. This nationwide registry is part of an annual audit which records over 20 key MI variables [9]. Herrett and colleagues [4] validated the current approach and showed the necessity of linking multiple sources to ascertain MI. The primary study outcome was the first HF event following MI, which similar to MI, was recorded in multiple CALIBER data sources; echocardiographic findings and NYHA class were unavailable. All phenotypes were created using a robust methodology described elsewhere [10].

Statistical methods

Incidence rates (cases per 1000 person-years) and Kaplan-Meier cumulative incidence rates of HF were estimated, with Kaplan-Meier curves stratified by age (<50, 50-65, ≥65 years) and type of MI. Association between baseline variables with the onset of HF following MI were explored using Cox proportional hazard models. The proportional hazards assumption was checked using Schoenfeld residuals, and by nonparametric correlation coefficients between survival time and the residuals. All models were stratified on general practice and calendar year periods of enrolment (1998-2001, 2001-2004, 2004-2007 and 2007-2010). Stratified models were used instead of frailty models because the former does not make any distributional assumption. Models were sequentially adjusted for: 1) age and sex, 2) cardiovascular risk factors, 3) type of MI, and 4) co-morbidities and prescribed medication. Associations are presented as hazard ratios (HRs) with 95% confidence intervals (CI). Analyses were performed using R (version 3.3.2)[11].

Sensitivity analyses

Due to the availability of electronic health care records from multiple sources, clinical diagnoses and prescriptions were completely recorded; electronic registration is required for a patient to fill a prescription. Biomarker and lifestyle measurements such as smoking, BMI, and blood pressure were however, incompletely recorded (see Table 1). It is probable that these data were preferentially recorded in subjects perceived to be at a higher risk for early progression of CVD. Pairwise analyses regressing a complete case indicator on observed variables indeed showed considerable dependency between recorded data and missingness (results not shown), violating the “missing completely at random” assumption. This dependency was utilized by multiply imputing missing data using the mice package,



For peer review only - http://bmjopen.bmj.com/site/about/guidelines.xhtml

which was implemented using 20 imputed datasets and pooled based on Rubin's rules (Supplementary)[12].

To account for the fact that patients may have died before the onset of HF (e.g., competing risk by mortality), analyses were repeated using Fine and Gray models[13,14]. As a hypothetical example of competing risk, let us assume that out of 100 MI subjects, one develops HF and 99 die. In a Cox's regression analysis all 99 dead subjects would be censored (implicitly, and incorrectly, assuming they may develop HF later in time), and the hazard of HF would equal 1/1. Instead a Fine and Gray model recognizes the 99 died subjects could never develop HF and hence calculates the hazard as 1/100.

In addition to these sensitivity analyses, we performed a cancer-stratified analysis. Due to discrepancies in sample size between the two subgroups we decided against formal interaction testing, which may suffer from inflated false positive rates and lower power in such settings[15].

Ethical approval

CALIBER has received ethics approval (Supplementary). This study is in compliance with the Declaration of Helsinki and was approved by the ISAC (Independent Scientific Advisory Committee) for MHRA Database Research (protocol no. 14_198R) and the MINAP Academic Group and is registered with ClinicalTrials.gov (NCT02384213).

## Results

Subjects and baseline characteristics

In total there were 52,770 patients with an index MI during the study period (Figure 1). Excluding patients with a fatal index MI (n = 15,104), a prior history of HF (n = 3,876), missing information on the type of MI (n = 97) and subjects with less than 1 year of follow-up prior to the indexing MI event (n = 9,214), resulted in an analytical cohort of 24,479 patients. Patients were recruited from 226 GP practices, with the median practice enrolling 101 patients (quartile (Q1) 65 and Q3 150). Of the included subjects 4,657 were STEMI


For peer review only - http://bmjopen.bmj.com/site/about/guidelines.xhtml

and 19,822 NSTEMI patients (0% missing), 15,969 (65.2%) were men (0% missing), 4,538 (42.1%) currently smoked (56% missing), 12,258 (50.1%) had hypertension (0% missing), and 3,014 (12.3%) had a history of diabetes at baseline (0% missing). In total, 6,129 (25.0%) were prescribed beta-blockers (0% missing) and 5,039 (20.6%) angiotensin converting enzyme (ACE) inhibitors (0% missing) prior to their indexing MI (Table 1). The number of patients identified across the previously defined calendar periods was reasonably stable, with a minimum of 5219 subjects in the period 1998-2001 compared to a maximum of 6838 subjects identified in the period 2004-2007.

Incidence

Patients contributed 90,482 person years of follow-up, during a median follow-up time of 3.7 years (IQR: 1.5; 6.7), 5,775 patients (23.6%) developed HF. The crude incidence rate of HF following a first MI was 63.8 (95% CI: 62.2 – 65.5) per 1000 person-years. Within the first 30 days post MI follow-up 2,438 (10.0%) patients developed HF (Figure 2). From day 30 onwards 3,337 (15.8%) of MI patients (event free during the first 30 days) developed HF, with 6.8% experiencing an HF event within the first year (Figure 2). The incidence of HF during the first 30-days of follow-up was 4.3% (102) in patients younger than 50 years, 6.0% (459) in 50-65 year olds and 12.9% (1877) in those 65 years and older, with HF incidence increasing proportionally as time progressed towards 10 years (Figure 3 top row). The 30-day incidence of HF was 9.5% (1892 events) for NSTEMI subjects and 11.7% (546 events) for STEMI subjects. Excluding patients who experienced a HF within the first 30 days showed that STEMI patients had a lower incidence of HF than NSTEMI subjects (Figure 3 lower panels). At 57 days the crude cumulative risk of HF in the NSTEMI subjects surpassed that of the STEMI subjects for the first time (0.0151 versus 0.0147), with the curves further diverging at 73 days of follow-up (since indexing MI).

During the first 30 days of follow-up only 1 patient died, limiting the potential for competing risk by all-cause mortality. In patients who did not have HF after 30 days accounting for competing risk by all-cause mortality attenuated the cumulative risk to be about 15% after 10 years of follow-up (Appendix Figure). Furthermore, the cumulative risks of all-cause mortality and HF converged as follow-up time progressed towards 10 years.

For peer review only



For peer review only - http://bmjopen.bmj.com/site/about/guidelines.xhtml

Prognostic factors after multivariable adjustments

Table 2 describes the results of the previously described nested Cox's models, showing little change in estimates between the increasingly complex models. Focusing on the final model, the hazard of HF increased with atrial fibrillation [HR 1.62, 95%CI 1.51 – 1.75], age [per 10 years increase: 1.45, 1.41 – 1.49], diabetes [1.45, 1.35 – 1.56], peripheral arterial disease [1.38, 1.26 – 1.51], COPD [1.28, 1.17 – 1.40], hypertension [1.16, 1.09 – 1.23], higher socioeconomic deprivation (p-value for the 5 groups < 0.001), and STEMI at presentation [1.19, 1.11 – 1.27)]. Accounting for competing risk by all-cause mortality had little impact on the presented results (Appendix Table S1). After multiply imputing the data models were extended to include smoking, BMI, and systolic and diastolic blood pressure variables which showed BMI, male gender and smoking to be conditionally independent prognostic factors (Supplementary Table S2). The extended model 5 was implemented using imputed data, and non-imputed (complete case) data, resulting in similar HR estimates (magnitude and direction). Stratifying the sample on cancer diagnosis status showed broadly similar results (See appendix table S3, focusing on the confidence intervals) between subject with and without a history of cancer.

**Discussion**

In this large population-based study using linked electronic health records, 23.6% of patients who survived a first MI developed HF during a median follow-up of 3.7 years, resulting in an incidence rate of 64 cases per 1000 person-years. Incident HF was associated with increasing age, higher socioeconomic deprivation, a history of diabetes, atrial fibrillation, peripheral arterial disease, COPD, STEMI at presentation, BMI and smoking.

A previous Canadian study, using data from the period 1994-2000[16], found that 71% of elderly patients without HF at index admission developed HF within 5 years' time after an MI, whereas the mortality due to MI decreased in the same period. The Framingham Heart Study[17], using data from 1990 to 1999, found a 5-years post-MI HF incidence of 31.9% after MI; lower than found in the Canadian study. Importantly,



For peer review only - http://bmjopen.bmj.com/site/about/guidelines.xhtml

both studies showed a higher incidence of post-MI HF than our more contemporary English cohort. This lower HF incidence is likely due to continued improvements of MI treatment strategies, which are reflected in a decreased HF incidence over calendar time. For example, a 20,812 sample of MI patients hospitalized in Western Australia showed that the overall 1-year incidence of HF after MI decreased from 28.1% 1996 in to 16.5% in 2007[18]. This decline is confirmed further by a national Swedish hospital discharge and death registry study reporting a one third decline in incidence between 1993-2004[19].The same Swedish group recently reported that this trend persists for the period 2004-2013[2] and showed improved pharmacological treatment and early revascularisation in this period. During a median follow-up of 4 years, 19% of the patients were re-hospitalized because of HF. An explanation for the higher percentage in our study is probably the inclusion of HF diagnosis made in the GP setting next to hospital records decreasing misclassification of HF events. Future efforts are needed to harmonize these national data sources to compare daily care and HF epidemiology across different countries.

Prognostic factors

Similar to the current study, Torabi et al[20] reported that HF after MI increased steeply with age. Socioeconomic deprivation has been shown to be an independent predictor of HF development and associated with an increased incidence of HF in MI-free subjects [21,22]. Socioeconomic deprivation has also been associated with more frequent hospital admission and higher mortality in HF patients[23–25]. The current study extends these observations to HF incidence after an MI. Furthermore, we showed that a history of diabetes, atrial fibrillation, peripheral arterial disease, COPD, STEMI at presentation, BMI and smoking are all independently prognostic of HF after MI. Interestingly, despite the size of the collected sample, gender only became significantly associated with HF after accounting for differences in BMI and smoking. Furthermore, the impact of male gender (HR of 1.07 95%CI 1.01 – 1.13) was modest indicating relative gender equality. It is of note that blood pressure was not significantly associated with HF. However, blood pressure measurements were frequently missing, which (even after multiple imputation) may be the cause of the observed lack of association hence this deserves independent exploration. In this light it is important to note that the diagnosis of hypertension was associated with HF. Potentially this discrepancies between the association of blood pressure measurement and hypertension diagnosis can


For peer review only - http://bmjopen.bmj.com/site/about/guidelines.xhtml

be explained by noting that a recorded diagnosis is indicative of long-term hypertensions, which may be prognostically different than a single blood pressure measurement. Further, given that both variables were included in the same model, the observed difference in association may suggest that conditional on hypertension, blood pressure itself is only modestly associated to HF, if at all.

Strengths and limitations

The linkage of multiple electronic health record sources from primary and hospital care allowed for the collection of a representative sample[26], which enabled us to explore the prognostic value of routinely collected data in primary care records and to detect non-hospitalised HF cases. The population of CPRD practices has been shown broadly representative of the UK population[6,27]. In total 226 GP practices consented to data linkage with Hospital Episode Statistics, MINAP, and the Office for National Statistics (containing 3.9% of the population of England in 2006). A potential limitation is that the ascertainment of cardiovascular outcomes was not based on clinical criteria (e.g., validated questionnaires and properly conducted physical examinations), practices of medical coding will have changed over time and there could be subgroups of patients with left ventricular dysfunction without clinical symptoms. Calendar dependent changes over time were accounted for by using time-period stratified Cox models. We further wish to highlight the infrequent use of PCI in STEMI patients, which was shown to be representative of the slower uptake in England[28]. Similarly, 32% of the patients used antiplatelet drugs at baseline which may be a reflection of non-MI CVD burden. The Kaplan-Meier plots for subtype of MI indicate a possible violation of the proportional hazard assumption for this variable. However, these plots represent the pairwise associations between MI subtype and time to HF, and as such assume that MI subtype is independent of other prognostic factors, which is known to be false. The importance of conditioning on covariates is underlined by noting the Kaplan-Meier plots indicating a protective effect of STEMI versus NSTEMI, which was significantly reversed after correcting for covariates (models 3-4). Similarly, instead of using Kaplan-Meier plots to assess the proportional hazard assumption of the *crude* associations, we used Schoenfeld residuals to explore this assumption for the *conditional* associations (multivariable model 4); which did not show any violations. Based on model 4 the absolute correlation between the Schoenfeld residuals and time was < 0.10 (e.g., for MI subtype this was 0.06) indicating an absence of


For peer review only - http://bmjopen.bmj.com/site/about/guidelines.xhtml

relevant interaction by time (e.g., non-proportionality of hazard). Residual confounding due to medication use (or other missing/mis-specified variables) might be another potential source of bias, however our intention was not to perform a causal analysis between drug prescriptions and time to HF. As such it is interesting to note that despite the large sample size we did not observe a significant association of ACE, ARB or beta-blocker prescribed prior to the indexing MI event and time to HF. We adhered to CPRD recommendations to obtain up-to-standard baseline data by excluding patients with less than 1 year at CPRD practice prior to index MI. Previously Lewis and colleagues showed that 3 months after registration with a new practice most patient characteristics were updated correctly, which approximated 100% after 1 year of follow-up[29]. Assuming that duration of the CPRD follow-up is independent of the relations assessed here, excluding such patients should not hamper generalizability of results. Using the first MI recorded in the database without a prior history of HF, might have introduced bias due to left truncation (e.g., some subjects may already have experienced an MI before enrolment)[30]. However CALIBER holds longitudinal records from primary and hospital care, making it unlikely a large part of the patients were misclassified as MI free and the one year of follow-up prior to entry further decreases the likelihood of misclassifying patients[29]. While the current data is adequate to identify subjects with a first MI, the sub-classification of MI patients into STEMI and NSTEMI, despite recent improvements, is known to be error prone [31]. As such, results for MI type need replication using higher quality data, in perhaps purposely designed studies. Due to our interest in HF occurrence after a first MI selection bias (e.g., index event bias) was introduced[32]. This index event bias does not impact the descriptive or prognostic value of the association presented, and is mostly relevant if one wants to develop an intervention based on the associations presented; which was not the aim of this study. Additionally, we note that we reduced the influence of selection bias by accounting for dependencies between predictors.

A final limitation is that there is a possible delay between primary and hospital care records. Previous research has shown that MI events tend to be recorded in primary care after the HES or MINAP record [4]. The lower 30-day HF incidence in unclassified MI patients primarily derived from primary care could be partly explained because of a delay in coding. Therefore, we showed cumulative incidence rates in patients (alive and HF event free within the first 30 days) from 30 days after index MI to account for a

For peer review only

delay in recording of MI in primary care. We were unable to differentiate between HF with preserved ejection fraction (HFpEF) and HF with reduced ejection fraction (HFrEF) as we had no access to detailed (echocardiographic) parameters to assess diastolic dysfunction. It is likely, however that the majority of our HF patients had developed systolic dysfunction after MI.

## Conclusion

In this large cohort study using linked electronic health records in England from primary and hospital care, about one in four people developed HF within a median of four years after surviving a first MI. Increasing age, higher socioeconomic deprivation, a history of hypertension, diabetes, atrial fibrillation, peripheral arterial disease, COPD, smoking and STEMI at presentation were associated with new onset HF following MI, while the hazard of post MI HF was lower for overweight and underweight patients.

## Authors' contributions

JMIHG, AFS, LP, SK, MPR, SD, FWA, HM, JGC, AWH, CPG, RSP, ADS contributed to the idea and design of the study. JMIHG extracted and prepared the data for analysis. AFS and JMIHG performed the analysis. JMIHG drafted the manuscript, with revisions by AFS, LP, SK, MPR, SD, FWA, HM, JGC, AWH, CPG, RSP, ADS. JMIHG guaranties the quality and accuracy of the results presented.

## Competing interests

We have read and understood BMJ policy on declaration of interests and declare that we have no competing interests

## Data Sharing Statement

Data access can be requested via the CALIBER portal: https://www.ucl.ac.uk/health-informatics/caliber.

## Funding

The Farr Institute is funded from the Medical Research Council, Arthritis Research UK, British Heart Foundation, Cancer Research UK, Chief Scientist Office, Economic and Social Research Council, Engineering and Physical Sciences Research Council, NIHR, National Institute for Social Care and Health Research, and Wellcome Trust. Part of this work is funded through the Innovative Medicines Initiative 2 Joint Undertaking under grant agreement No 116074, BigData@Heart. AFS is funded by UCLH NIHR Biomedical Research Centre and is a UCL Springboard Population Health Sciences Fellow. HH is an NIHR Senior Investigator, and receives support from the NIHR University College Hospitals / University College London Biomedical Research Centre. FWA is supported by a Dekker scholarship-

For peer review only

Junior Staff Member 2014T001 – Netherlands Heart Foundation and UCL Hospitals NIHR Biomedical Research Centre.

For peer review only

**References**


1 Torabi A, Cleland JGF, Khan NK, *et al.* The timing of development and subsequent clinical course of heart failure after a myocardial infarction. *Eur Heart J* 2008;**29**:859–70. doi:10.1093/eurheartj/ehn096

2 Desta L, Jernberg T, Spaak J, *et al.* Risk and predictors of readmission for heart failure following a myocardial infarction between 2004 and 2013: A Swedish nationwide observational study. *Int J Cardiol* Published Online First: May 2017. doi:10.1016/j.ijcard.2017.05.086

3 Gjesing A, Gislason GH, Kober L, *et al.* Nationwide trends in development of heart failure and mortality after first-time myocardial infarction 1997-2010: A Danish cohort study. *Eur J Intern Med* 2014;**25**:731–8. doi:10.1016/j.ejim.2014.08.009

4 Herrett E, Shah AD, Boggon R, *et al.* Completeness and diagnostic validity of recording acute myocardial infarction events in primary care, hospital care, disease registry, and national mortality records: cohort study. *BMJ* 2013;**346**:f2350.

5 Hripcsak G, Albers DJ. Next-generation phenotyping of electronic health records. *J Am Med Inform Assoc* 2013;**20**:117–21. doi:10.1136/amiajnl-2012-001145

6 Herrett E, Gallagher AM, Bhaskaran K, *et al.* Data Resource Profile: Clinical Practice Research Datalink (CPRD). *Int J Epidemiol* 2015;**44**:827–36. doi:10.1093/ije/dyv098

7 Denaxas SC, George J, Herrett E, *et al.* Data resource profile: Cardiovascular disease research using linked bespoke studies and electronic health records (CALIBER). *Int J Epidemiol* 2012;**41**:1625–38. doi:10.1093/ije/dys188

8 DCLG. The English Indices of Deprivation 2010. *Neighb Stat Release* 2011;:1–20. doi:http://dx.doi.org/http://www.communities.gov.uk/publications/corporate/statistics/indices2010technicalreport

9 Herrett E, Smeeth L, Walker L, *et al.* The Myocardial Ischaemia National Audit Project (MINAP). *Heart* 2010;**96**:1264–7. doi:10.1136/hrt.2009.192328

10 Morley KI, Wallace J, Denaxas SC, *et al.* Defining Disease Phenotypes Using National Linked Electronic Health Records: A Case Study of Atrial Fibrillation. *PLoS One* 2014;**9**:e110900. doi:10.1371/journal.pone.0110900


For peer review only



For peer review only - http://bmjopen.bmj.com/site/about/guidelines.xhtml

11 R Core Team. R: A language and environment for statistical computing. 2017.

12 Marshall A, Altman DG, Holder RL, *et al.* Combining estimates of interest in prognostic modelling studies after multiple imputation: current practice and guidelines. BMC Med. Res. Methodol. 2009;**9**:57-.

13 Satagopan JM, Ben-Porat L, Berwick M, *et al.* A note on competing risks in survival data analysis. *Br J Cancer* 2004;**91**:1229–35. doi:10.1038/sj.bjc.6602102

14 Gray B. cmprsk: Subdistribution Analysis of Competing Risks. 2014.http://cran.r-project.org/package=cmprsk

15 Schmidt AF, Groenwold RHH, Knol MJ, *et al.* Exploring interaction effects in small samples increases rates of false-positive and false-negative findings: Results from a systematic review and simulation study. *J Clin Epidemiol* Published Online First: 2014. doi:10.1016/j.jclinepi.2014.02.008

16 Ezekowitz JA, Kaul P, Bakal JA, *et al.* Declining in-hospital mortality and increasing heart failure incidence in elderly patients with first myocardial infarction. *J Am Coll Cardiol* 2009;**53**:13–20. doi:10.1016/j.jacc.2008.08.067

17 Velagaleti RS, Pencina MJ, Murabito JM, *et al.* Long-term trends in the incidence of heart failure after myocardial infarction. *Circulation* 2008;**118**:2057–62. doi:10.1161/CIRCULATIONAHA.108.784215

18 Hung J, Teng T-HK, Finn J, *et al.* Trends from 1996 to 2007 in incidence and mortality outcomes of heart failure after acute myocardial infarction: a population-based study of 20,812 patients with first acute myocardial infarction in Western Australia. *J Am Heart Assoc* 2013;**2**:e000172. doi:10.1161/JAHA.113.000172

19 Shafazand M, Rosengren A, Lappas G, *et al.* Decreasing trends in the incidence of heart failure after acute myocardial infarction from 1993-2004: a study of 175,216 patients with a first acute myocardial infarction in Sweden. *Eur J Heart Fail* 2011;**13**:135–41. doi:10.1093/eurjhf/hfq205

20 Torabi A, Cleland JG, Rigby AS, *et al.* Development and course of heart failure after a myocardial infarction in younger and older people. *J Geriatr Cardiol* 2014;**11**:1–12. doi:10.3969/j.issn.1671-5411.2014.01.002

21 McAlister FA, Murphy NF, Simpson CR, *et al.* Influence of socioeconomic deprivation on the

For peer review only



For peer review only - http://bmjopen.bmj.com/site/about/guidelines.xhtml

primary care burden and treatment of patients with a diagnosis of heart failure in general practice in Scotland: population based study. *BMJ* 2004;**328**:1110. doi:10.1136/bmj.38043.414074.EE

22 Hawkins NM, Jhund PS, McMurray JJ V, *et al.* Heart failure and socioeconomic status: Accumulating evidence of inequality. *Eur J Heart Fail* 2012;**14**:138–46. doi:10.1093/eurjhf/hfr168

23 Philbin EF, Dec GW, Jenkins PL, *et al.* Socioeconomic status as an independent risk factor for hospital readmission for heart failure. *Am J Cardiol* 2001;**87**:1367–71.

24 Struthers AD, Anderson G, Donnan PT, *et al.* Social deprivation increases cardiac hospitalisations in chronic heart failure independent of disease severity and diuretic non-adherence. *Heart* 2000;**83**:12–6.

25 Pujades-Rodriguez M, Timmis A, Stogiannis D, *et al.* Socioeconomic deprivation and the incidence of 12 cardiovascular diseases in 1.9 million women and men: implications for risk prediction and prevention. *PLoS One* 2014;**9**:e104671. doi:10.1371/journal.pone.0104671

26 Mann RD, Andrews EB. *Pharmacovigilance: Second Edition*. 2007. doi:10.1002/9780470059210

27 Walley T, Mantgani A. The UK General Practice Research Database. *Lancet (London, England)* 1997;**350**:1097–9. doi:10.1016/S0140-6736(97)04248-7

28 Chung SC, Gedeborg R, Nicholas O, *et al.* Acute myocardial infarction: A comparison of short-term survival in national outcome registries in Sweden and the UK. *Lancet* 2014;**383**:1305–12. doi:10.1016/S0140-6736(13)62070-X

29 Lewis JD, Bilker WB, Weinstein RB, *et al.* The relationship between time since registration and measured incidence rates in the General Practice Research Database. *Pharmacoepidemiol Drug Saf* 2005;**14**:443–51. doi:10.1002/pds.1115

30 Hazelbag CM, Klungel OH, van Staa TP, *et al.* Left truncation results in substantial bias of the relation between time-dependent exposures and adverse events. *Ann Epidemiol* 2015;**25**:590–6. doi:10.1016/j.annepidem.2015.03.019

31 Alexandrescu R, Bottle A, Jarman B, *et al.* Current ICD10 codes are insufficient to clearly distinguish acute myocardial infarction type: a descriptive study. *BMC Health Serv Res* 2013;**13**:468. doi:10.1186/1472-6963-13-468

32 Dahabreh IJ, Kent DM. Index event bias as an explanation for the paradoxes of recurrence risk

For peer review only

research. *Jama* 2011;**305**:822–3. doi:10.1001/jama.2011.163

For peer review only

## Figure titles and legends

**Figure 1 – Flowchart of study population**

CALIBER=Cardiovascular disease research using Linked Bespoke studies and electronic health Records; CPRD=Clinical Practice Research Datalink; HF=heart failure; MI=myocardial infarction.

**Figure 2 – Incidence of HF after index MI**

Kaplan-Meier curves for crude incidence of HF after MI. **Left panel** 30-day follow-up after index MI. **Right panel** 10 year follow-up (median follow-up time was 3.7 years) in patients who survived the first 30 days and did not develop HF during the first 30 days (30-days event free). HF=heart failure; MI=myocardial infarction. “N at risk” represent the number of subjects at risk at a certain time point. “Cum events” represents the cumulative number of events since the previous time points. A spike histogram is provided (in red) at the top of the graphs providing information on the number of event across time.

**Figure 3 – Incidence of HF after index MI by group**

Kaplan-Meier curves for crude incidence of HF after MI stratified by group. **Top left panel:** 30-day follow-up after index MI stratified by age group. **Top right panel:** 10-year follow-up (median follow-up time was 3.7 years) in 30-day event free patients stratified by age group. Log-rank test of ≥65 year old patients compared to patients aged 50-65 years ($p<0.001$). **Bottom left panel:** 30-day follow-up after index MI stratified by MI type. **Bottom right panel:** 10-year follow-up (median follow-up time was 3.7 years) in patients who survived the first 30 days and did not develop HF during the first 30 days stratified by MI type. Log-rank test of NSTEMI compared to STEMI ($p<0.001$). HF=heart failure; MI=myocardial infarction. N at risk” represent the number of subjects at risk at a certain time point, with the cumulative number of events since the previous time points presented between brackets.

For peer review only

487 Table 1 - Baseline characteristics at index myocardial infarction

| | STEMI patients n = 4,657 | NSTEMI patients n = 19,822 | Total MI patients n = 24,479 | Unknown (%) |
|---|---|---|---|---|
| Follow-up time (years), median (IQR) | 3.3 (1.4–5.7) | 3.9 (1.5–7.1) | 3.7 (1.5–6.7) | 0% |
| Mean age, years (SD) | 65.7 (13.2) | 68.7 (13.2) | 68.1 (13.2) | 0% |
| Male sex | 3311 (71.1%) | 12658 (63.9%) | 15969 (65.2%) | 0% |
| Ethnicity | | | | |
| White | 3466 (76.4%) | 14288 (76.1%) | 17754 (76.2%) | 4.8% |
| Asian | 83 (1.8%) | 327 (1.7%) | 410 (1.8%) | |
| Black | 20 (0.4%) | 63 (0.3%) | 83 (0.4%) | |
| Other | 968 (21.3%) | 4085 (21.7%) | 5053 (21.7%) | |
| Body mass index (BMI in kg/m$^2$) | | | | |
| Underweight (18.5 <) | 22 (1.7%) | 120 (2.0%) | 142 (1.9%) | 70.0% |
| Normal (18.5; 25) | 335 (25.5%) | 1661 (27.4%) | 1996 (27.2%) | |
| Overweight (25, 30) | 557 (43.0%) | 2431 (40.2%) | 2988 (40.7%) | |
| Obese (>30) | 381 (29.4%) | 1842 (30.4%) | 2223 (30.2%) | |
| Index of multiple deprivation | | | | |
| Most deprived quintile | 892 (19.2%) | 3985 (20.2%) | 4877 (20%) | 0.4% |
| | | | | |
| **Risk factors before index MI** | | | | |
| Current smoker | 1235 (51.1%) | 3303 (39.5%) | 4538 (42.1%) | 55.9% |
| Excess alcohol consumption | 55 (9.0%) | 219 (8.1%) | 274 (8.3%) | 86.4% |
| History of atrial fibrillation | 358 (7.7%) | 2210 (11.1%) | 2568 (10.5%) | 0% |
| History of hypertension | 2117 (45.5%) | 10141 (51.2%) | 12258 (50.1%) | 0% |
| History of peripheral arterial disease | 236 (5.1%) | 1475 (7.4%) | 1711 (7%) | 0% |
| Previous revascularization | | | | |
| PCI | 700 (15.0%) | 2044 (10.3%) | 2744 (11.2%) | 0% |
| CABG | 126 (2.7%) | 843 (4.3%) | 969 (4.0%) | 0% |
| Previous TIA | 154 (3.3%) | 998 (5.0%) | 1152 (4.7%) | 0% |
| Previous stroke | 80 (1.7%) | 410 (2.1%) | 490 (2.0%) | 0% |
| History of diabetes | 538 (11.6%) | 2476 (12.6%) | 3014 (12.3%) | 0% |
| History of thyroid disease | 237 (5.1%) | 1374 (6.9%) | 1661 (6.6%) | 0% |
| History of COPD | 304 (6.5%) | 1634 (8.2%) | 1938 (7.9%) | 0% |
| History of non-metastatic cancer | 478 (10.3%) | 2282 (11.5%) | 2760 (11.3%) | 0% |
| | | | | |
| **Vital signs before admission** median (IQR) | | | | |
| Systolic blood pressure, mmHg | 140 (130–153) | 140 (130–154) | 140 (130–154) | 34.2% |
| Diastolic blood pressure, mmHg | 80 (74–88) | 80 (71–88) | 80 (72–88) | 34.2% |
| | | | | |
| **Biomarkers,** median (IQR) | | | | |
| Troponin I (maximum) | 18.4 (3.6–50.0) | 2.2 (0.3–10.0) | 3.88 (0.62–21.4) | 88.1% |
| Troponin T (maximum) | 1.8 (0.60–4.8) | 0.4 (0.14–1.1) | 0.65 (0.18–2) | 90.6% |
| CK (maximum) | 721 (222–1631) | 219 (97–648) | 331 (123–1068) | 79.6% |
| | | | | |
| **Biomarkers before index MI** mean (SD) | | | | |
| Haemoglobin, g/dL, | 14.1 (1.72) | 13.6 (1.84) | 13.7 (1.83) | 67.3% |
| White blood cell count | 8.0 (2.94) | 7.9 (2.92) | 7.9 (2.92) | 69.3% |
| Neutrophil count | 5.0 (2.12) | 5.0 (2.40) | 5.0 (2.35) | 72.1% |
| Platelets | 270 (85.2) | 266 (88.0) | 267 (87.5) | 69.3% |
| Erythrocyte sedimentation rate | 19.5 (18.8) | 22.2 (21.6) | 21.8 (21.2) | 89.5% |
| Creatinine, μmol/L | 98.5 (39.1) | 102.5 (47.3) | 102 (46.0) | 59.0% |
| eGFR - CKD-EPI | 70.4 (19.9) | 66.2 (19.9) | 67.0 (20.0) | 60.7% |
| Random glucose concentration, mmol/L | 7.09 (3.53) | 7.26 (3.89) | 7.23 (3.82) | 76.7% |
| Total cholesterol | 5.39 (1.45) | 5.24 (1.28) | 5.27 (1.31) | 63.7% |
| LDL cholesterol | 3.24 (1.12) | 3.08 (1.10) | 3.11 (1.11) | 81.4% |
| | | | | |
| **STEMI** | | | | |
| Site of infarction | | | | |
| Anterior | 393 (40.2%) | | | 92.6% |
| Primary PCI | 258 (19.3%) | | | 85.3% |
| Prehospital fibrinolysis | 324 (7.0%) | | | 0% |
| Median (IQR) delay from symptom to reperfusion (min) | 150 (97 – 280) | | | 37.5% |
| | | | | |


For peer review only - http://bmjopen.bmj.com/site/about/guidelines.xhtml

| **Prescribed medication before index MI** | | | | |
|---|---|---|---|---|
| Antiplatelet | 867 (18.6%) | 6979 (35.2%) | 7846 (32.1%) | 0% |
| Oral anticoagulant | 67 (1.4%) | 624 (3.1%) | 691 (2.8%) | 0% |
| Statin | 825 (17.7%) | 5297 (26.7%) | 6122 (25.0%) | 0% |
| ACE inhibitor | 676 (14.5%) | 4363 (22.0%) | 5039 (20.6%) | 0% |
| Angiotensin receptor blocker (ARB) | 220 (4.7%) | 1208 (6.1%) | 1428 (5.8%) | 0% |
| Beta-blocker | 766 (16.4%) | 5363 (27.1%) | 6129 (25.0%) | 0% |
| Calcium channel blocker | 804 (17.3%) | 4621 (23.3%) | 5425 (22.2%) | 0% |
| Loop diuretic | 255 (5.5%) | 2188 (11.0%) | 2443 (10.0%) | 0% |
| Aldosterone antagonist | 24 (0.5%) | 198 (1.0%) | 222 (0.9%) | 0% |
| Digoxin | 53 (1.1%) | 515 (2.6%) | 568 (2.3%) | 0% |

IQR=inter-quartile range; SD=standard deviation; MI=myocardial infarction; STEMI=ST-segment-elevation myocardial infarction; PCI=Percutaneous Coronary Intervention; CABG=Coronary Artery Bypass Grafting; TIA=Transient Ischaemic Attack; COPD=Chronic Obstructive Pulmonary Disease; CK=Creatine kinase; ACE=Angiotensin-converting-enzyme. Prehospital and in-hospital fibrinolysis are not mutually exclusive.

For peer review only



For peer review only - http://bmjopen.bmj.com/site/about/guidelines.xhtml

**Table 2 - Hazard ratio for heart failure in patients following a first myocardial infarction using multivariable Cox regression**

| | Model 1<br>n = 24,479<br>n events = 5,775 | Model 2<br>n = 24,479<br>n events = 5,775 | Model 3<br>n = 24,479<br>n events = 5,775 | Model 4<br>n = 24,479<br>n events = 5,775 |
|---|---|---|---|---|
| | HR (95% CI) | HR (95% CI) | HR (95% CI) | HR (95% CI) |
| Age, per 10 years | 1.51 (1.48 – 1.55) | 1.49 (1.46 – 1.53) | 1.46 (1.43 – 1.50) | 1.45 (1.41 – 1.49) |
| Men | 1.06 (1.00 – 1.12) | 1.08 (1.02 – 1.14) | 1.07 (1.01 – 1.13) | 1.06 (1.00 – 1.12) |
| Index of multiple deprivation<br>Q1 (least deprived)<br>Q2<br>Q3<br>Q4<br>Q5 (most deprived) | | overall p = <0.001<br>Reference<br>1.09 (0.99 – 1.20)<br>1.20 (1.09 – 1.33)<br>1.20 (1.08 – 1.33)<br>1.30 (1.16 – 1.45) | overall p = <0.001<br>Reference<br>1.09 (0.99 – 1.20)<br>1.20 (1.08 – 1.32)<br>1.20 (1.08 – 1.33)<br>1.30 (1.16 – 1.45) | overall p = <0.001<br>Reference<br>1.08 (0.99 – 1.19)<br>1.18 (1.07 – 1.31)<br>1.17 (1.06 – 1.30)<br>1.27 (1.13 – 1.41) |
| History of hypertension | | 1.19 (1.12 – 1.26) | 1.17 (1.10 – 1.24) | 1.16 (1.09 – 1.23) |
| History of diabetes | | 1.48 (1.38 – 1.60) | 1.49 (1.38 – 1.60) | 1.45 (1.35 – 1.56) |
| History of atrial fibrillation | | | 1.65 (1.53 – 1.77) | 1.62 (1.51 – 1.75) |
| Type of MI<br>STEMI | | | 1.17 (1.10 – 1.26) | 1.19 (1.11 – 1.27) |
| History of peripheral arterial disease | | | | 1.38 (1.26 – 1.51) |
| History of COPD | | | | 1.28 (1.17 – 1.40) |
| Prescribed ACE inhibitor before MI | | | | 1.06 (0.99 – 1.13) |
| Prescribed Angiotensin receptor blocker (ARB) before MI | | | | 1.00 (0.89 – 1.11) |
| Prescribed Beta-blocker before MI | | | | 0.93 (0.88 – 1.00) |

All analyses presented in table 2 were performed on a complete case dataset of 24,479 subjects. HR=hazard ratio; CI=confidence interval; Q=quintile; MI=myocardial infarction; NSTEMI=non-ST-segment-elevation myocardial infarction; STEMI=ST-segment-elevation myocardial infarction; COPD=chronic obstructive pulmonary disease; ACE=Angiotensin-converting enzyme.

For peer review only

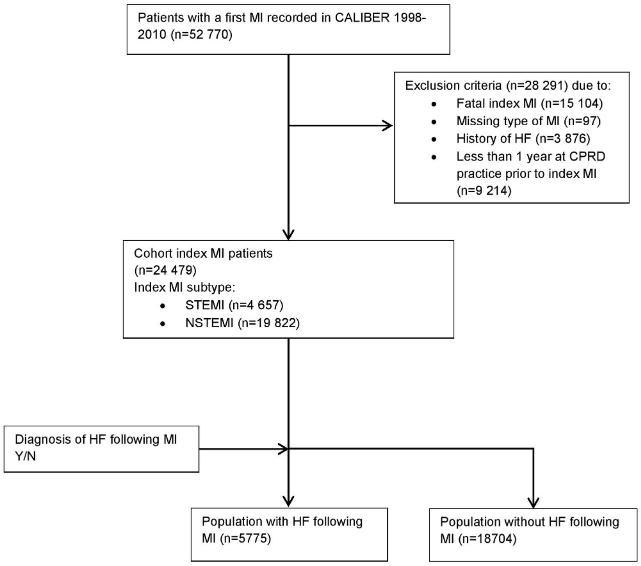


209x297mm (300 x 300 DPI)

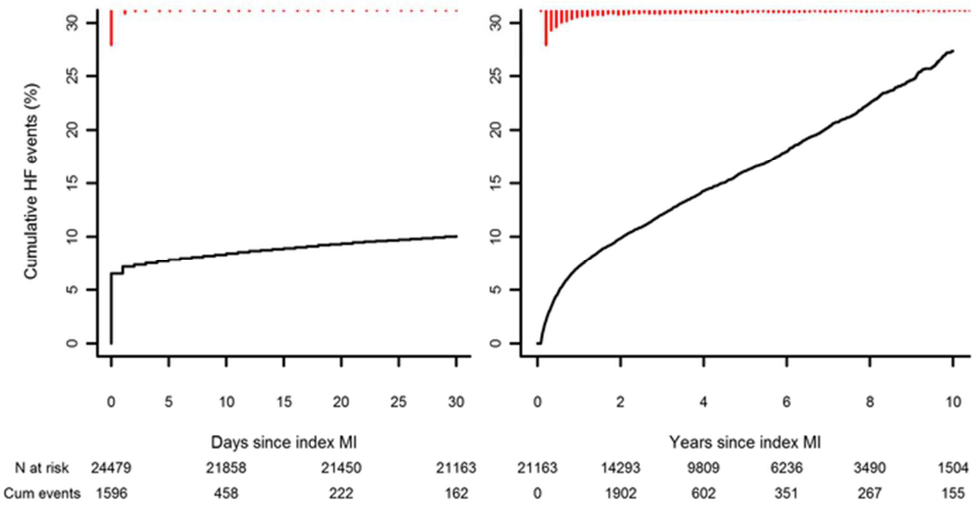


59x35mm (300 x 300 DPI)

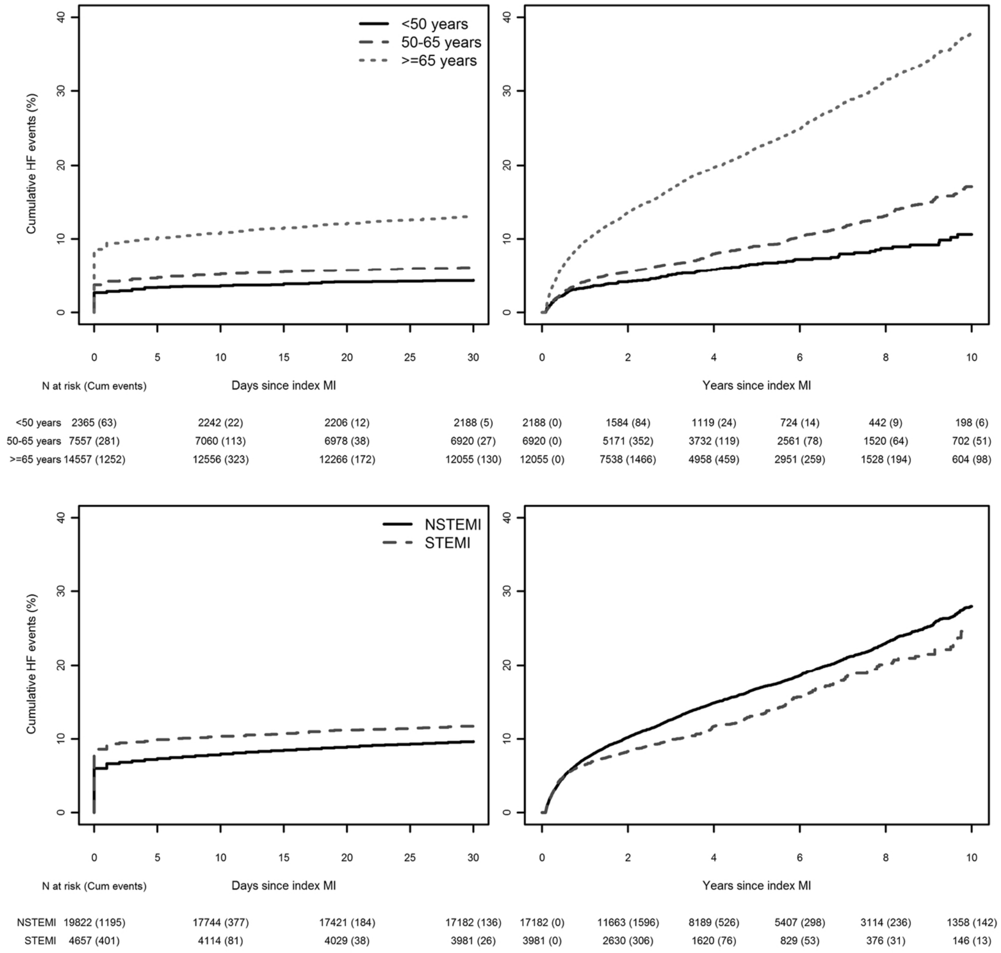


139x139mm (300 x 300 DPI)

**Supplementary Data**

# An Electronic Health Records cohort study on Heart Failure Following Myocardial Infarction in England: Incidence and Prognostic Factors

Johannes M.I.H. Gho, Amand F. Schmidt, Laura Pasea, Stefan Koudstaal, Mar Pujades-Rodriguez, Spiros Denaxas, Anoop D. Shah, Riyaz S. Patel, Chris P. Gale, Arno W. Hoes, John G. Cleland, Harry Hemingway, Folkert W. Asselbergs

For peer review only

## Phenotype coding lists

**CALIBER myocardial infarction phenotype**
Fatal or non-fatal myocardial infarction (MI) as recorded in any of the constituent CALIBER data sources. The type of myocardial infarction (ST elevation MI, STEMI; or non ST elevation MI, NSTEMI) is recorded only in MINAP and CPRD.

Read codes used to identify myocardial infarction in primary care records (Clinical Research Practice Datalink)

| Category | Clinical code | Clinical term | Datasource lookup |
|---|---|---|---|
| **STEMI (3)** | G30X000 | Acute ST segment elevation myocardial infarction | 12229 |
| **NSTEMI (4)** | G307100 | Acute non-ST segment elevation myocardial infarction | 10562 |
| **Acute MI not further specified (5)** | 323..00 | ECG: myocardial infarction | 7783 |
| **Acute MI not further specified (5)** | 3233.00 | ECG: antero-septal infarct. | 26975 |
| **Acute MI not further specified (5)** | 3234.00 | ECG:posterior/inferior infarct | 26972 |
| **Acute MI not further specified (5)** | 3235.00 | ECG: subendocardial infarct | 55401 |
| **Acute MI not further specified (5)** | 3236.00 | ECG: lateral infarction | 52705 |
| **Acute MI not further specified (5)** | 323Z.00 | ECG: myocardial infarct NOS | 59032 |
| **Acute MI not further specified (5)** | 889A.00 | Diab mellit insulin-glucose infus acute myocardial infarct | 61670 |
| **Acute MI not further specified (5)** | G30..00 | Acute myocardial infarction | 241 |
| **Acute MI not further specified (5)** | G30..12 | Coronary thrombosis | 2491 |
| **Acute MI not further specified (5)** | G30..13 | Cardiac rupture following myocardial infarction (MI) | 30421 |
| **Acute MI not further specified (5)** | G30..15 | MI - acute myocardial infarction | 1677 |
| **Acute MI not further specified (5)** | G30..16 | Thrombosis - coronary | 13571 |
| **Acute MI not further specified (5)** | G300.00 | Acute anterolateral infarction | 12139 |
| **Acute MI not further specified (5)** | G301.00 | Other specified anterior myocardial infarction | 5387 |
| **Acute MI not further specified (5)** | G301000 | Acute anteroapical infarction | 40429 |
| **Acute MI not further specified (5)** | G301100 | Acute anteroseptal infarction | 17872 |
| **Acute MI not further specified (5)** | G301z00 | Anterior myocardial infarction NOS | 14897 |
| **Acute MI not further specified (5)** | G302.00 | Acute inferolateral infarction | 8935 |
| **Acute MI not further specified (5)** | G303.00 | Acute inferoposterior infarction | 29643 |
| **Acute MI not further specified (5)** | G304.00 | Posterior myocardial infarction NOS | 23892 |
| **Acute MI not further specified (5)** | G305.00 | Lateral myocardial infarction NOS | 14898 |
| **Acute MI not further specified (5)** | G306.00 | True posterior myocardial infarction | 63467 |
| **Acute MI not further specified (5)** | G307.00 | Acute subendocardial infarction | 3704 |
| **Acute MI not further specified (5)** | G307000 | Acute non-Q wave infarction | 9507 |

For peer review only

| | | | |
|---|---|---|---|
| **Acute MI not further specified (5)** | G308.00 | Inferior myocardial infarction NOS | 1678 |
| **Acute MI not further specified (5)** | G309.00 | Acute Q-wave infarct | 30330 |
| **Acute MI not further specified (5)** | G30B.00 | Acute posterolateral myocardial infarction | 32854 |
| **Acute MI not further specified (5)** | G30X.00 | Acute transmural myocardial infarction of unspecif site | 29758 |
| **Acute MI not further specified (5)** | G30y.00 | Other acute myocardial infarction | 34803 |
| **Acute MI not further specified (5)** | G30y000 | Acute atrial infarction | 28736 |
| **Acute MI not further specified (5)** | G30y100 | Acute papillary muscle infarction | 62626 |
| **Acute MI not further specified (5)** | G30y200 | Acute septal infarction | 41221 |
| **Acute MI not further specified (5)** | G30yz00 | Other acute myocardial infarction NOS | 46017 |
| **Acute MI not further specified (5)** | G30z.00 | Acute myocardial infarction NOS | 14658 |
| **Acute MI not further specified (5)** | G31y100 | Microinfarction of heart | 68357 |
| **Acute MI not further specified (5)** | G38..00 | Postoperative myocardial infarction | 32272 |
| **Acute MI not further specified (5)** | G380.00 | Postoperative transmural myocardial infarction anterior wall | 46112 |
| **Acute MI not further specified (5)** | G381.00 | Postoperative transmural myocardial infarction inferior wall | 46276 |
| **Acute MI not further specified (5)** | G384.00 | Postoperative subendocardial myocardial infarction | 41835 |
| **Acute MI not further specified (5)** | G38z.00 | Postoperative myocardial infarction, unspecified | 68748 |
| **Acute MI not further specified (5)** | Gyu3400 | [X]Acute transmural myocardial infarction of unspecif site | 96838 |

Hospital Episode Statistics

| **Category (code)** | ICD10 code | ICD10 term |
|---|---|---|
| **Acute MI not further specified (5)** | I21 | Acute myocardial infarction |

OPCS

| **Category (code)** | OPCS 4 | OPCS 4 term |
|---|---|---|
| **Transluminal coronary thrombolysis (2)** | K50.2 | Percutaneous transluminal coronary thrombolysis using streptokinase |
| **Transluminal coronary thrombolysis (2)** | K50.3 | Percutaneous transluminal injection of therapeutic substance into coronary artery NEC |

Office for National Statistics (ONS)

| **Category (code)** | ICD10 code | ICD10 term |
|---|---|---|
| **Death from MI (3)** | I21 | Acute myocardial infarction |
| **Death from MI (3)** | I22 | Subsequent myocardial infarction |
| **Death from MI (3)** | I23 | Certain current complications following acute myocardial infarction |

Office for National Statistics (ONS) prior to 2000 (ICD9)

| **Category (code)** | ICD9 code | ICD9 term |
|---|---|---|
| **Death from myocardial infarction (3)** | 410 | Acute myocardial infarction |
| **Death from myocardial infarction (3)** | 4110 | Other acute and subacute forms of ischemic heart disease ; Postmyocardial infarction syndrome |

For peer review only

| | | |
|---|---|---|
| **Death from myocardial infarction (3)** | 4297 | Ill-defined descriptions and complications of heart disease ; Certain sequelae of myocardial infarction, not elsewhere classified |

Myocardial Ischaemia National Audit Project (MINAP)

1 Acute STEMI

2 Acute NSTEMI

## Phenotype coding lists

**CALIBER myocardial infarction phenotype**

Fatal or non-fatal myocardial infarction (MI) as recorded in any of the constituent CALIBER data sources. The type of myocardial infarction (ST elevation MI, STEMI; or non ST elevation MI, NSTEMI) is recorded only in MINAP and CPRD.

Read codes used to identify myocardial infarction in primary care records (Clinical Research Practice Datalink)

| Category | Clinical code | Clinical term | Datasource lookup |
|---|---|---|---|
| **STEMI (3)** | G30X000 | Acute ST segment elevation myocardial infarction | 12229 |
| **NSTEMI (4)** | G307100 | Acute non-ST segment elevation myocardial infarction | 10562 |
| **Acute MI not further specified (5)** | 323..00 | ECG: myocardial infarction | 7783 |
| **Acute MI not further specified (5)** | 3233.00 | ECG: antero-septal infarct. | 26975 |
| **Acute MI not further specified (5)** | 3234.00 | ECG:posterior/inferior infarct | 26972 |
| **Acute MI not further specified (5)** | 3235.00 | ECG: subendocardial infarct | 55401 |
| **Acute MI not further specified (5)** | 3236.00 | ECG: lateral infarction | 52705 |
| **Acute MI not further specified (5)** | 323Z.00 | ECG: myocardial infarct NOS | 59032 |
| **Acute MI not further specified (5)** | 889A.00 | Diab mellit insulin-glucose infus acute myocardial infarct | 61670 |
| **Acute MI not further specified (5)** | G30..00 | Acute myocardial infarction | 241 |
| **Acute MI not further specified (5)** | G30..12 | Coronary thrombosis | 2491 |
| **Acute MI not further specified (5)** | G30..13 | Cardiac rupture following myocardial infarction (MI) | 30421 |
| **Acute MI not further specified (5)** | G30..15 | MI - acute myocardial infarction | 1677 |
| **Acute MI not further specified (5)** | G30..16 | Thrombosis - coronary | 13571 |
| **Acute MI not further specified (5)** | G300.00 | Acute anterolateral infarction | 12139 |
| **Acute MI not further specified (5)** | G301.00 | Other specified anterior myocardial infarction | 5387 |
| **Acute MI not further specified (5)** | G301000 | Acute anteroapical infarction | 40429 |
| **Acute MI not further specified (5)** | G301100 | Acute anteroseptal infarction | 17872 |
| **Acute MI not further specified (5)** | G301z00 | Anterior myocardial infarction NOS | 14897 |
| **Acute MI not further specified (5)** | G302.00 | Acute inferolateral infarction | 8935 |
| **Acute MI not further specified (5)** | G303.00 | Acute inferoposterior infarction | 29643 |
| **Acute MI not further specified (5)** | G304.00 | Posterior myocardial infarction NOS | 23892 |
| **Acute MI not further specified (5)** | G305.00 | Lateral myocardial infarction NOS | 14898 |

For peer review only

| | | | |
|---|---|---|---|
| **Acute MI not further specified (5)** | G306.00 | True posterior myocardial infarction | 63467 |
| **Acute MI not further specified (5)** | G307.00 | Acute subendocardial infarction | 3704 |
| **Acute MI not further specified (5)** | G307000 | Acute non-Q wave infarction | 9507 |
| **Acute MI not further specified (5)** | G308.00 | Inferior myocardial infarction NOS | 1678 |
| **Acute MI not further specified (5)** | G309.00 | Acute Q-wave infarct | 30330 |
| **Acute MI not further specified (5)** | G30B.00 | Acute posterolateral myocardial infarction | 32854 |
| **Acute MI not further specified (5)** | G30X.00 | Acute transmural myocardial infarction of unspecif site | 29758 |
| **Acute MI not further specified (5)** | G30y.00 | Other acute myocardial infarction | 34803 |
| **Acute MI not further specified (5)** | G30y000 | Acute atrial infarction | 28736 |
| **Acute MI not further specified (5)** | G30y100 | Acute papillary muscle infarction | 62626 |
| **Acute MI not further specified (5)** | G30y200 | Acute septal infarction | 41221 |
| **Acute MI not further specified (5)** | G30yz00 | Other acute myocardial infarction NOS | 46017 |
| **Acute MI not further specified (5)** | G30z.00 | Acute myocardial infarction NOS | 14658 |
| **Acute MI not further specified (5)** | G31y100 | Microinfarction of heart | 68357 |
| **Acute MI not further specified (5)** | G38..00 | Postoperative myocardial infarction | 32272 |
| **Acute MI not further specified (5)** | G380.00 | Postoperative transmural myocardial infarction anterior wall | 46112 |
| **Acute MI not further specified (5)** | G381.00 | Postoperative transmural myocardial infarction inferior wall | 46276 |
| **Acute MI not further specified (5)** | G384.00 | Postoperative subendocardial myocardial infarction | 41835 |
| **Acute MI not further specified (5)** | G38z.00 | Postoperative myocardial infarction, unspecified | 68748 |
| **Acute MI not further specified (5)** | Gyu3400 | [X]Acute transmural myocardial infarction of unspecif site | 96838 |

Hospital Episode Statistics

| **Category (code)** | ICD10 code | ICD10 term |
|---|---|---|
| **Acute MI not further specified (5)** | I21 | Acute myocardial infarction |

OPCS

| **Category (code)** | OPCS 4 | OPCS 4 term |
|---|---|---|
| **Transluminal coronary thrombolysis (2)** | K50.2 | Percutaneous transluminal coronary thrombolysis using streptokinase |
| **Transluminal coronary thrombolysis (2)** | K50.3 | Percutaneous transluminal injection of therapeutic substance into coronary artery NEC |

Office for National Statistics (ONS)

| **Category (code)** | ICD10 code | ICD10 term |
|---|---|---|
| **Death from MI (3)** | I21 | Acute myocardial infarction |
| **Death from MI (3)** | I22 | Subsequent myocardial infarction |
| **Death from MI (3)** | I23 | Certain current complications following acute myocardial infarction |

Office for National Statistics (ONS) prior to 2000 (ICD9)

For peer review only

| Category (code) | ICD9 code | ICD9 term |
|---|---|---|
| **Death from myocardial infarction (3)** | 410 | Acute myocardial infarction |
| **Death from myocardial infarction (3)** | 4110 | Other acute and subacute forms of ischemic heart disease ; Postmyocardial infarction syndrome |
| **Death from myocardial infarction (3)** | 4297 | Ill-defined descriptions and complications of heart disease ; Certain sequelae of myocardial infarction, not elsewhere classified |

Myocardial Ischaemia National Audit Project (MINAP)

1 Acute STEMI

2 Acute NSTEMI

**CALIBER heart failure phenotype**

Primary care
1. Diagnosis of left ventricular dysfunction during echocardiogram during a consultation
2. Diagnosis of heart failure of valvular cause during a consultation
3. Diagnosis of heart failure due to hypertension during a consultation
4. Diagnosis of heart failure due to unspecified cause during a consultation
5. Diagnosis of heart failure not otherwise specified during a consultation

Secondary care
1. Diagnosis of heart failure due to hypertension as the primary diagnosis during a hospitalization
2. Diagnosis of heart failure not otherwise specified as the primary diagnosis during a hospitalization

Mortality
1. Hypertensive heart failure as the underlying cause of death
2. Heart failure not otherwise specified as the underlying cause of death

Read codes used to identify myocardial infarction in primary care records (Clinical Research Practice Datalink)

| **Category (code)** | **Clinical code** | **Clinical term** | **Datasource lookup** |
|---|---|---|---|
| **Heart failure due to valvular disease (3)** | G580400 | Congestive heart failure due to valvular disease | 94870 |
| **Heart failure due to hypertension (4)** | G210.00 | Malignant hypertensive heart disease | 50157 |
| **Heart failure due to hypertension (4)** | G210000 | Malignant hypertensive heart disease without CCF | 95334 |
| **Heart failure due to hypertension (4)** | G210100 | Malignant hypertensive heart disease with CCF | 72668 |
| **Heart failure due to hypertension (4)** | G211100 | Benign hypertensive heart disease with CCF | 52127 |
| **Heart failure due to hypertension (4)** | G21z100 | Hypertensive heart disease NOS with CCF | 62718 |
| **Heart failure due to hypertension (4)** | G230.00 | Malignant hypertensive heart and renal disease | 67232 |
| **Heart failure due to hypertension (4)** | G232.00 | Hypertensive heart&renal dis wth (congestive) heart failure | 21837 |
| **Heart failure due to hypertension (4)** | G234.00 | Hyperten heart&renal dis+both(congestv)heart and renal fail | 57987 |
| **Heart failure due to other cause (5)** | G1yz100 | Rheumatic left ventricular failure | 22262 |
| **Heart failure NOS (6)** | 1O1..00 | Heart failure confirmed | 9913 |
| **Heart failure NOS (6)** | 662W.00 | Heart failure annual review | 30779 |
| **Heart failure NOS (6)** | 662p.00 | Heart failure 6 month review | 83502 |
| **Heart failure NOS (6)** | 8B29.00 | Cardiac failure therapy | 24503 |
| **Heart failure NOS (6)** | 8H2S.00 | Admit heart failure emergency | 32898 |
| **Heart failure NOS (6)** | 9Or0.00 | Heart failure review completed | 19380 |
| **Heart failure NOS (6)** | G400.00 | Acute cor pulmonale | 8464 |
| **Heart failure NOS (6)** | G41z.11 | Chronic cor pulmonale | 5695 |
| **Heart failure NOS (6)** | G554000 | Congestive cardiomyopathy | 5141 |
| **Heart failure NOS (6)** | G554011 | Congestive obstructive cardiomyopathy | 68766 |
| **Heart failure NOS (6)** | G58..00 | Heart failure | 2062 |
| **Heart failure NOS (6)** | G58..11 | Cardiac failure | 1223 |
| **Heart failure NOS (6)** | G580.00 | Congestive heart failure | 398 |

For peer review only

| | | | |
|---|---|---|---|
| **Heart failure NOS (6)** | G580.11 | Congestive cardiac failure | 2906 |
| **Heart failure NOS (6)** | G580.12 | Right heart failure | 10079 |
| **Heart failure NOS (6)** | G580.13 | Right ventricular failure | 10154 |
| **Heart failure NOS (6)** | G580.14 | Biventricular failure | 9524 |
| **Heart failure NOS (6)** | G580000 | Acute congestive heart failure | 23707 |
| **Heart failure NOS (6)** | G580100 | Chronic congestive heart failure | 32671 |
| **Heart failure NOS (6)** | G580200 | Decompensated cardiac failure | 27884 |
| **Heart failure NOS (6)** | G580300 | Compensated cardiac failure | 11424 |
| **Heart failure NOS (6)** | G581.00 | Left ventricular failure | 884 |
| **Heart failure NOS (6)** | G581.11 | Asthma - cardiac | 23481 |
| **Heart failure NOS (6)** | G581.13 | Impaired left ventricular function | 5942 |
| **Heart failure NOS (6)** | G581000 | Acute left ventricular failure | 5255 |
| **Heart failure NOS (6)** | G582.00 | Acute heart failure | 27964 |
| **Heart failure NOS (6)** | G58z.00 | Heart failure NOS | 4024 |
| **Heart failure NOS (6)** | G58z.12 | Cardiac failure NOS | 17278 |
| **Heart failure NOS (6)** | G5yy900 | Left ventricular systolic dysfunction | 8966 |
| **Heart failure NOS (6)** | G5yyA00 | Left ventricular diastolic dysfunction | 12550 |
| **Heart failure NOS (6)** | R2y1000 | [D]Cardiorespiratory failure | 20324 |
| **Left ventricular dysfunction (3)** | 585f.00 | Echocardiogram shows left ventricular systolic dysfunction | 11284 |
| **Left ventricular dysfunction (3)** | 585g.00 | Echocardiogram shows left ventricular diastolic dysfunction | 11351 |

Hospital Episode Statistics

| **Category (code)** | **ICD10 code** | **ICD10 term** |
|---|---|---|
| **HF - hypertension cause (4)** | I110 | Hypertensive heart disease with (congestive) heart failure |
| **HF - hypertension cause (4)** | I130 | Hypertensive heart and renal disease with (congestive) heart failure |
| **HF - hypertension cause (4)** | I132 | Hypertensive heart and renal disease with both (congestive) heart failure and renal failure |
| **HF - other unspecified cause (5)** | I260 | Pulmonary embolism with mention of acute cor pulmonale |
| **HF - not otherwise specified (6)** | I50 | Heart failure |

Office for National Statistics (ONS)

| **Category (code)** | **ICD10 code** | **ICD10 term** |
|---|---|---|
| **Hypertensive heart failure (4)** | I110 | Hypertensive heart disease with (congestive) heart failure |
| **Hypertensive heart failure (4)** | I130 | Hypertensive heart and renal disease with (congestive) heart failure |
| **Hypertensive heart failure (4)** | I132 | Hypertensive heart and renal disease with both (congestive) heart failure and renal failure |
| **Heart failure with other specified cause (5)** | I260 | Pulmonary embolism with mention of acute cor pulmonale |
| **Heart failure with unspecified cause (6)** | I50 | Heart failure |

Office for National Statistics (ONS) prior to 2000 (ICD9)

| **Category (code)** | **ICD9 code** | **ICD9 term** |
|---|---|---|
| **Heart failure with other specified cause (5)** | 4151 | Acute pulmonary heart disease ; Pulmonary embolism and infarction |
| **Heart failure with unspecified cause (6)** | 428 | Heart failure |

Myocardial Ischaemia National Audit Project (MINAP)

1 CCF

**CALIBER baseline definitions**

See also: https://www.caliberresearch.org

For peer review only

Definitions used for smoking at baseline
CPRD:
**smoking_cessation_gprd**
Prescription of smoking cessation medication in primary care. Currently lookup is performed from using BNF chapter 4.10.2.
**smoking_status_gprd**
An individual record of smoking status in primary care. A patient may have multiple records of smoking status.
HES:
**smoking_status_hes**
Smoking status recorded in HES. Currently there are only ICD-10 codes for smoking, no codes for ex-smoking or non-smoking.

Definitions used for hypertension at baseline
CPRD:
**ht_gprd**
Diagnosis of hypertension
HES:
**ht_hes**
Hypertension diagnosis recorded in secondary care.

Definitions used for diabetes at baseline
CPRD:
**dm_gprd**
Type of diabetes diagnosis at a particular point in time.
**diabdiag_gprd**
GP diagnosis of diabetes mellitus using a specific type I or type II diagnosis in the Read diagnosis chapter (i.e. codes starting with C08, C09, C10E, C10F). Vague terms such as `juvenile onset diabetes` or `insulin dependent diabetes` are not included.
HES:
**dm_hes**
Diagnosis of diabetes in secondary care (HES).

For peer review only

Supplementary Methods

**Ethical approval**
CALIBER was approved by the Lewisham Local Research Ethics Committee (ref: 09/H0810/16 date: 08/04/2009) and the Ethics and Confidentiality Committee (ECC) (ref: ECC 2-06(b)/2009 CALIBER dataset). CALIBER has been registered with the University College London Data Protection Officer (ref: Z6364106/2009/2/26). CALIBER EHR data are anonymised; individual informed consent was not sought from study participants.

**Multiple imputation algorithm**
Multiple imputation was implemented using the 'mice' package for R, to replace missing values in prognostic covariates (body mass index, smoking, systolic and diastolic blood pressure).[1] Imputation models included general practice and 3-year periods from 1998 to 2010 of index myocardial infarction (MI) and:

1. All the baseline covariates as used in the multivariable analysis (age, sex, index of multiple deprivation, hypertension, diabetes, type of MI, body mass index, smoking, systolic blood pressure, diastolic blood pressure);
2. Baseline measurements of covariates not considered in the multivariable analysis (alcohol intake, haemoglobin, white blood cell count, creatinine, alanine aminotransferase (ALAT), total cholesterol, HDL cholesterol);
3. Baseline medications prior to index MI (antiplatelet, oral anticoagulant, statin, ACE inhibitor, angiotensin receptor blocker (ARB), beta-blocker, calcium channel blocker, loop diuretic, aldosterone antagonist, digoxin);
4. Coexisting medical conditions (history of atrial fibrillation, peripheral arterial disease, depression, cancer, chronic obstructive pulmonary disease);
5. The Nelson-Aalen hazard and the event status for the outcomes analysed.

Missing data was multiply imputed and across 20 datasets a (stratified) multivariable Cox regression model was fit and results were combined using Rubin's rules.[2]

References

1. van Buuren S. Multiple imputation of discrete and continuous data by fully conditional specification. Stat Methods Med Res 2007;16:219-42.
2. Carpenter JR, Kenward MG. *Multiple Imputation and its Application*: Wiley; 2013.

For peer review only

**Table S1 - Hazard ratio for heart failure in patients following a first myocardial infarction using the competing risks regression based on Fine and Gray's proportional subhazards model to account for the competing risk of all cause mortality**

| | Model 1 | Model 2 | Model 3 | Model 4 |
|---|---|---|---|---|
| | HR (95% CI) | HR (95% CI) | HR (95% CI) | HR (95% CI) |
| Age, per 10 years increase | 1.41 (1.38 – 1.44) | 1.39 (1.36 – 1.42) | 1.36 (1.33 – 1.39) | 1.35 (1.32 – 1.38) |
| Men | 1.04 (0.99 – 1.10) | 1.07 (1.01 – 1.13) | 1.06 (1.00 – 1.12) | 1.05 (1.00 – 1.11) |
| Index of multiple deprivation | | overall p = <0.001 | overall p = <0.001 | overall p = <0.001 |
| Q1 | | Reference | Reference | Reference |
| Q2 | | 1.11 (1.03 – 1.21) | 1.11 (1.02 – 1.20) | 1.10 (1.01 – 1.20) |
| Q3 | | 1.21 (1.12 – 1.32) | 1.20 (1.11 – 1.31) | 1.19 (1.09 – 1.29) |
| Q4 | | 1.24 (1.14 – 1.35) | 1.24 (1.14 – 1.34) | 1.22 (1.12 – 1.32) |
| Q5 | | 1.36 (1.25 – 1.47) | 1.35 (1.25 – 1.47) | 1.32 (1.21 – 1.43) |
| History of hypertension | | 1.15 (1.09 – 1.22) | 1.13 (1.07 – 1.19) | 1.14 (1.08 – 1.21) |
| History of diabetes | | 1.39 (1.29 – 1.49) | 1.38 (1.29 – 1.48) | 1.36 (1.27 – 1.47) |
| History of atrial fibrillation | | | 1.50 (1.40 – 1.61) | 1.49 (1.39 – 1.60) |
| Type of MI | | | | |
| STEMI | | | 1.10 (1.03 – 1.18) | 1.11 (1.03 – 1.18) |
| History of peripheral arterial disease | | | | 1.29 (1.19 – 1.40) |
| History of COPD | | | | 1.18 (1.09 – 1.29) |
| ACE inhibitor use before MI | | | | 1.01 (0.95 – 1.08) |
| Angiotensin receptor blocker (ARB) use before MI | | | | 0.94 (0.85 – 1.05) |
| Beta-blocker use before MI | | | | 0.94 (0.88 – 1.00) |

Complete case analysis with the addition of clinically relevant variables per model (n = 24,479, n events = 5,775). Overall p-values are shown for categorical covariates. ACE=Angiotensin-converting enzyme; CI=confidence interval; COPD=chronic obstructive pulmonary disease; MI=myocardial infarction; NSTEMI=non-ST-segment-elevation myocardial infarction; STEMI=ST-segment-elevation myocardial infarction; Q=quintile; HR=ratio of the subdistribution hazards.

For peer review only

**Table S2 - Hazard ratio for heart failure in patients following a first myocardial infarction in sensitivity analyses**

| | Model 5<br>Cox regression<br>complete case without multiple imputation<br>n = 4,898<br>n events = 1,159 | Model 5<br>Cox regression<br>with multiple imputation<br>n = 24,479<br>n events = 5,775 | Model 5<br>Competing risk analysis<br>with complete case<br>n = 4,898<br>n events = 1,159 |
|---|---|---|---|
| | HR (95% CI) | HR (95% CI) | HR (95% CI) |
| Age, per 10 years increase | 1.48 (1.38 – 1.60) | 1.47 (1.43 – 1.51) | 1.44 (1.35 – 1.54) |
| Men | 1.03 (0.89 – 1.19) | 1.07 (1.01 – 1.13) | 1.07 (0.95 – 1.22) |
| Body mass index (BMI in kg/m$^2$) | p = 0.005 | p = <0.001 | p = < 0.001 |
| Underweight (18.5 <) | 0.78 (0.48 – 1.32) | 0.76 (0.58 – 0.99) | 0.71 (0.45 – 1.12) |
| Normal (18.5; 25) | Reference | Reference | Reference |
| Overweight (25, 30) | 0.84 (0.71 – 0.99) | 0.84 (0.76 – 0.92) | 0.84 (0.73 – 0.97) |
| Obese (>30) | 1.12 (0.94 – 1.34) | 0.98 (0.90 – 1.08) | 1.17 (1.00 – 1.36) |
| Index of multiple deprivation | p = 0.040 | p < 0.001 | p = < 0.001 |
| Q1 | Reference | Reference | Reference |
| Q2 | 1.22 (0.94 – 1.58) | 1.08 (0.98 – 1.19) | 1.35 (1.10 – 1.66) |
| Q3 | 1.34 (1.03 – 1.75) | 1.17 (1.06 – 1.30) | 1.40 (1.14 – 1.71) |
| Q4 | 1.23 (0.93 – 1.63) | 1.16 (1.05 – 1.29) | 1.31 (1.07 – 1.61) |
| Q5 | 1.45 (1.09 – 1.92) | 1.25 (1.12 – 1.40) | 1.61 (1.33 – 1.96) |
| Smoking status | p = 0.792 | p = 0.012 | p = 0.952 |
| Non-smoker | Reference | Reference | Reference |
| Current smoker | 0.95 (0.77 – 1.14) | 1.14 (1.04 – 1.25) | 1.02 (0.86 – 1. 20) |
| Ex-smoker | 0.98 (0.83 – 1.15) | 1.03 (0.94 – 1.12) | 0.99 (0.87 – 1.14) |
| History of hypertension | 1.14 (0.96 – 1.35) | 1.16 (1.09 – 1.24) | 1.18 (1.02 – 1.37) |
| History of diabetes | 1.28 (1.09 – 1.49) | 1.46 (1.36 – 1.58) | 1.23 (1.08 – 1.39) |
| History of atrial fibrillation | 1.49 (1.25 – 1.76) | 1.63 (1.51 – 1.75) | 1.37 (1.18 – 1.59) |
| Type of MI<br>STEMI | 1.15 (0.97 – 1.37) | 1.18 (1.10 – 1.27) | 1.10 (0.95 – 1.28) |
| History of peripheral arterial disease | 1.53 (1.25 – 1.87) | 1.37 (1.25 – 1.50) | 1.33 (1.13 – 1.57) |
| History of COPD | 1.21 (0.99 – 1.48) | 1.24 (1.15 – 1.38) | 1.22 (1.03 – 1.44) |
| Systolic blood pressure, per 10 mmHg increase | 0.99 (0.95 – 1.03) | 1.00 (0.98 – 1.02) | 1.01 (0.97 – 1.04) |
| Diastolic blood pressure, per 10 mmHg increase | 1.01 (0.94 – 1.09) | 0.99 (0.96 – 1.03) | 0.99 (0.93 – 1.06) |
| ACE inhibitor use before MI | 1.01 (0.87 – 1.17) | 1.06 (0.99 – 1.14) | 1.07 (0.94 – 1.21) |
| Angiotensin receptor blocker (ARB) use before MI | 0.99 (0.95 – 1.03) | 1.00 (0.90 – 1.12) | 0.93 (0.78 – 1.12) |
| Beta-blocker use before MI | 1.01 (0.94 – 1.09) | 0.94 (0.88 – 1.00) | 0.90 (0.79 – 1.02) |

Using multivariable Cox regression without and with multiple imputation and the competing risks regression based on Fine and Gray's proportional subhazards model to account for the competing risk of all cause mortality (n = 4898, n events = 1159). Additional prognostic covariates: body mass index, smoking, systolic and diastolic blood pressure. Overall p-values are shown for categorical covariates. HR=hazard ratio; CI=confidence interval; MI=myocardial infarction; NSTEMI=non-ST-segment-elevation myocardial infarction; STEMI=ST-segment-elevation myocardial infarction; COPD=chronic obstructive pulmonary disease; ACE=Angiotensin-converting enzyme; Q=quintile.

For peer review only

**Table S3 - Hazard ratio for heart failure in patients following a first myocardial infarction stratified on having a previous cancer diagnosis or not.**

| | Model 4<br>**Subjects without a history of cancer**<br>n = 21,719<br>n events = 5,029 | Model 4<br>**Subjects with a history of cancer**<br>n = 2,760<br>n events = 746 |
|---|---|---|
| | HR (95% CI) | HR (95% CI) |
| Age, per 10 years increase | 1.49 (1.45 – 1.53) | 1.45 (1.30 – 1.61) |
| Men | 1.08 (1.01 – 1.15) | 1.13 (0.93 – 1.37) |
| Index of multiple deprivation | overall p = <0.001 | overall p = <0.001 |
| Q1 | Reference | Reference |
| Q2 | 1.08 (0.97 – 1.20) | 1.07 (0.77 – 1.48) |
| Q3 | 1.17 (1.05 – 1.31) | 1.27 (0.91 – 1.78) |
| Q4 | 1.17 (1.04 – 1.31) | 1.14 (0.79 – 1.66) |
| Q5 | 1.25 (1.10 – 1.41) | 1.43 (0.97 – 2.11) |
| History of hypertension | 1.11 (1.03 – 1.18) | 1.45 (1.16 – 1.81) |
| History of diabetes | 1.53 (1.41 – 1.66) | 1.19 (0.91 – 1.55) |
| History of atrial fibrillation | 1.62 (1.49 – 1.76) | 1.67 (1.33 – 2.09) |
| Type of MI | | |
| STEMI | 1.18 (1.09 – 1.28) | 1.01 (0.77 – 1.31) |
| History of peripheral arterial disease | 1.39 (1.26 – 1.54) | 1.19 (0.88 – 1.62) |
| History of COPD | 1.30 (1.18 – 1.43) | 1.36 (1.03 – 1.81) |
| ACE inhibitor use before MI | 1.11 (1.03 – 1.20) | 1.00 (0.79 – 1.26) |
| Angiotensin receptor blocker (ARB) use before MI | 1.06 (0.93 – 1.20) | 0.92 (0.65 – 1.31) |
| Beta-blocker use before MI | 0.97 (0.90 – 1.04) | 0.99 (0.79 – 1.24) |

All analyses are based on a complete case analysis. Overall p-values are shown for categorical covariates. ACE=Angiotensin-converting enzyme; CI=confidence interval; COPD=chronic obstructive pulmonary disease; MI=myocardial infarction; NSTEMI=non-ST-segment-elevation myocardial infarction; STEMI=ST-segment-elevation myocardial infarction; Q=quintile.

For peer review only

**Supplementary Figure 1 – Cumulative incidence curves for HF accounting for mortality as first event after index MI**

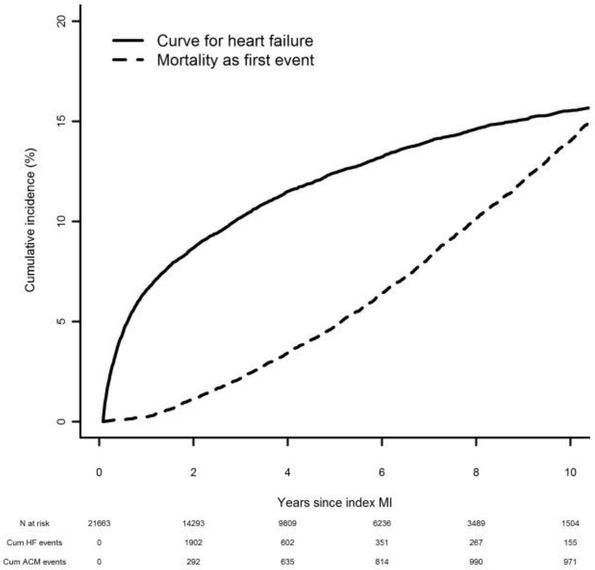


Cumulative incidence curves adjusted for competing risk of mortality as first event. 10-year follow-up in patients who survived the first 30 days and did not develop HF during the first 30 days (30 days event free).

For peer review only

STROBE Statement—checklist of items that should be included in reports of observational studies

| | Item No | Recommendation |
|---|---|---|
| **Title and abstract** | 1 ✓ | (*a*) ✓*page 2* - Indicate the study's design with a commonly used term in the title or the abstract |
| | | (*b*) ✓*page 2* - Provide in the abstract an informative and balanced summary of what was done and what was found |
| **Introduction** | | |
| Background/rationale | 2✓ | ✓*page 4* - Explain the scientific background and rationale for the investigation being reported |
| Objectives | 3✓ | ✓ *page 4* - State specific objectives, including any prespecified hypotheses |
| **Methods** | | |
| Study design | 4✓ | ✓*pages 4 through 6* - Present key elements of study design early in the paper |
| Setting | 5✓ | Describe the setting, locations, and relevant dates, including periods of recruitment, exposure, follow-up, and data collection |
| Participants | 6✓ | ✓*page 5* - (*a*) *Cohort study*—Give the eligibility criteria, and the sources and methods of selection of participants. Describe methods of follow-up<br>*Case-control study*—Give the eligibility criteria, and the sources and methods of case ascertainment and control selection. Give the rationale for the choice of cases and controls<br>*Cross-sectional study*—Give the eligibility criteria, and the sources and methods of selection of participants |
| | | (*b*) *Cohort study*—For matched studies, give matching criteria and number of exposed and unexposed<br>*Case-control study*—For matched studies, give matching criteria and the number of controls per case |
| Variables | 7✓ | ✓*page 5* - Clearly define all outcomes, exposures, predictors, potential confounders, and effect modifiers. Give diagnostic criteria, if applicable |
| Data sources/ measurement | 8✓* | ✓*page 5* - For each variable of interest, give sources of data and details of methods of assessment (measurement). Describe comparability of assessment methods if there is more than one group |
| Bias | 9✓ | ✓*page 6* - Describe any efforts to address potential sources of bias |
| Study size | 10✓ | ✓*page 7 and figure 1*- Explain how the study size was arrived at |
| Quantitative variables | 11✓ | ✓*page 6* - Explain how quantitative variables were handled in the analyses. If applicable, describe which groupings were chosen and why |
| Statistical methods | 12✓ | (*a*) *NA* Describe all statistical methods, including those used to control for confounding |
| | | (*b*) *NA* Describe any methods used to examine subgroups and interactions |
| | | (*c*) ✓*page 6* - Explain how missing data were addressed |
| | | (*d*) ✓*page 6* - *Cohort study*—If applicable, explain how loss to follow-up was addressed<br>*Case-control study*—If applicable, explain how matching of cases and controls was addressed<br>*Cross-sectional study*—If applicable, describe analytical methods taking account of sampling strategy |
| | | (*e*) ✓Describe any sensitivity analyses |

For peer review only

Continued on next page

For peer review only


For peer review only - http://bmjopen.bmj.com/site/about/guidelines.xhtml

| | | |
|---|---|---|
| **Results** | | |
| Participants | 13* | ✓*page 6* - (a) Report numbers of individuals at each stage of study—eg numbers potentially eligible, examined for eligibility, confirmed eligible, included in the study, completing follow-up, and analysed |
| | | ✓*page 6 and figure 1* - (b) Give reasons for non-participation at each stage |
| | | ✓*figure 1* - (c) Consider use of a flow diagram |
| Descriptive data | 14*✓ | (a) ✓*table 1* - Give characteristics of study participants (eg demographic, clinical, social) and information on exposures and potential confounders |
| | | (b) ✓*table 1* - Indicate number of participants with missing data for each variable of interest |
| | | (c) ✓*table 1*- *Cohort study*—Summarise follow-up time (eg, average and total amount) |
| Outcome data | 15* | ✓*table 1, figures 2-3 and pages 7-8 Cohort study*—Report numbers of outcome events or summary measures over time |
| | | *Case-control study*—Report numbers in each exposure category, or summary measures of exposure |
| | | *Cross-sectional study*—Report numbers of outcome events or summary measures |
| Main results | 16 | ✓*table 2, appendix tables* and pages 7-8 - (*a*) Give unadjusted estimates and, if applicable, confounder-adjusted estimates and their precision (eg, 95% confidence interval). Make clear which confounders were adjusted for and why they were included |
| | | *NA* (*b*) Report category boundaries when continuous variables were categorized |
| | | ✓ absolute rates reported in pages 7-8 - (*c*) If relevant, consider translating estimates of relative risk into absolute risk for a meaningful time period |
| Other analyses | 17 | ✓*pages 7-8* - Report other analyses done—eg analyses of subgroups and interactions, and sensitivity analyses |
| **Discussion** | | |
| Key results | 18 | ✓*page 8* Summarise key results with reference to study objectives |
| Limitations | 19 | ✓*pages 9-10* - Discuss limitations of the study, taking into account sources of potential bias or imprecision. Discuss both direction and magnitude of any potential bias |
| Interpretation | 20 | ✓*pages 8-10* Give a cautious overall interpretation of results considering objectives, limitations, multiplicity of analyses, results from similar studies, and other relevant evidence |
| Generalisability | 21 | ✓ *page 9* Discuss the generalisability (external validity) of the study results |
| **Other information** | | |
| Funding | 22 | ✓*page 11* Give the source of funding and the role of the funders for the present study and, if applicable, for the original study on which the present article is based |

*Give information separately for cases and controls in case-control studies and, if applicable, for exposed and unexposed groups in cohort and cross-sectional studies.

**Note:** An Explanation and Elaboration article discusses each checklist item and gives methodological background and published examples of transparent reporting. The STROBE checklist is best used in conjunction with this article (freely available on the Web sites of PLoS Medicine at http://www.plosmedicine.org/, Annals of Internal Medicine at http://www.annals.org/, and Epidemiology at http://www.epidem.com/). Information on the STROBE Initiative is available at www.strobe-statement.org.

For peer review only


For peer review only - http://bmjopen.bmj.com/site/about/guidelines.xhtml

BMJ Open

# An Electronic Health Records cohort study on Heart Failure Following Myocardial Infarction in England: Incidence and Predictors

| | |
|---|---|
| Journal: | *BMJ Open* |
| Manuscript ID | bmjopen-2017-018331.R2 |
| Article Type: | Research |
| Date Submitted by the Author: | 25-Sep-2017 |
| Complete List of Authors: | Gho, Johannes; Universitair Medisch Centrum Utrecht, Cardiology; Farr Institute of Health Informatics Research, University College London, Epidemiology and Public Health<br>Schmidt, Amand; Faculty of Science and Engineering, Groningen Research Institute of Pharmacy; University College London, Institute of Cardiovascular Science<br>Pasea, Laura; Farr Institute of Health Informatics Research, University College London, Epidemiology and Public Health<br>Koudstaal, Stefan; Farr Institute of Health Informatics Research, University College London, Epidemiology and Public Health; University Medical Centre Utrecht, Department of Cardiology<br>Pujades Rodriguez, Mar; Farr Institute of Health Informatics Research, University College London, Epidemiology and Public Health; University of Leeds, Medical Research Council Bioinformatics Centre, Leeds Institute of Biomedical and Clinical Sciences<br>Denaxas, S; Farr Institute of Health Informatics Research, University College London, Epidemiology and Public Health<br>Shah, Anoop; UCL, Farr Institute of Health Informatics research<br>Patel, Riyaz; Farr Institute of Health Informatics Research, University College London, Epidemiology and Public Health<br>Gale, Chris; University of Leeds, Medical Research Council Bioinformatics Centre, Leeds Institute for Cardiovascular and Metabolic Medicine<br>Hoes, Arno; Julius Center for Health Sciences and Primary Care, University Medical Center, Utrecht<br>Cleland, J; Imperial College London, National Heart & Lung Institute, Royal Brompton & Harefield Hospitals<br>Hemingway, Harry; Farr Institute of Health Informatics Research, University College London, Epidemiology and Public Health<br>Asselbergs, Folkert; University Medical Centre Utrecht, Department of Cardiology; ICIN-Netherlands Heart Institute, Durrer Center for Cardiogenetic Research |
| <b>Primary Subject Heading</b>: | General practice / Family practice |
| Secondary Subject Heading: | Cardiovascular medicine, Epidemiology, General practice / Family practice, Health informatics, Public health |
| Keywords: | Heart failure < CARDIOLOGY, Myocardial infarction < CARDIOLOGY, electronic health records |

For peer review only

# An Electronic Health Records cohort study on Heart Failure Following Myocardial Infarction in England: Incidence and Predictors

Johannes M.I.H. Gho, MD, PhD,[1,2,*] Amand F. Schmidt, PhD,[2,3,4*] Laura Pasea, MSc,[1] Stefan Koudstaal, MD, MSc, PhD,[1,2] Mar Pujades-Rodriguez, PhD,[1,5] Spiros Denaxas, PhD,[1] Anoop D. Shah, MRCP,[1] Riyaz S. Patel, MD, PhD,[1,3] Chris P. Gale, PhD, FRCP,[6] Arno W. Hoes, MD, PhD,[7] John G. Cleland, MD, FRCP,[8] Harry Hemingway, FFPH, FRCP,[1] Folkert W. Asselbergs, MD, PhD,[1,2,3,9]

[1]Farr Institute of Health Informatics Research, UCL Institute of Health Informatics, University College London, London, UK
[2]Department of Cardiology, Division Heart and Lungs, University Medical Center Utrecht, Utrecht, the Netherlands
[3]Institute of Cardiovascular Science, Faculty of Population Health Sciences, University College London, London, UK
[4]Groningen Research Institute of Pharmacy, University of Groningen, Groningen, the Netherlands
[5]Medical Research Council Bioinformatics Centre, Leeds Institute of Biomedical and Clinical Sciences, University of Leeds, Leeds, UK
[6]Medical Research Council Bioinformatics Centre, Leeds Institute for Cardiovascular and Metabolic Medicine, University of Leeds, Leeds, UK
[7]Julius Center for Health Sciences and Primary Care, University Medical Center Utrecht and Utrecht University, Utrecht, the Netherlands
[8]National Heart & Lung Institute, Royal Brompton & Harefield Hospitals, Imperial College London, London, UK
[9]Durrer Center for Cardiogenetic Research, ICIN-Netherlands Heart Institute, Utrecht, the Netherlands
* Shared first author

**Corresponding author:**
A Floriaan Schmidt
Institute of Cardiovascular Science, University College London
222 Euston Road, London NW1 2DA, United Kingdom
E-mail: amand.schmidt@ucl.ac.uk; a.f.schmidt@rug.nl
Tel: 0044 (0)20 3549 5625

**Word count text**: 3427
**Word count abstract**: 265
**Number of references**: 32
**Number of tables**: 2
**Number of figures**: 3
**(Web)appendix**: 1

For peer review only

**Abstract**

**Objectives** To investigate the incidence and determinants of heart failure (HF) following a myocardial infarction (MI) in a contemporary cohort of MI patients using routinely collected primary and hospital care electronic health records.

**Methods** Data were used from the CALIBER programme, linking electronic health records in England from primary care, hospital admissions, an MI registry and mortality data. Subjects were eligible if they were 18 years or older, did not have a history of HF, and survived a first MI. Factors associated with time to HF were examined using Cox proportional hazard models.

**Results** Of the 24,479 MI patients 5,775 (23.6%) developed HF during a median follow-up of 3.7 years (incidence rate per 1000 person-years 63.8 (95% CI: 62.2 – 65.5). Baseline characteristics significantly associated with developing HF were: atrial fibrillation [HR 1.62, 95% CI 1.51 – 1.75], age [per 10 years increase: 1.45, 1.41 – 1.49], diabetes [1.45, 1.35 – 1.56], peripheral arterial disease [1.38, 1.26 – 1.51], COPD [1.28, 1.17 – 1.40], greater socioeconomic deprivation [5$^{th}$ vs. 1$^{st}$ quintile 1.27, 1.13 – 1.41], ST-segment elevation MI at presentation [1.19, 1.11 – 1.27], and hypertension [1.16, 1.09 – 1.23]. Results were robust to various sensitivity analyses such as competing risk analysis and multiple imputation.

**Conclusion** In England, one in four survivors of a first MI develop HF within four years. This contemporary study demonstrates MI patients are at considerable, risk of HF. Baseline patient characteristics associated with time till HF were identified, which may fruitfully be used to target preventive strategies.


**Keywords:** heart failure; myocardial infarction; incidence; risk factors; electronic health records



For peer review only - http://bmjopen.bmj.com/site/about/guidelines.xhtml

**Strengths and limitations of this study**

- ❖ The current study provides a contemporary description of the burden of heart failure in a representative sample of first time myocardial infarction patient included in the CALIBER, an UK based electronic health care database. Contrary to previous electronic health care studies, who exclusively focussed on hospital records, the current study includes both primary (General Practitioner, GP) and hospital electronic health records increasing generalizability.
- ❖ Stratified methods were used to account for potential calendar and centre effects, and competing risk model were used addressing potential competing effects on both heart failure and mortality.
- ❖ While most patient characteristics (e.g., disease history and drug prescriptions) were completely recorded, information regarding baseline BMI, smoking and blood pressure was missing for up to 70% of patients. Analyses based on multiple imputed data were similar to those from complete case analyses. Despite these reassuring findings, results for variables with a high number of missing observations should be carefully interpreted.
- ❖ Further potential limitations include the reliance on ICD and Read codes for disease classification. While we linked data from three sources (Myocardial Ischaemia National Audit Project, Hospital Episode Statistics, and Clinical Practice Research Datalink), some diseased subjects may have been misclassified as disease free if their diagnoses were not recorded in these databases. Provided that electronic registration is mandatory before patients can dispense prescriptions, misclassification of drug exposures was likely minimal.
- ❖ A small proportion of subjects may have had an earlier myocardial infarction or heart failure event, which was not recorded in our dataset due to left truncation of follow-up time and/or misclassification.

**Abbreviations list:** heart failure (HF); myocardial infarction (MI); ST-segment elevation MI (STEMI); hazard ratio (HR); confidence interval (CI); chronic obstructive pulmonary disease (COPD); body mass index (BMI).



For peer review only - http://bmjopen.bmj.com/site/about/guidelines.xhtml

## Introduction

Research describing the incidence of heart failure (HF) following myocardial infarction (MI) is limited, mainly originating from the thrombolytic era, often using small sample sizes with contradictory findings, making it difficult to provide evidence based medicine. For example, a previous UK study among almost 900 patients hospitalised with MI in 1998, found that one fifth developed HF during their hospital stay and a further third following hospital discharge[1]. More recently, a Swedish study found a 5-year cumulative risk of HF after MI of 21.8% in the calendar period 2004-2013[2]. Further, a Danish nationwide cohort study reported an incidence of HF at 90-days following MI of 19.6% in 2009-10[3]. Differences between these studies could be related to a number of factors, for example change in treatment, national policies, or definitions of heart failure, all of which potentially limit the generalizability of results. We used a large contemporary and representative sample of patients with MI based on electronic health records (EHR) to 1) describe the current incidence of HF after MI in England, using both primary and hospital care data, and 2) explore patient characteristics predictive of post MI HF.

## Methods

Study design

Data were used from the CALIBER dataset, which included linked data from (i) primary care electronic health records, with diagnoses coded using the Read terminology (Clinical Practice Research Datalink, CPRD) from 226 consenting general practices[4], (ii) hospital care administrative records (Hospital Episode Statistics, HES) and disease registry/audit records (Myocardial Ischaemia National Audit Project, MINAP), and (iii) the mortality register (from UK Office for National Statistics, ONS) [5].

Setting and participants

Patients were eligible if they were aged ≥18 years, registered in CPRD practices in England consenting to data linkage, with at least one year of up-to-standard pre-study follow-up (meeting CPRD practice quality standards), and experiencing a first MI between 1$^{st}$ January 1998 and 25$^{th}$ March 2010. The first record of



For peer review only - http://bmjopen.bmj.com/site/about/guidelines.xhtml

MI across the linked data sources was considered as the index event and subsequent MI records within 30 days from the same or other sources were considered as representing the same event [6]. Patients with a fatal index MI or a history of HF before their index MI were excluded. Patients were censored if they ceased to be registered at a participating general practice, at the date of death or the administrative censoring of the dataset (25th March 2010).

Variables and data sources

Study variables were derived from diagnoses recorded across several controlled clinical terminology and statistical classification systems: Read, ICD-9 or ICD-10, medication prescription information or the Office of Population Censuses and Surveys Classification of Interventions and Procedures (OPCS-4) codes (see https://rdrr.io/rforge/CALIBERcodelists/man/PRODDICT.html) with the electronic health record phenotyping algorithms published at https://caliberresearch.org/portal [5].

For continuous variables, the most recent measurement recorded in CPRD in the year before study entry was used as the baseline value. Data before study entry were used to determine the prognostic potential of age (years, using the date of birth), sex, ethnicity, social deprivation (Index of Multiple Deprivation, IMD score as recorded in ONS in quintiles), smoking, alcohol use, history of cardiovascular disease, previous coronary revascularization, history of diabetes, history of thyroid disease, history of chronic obstructive pulmonary disease (COPD), history of depression and history of non-metastatic cancer. (see the CALIBER portal https://caliberresearch.org/portal for details). Diabetes and hypertension diagnosis were based on Read codes from primary care or ICD-10 codes from Hospital Episode Statistics, both of which were classified using primary care consultation records and hospitals diagnosis records. Socioeconomic status was based on the Index of Multiple Deprivation which includes seven domains of deprivation (income deprivation, employment deprivation, health deprivation and disability, education, skills and training deprivation, barriers to housing and services, living environment deprivation and crime) [7]. The index MI event was characterized using ECG findings (e.g. ST-segment-elevation myocardial infarction (STEMI), or non-STEMI (NSTEMI)), site of infarction, mode and timing of reperfusion and peak cardiac biomarkers (troponin I, troponin T and CK). MI was defined by linking all of the CALIBER data sources



For peer review only - http://bmjopen.bmj.com/site/about/guidelines.xhtml

(e.g., Hospital Episode Statistics and CPRD), with the type of MI recorded in MINAP and CPRD, see for more detail https://www.caliberresearch.org and the appendix. Characteristics regarding the index MI (e.g., type of revascularisation and delay from symptom onset to delivery of reperfusion therapy) were derived from MINAP. This nationwide registry is part of an annual audit which records over 20 key MI variables [8]. Herrett and colleagues [6] validated the current approach and showed the necessity of linking multiple sources to ascertain MI (see appendix for codes used). The primary study outcome was the first HF event following MI, which similar to MI, was recorded in multiple CALIBER data sources; echocardiographic findings and NYHA class were unavailable. All phenotypes were created and validated using a robust methodology described elsewhere [9] and have been used in previously published studies[10,11].

For peer review only

Statistical methods

Incidence rates (cases per 1000 person-years) and Kaplan-Meier cumulative incidence rates of HF were estimated, with Kaplan-Meier curves stratified by age (<50, 50-65, ≥65 years) and type of MI. Association between baseline variables with the onset of HF following MI were explored using Cox proportional hazard models. The proportional hazards assumption was checked using Schoenfeld residuals, and by nonparametric correlation coefficients between survival time and the parameter specific residuals. All models were stratified on general practice and calendar year periods of enrolment (1998-2001, 2001-2004, 2004-2007 and 2007-2010). Stratified models were used instead of frailty models because the former does not make any distributional assumption. Models were sequentially adjusted for: 1) age and sex, 2) cardiovascular risk factors, 3) type of MI, and 4) co-morbidities and prescribed medication. Associations are presented as hazard ratios (HRs) with 95% confidence intervals (CI). Analyses were performed using R (version 3.3.2)[12].

Sensitivity analyses

Due to the availability of EHR from multiple sources, clinical diagnoses and prescriptions were completely recorded. Biomarker and lifestyle measurements such as smoking, BMI, and blood pressure were however, incompletely recorded (see Table 1). It is probable that these data were preferentially recorded



For peer review only - http://bmjopen.bmj.com/site/about/guidelines.xhtml

in subjects perceived to be at a higher risk for early progression of CVD. Pairwise analyses regressing a complete case indicator on observed variables indeed showed considerable dependency between recorded data and missingness (results not shown), violating the “missing completely at random” assumption. This dependency was utilized by multiply imputing missing data using the mice package, which was implemented using 20 imputed datasets and pooled based on Rubin’s rules (Supplementary)[13].

To account for the fact that patients may have died before the onset of HF (e.g., competing risk by mortality), analyses were repeated using Fine and Gray models[14,15]. As a hypothetical example of competing risk, let us assume that out of 100 MI subjects, one develops HF and 99 die. In a Cox’s regression analysis all 99 dead subjects would be censored (implicitly, and incorrectly, assuming they may develop HF later in time), and the hazard of HF would equal 1/1. Instead a Fine and Gray model recognizes the 99 died subjects could never develop HF and hence calculates the hazard as 1/100.

In addition to these sensitivity analyses, we performed a cancer- and revascularization-stratified analysis. Due to discrepancies in sample size between the subgroups we decided against formal interaction testing, which may suffer from inflated false positive rates and lower power in such settings[16].

Ethical approval

CALIBER has received ethics approval (Supplementary). This study is in compliance with the Declaration of Helsinki and was approved by the ISAC (Independent Scientific Advisory Committee) for MHRA Database Research (protocol no. 14_198R) and the MINAP Academic Group and is registered with ClinicalTrials.gov (NCT02384213).

## Results

Subjects and baseline characteristics

For peer review only

In total there were 52,770 patients with an index MI during the study period (Figure 1). Excluding patients with a fatal index MI (n = 15,104), a prior history of HF (n = 3,876), missing information on the type of MI (n = 97) and subjects with less than 1 year of follow-up prior to the indexing MI event (n = 9,214), resulted in an analytical cohort of 24,479 patients. Patients were recruited from 226 GP practices, with the median practice enrolling 101 patients (quartile (Q1) 65 and Q3 150). Of the included subjects 4,657 were STEMI and 19,822 NSTEMI patients (0% missing), 15,969 (65.2%) were men (0% missing), 4,538 (42.1%) currently smoked (56% missing), 12,258 (50.1%) had hypertension (0% missing), and 3,014 (12.3%) had a history of diabetes at baseline (0% missing). In total, 6,129 (25.0%) were prescribed beta-blockers (0% missing) and 5,039 (20.6%) angiotensin converting enzyme (ACE) inhibitors (0% missing) prior to their indexing MI (Table 1). The number of patients identified across the previously defined calendar periods was reasonably stable, with a minimum of 5219 subjects in the period 1998-2001 compared to a maximum of 6838 subjects identified in the period 2004-2007.

Incidence

Patients contributed 90,482 person years of follow-up, during a median follow-up time of 3.7 years (IQR: 1.5; 6.7), 5,775 patients (23.6%) developed HF. The crude incidence rate of HF following a first MI was 63.8 (95% CI: 62.2 – 65.5) per 1000 person-years. Within the first 30 days post MI follow-up 2,438 (10.0%) patients developed HF (Figure 2). From day 30 onwards 3,337 (15.8%) of MI patients (event free during the first 30 days) developed HF, with 6.8% experiencing an HF event within the first year (Figure 2). The incidence of HF during the first 30-days of follow-up was 4.3% (102) in patients younger than 50 years, 6.0% (459) in 50-65 year olds and 12.9% (1877) in those 65 years and older, with HF incidence increasing proportionally as time progressed towards 10 years (Figure 3 top row). The 30-day incidence of HF was 9.5% (1892 events) for NSTEMI subjects and 11.7% (546 events) for STEMI subjects. Excluding patients who experienced a HF within the first 30 days showed that STEMI patients had a lower incidence of HF than NSTEMI subjects (Figure 3 lower panels). At 57 days the crude cumulative risk of HF in the NSTEMI subjects surpassed that of the STEMI subjects for the first time (0.0151 versus 0.0147), with the curves further diverging at 73 days of follow-up (since indexing MI).



For peer review only - http://bmjopen.bmj.com/site/about/guidelines.xhtml

Over the entire follow-up 5,921 subjects died, of which 3,538 where free of HF at the time. During the first 30 days of follow-up only 1 patient died, limiting the potential for competing risk by all-cause mortality. In patients who did not have HF after 30 days accounting for competing risk by all-cause mortality attenuated the cumulative risk to be about 15% after 10 years of follow-up (Appendix Figure). Furthermore, the cumulative risks of all-cause mortality and HF converged as follow-up time progressed towards 10 years.

Predictors of HF after multivariable adjustments

Table 2 describes the results of the previously described nested Cox's models, showing little change in estimates between the increasingly complex models. Focusing on the final model, the hazard of HF increased with atrial fibrillation [HR 1.62, 95%CI 1.51 – 1.75], age [per 10 years increase: 1.45, 1.41 – 1.49], diabetes [1.45, 1.35 – 1.56], peripheral arterial disease [1.38, 1.26 – 1.51], COPD [1.28, 1.17 – 1.40], hypertension [1.16, 1.09 – 1.23], higher socioeconomic deprivation (p-value for the 5 groups < 0.001), and STEMI at presentation [1.19, 1.11 – 1.27)]. Accounting for competing risk by all-cause mortality had little impact on the presented results (Appendix Table S1). After multiply imputing the data models were extended to include smoking, BMI, and systolic and diastolic blood pressure variables which showed BMI, male sex and smoking to be conditionally independent prognostic factors (Supplementary Table S2). The extended model 5 was implemented using imputed data, and non-imputed (complete case) data, resulting in similar HR estimates (magnitude and direction). Stratifying the sample on cancer diagnosis or history of revascularization showed broadly similar results between subgroups (See appendix table S3 and S4, focusing on the confidence intervals), however precision was low due to the limited number of patients with an history of revascularization or cancer.

## Discussion

In this large population-based study using linked electronic health records, 23.6% of patients who survived a first MI developed HF during a median follow-up of 3.7 years, resulting in an incidence rate of 64 cases per 1000 person-years. Incident HF was associated with increasing age, higher socioeconomic

For peer review only

deprivation, a history of diabetes, atrial fibrillation, peripheral arterial disease, COPD, STEMI at presentation, BMI and smoking.

A previous Canadian study, using data from the period 1994-2000[17], found that 71% of elderly patients without HF at index admission developed HF within 5 years' time after an MI, whereas the mortality due to MI decreased in the same period. The Framingham Heart Study[18], using data from 1990 to 1999, found a 5-years post-MI HF incidence of 31.9% after MI; lower than found in the Canadian study. Importantly, both studies showed a higher incidence of post-MI HF than our more contemporary English cohort. This lower HF incidence is likely due to continued improvements of MI treatment strategies, which are reflected in a decreased HF incidence over calendar time. For example, a 20,812 sample of MI patients hospitalized in Western Australia showed that the overall 1-year incidence of HF after MI decreased from 28.1% 1996 in to 16.5% in 2007[19]. This decline is confirmed further by a national Swedish hospital discharge and death registry study reporting a one third decline in incidence between 1993-2004[20].The same Swedish group recently reported that this trend persists for the period 2004-2013[2] and showed improved pharmacological treatment and early revascularisation in this period. During a median follow-up of 4 years, 19% of the patients were re-hospitalized because of HF. An explanation for the higher percentage in our study is probably the inclusion of HF diagnosis made in the GP setting next to hospital records decreasing misclassification of HF events. Future efforts are needed to harmonize these national data sources to compare daily care and HF epidemiology across different countries.

Prognostic factors

Similar to the current study, Torabi et al[21] reported that HF after MI increased steeply with age. Socioeconomic deprivation has been shown to be an independent predictor of HF development and associated with an increased incidence of HF in MI-free subjects [22,23]. Socioeconomic deprivation has also been associated with more frequent hospital admission and higher mortality in HF patients[24–26]. The current study extends these observations to HF incidence after an MI. Furthermore, we showed that a history of diabetes, atrial fibrillation, peripheral arterial disease, COPD, STEMI at presentation, BMI and smoking are all independently prognostic of HF after MI. Interestingly, despite the size of the collected

For peer review only



For peer review only - http://bmjopen.bmj.com/site/about/guidelines.xhtml

sample, sex only became significantly associated with HF after accounting for differences in BMI and smoking. Furthermore, the impact of male sex (HR of 1.07 95%CI 1.01 – 1.13) was modest indicating relative sex equality. It is of note that blood pressure was not significantly associated with HF. However, blood pressure measurements were frequently missing, which (even after multiple imputation) may be the cause of the observed lack of association hence this deserves independent exploration. In this light it is important to note that the diagnosis of hypertension was associated with HF. Potentially this discrepancies between the association of blood pressure measurement and hypertension diagnosis can be explained by noting that a recorded diagnosis is indicative of long-term hypertensions, which may be different than a single blood pressure measurement in terms of prognosis. Further, given that both variables were included in the same model, the observed difference in association may suggest that conditional on hypertension, blood pressure itself is only modestly associated to HF, if at all.

Strengths and limitations

The linkage of multiple electronic health record sources from primary and hospital care allowed for the collection of a representative sample[27], which enabled us to explore the prognostic value of routinely collected data in primary care records and to detect non-hospitalised HF cases. The population of CPRD practices has been shown broadly representative of the UK population[4,28]. In total 226 GP practices consented to data linkage with Hospital Episode Statistics, MINAP, and the Office for National Statistics (containing 3.9% of the population of England in 2006). A potential limitation is that the ascertainment of cardiovascular outcomes was not based on clinical criteria (e.g., validated questionnaires and properly conducted physical examinations), practices of medical coding will have changed over time and there could be subgroups of patients with left ventricular dysfunction without clinical symptoms. Calendar dependent changes over time were accounted for by using time-period stratified Cox models. We further wish to highlight the infrequent use of PCI in STEMI patients, which was shown to be representative of the slower uptake in England[29]. Similarly, 32% of the patients used antiplatelet drugs at baseline which may be a reflection of non-MI CVD burden. The Kaplan-Meier plots for subtype of MI indicate a possible violation of the proportional hazard assumption for this variable. However, these plots represent the pairwise associations between MI subtype and time to HF, and as such assume that MI subtype is

For peer review only



For peer review only - http://bmjopen.bmj.com/site/about/guidelines.xhtml

independent of other prognostic factors, which is known to be false. The importance of conditioning on covariates is underlined by noting the Kaplan-Meier plots indicating a protective effect of STEMI versus NSTEMI, which was significantly reversed after correcting for covariates (models 3-4). Similarly, instead of using Kaplan-Meier plots to assess the proportional hazard assumption of the *crude* associations, we used Schoenfeld residuals to explore this assumption for the *conditional* associations (multivariable model 4); which did not show any violations. Based on model 4 the absolute correlation between the Schoenfeld residuals and time was < 0.10 (e.g., for MI subtype this was 0.06) indicating an absence of relevant interaction by time (e.g., non-proportionality of hazard). Residual confounding due to medication use (or other missing/mis-specified variables) might be another potential source of bias, however our intention was not to perform a causal analysis between drug prescriptions and time to HF. As such it is interesting to note that despite the large sample size we did not observe a significant association of ACE, ARB or beta-blocker prescribed prior to the indexing MI event and time to HF. We adhered to CPRD recommendations to obtain up-to-standard baseline data by excluding patients with less than 1 year at CPRD practice prior to index MI. Previously Lewis and colleagues showed that 3 months after registration with a new practice most patient characteristics were updated correctly, which approximated 100% after 1 year of follow-up[30]. Assuming that duration of the CPRD follow-up is independent of the relations assessed here, excluding such patients should not hamper generalizability of results. Using the first MI recorded in the database without a prior history of HF, might have introduced bias due to left truncation (e.g., some subjects may already have experienced an MI before enrolment)[31]. However CALIBER holds longitudinal records from primary and hospital care, making it unlikely a large part of the patients were misclassified as MI free and the one year of follow-up prior to entry further decreases the likelihood of misclassifying patients[30]. While the current data is adequate to identify subjects with a first MI, the sub-classification of MI patients into STEMI and NSTEMI, despite recent improvements, is known to be error prone [32]. As such, results for MI type need replication using higher quality data, in perhaps purposely designed studies. Due to our interest in HF occurrence after a first MI selection bias (e.g., index event bias) was introduced[33]. This index event bias does not impact the descriptive or prognostic value of the association presented, and is mostly relevant if one wants to develop an intervention based on the associations presented; which was not the aim of this study. Additionally, we note that we reduced the

For peer review only

influence of selection bias by accounting for dependencies between predictors. An important caveat of electronic health care records is that these data are predominantly focussed on recording diagnoses and prospections but not on their complement (i.e., who is **not** diseased or who **did** not receive a drug). As such we have assumed that subjects without a recorded drug prescription or diagnoses was unexposed or free of (that specific) disease. Provided that electronic registration is required for a patient to fill a prescription we can be fairly confident that we did not miss many prescribed treatments. However, it is likely that some subjects were misclassified as free of disease while in fact they were not. We've attempted to minimize this misclassification by linking data across multiple healthcare settings and data sources (MINAP, HES and CPRD, ONS).

A final limitation is that there is a possible delay between primary and hospital care records. Previous research has shown that MI events tend to be recorded in primary care after the HES or MINAP record [6]. The lower 30-day HF incidence in unclassified MI patients primarily derived from primary care could be partly explained because of a delay in coding. Therefore, we showed cumulative incidence rates in patients (alive and HF event free within the first 30 days) from 30 days after index MI to account for a delay in recording of MI in primary care. We were unable to differentiate between HF with preserved ejection fraction (HFpEF) and HF with reduced ejection fraction (HFrEF) as we had no access to detailed (echocardiographic) parameters to assess diastolic dysfunction. It is likely, however that the majority of our HF patients had developed systolic dysfunction after MI.

Conclusion

In this large cohort study using linked electronic health records in England from primary and hospital care, about one in four people developed HF within a median of four years after surviving a first MI. Increasing age, higher socioeconomic deprivation, a history of hypertension, diabetes, atrial fibrillation, peripheral arterial disease, COPD, smoking and STEMI at presentation were independent determinants new onset HF following MI.

**Authors' contributions**



For peer review only - http://bmjopen.bmj.com/site/about/guidelines.xhtml

JMIHG, AFS, LP, SK, MPR, SD, ADPS, RSP, CPG, AWH, HH, FWA contributed to the idea and design of the study. JMIHG extracted and prepared the data for analysis. AFS and JMIHG performed the analysis. JMIHG drafted the manuscript, with revisions AFS, LP, SK, MPR, SD, ADPS, RSP, CPG, AWH, HH, FWA. JMIHG guaranties the quality and accuracy of the results presented.

**Competing interests**

We have read and understood BMJ policy on declaration of interests and declare that we have no competing interests

**Data Sharing Statement**

Access to raw data can be requested from the CPRD http://cprd.com

**Funding**

This study was supported by the National Institute for Health Research (RP-PG-0407–10314), Wellcome Trust (086091/Z/08/Z), the Medical Research Council Prognosis Research Strategy Partnership (G0902393/99558) and the Farr Institute of Health Informatics Research, funded by The Medical Research Council (MR/K006584/1), in partnership with Arthritis Research UK, the British Heart Foundation, Cancer Research UK, the Economic and Social Research Council, the Engineering and Physical Sciences Research Council, the National Institute of Health Research, the National Institute for Social Care and Health Research (Welsh Assembly Government), the Chief Scientist Office (Scottish Government Health Directorates) and the Wellcome Trust. Part of this work is funded through the Innovative Medicines Initiative 2 Joint Undertaking under grant agreement No 116074, BigData@Heart. AFS is funded by UCLH NIHR Biomedical Research Centre and is a UCL Springboard Population Health Sciences Fellow. HH is an NIHR Senior Investigator, and receives support from the NIHR University College Hospitals / University College London Biomedical Research Centre. FWA is supported by a Dekker scholarship-Junior Staff Member 2014T001 – Netherlands Heart Foundation and UCL Hospitals NIHR Biomedical Research Centre.

For peer review only



For peer review only - http://bmjopen.bmj.com/site/about/guidelines.xhtml

## References


1 Torabi A, Cleland JGF, Khan NK, *et al.* The timing of development and subsequent clinical course of heart failure after a myocardial infarction. *Eur Heart J* 2008;**29**:859–70.

2 Desta L, Jernberg T, Spaak J, *et al.* Risk and predictors of readmission for heart failure following a myocardial infarction between 2004 and 2013: A Swedish nationwide observational study. *Int J Cardiol* Published Online First: May 2017.http://linkinghub.elsevier.com/retrieve/pii/S0167527316348677 (accessed 22 Aug 2017).

3 Gjesing A, Gislason GH, Kober L, *et al.* Nationwide trends in development of heart failure and mortality after first-time myocardial infarction 1997-2010: A Danish cohort study. *Eur J Intern Med* 2014;**25**:731–8.

4 Herrett E, Gallagher AM, Bhaskaran K, *et al.* Data Resource Profile: Clinical Practice Research Datalink (CPRD). *Int J Epidemiol* 2015;**44**:827–36.

5 Denaxas SC, George J, Herrett E, *et al.* Data resource profile: Cardiovascular disease research using linked bespoke studies and electronic health records (CALIBER). *Int J Epidemiol* 2012;**41**:1625–38.

6 Herrett E, Shah AD, Boggon R, *et al.* Completeness and diagnostic validity of recording acute myocardial infarction events in primary care, hospital care, disease registry, and national mortality records: cohort study. *BMJ* 2013;**346**:f2350.

7 DCLG. The English Indices of Deprivation 2010. *Neighb Stat Release* 2011;:1–20.http://www.communities.gov.uk/publications/corporate/statistics/indices2010technicalreport

8 Herrett E, Smeeth L, Walker L, *et al.* The Myocardial Ischaemia National Audit Project (MINAP). *Heart* 2010;**96**:1264–7.http://www.ncbi.nlm.nih.gov/pubmed/23177996

9 Morley KI, Wallace J, Denaxas SC, *et al.* Defining Disease Phenotypes Using National Linked Electronic Health Records: A Case Study of Atrial Fibrillation. *PLoS One* 2014;**9**:e110900.

10 Rapsomaniki E, Timmis A, George J, *et al.* Blood pressure and incidence of twelve cardiovascular diseases: Lifetime risks, healthy life-years lost, and age-specific associations in 1·25 million people. *Lancet* 2014;**383**:1899–911. doi:10.1016/S0140-6736(14)60685-1

11 Shah AD, Langenberg C, Rapsomaniki E, *et al.* Type 2 diabetes and incidence of cardiovascular


For peer review only



For peer review only - http://bmjopen.bmj.com/site/about/guidelines.xhtml

diseases: a cohort study in 1·9 million people. *Lancet Diabetes Endocrinol* 2015;**3**:105–13. doi:10.1016/S2213-8587(14)70219-0

12 R Core Team. R: A language and environment for statistical computing. 2017.

13 Marshall A, Altman DG, Holder RL, *et al.* Combining estimates of interest in prognostic modelling studies after multiple imputation: current practice and guidelines. BMC Med. Res. Methodol. 2009;**9**:57-.

14 Satagopan JM, Ben-Porat L, Berwick M, *et al.* A note on competing risks in survival data analysis. *Br J Cancer* 2004;**91**:1229–35.http://www.nature.com/doifinder/10.1038/sj.bjc.6602102

15 Gray B. cmprsk: Subdistribution Analysis of Competing Risks. 2014.http://cran.r-project.org/package=cmprsk

16 Schmidt AF, Groenwold RHH, Knol MJ, *et al.* Exploring interaction effects in small samples increases rates of false-positive and false-negative findings: Results from a systematic review and simulation study. *J Clin Epidemiol* Published Online First: 2014. doi:10.1016/j.jclinepi.2014.02.008

17 Ezekowitz JA, Kaul P, Bakal JA, *et al.* Declining in-hospital mortality and increasing heart failure incidence in elderly patients with first myocardial infarction. *J Am Coll Cardiol* 2009;**53**:13–20.

18 Velagaleti RS, Pencina MJ, Murabito JM, *et al.* Long-term trends in the incidence of heart failure after myocardial infarction. *Circulation* 2008;**118**:2057–62.

19 Hung J, Teng T-HK, Finn J, *et al.* Trends from 1996 to 2007 in incidence and mortality outcomes of heart failure after acute myocardial infarction: a population-based study of 20,812 patients with first acute myocardial infarction in Western Australia. *J Am Heart Assoc* 2013;**2**:e000172.

20 Shafazand M, Rosengren A, Lappas G, *et al.* Decreasing trends in the incidence of heart failure after acute myocardial infarction from 1993-2004: a study of 175,216 patients with a first acute myocardial infarction in Sweden. *Eur J Heart Fail* 2011;**13**:135–41.

21 Torabi A, Cleland JG, Rigby AS, *et al.* Development and course of heart failure after a myocardial infarction in younger and older people. *J Geriatr Cardiol* 2014;**11**:1–12.http://www.pubmedcentral.nih.gov/articlerender.fcgi?artid=3981977&tool=pmcentrez&rendertype=abstract

22 McAlister FA, Murphy NF, Simpson CR, *et al.* Influence of socioeconomic deprivation on the

For peer review only



For peer review only - http://bmjopen.bmj.com/site/about/guidelines.xhtml

primary care burden and treatment of patients with a diagnosis of heart failure in general practice in Scotland: population based study. *BMJ* 2004;**328**:1110.

23 Hawkins NM, Jhund PS, McMurray JJ V, *et al.* Heart failure and socioeconomic status: Accumulating evidence of inequality. *Eur J Heart Fail* 2012;**14**:138–46.

24 Philbin EF, Dec GW, Jenkins PL, *et al.* Socioeconomic status as an independent risk factor for hospital readmission for heart failure. *Am J Cardiol* 2001;**87**:1367–71.

25 Struthers AD, Anderson G, Donnan PT, *et al.* Social deprivation increases cardiac hospitalisations in chronic heart failure independent of disease severity and diuretic non-adherence. *Heart* 2000;**83**:12–6.

26 Pujades-Rodriguez M, Timmis A, Stogiannis D, *et al.* Socioeconomic deprivation and the incidence of 12 cardiovascular diseases in 1.9 million women and men: implications for risk prediction and prevention. *PLoS One* 2014;**9**:e104671.

27 Mann RD, Andrews EB. *Pharmacovigilance: Second Edition*. 2007.

28 Walley T, Mantgani A. The UK General Practice Research Database. *Lancet (London, England)* 1997;**350**:1097–9.

29 Chung SC, Gedeborg R, Nicholas O, *et al.* Acute myocardial infarction: A comparison of short-term survival in national outcome registries in Sweden and the UK. *Lancet* 2014;**383**:1305–12.

30 Lewis JD, Bilker WB, Weinstein RB, *et al.* The relationship between time since registration and measured incidence rates in the General Practice Research Database. *Pharmacoepidemiol Drug Saf* 2005;**14**:443–51.

31 Hazelbag CM, Klungel OH, van Staa TP, *et al.* Left truncation results in substantial bias of the relation between time-dependent exposures and adverse events. *Ann Epidemiol* 2015;**25**:590–6.

32 Alexandrescu R, Bottle A, Jarman B, *et al.* Current ICD10 codes are insufficient to clearly distinguish acute myocardial infarction type: a descriptive study. *BMC Health Serv Res* 2013;**13**:468.http://www.ncbi.nlm.nih.gov/pubmed/24195773

33 Dahabreh IJ, Kent DM. Index event bias as an explanation for the paradoxes of recurrence risk research. *Jama* 2011;**305**:822–3.

For peer review only

**Figure titles and legends**

**Figure 1 – Flowchart of study population**

CALIBER=Cardiovascular disease research using Linked Bespoke studies and electronic health Records; CPRD=Clinical Practice Research Datalink; HF=heart failure; MI=myocardial infarction.

**Figure 2 – Incidence of HF after index MI**

Kaplan-Meier curves for crude incidence of HF after MI. **Left panel** 30-day follow-up after index MI. **Right panel** 10 year follow-up (median follow-up time was 3.7 years) in patients who survived the first 30 days and did not develop HF during the first 30 days (30-days event free). HF=heart failure; MI=myocardial infarction. “N at risk” represent the number of subjects at risk at a certain time point. “Cum events” represents the cumulative number of events since the previous time points. A spike histogram is provided (in red) at the top of the graphs providing information on the number of event across time.

**Figure 3 – Incidence of HF after index MI by group**

Kaplan-Meier curves for crude incidence of HF after MI stratified by group. **Top left panel:** 30-day follow-up after index MI stratified by age group. **Top right panel:** 10-year follow-up (median follow-up time was 3.7 years) in 30-day event free patients stratified by age group. Log-rank test of ≥65 year old patients compared to patients aged 50-65 years ($p<0.001$). **Bottom left panel:** 30-day follow-up after index MI stratified by MI type. **Bottom right panel:** 10-year follow-up (median follow-up time was 3.7 years) in patients who survived the first 30 days and did not develop HF during the first 30 days stratified by MI type. Log-rank test of NSTEMI compared to STEMI ($p<0.001$). HF=heart failure; MI=myocardial infarction. N at risk” represent the number of subjects at risk at a certain time point, with the cumulative number of events since the previous time points presented between brackets.

For peer review only

Table 1 - Baseline characteristics at index myocardial infarction

| | STEMI patients n = 4,657 | NSTEMI patients n = 19,822 | Total MI patients n = 24,479 | Unknown (%) |
|---|---|---|---|---|
| Follow-up time (years), median (IQR) | 3.3 (1.4–5.7) | 3.9 (1.5–7.1) | 3.7 (1.5–6.7) | 0% |
| Mean age, years (SD) | 65.7 (13.2) | 68.7 (13.2) | 68.1 (13.2) | 0% |
| Male sex | 3311 (71.1%) | 12658 (63.9%) | 15969 (65.2%) | 0% |
| Ethnicity | | | | |
| White | 3466 (76.4%) | 14288 (76.1%) | 17754 (76.2%) | 4.8% |
| Asian | 83 (1.8%) | 327 (1.7%) | 410 (1.8%) | |
| Black | 20 (0.4%) | 63 (0.3%) | 83 (0.4%) | |
| Other | 968 (21.3%) | 4085 (21.7%) | 5053 (21.7%) | |
| Body mass index (BMI in kg/$m^2$) | | | | |
| Underweight (18.5 <) | 22 (1.7%) | 120 (2.0%) | 142 (1.9%) | 70.0% |
| Normal (18.5; 25) | 335 (25.5%) | 1661 (27.4%) | 1996 (27.2%) | |
| Overweight (25, 30) | 557 (43.0%) | 2431 (40.2%) | 2988 (40.7%) | |
| Obese (>30) | 381 (29.4%) | 1842 (30.4%) | 2223 (30.2%) | |
| Index of multiple deprivation | | | | |
| Most deprived quintile | 892 (19.2%) | 3985 (20.2%) | 4877 (20%) | 0.4% |
| | | | | |
| **Risk factors before index MI** | | | | |
| Current smoker | 1235 (51.1%) | 3303 (39.5%) | 4538 (42.1%) | 55.9% |
| Excess alcohol consumption | 55 (9.0%) | 219 (8.1%) | 274 (8.3%) | 86.4% |
| History of atrial fibrillation | 358 (7.7%) | 2210 (11.1%) | 2568 (10.5%) | 0% |
| History of hypertension | 2117 (45.5%) | 10141 (51.2%) | 12258 (50.1%) | 0% |
| History of peripheral arterial disease | 236 (5.1%) | 1475 (7.4%) | 1711 (7%) | 0% |
| Previous revascularization | | | | |
| PCI | 700 (15.0%) | 2044 (10.3%) | 2744 (11.2%) | 0% |
| CABG | 126 (2.7%) | 843 (4.3%) | 969 (4.0%) | 0% |
| Previous TIA | 154 (3.3%) | 998 (5.0%) | 1152 (4.7%) | 0% |
| Previous stroke | 80 (1.7%) | 410 (2.1%) | 490 (2.0%) | 0% |
| History of diabetes | 538 (11.6%) | 2476 (12.6%) | 3014 (12.3%) | 0% |
| History of thyroid disease | 237 (5.1%) | 1374 (6.9%) | 1661 (6.6%) | 0% |
| History of COPD | 304 (6.5%) | 1634 (8.2%) | 1938 (7.9%) | 0% |
| History of non-metastatic cancer | 478 (10.3%) | 2282 (11.5%) | 2760 (11.3%) | 0% |
| | | | | |
| **Vital signs before admission** median (IQR) | | | | |
| Systolic blood pressure, mmHg | 140 (130–153) | 140 (130–154) | 140 (130–154) | 34.2% |
| Diastolic blood pressure, mmHg | 80 (74–88) | 80 (71–88) | 80 (72–88) | 34.2% |
| | | | | |
| **Biomarkers,** median (IQR) | | | | |
| Troponin I (maximum) | 18.4 (3.6–50.0) | 2.2 (0.3–10.0) | 3.88 (0.62–21.4) | 88.1% |
| Troponin T (maximum) | 1.8 (0.60–4.8) | 0.4 (0.14–1.1) | 0.65 (0.18–2) | 90.6% |
| CK (maximum) | 721 (222–1631) | 219 (97–648) | 331 (123–1068) | 79.6% |
| | | | | |
| **Biomarkers before index MI** mean (SD) | | | | |
| Haemoglobin, g/dL, | 14.1 (1.72) | 13.6 (1.84) | 13.7 (1.83) | 67.3% |
| White blood cell count | 8.0 (2.94) | 7.9 (2.92) | 7.9 (2.92) | 69.3% |
| Neutrophil count | 5.0 (2.12) | 5.0 (2.40) | 5.0 (2.35) | 72.1% |
| Platelets | 270 (85.2) | 266 (88.0) | 267 (87.5) | 69.3% |
| Erythrocyte sedimentation rate | 19.5 (18.8) | 22.2 (21.6) | 21.8 (21.2) | 89.5% |
| Creatinine, μmol/L | 98.5 (39.1) | 102.5 (47.3) | 102 (46.0) | 59.0% |
| eGFR - CKD-EPI | 70.4 (19.9) | 66.2 (19.9) | 67.0 (20.0) | 60.7% |
| Random glucose concentration, mmol/L | 7.09 (3.53) | 7.26 (3.89) | 7.23 (3.82) | 76.7% |
| Total cholesterol | 5.39 (1.45) | 5.24 (1.28) | 5.27 (1.31) | 63.7% |
| LDL cholesterol | 3.24 (1.12) | 3.08 (1.10) | 3.11 (1.11) | 81.4% |
| | | | | |
| **Revascularization characteristics** | | | | |
| Site of infarction | | | | |
| Anterior | 393 (40.2%) | 255 (30.7%) | 648 (35.9%) | 92.6% |
| Primary PCI | 258 (19.3%) | 73 (3.2%) | 331 (9.2%) | 85.3% |
| Prehospital fibrinolysis | 324 (7.0%) | 94 (0.5%) | 418 (1.7%) | 0% |
| Median (IQR) delay from symptom to reperfusion (min) | 150 (97 – 280) | 160 (101 – 296) | 153 (99 – 285) | 37.5% |
| | | | | |

For peer review only



For peer review only - http://bmjopen.bmj.com/site/about/guidelines.xhtml

| | | | | |
|---|---|---|---|---|
| **Prescribed medication before index MI** | | | | |
| Antiplatelet | 867 (18.6%) | 6979 (35.2%) | 7846 (32.1%) | 0% |
| Oral anticoagulant | 67 (1.4%) | 624 (3.1%) | 691 (2.8%) | 0% |
| Statin | 825 (17.7%) | 5297 (26.7%) | 6122 (25.0%) | 0% |
| ACE inhibitor | 676 (14.5%) | 4363 (22.0%) | 5039 (20.6%) | 0% |
| Angiotensin receptor blocker (ARB) | 220 (4.7%) | 1208 (6.1%) | 1428 (5.8%) | 0% |
| Beta-blocker | 766 (16.4%) | 5363 (27.1%) | 6129 (25.0%) | 0% |
| Calcium channel blocker | 804 (17.3%) | 4621 (23.3%) | 5425 (22.2%) | 0% |
| Loop diuretic | 255 (5.5%) | 2188 (11.0%) | 2443 (10.0%) | 0% |
| Aldosterone antagonist | 24 (0.5%) | 198 (1.0%) | 222 (0.9%) | 0% |
| Digoxin | 53 (1.1%) | 515 (2.6%) | 568 (2.3%) | 0% |

IQR=inter-quartile range; SD=standard deviation; MI=myocardial infarction; STEMI=ST-segment-elevation myocardial infarction; PCI=Percutaneous Coronary Intervention; CABG=Coronary Artery Bypass Grafting; TIA=Transient Ischaemic Attack; COPD=Chronic Obstructive Pulmonary Disease; CK=Creatine kinase; ACE=Angiotensin-converting-enzyme. Prehospital and in-hospital fibrinolysis are not mutually exclusive.

For peer review only



For peer review only - http://bmjopen.bmj.com/site/about/guidelines.xhtml

512 **Table 2 - Hazard ratio for heart failure in patients following a first myocardial infarction using multivariable Cox regression**

| | Model 1<br>n = 24,479<br>n events = 5,775 | Model 2<br>n = 24,479<br>n events = 5,775 | Model 3<br>n = 24,479<br>n events = 5,775 | Model 4<br>n = 24,479<br>n events = 5,775 |
|---|---|---|---|---|
| | HR (95% CI) | HR (95% CI) | HR (95% CI) | HR (95% CI) |
| Age, per 10 years | 1.51 (1.48 – 1.55) | 1.49 (1.46 – 1.53) | 1.46 (1.43 – 1.50) | 1.45 (1.41 – 1.49) |
| Men | 1.06 (1.00 – 1.12) | 1.08 (1.02 – 1.14) | 1.07 (1.01 – 1.13) | 1.06 (1.00 – 1.12) |
| Index of multiple deprivation | | overall p = <0.001 | overall p = <0.001 | overall p = <0.001 |
| Q1 (least deprived) | | Reference | Reference | Reference |
| Q2 | | 1.09 (0.99 – 1.20) | 1.09 (0.99 – 1.20) | 1.08 (0.99 – 1.19) |
| Q3 | | 1.20 (1.09 – 1.33) | 1.20 (1.08 – 1.32) | 1.18 (1.07 – 1.31) |
| Q4 | | 1.20 (1.08 – 1.33) | 1.20 (1.08 – 1.33) | 1.17 (1.06 – 1.30) |
| Q5 (most deprived) | | 1.30 (1.16 – 1.45) | 1.30 (1.16 – 1.45) | 1.27 (1.13 – 1.41) |
| History of hypertension | | 1.19 (1.12 – 1.26) | 1.17 (1.10 – 1.24) | 1.16 (1.09 – 1.23) |
| History of diabetes | | 1.48 (1.38 – 1.60) | 1.49 (1.38 – 1.60) | 1.45 (1.35 – 1.56) |
| History of atrial fibrillation | | | 1.65 (1.53 – 1.77) | 1.62 (1.51 – 1.75) |
| Type of MI<br>STEMI | | | 1.17 (1.10 – 1.26) | 1.19 (1.11 – 1.27) |
| History of peripheral arterial disease | | | | 1.38 (1.26 – 1.51) |
| History of COPD | | | | 1.28 (1.17 – 1.40) |
| Prescribed ACE inhibitor before MI | | | | 1.06 (0.99 – 1.13) |
| Prescribed Angiotensin receptor blocker (ARB) before MI | | | | 1.00 (0.89 – 1.11) |
| Prescribed Beta-blocker before MI | | | | 0.93 (0.88 – 1.00) |

513 All analyses presented in table 2 were performed on a complete case dataset of 24,479 subjects. HR=hazard ratio; CI=confidence
514 interval; Q=quintile; MI=myocardial infarction; NSTEMI=non-ST-segment-elevation myocardial infarction; STEMI=ST-segment-
515 elevation myocardial infarction; COPD=chronic obstructive pulmonary disease; ACE=Angiotensin-converting enzyme.

516

For peer review only



For peer review only - http://bmjopen.bmj.com/site/about/guidelines.xhtml

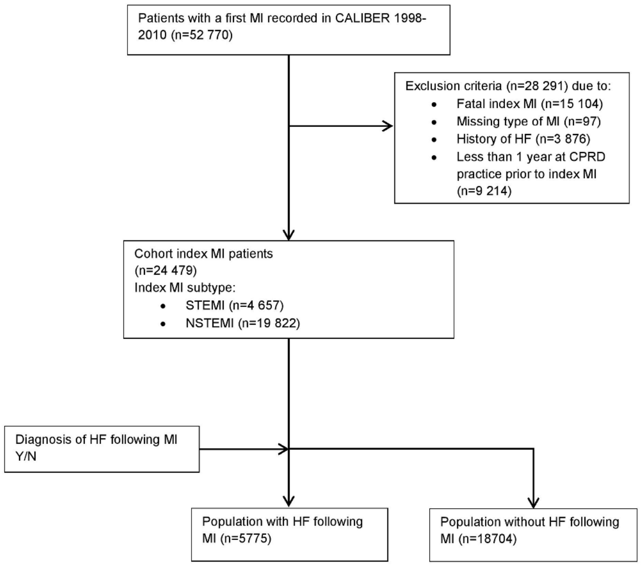


209x297mm (300 x 300 DPI)

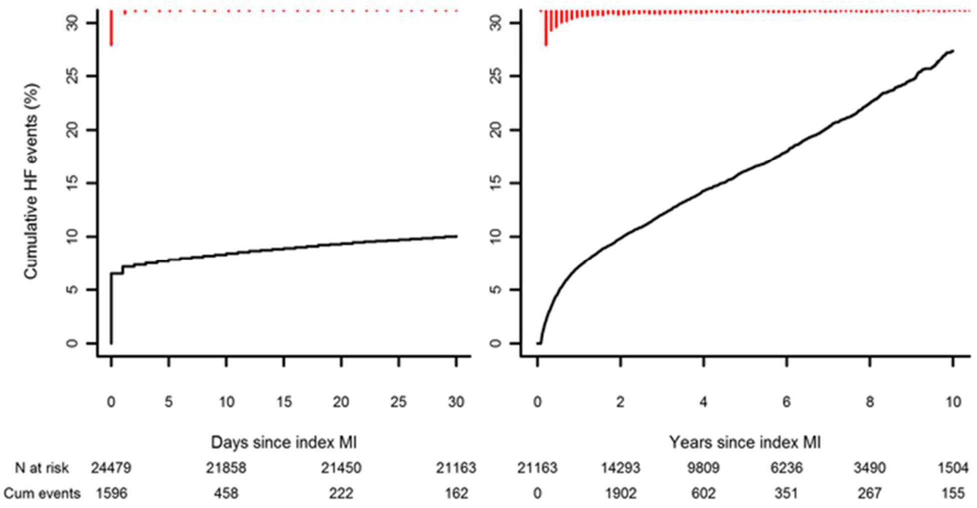


59x35mm (300 x 300 DPI)

For peer review only

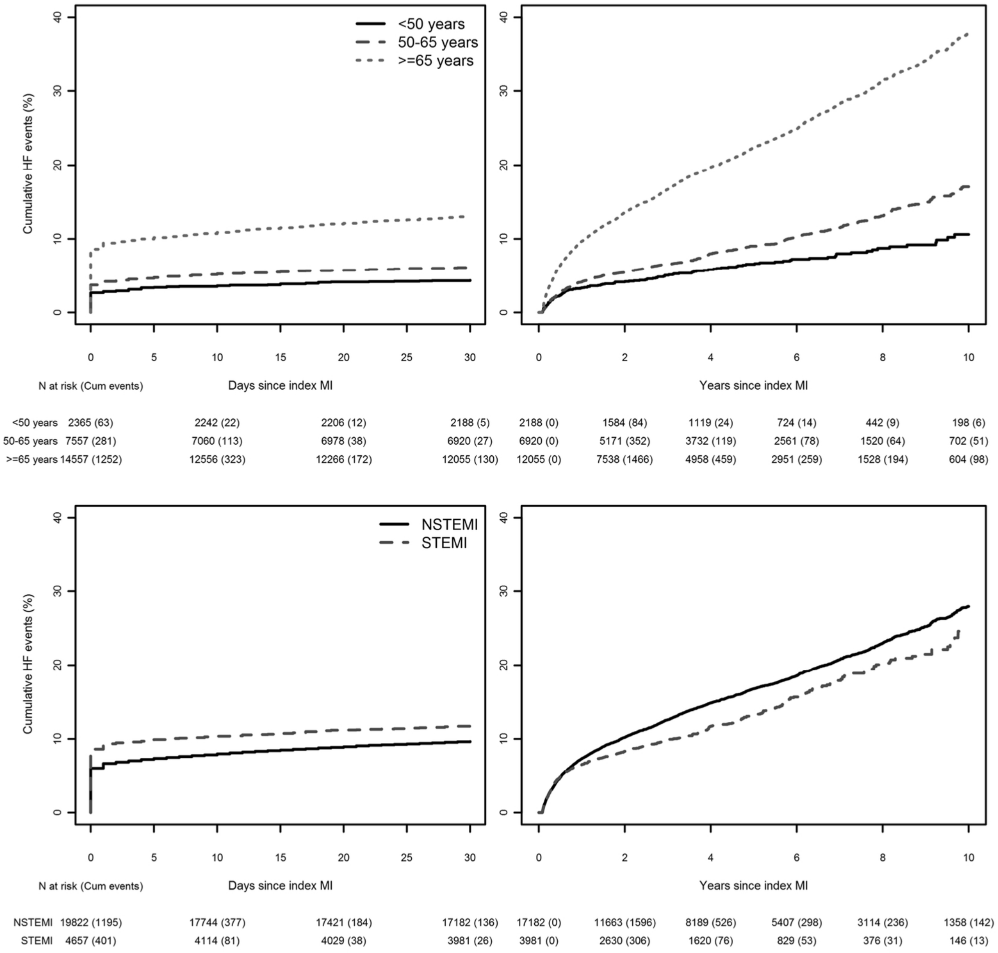


139x139mm (300 x 300 DPI)

**Supplementary Data**

## An Electronic Health Records cohort study on Heart Failure Following Myocardial Infarction in England: Incidence and Predictors

Johannes M.I.H. Gho, Amand F. Schmidt, Laura Pasea, Stefan Koudstaal, Mar Pujades-Rodriguez, Spiros Denaxas, Anoop D. Shah, Riyaz S. Patel, Chris P. Gale, Arno W. Hoes, John G. Cleland, Harry Hemingway, Folkert W. Asselbergs

For peer review only

Phenotype coding lists

**CALIBER myocardial infarction phenotype**
Fatal or non-fatal myocardial infarction (MI) as recorded in any of the constituent CALIBER data sources. The type of myocardial infarction (ST elevation MI, STEMI; or non ST elevation MI, NSTEMI) is recorded only in MINAP and CPRD.

Read codes used to identify myocardial infarction in primary care records (Clinical Research Practice Datalink)

| Category | Clinical code | Clinical term | Datasource lookup |
|---|---|---|---|
| **STEMI (3)** | G30X000 | Acute ST segment elevation myocardial infarction | 12229 |
| **NSTEMI (4)** | G307100 | Acute non-ST segment elevation myocardial infarction | 10562 |
| **Acute MI not further specified (5)** | 323..00 | ECG: myocardial infarction | 7783 |
| **Acute MI not further specified (5)** | 3233.00 | ECG: antero-septal infarct. | 26975 |
| **Acute MI not further specified (5)** | 3234.00 | ECG:posterior/inferior infarct | 26972 |
| **Acute MI not further specified (5)** | 3235.00 | ECG: subendocardial infarct | 55401 |
| **Acute MI not further specified (5)** | 3236.00 | ECG: lateral infarction | 52705 |
| **Acute MI not further specified (5)** | 323Z.00 | ECG: myocardial infarct NOS | 59032 |
| **Acute MI not further specified (5)** | 889A.00 | Diab mellit insulin-glucose infus acute myocardial infarct | 61670 |
| **Acute MI not further specified (5)** | G30..00 | Acute myocardial infarction | 241 |
| **Acute MI not further specified (5)** | G30..12 | Coronary thrombosis | 2491 |
| **Acute MI not further specified (5)** | G30..13 | Cardiac rupture following myocardial infarction (MI) | 30421 |
| **Acute MI not further specified (5)** | G30..15 | MI - acute myocardial infarction | 1677 |
| **Acute MI not further specified (5)** | G30..16 | Thrombosis - coronary | 13571 |
| **Acute MI not further specified (5)** | G300.00 | Acute anterolateral infarction | 12139 |
| **Acute MI not further specified (5)** | G301.00 | Other specified anterior myocardial infarction | 5387 |
| **Acute MI not further specified (5)** | G301000 | Acute anteroapical infarction | 40429 |
| **Acute MI not further specified (5)** | G301100 | Acute anteroseptal infarction | 17872 |
| **Acute MI not further specified (5)** | G301z00 | Anterior myocardial infarction NOS | 14897 |
| **Acute MI not further specified (5)** | G302.00 | Acute inferolateral infarction | 8935 |
| **Acute MI not further specified (5)** | G303.00 | Acute inferoposterior infarction | 29643 |
| **Acute MI not further specified (5)** | G304.00 | Posterior myocardial infarction NOS | 23892 |
| **Acute MI not further specified (5)** | G305.00 | Lateral myocardial infarction NOS | 14898 |
| **Acute MI not further specified (5)** | G306.00 | True posterior myocardial infarction | 63467 |
| **Acute MI not further specified (5)** | G307.00 | Acute subendocardial infarction | 3704 |
| **Acute MI not further specified (5)** | G307000 | Acute non-Q wave infarction | 9507 |

For peer review only

| | | | |
|---|---|---|---|
| **Acute MI not further specified (5)** | G308.00 | Inferior myocardial infarction NOS | 1678 |
| **Acute MI not further specified (5)** | G309.00 | Acute Q-wave infarct | 30330 |
| **Acute MI not further specified (5)** | G30B.00 | Acute posterolateral myocardial infarction | 32854 |
| **Acute MI not further specified (5)** | G30X.00 | Acute transmural myocardial infarction of unspecif site | 29758 |
| **Acute MI not further specified (5)** | G30y.00 | Other acute myocardial infarction | 34803 |
| **Acute MI not further specified (5)** | G30y000 | Acute atrial infarction | 28736 |
| **Acute MI not further specified (5)** | G30y100 | Acute papillary muscle infarction | 62626 |
| **Acute MI not further specified (5)** | G30y200 | Acute septal infarction | 41221 |
| **Acute MI not further specified (5)** | G30yz00 | Other acute myocardial infarction NOS | 46017 |
| **Acute MI not further specified (5)** | G30z.00 | Acute myocardial infarction NOS | 14658 |
| **Acute MI not further specified (5)** | G31y100 | Microinfarction of heart | 68357 |
| **Acute MI not further specified (5)** | G38..00 | Postoperative myocardial infarction | 32272 |
| **Acute MI not further specified (5)** | G380.00 | Postoperative transmural myocardial infarction anterior wall | 46112 |
| **Acute MI not further specified (5)** | G381.00 | Postoperative transmural myocardial infarction inferior wall | 46276 |
| **Acute MI not further specified (5)** | G384.00 | Postoperative subendocardial myocardial infarction | 41835 |
| **Acute MI not further specified (5)** | G38z.00 | Postoperative myocardial infarction, unspecified | 68748 |
| **Acute MI not further specified (5)** | Gyu3400 | [X]Acute transmural myocardial infarction of unspecif site | 96838 |

Hospital Episode Statistics

| **Category (code)** | ICD10 code | ICD10 term |
|---|---|---|
| **Acute MI not further specified (5)** | I21 | Acute myocardial infarction |

OPCS

| **Category (code)** | OPCS 4 | OPCS 4 term |
|---|---|---|
| **Transluminal coronary thrombolysis (2)** | K50.2 | Percutaneous transluminal coronary thrombolysis using streptokinase |
| **Transluminal coronary thrombolysis (2)** | K50.3 | Percutaneous transluminal injection of therapeutic substance into coronary artery NEC |

Office for National Statistics (ONS)

| **Category (code)** | ICD10 code | ICD10 term |
|---|---|---|
| **Death from MI (3)** | I21 | Acute myocardial infarction |
| **Death from MI (3)** | I22 | Subsequent myocardial infarction |
| **Death from MI (3)** | I23 | Certain current complications following acute myocardial infarction |

Office for National Statistics (ONS) prior to 2000 (ICD9)

| **Category (code)** | ICD9 code | ICD9 term |
|---|---|---|
| **Death from myocardial infarction (3)** | 410 | Acute myocardial infarction |
| **Death from myocardial infarction (3)** | 4110 | Other acute and subacute forms of ischemic heart disease ; Postmyocardial infarction syndrome |

For peer review only

| | | |
|---|---|---|
| **Death from myocardial infarction (3)** | 4297 | Ill-defined descriptions and complications of heart disease ; Certain sequelae of myocardial infarction, not elsewhere classified |

Myocardial Ischaemia National Audit Project (MINAP)

1 Acute STEMI

2 Acute NSTEMI

## Phenotype coding lists

**CALIBER myocardial infarction phenotype**

Fatal or non-fatal myocardial infarction (MI) as recorded in any of the constituent CALIBER data sources. The type of myocardial infarction (ST elevation MI, STEMI; or non ST elevation MI, NSTEMI) is recorded only in MINAP and CPRD.

Read codes used to identify myocardial infarction in primary care records (Clinical Research Practice Datalink)

| Category | Clinical code | Clinical term | Datasource lookup |
|---|---|---|---|
| **STEMI (3)** | G30X000 | Acute ST segment elevation myocardial infarction | 12229 |
| **NSTEMI (4)** | G307100 | Acute non-ST segment elevation myocardial infarction | 10562 |
| **Acute MI not further specified (5)** | 323..00 | ECG: myocardial infarction | 7783 |
| **Acute MI not further specified (5)** | 3233.00 | ECG: antero-septal infarct. | 26975 |
| **Acute MI not further specified (5)** | 3234.00 | ECG:posterior/inferior infarct | 26972 |
| **Acute MI not further specified (5)** | 3235.00 | ECG: subendocardial infarct | 55401 |
| **Acute MI not further specified (5)** | 3236.00 | ECG: lateral infarction | 52705 |
| **Acute MI not further specified (5)** | 323Z.00 | ECG: myocardial infarct NOS | 59032 |
| **Acute MI not further specified (5)** | 889A.00 | Diab mellit insulin-glucose infus acute myocardial infarct | 61670 |
| **Acute MI not further specified (5)** | G30..00 | Acute myocardial infarction | 241 |
| **Acute MI not further specified (5)** | G30..12 | Coronary thrombosis | 2491 |
| **Acute MI not further specified (5)** | G30..13 | Cardiac rupture following myocardial infarction (MI) | 30421 |
| **Acute MI not further specified (5)** | G30..15 | MI - acute myocardial infarction | 1677 |
| **Acute MI not further specified (5)** | G30..16 | Thrombosis - coronary | 13571 |
| **Acute MI not further specified (5)** | G300.00 | Acute anterolateral infarction | 12139 |
| **Acute MI not further specified (5)** | G301.00 | Other specified anterior myocardial infarction | 5387 |
| **Acute MI not further specified (5)** | G301000 | Acute anteroapical infarction | 40429 |
| **Acute MI not further specified (5)** | G301100 | Acute anteroseptal infarction | 17872 |
| **Acute MI not further specified (5)** | G301z00 | Anterior myocardial infarction NOS | 14897 |
| **Acute MI not further specified (5)** | G302.00 | Acute inferolateral infarction | 8935 |
| **Acute MI not further specified (5)** | G303.00 | Acute inferoposterior infarction | 29643 |
| **Acute MI not further specified (5)** | G304.00 | Posterior myocardial infarction NOS | 23892 |
| **Acute MI not further specified (5)** | G305.00 | Lateral myocardial infarction NOS | 14898 |

For peer review only

| | | | |
|---|---|---|---|
| **Acute MI not further specified (5)** | G306.00 | True posterior myocardial infarction | 63467 |
| **Acute MI not further specified (5)** | G307.00 | Acute subendocardial infarction | 3704 |
| **Acute MI not further specified (5)** | G307000 | Acute non-Q wave infarction | 9507 |
| **Acute MI not further specified (5)** | G308.00 | Inferior myocardial infarction NOS | 1678 |
| **Acute MI not further specified (5)** | G309.00 | Acute Q-wave infarct | 30330 |
| **Acute MI not further specified (5)** | G30B.00 | Acute posterolateral myocardial infarction | 32854 |
| **Acute MI not further specified (5)** | G30X.00 | Acute transmural myocardial infarction of unspecif site | 29758 |
| **Acute MI not further specified (5)** | G30y.00 | Other acute myocardial infarction | 34803 |
| **Acute MI not further specified (5)** | G30y000 | Acute atrial infarction | 28736 |
| **Acute MI not further specified (5)** | G30y100 | Acute papillary muscle infarction | 62626 |
| **Acute MI not further specified (5)** | G30y200 | Acute septal infarction | 41221 |
| **Acute MI not further specified (5)** | G30yz00 | Other acute myocardial infarction NOS | 46017 |
| **Acute MI not further specified (5)** | G30z.00 | Acute myocardial infarction NOS | 14658 |
| **Acute MI not further specified (5)** | G31y100 | Microinfarction of heart | 68357 |
| **Acute MI not further specified (5)** | G38..00 | Postoperative myocardial infarction | 32272 |
| **Acute MI not further specified (5)** | G380.00 | Postoperative transmural myocardial infarction anterior wall | 46112 |
| **Acute MI not further specified (5)** | G381.00 | Postoperative transmural myocardial infarction inferior wall | 46276 |
| **Acute MI not further specified (5)** | G384.00 | Postoperative subendocardial myocardial infarction | 41835 |
| **Acute MI not further specified (5)** | G38z.00 | Postoperative myocardial infarction, unspecified | 68748 |
| **Acute MI not further specified (5)** | Gyu3400 | [X]Acute transmural myocardial infarction of unspecif site | 96838 |

Hospital Episode Statistics

| **Category (code)** | ICD10 code | ICD10 term |
|---|---|---|
| **Acute MI not further specified (5)** | I21 | Acute myocardial infarction |

OPCS

| **Category (code)** | OPCS 4 | OPCS 4 term |
|---|---|---|
| **Transluminal coronary thrombolysis (2)** | K50.2 | Percutaneous transluminal coronary thrombolysis using streptokinase |
| **Transluminal coronary thrombolysis (2)** | K50.3 | Percutaneous transluminal injection of therapeutic substance into coronary artery NEC |

Office for National Statistics (ONS)

| **Category (code)** | ICD10 code | ICD10 term |
|---|---|---|
| **Death from MI (3)** | I21 | Acute myocardial infarction |
| **Death from MI (3)** | I22 | Subsequent myocardial infarction |
| **Death from MI (3)** | I23 | Certain current complications following acute myocardial infarction |

Office for National Statistics (ONS) prior to 2000 (ICD9)

For peer review only

| **Category (code)** | ICD9 code | ICD9 term |
|---|---|---|
| **Death from myocardial infarction (3)** | 410 | Acute myocardial infarction |
| **Death from myocardial infarction (3)** | 4110 | Other acute and subacute forms of ischemic heart disease ; Postmyocardial infarction syndrome |
| **Death from myocardial infarction (3)** | 4297 | Ill-defined descriptions and complications of heart disease ; Certain sequelae of myocardial infarction, not elsewhere classified |

Myocardial Ischaemia National Audit Project (MINAP)

| | |
|---|---|
| 1 | Acute STEMI |
| 2 | Acute NSTEMI |

**CALIBER heart failure phenotype**

Primary care

1. Diagnosis of left ventricular dysfunction during echocardiogram during a consultation
2. Diagnosis of heart failure of valvular cause during a consultation
3. Diagnosis of heart failure due to hypertension during a consultation
4. Diagnosis of heart failure due to unspecified cause during a consultation
5. Diagnosis of heart failure not otherwise specified during a consultation

Secondary care

1. Diagnosis of heart failure due to hypertension as the primary diagnosis during a hospitalization
2. Diagnosis of heart failure not otherwise specified as the primary diagnosis during a hospitalization

Mortality

1. Hypertensive heart failure as the underlying cause of death
2. Heart failure not otherwise specified as the underlying cause of death

Read codes used to identify myocardial infarction in primary care records (Clinical Research Practice Datalink)

| **Category (code)** | **Clinical code** | **Clinical term** | **Datasource lookup** |
|---|---|---|---|
| **Heart failure due to valvular disease (3)** | G580400 | Congestive heart failure due to valvular disease | 94870 |
| **Heart failure due to hypertension (4)** | G210.00 | Malignant hypertensive heart disease | 50157 |
| **Heart failure due to hypertension (4)** | G210000 | Malignant hypertensive heart disease without CCF | 95334 |
| **Heart failure due to hypertension (4)** | G210100 | Malignant hypertensive heart disease with CCF | 72668 |
| **Heart failure due to hypertension (4)** | G211100 | Benign hypertensive heart disease with CCF | 52127 |
| **Heart failure due to hypertension (4)** | G21z100 | Hypertensive heart disease NOS with CCF | 62718 |
| **Heart failure due to hypertension (4)** | G230.00 | Malignant hypertensive heart and renal disease | 67232 |
| **Heart failure due to hypertension (4)** | G232.00 | Hypertensive heart&renal dis wth (congestive) heart failure | 21837 |
| **Heart failure due to hypertension (4)** | G234.00 | Hyperten heart&renal dis+both(congestv)heart and renal fail | 57987 |
| **Heart failure due to other cause (5)** | G1yz100 | Rheumatic left ventricular failure | 22262 |
| **Heart failure NOS (6)** | 1O1..00 | Heart failure confirmed | 9913 |
| **Heart failure NOS (6)** | 662W.00 | Heart failure annual review | 30779 |
| **Heart failure NOS (6)** | 662p.00 | Heart failure 6 month review | 83502 |
| **Heart failure NOS (6)** | 8B29.00 | Cardiac failure therapy | 24503 |
| **Heart failure NOS (6)** | 8H2S.00 | Admit heart failure emergency | 32898 |
| **Heart failure NOS (6)** | 9Or0.00 | Heart failure review completed | 19380 |
| **Heart failure NOS (6)** | G400.00 | Acute cor pulmonale | 8464 |
| **Heart failure NOS (6)** | G41z.11 | Chronic cor pulmonale | 5695 |
| **Heart failure NOS (6)** | G554000 | Congestive cardiomyopathy | 5141 |
| **Heart failure NOS (6)** | G554011 | Congestive obstructive cardiomyopathy | 68766 |
| **Heart failure NOS (6)** | G58..00 | Heart failure | 2062 |
| **Heart failure NOS (6)** | G58..11 | Cardiac failure | 1223 |
| **Heart failure NOS (6)** | G580.00 | Congestive heart failure | 398 |

For peer review only

| | | | |
|---|---|---|---|
| **Heart failure NOS (6)** | G580.11 | Congestive cardiac failure | 2906 |
| **Heart failure NOS (6)** | G580.12 | Right heart failure | 10079 |
| **Heart failure NOS (6)** | G580.13 | Right ventricular failure | 10154 |
| **Heart failure NOS (6)** | G580.14 | Biventricular failure | 9524 |
| **Heart failure NOS (6)** | G580000 | Acute congestive heart failure | 23707 |
| **Heart failure NOS (6)** | G580100 | Chronic congestive heart failure | 32671 |
| **Heart failure NOS (6)** | G580200 | Decompensated cardiac failure | 27884 |
| **Heart failure NOS (6)** | G580300 | Compensated cardiac failure | 11424 |
| **Heart failure NOS (6)** | G581.00 | Left ventricular failure | 884 |
| **Heart failure NOS (6)** | G581.11 | Asthma - cardiac | 23481 |
| **Heart failure NOS (6)** | G581.13 | Impaired left ventricular function | 5942 |
| **Heart failure NOS (6)** | G581000 | Acute left ventricular failure | 5255 |
| **Heart failure NOS (6)** | G582.00 | Acute heart failure | 27964 |
| **Heart failure NOS (6)** | G58z.00 | Heart failure NOS | 4024 |
| **Heart failure NOS (6)** | G58z.12 | Cardiac failure NOS | 17278 |
| **Heart failure NOS (6)** | G5yy900 | Left ventricular systolic dysfunction | 8966 |
| **Heart failure NOS (6)** | G5yyA00 | Left ventricular diastolic dysfunction | 12550 |
| **Heart failure NOS (6)** | R2y1000 | [D]Cardiorespiratory failure | 20324 |
| **Left ventricular dysfunction (3)** | 585f.00 | Echocardiogram shows left ventricular systolic dysfunction | 11284 |
| **Left ventricular dysfunction (3)** | 585g.00 | Echocardiogram shows left ventricular diastolic dysfunction | 11351 |

Hospital Episode Statistics

| **Category (code)** | **ICD10 code** | **ICD10 term** |
|---|---|---|
| **HF - hypertension cause (4)** | I110 | Hypertensive heart disease with (congestive) heart failure |
| **HF - hypertension cause (4)** | I130 | Hypertensive heart and renal disease with (congestive) heart failure |
| **HF - hypertension cause (4)** | I132 | Hypertensive heart and renal disease with both (congestive) heart failure and renal failure |
| **HF - other unspecified cause (5)** | I260 | Pulmonary embolism with mention of acute cor pulmonale |
| **HF - not otherwise specified (6)** | I50 | Heart failure |

Office for National Statistics (ONS)

| **Category (code)** | **ICD10 code** | **ICD10 term** |
|---|---|---|
| **Hypertensive heart failure (4)** | I110 | Hypertensive heart disease with (congestive) heart failure |
| **Hypertensive heart failure (4)** | I130 | Hypertensive heart and renal disease with (congestive) heart failure |
| **Hypertensive heart failure (4)** | I132 | Hypertensive heart and renal disease with both (congestive) heart failure and renal failure |
| **Heart failure with other specified cause (5)** | I260 | Pulmonary embolism with mention of acute cor pulmonale |
| **Heart failure with unspecified cause (6)** | I50 | Heart failure |

Office for National Statistics (ONS) prior to 2000 (ICD9)

| **Category (code)** | **ICD9 code** | **ICD9 term** |
|---|---|---|
| **Heart failure with other specified cause (5)** | 4151 | Acute pulmonary heart disease ; Pulmonary embolism and infarction |
| **Heart failure with unspecified cause (6)** | 428 | Heart failure |

Myocardial Ischaemia National Audit Project (MINAP)

1 CCF

**CALIBER baseline definitions**

See also: https://www.caliberresearch.org

For peer review only

Definitions used for smoking at baseline
CPRD:
**smoking_cessation_gprd**
Prescription of smoking cessation medication in primary care. Currently lookup is performed from using BNF chapter 4.10.2.
**smoking_status_gprd**
An individual record of smoking status in primary care. A patient may have multiple records of smoking status.
HES:
**smoking_status_hes**
Smoking status recorded in HES. Currently there are only ICD-10 codes for smoking, no codes for ex-smoking or non-smoking.

Definitions used for hypertension at baseline
CPRD:
**ht_gprd**
Diagnosis of hypertension
HES:
**ht_hes**
Hypertension diagnosis recorded in secondary care.

Definitions used for diabetes at baseline
CPRD:
**dm_gprd**
Type of diabetes diagnosis at a particular point in time.
**diabdiag_gprd**
GP diagnosis of diabetes mellitus using a specific type I or type II diagnosis in the Read diagnosis chapter (i.e. codes starting with C08, C09, C10E, C10F). Vague terms such as `juvenile onset diabetes` or `insulin dependent diabetes` are not included.
HES:
**dm_hes**
Diagnosis of diabetes in secondary care (HES).

For peer review only

Supplementary Methods

**Ethical approval**

CALIBER was approved by the Lewisham Local Research Ethics Committee (ref: 09/H0810/16 date: 08/04/2009) and the Ethics and Confidentiality Committee (ECC) (ref: ECC 2-06(b)/2009 CALIBER dataset). CALIBER has been registered with the University College London Data Protection Officer (ref: Z6364106/2009/2/26). CALIBER EHR data are anonymised; individual informed consent was not sought from study participants.

**Multiple imputation algorithm**

Multiple imputation was implemented using the 'mice' package for R, to replace missing values in prognostic covariates (body mass index, smoking, systolic and diastolic blood pressure).[1] Imputation models included general practice and 3-year periods from 1998 to 2010 of index myocardial infarction (MI) and:

1. All the baseline covariates as used in the multivariable analysis (age, sex, index of multiple deprivation, hypertension, diabetes, type of MI, body mass index, smoking, systolic blood pressure, diastolic blood pressure);
2. Baseline measurements of covariates not considered in the multivariable analysis (alcohol intake, haemoglobin, white blood cell count, creatinine, alanine aminotransferase (ALAT), total cholesterol, HDL cholesterol);
3. Baseline medications prior to index MI (antiplatelet, oral anticoagulant, statin, ACE inhibitor, angiotensin receptor blocker (ARB), beta-blocker, calcium channel blocker, loop diuretic, aldosterone antagonist, digoxin);
4. Coexisting medical conditions (history of atrial fibrillation, peripheral arterial disease, depression, cancer, chronic obstructive pulmonary disease);
5. The Nelson-Aalen hazard and the event status for the outcomes analysed.

Missing data was multiply imputed and across 20 datasets a (stratified) multivariable Cox regression model was fit and results were combined using Rubin's rules.[2]

References

1. van Buuren S. Multiple imputation of discrete and continuous data by fully conditional specification. Stat Methods Med Res 2007;16:219-42.
2. Carpenter JR, Kenward MG. *Multiple Imputation and its Application*: Wiley; 2013.

For peer review only

**Table S1 - Hazard ratios for heart failure in patients following a first myocardial infarction using the competing risks regression based on Fine and Gray's proportional subhazards model to account for the competing risk of all cause mortality**

| | Model 1 | Model 2 | Model 3 | Model 4 |
|---|---|---|---|---|
| | HR (95% CI) | HR (95% CI) | HR (95% CI) | HR (95% CI) |
| Age, per 10 years increase | 1.41 (1.38 – 1.44) | 1.39 (1.36 – 1.42) | 1.36 (1.33 – 1.39) | 1.35 (1.32 – 1.38) |
| Men | 1.04 (0.99 – 1.10) | 1.07 (1.01 – 1.13) | 1.06 (1.00 – 1.12) | 1.05 (1.00 – 1.11) |
| Index of multiple deprivation | | overall p = <0.001 | overall p = <0.001 | overall p = <0.001 |
| Q1 | | Reference | Reference | Reference |
| Q2 | | 1.11 (1.03 – 1.21) | 1.11 (1.02 – 1.20) | 1.10 (1.01 – 1.20) |
| Q3 | | 1.21 (1.12 – 1.32) | 1.20 (1.11 – 1.31) | 1.19 (1.09 – 1.29) |
| Q4 | | 1.24 (1.14 – 1.35) | 1.24 (1.14 – 1.34) | 1.22 (1.12 – 1.32) |
| Q5 | | 1.36 (1.25 – 1.47) | 1.35 (1.25 – 1.47) | 1.32 (1.21 – 1.43) |
| History of hypertension | | 1.15 (1.09 – 1.22) | 1.13 (1.07 – 1.19) | 1.14 (1.08 – 1.21) |
| History of diabetes | | 1.39 (1.29 – 1.49) | 1.38 (1.29 – 1.48) | 1.36 (1.27 – 1.47) |
| History of atrial fibrillation | | | 1.50 (1.40 – 1.61) | 1.49 (1.39 – 1.60) |
| Type of MI | | | | |
| STEMI | | | 1.10 (1.03 – 1.18) | 1.11 (1.03 – 1.18) |
| History of peripheral arterial disease | | | | 1.29 (1.19 – 1.40) |
| History of COPD | | | | 1.18 (1.09 – 1.29) |
| ACE inhibitor use before MI | | | | 1.01 (0.95 – 1.08) |
| Angiotensin receptor blocker (ARB) use before MI | | | | 0.94 (0.85 – 1.05) |
| Beta-blocker use before MI | | | | 0.94 (0.88 – 1.00) |

Complete case analysis with the addition of clinically relevant variables per model (n = 24,479, n events = 5,775). Overall p-values are shown for categorical covariates. ACE=Angiotensin-converting enzyme; CI=confidence interval; COPD=chronic obstructive pulmonary disease; MI=myocardial infarction; NSTEMI=non-ST-segment-elevation myocardial infarction; STEMI=ST-segment-elevation myocardial infarction; Q=quintile; HR=ratio of the subdistribution hazards.

For peer review only

**Table S2 - Hazard ratios for heart failure in patients following a first myocardial infarction in sensitivity analyses**

| | Model 5<br>Cox regression<br>complete case without multiple imputation<br>n = 4,898<br>n events = 1,159 | Model 5<br>Cox regression<br>with multiple imputation<br>n = 24,479<br>n events = 5,775 | Model 5<br>Competing risk analysis<br>with complete case<br>n = 4,898<br>n events = 1,159 |
|---|---|---|---|
| | HR (95% CI) | HR (95% CI) | HR (95% CI) |
| Age, per 10 years increase | 1.48 (1.38 – 1.60) | 1.47 (1.43 – 1.51) | 1.44 (1.35 – 1.54) |
| Men | 1.03 (0.89 – 1.19) | 1.07 (1.01 – 1.13) | 1.07 (0.95 – 1.22) |
| Body mass index (BMI in kg/m$^2$) | p = 0.005 | p = <0.001 | p = < 0.001 |
| Underweight (18.5 <) | 0.78 (0.48 – 1.32) | 0.76 (0.58 – 0.99) | 0.71 (0.45 – 1.12) |
| Normal (18.5; 25) | Reference | Reference | Reference |
| Overweight (25, 30) | 0.84 (0.71 – 0.99) | 0.84 (0.76 – 0.92) | 0.84 (0.73 – 0.97) |
| Obese (>30) | 1.12 (0.94 – 1.34) | 0.98 (0.90 – 1.08) | 1.17 (1.00 – 1.36) |
| Index of multiple deprivation | p = 0.040 | p < 0.001 | p = < 0.001 |
| Q1 | Reference | Reference | Reference |
| Q2 | 1.22 (0.94 – 1.58) | 1.08 (0.98 – 1.19) | 1.35 (1.10 – 1.66) |
| Q3 | 1.34 (1.03 – 1.75) | 1.17 (1.06 – 1.30) | 1.40 (1.14 – 1.71) |
| Q4 | 1.23 (0.93 – 1.63) | 1.16 (1.05 – 1.29) | 1.31 (1.07 – 1.61) |
| Q5 | 1.45 (1.09 – 1.92) | 1.25 (1.12 – 1.40) | 1.61 (1.33 – 1.96) |
| Smoking status | p = 0.792 | p = 0.012 | p = 0.952 |
| Non-smoker | Reference | Reference | Reference |
| Current smoker | 0.95 (0.77 – 1.14) | 1.14 (1.04 – 1.25) | 1.02 (0.86 – 1. 20) |
| Ex-smoker | 0.98 (0.83 – 1.15) | 1.03 (0.94 – 1.12) | 0.99 (0.87 – 1.14) |
| History of hypertension | 1.14 (0.96 – 1.35) | 1.16 (1.09 – 1.24) | 1.18 (1.02 – 1.37) |
| History of diabetes | 1.28 (1.09 – 1.49) | 1.46 (1.36 – 1.58) | 1.23 (1.08 – 1.39) |
| History of atrial fibrillation | 1.49 (1.25 – 1.76) | 1.63 (1.51 – 1.75) | 1.37 (1.18 – 1.59) |
| Type of MI | | | |
| STEMI | 1.15 (0.97 – 1.37) | 1.18 (1.10 – 1.27) | 1.10 (0.95 – 1.28) |
| History of peripheral arterial disease | 1.53 (1.25 – 1.87) | 1.37 (1.25 – 1.50) | 1.33 (1.13 – 1.57) |
| History of COPD | 1.21 (0.99 – 1.48) | 1.24 (1.15 – 1.38) | 1.22 (1.03 – 1.44) |
| Systolic blood pressure, per 10 mmHg increase | 0.99 (0.95 – 1.03) | 1.00 (0.98 – 1.02) | 1.01 (0.97 – 1.04) |
| Diastolic blood pressure, per 10 mmHg increase | 1.01 (0.94 – 1.09) | 0.99 (0.96 – 1.03) | 0.99 (0.93 – 1.06) |
| ACE inhibitor use before MI | 1.01 (0.87 – 1.17) | 1.06 (0.99 – 1.14) | 1.07 (0.94 – 1.21) |
| Angiotensin receptor blocker (ARB) use before MI | 0.99 (0.95 – 1.03) | 1.00 (0.90 – 1.12) | 0.93 (0.78 – 1.12) |
| Beta-blocker use before MI | 1.01 (0.94 – 1.09) | 0.94 (0.88 – 1.00) | 0.90 (0.79 – 1.02) |

Using multivariable Cox regression without and with multiple imputation and the competing risks regression based on Fine and Gray's proportional subhazards model to account for the competing risk of all cause mortality (n = 4898, n events = 1159). Additional prognostic covariates: body mass index, smoking, systolic and diastolic blood pressure. Overall p-values are shown for categorical covariates. HR=hazard ratio; CI=confidence interval; MI=myocardial infarction; NSTEMI=non-ST-segment-elevation myocardial infarction; STEMI=ST-segment-elevation myocardial infarction; COPD=chronic obstructive pulmonary disease; ACE=Angiotensin-converting enzyme; Q=quintile.

For peer review only

**Table S3 - Hazard ratios for heart failure in patients following a first myocardial infarction stratified on having a previous cancer diagnosis or not.**

| | Model 4 **Subjects without a history of cancer** n = 21,719 n events = 5,029 | Model 4 **Subjects with a history of cancer** n = 2,760 n events = 746 |
|---|---|---|
| | HR (95% CI) | HR (95% CI) |
| Age, per 10 years increase | 1.49 (1.45 – 1.53) | 1.45 (1.30 – 1.61) |
| Men | 1.08 (1.01 – 1.15) | 1.13 (0.93 – 1.37) |
| Index of multiple deprivation | overall p = <0.001 | overall p = <0.001 |
| Q1 | Reference | Reference |
| Q2 | 1.08 (0.97 – 1.20) | 1.07 (0.77 – 1.48) |
| Q3 | 1.17 (1.05 – 1.31) | 1.27 (0.91 – 1.78) |
| Q4 | 1.17 (1.04 – 1.31) | 1.14 (0.79 – 1.66) |
| Q5 | 1.25 (1.10 – 1.41) | 1.43 (0.97 – 2.11) |
| History of hypertension | 1.11 (1.03 – 1.18) | 1.45 (1.16 – 1.81) |
| History of diabetes | 1.53 (1.41 – 1.66) | 1.19 (0.91 – 1.55) |
| History of atrial fibrillation | 1.62 (1.49 – 1.76) | 1.67 (1.33 – 2.09) |
| Type of MI | | |
| STEMI | 1.18 (1.09 – 1.28) | 1.01 (0.77 – 1.31) |
| History of peripheral arterial disease | 1.39 (1.26 – 1.54) | 1.19 (0.88 – 1.62) |
| History of COPD | 1.30 (1.18 – 1.43) | 1.36 (1.03 – 1.81) |
| ACE inhibitor use before MI | 1.11 (1.03 – 1.20) | 1.00 (0.79 – 1.26) |
| Angiotensin receptor blocker (ARB) use before MI | 1.06 (0.93 – 1.20) | 0.92 (0.65 – 1.31) |
| Beta-blocker use before MI | 0.97 (0.90 – 1.04) | 0.99 (0.79 – 1.24) |

All analyses are based on a complete case analysis. Overall p-values are shown for categorical covariates. ACE=Angiotensin-converting enzyme; CI=confidence interval; COPD=chronic obstructive pulmonary disease; MI=myocardial infarction; NSTEMI=non-ST-segment-elevation myocardial infarction; STEMI=ST-segment-elevation myocardial infarction; Q=quintile.

For peer review only

**Table S4 - Hazard ratios for heart failure in patients following a first myocardial infarction stratified on a history of revascularization.**

| | Model 4 **Subjects without a history of revascularization** n = 21,160 n events = 5,222 | Model 4 **Subjects with a history of revascularization** n = 3.319 n events = 553 |
|---|---|---|
| | HR (95% CI) | HR (95% CI) |
| Age, per 10 years increase | 1.48 (1.44 – 1.52) | 1.30 (1.17 – 1.44) |
| Men | 1.07 (1.01 – 1.14) | 0.96 (0.75 – 1.23) |
| Index of multiple deprivation | overall p = <0.001 | overall p = <0.001 |
| Q1 | Reference | Reference |
| Q2 | 1.06 (0.95 – 1.17) | 1.13 (0.80 – 1.61) |
| Q3 | 1.14 (1.02 – 1.27) | 1.39 (0.95 – 2.03) |
| Q4 | 1.15 (1.02 – 1.28) | 1.34 (0.85 – 2.02) |
| Q5 | 1.22 (1.08 – 1.38) | 1.29 (0.84 – 1.99) |
| History of hypertension | 1.14 (1.07 – 1.22 | 1.18 (0.92 – 1.50) |
| History of diabetes | 1.49 (1.37 – 1.61) | 1.32 (1.00 – 1.75) |
| History of atrial fibrillation | 1.61 (1.48 – 1.74) | 1.90 (1.39 – 2.58) |
| Type of MI | | |
| STEMI | 1.14 (1.05 – 1.23) | 1.39 (1.07 – 1.79) |
| History of peripheral arterial disease | 1.40 (1.27 – 1.55) | 1.22 (0.88 – 1.62) |
| History of COPD | 1.30 (1.18 – 1.43) | 1.36 (0.92 – 2.03) |
| ACE inhibitor use before MI | 1.11 (1.03 – 1.19) | 1.15 (0.89 – 1.49) |
| Angiotensin receptor blocker (ARB) use before MI | 1.06 (0.94 – 1.19) | 0.79 (0.54 – 1.17) |
| Beta-blocker use before MI | 1.00 (0.94 – 1.07) | 0.84 (0.65 – 1.08) |

All analyses are based on a complete case analysis. Overall p-values are shown for categorical covariates. ACE=Angiotensin-converting enzyme; CI=confidence interval; COPD=chronic obstructive pulmonary disease; MI=myocardial infarction; NSTEMI=non-ST-segment-elevation myocardial infarction; STEMI=ST-segment-elevation myocardial infarction; Q=quintile.

For peer review only

**Supplementary Figure 1 – Cumulative incidence curves for HF accounting for mortality as first event after index MI**

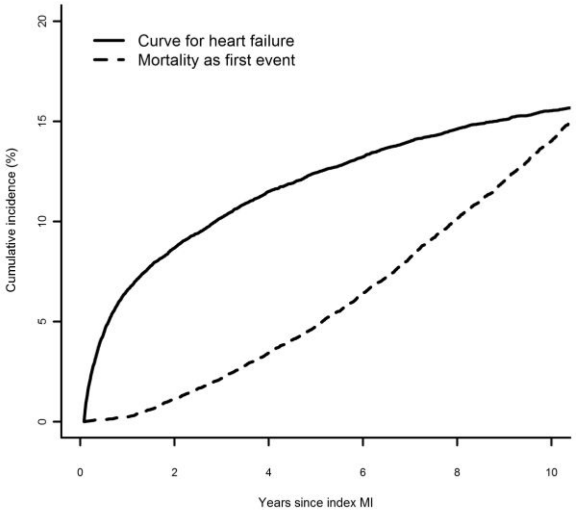


| | 0 | 2 | 4 | 6 | 8 | 10 |
|---|---|---|---|---|---|---|
| N at risk | 21663 | 14293 | 9809 | 6236 | 3489 | 1504 |
| Cum HF events | 0 | 1902 | 602 | 351 | 267 | 155 |
| Cum ACM events | 0 | 292 | 635 | 814 | 990 | 971 |

Cumulative incidence curves adjusted for competing risk of mortality as first event. 10-year follow-up in patients who survived the first 30 days and did not develop HF during the first 30 days (30 days event free).

For peer review only

STROBE Statement—checklist of items that should be included in reports of observational studies

| | Item No | Recommendation |
|---|---|---|
| **Title and abstract** | 1 ✓ | (*a*) ✓*page 2* - Indicate the study's design with a commonly used term in the title or the abstract |
| | | (*b*) ✓*page 2* - Provide in the abstract an informative and balanced summary of what was done and what was found |
| **Introduction** | | |
| Background/rationale | 2✓ | ✓*page 4* - Explain the scientific background and rationale for the investigation being reported |
| Objectives | 3✓ | ✓ *page 4* - State specific objectives, including any prespecified hypotheses |
| **Methods** | | |
| Study design | 4✓ | ✓*pages 4 through 6* - Present key elements of study design early in the paper |
| Setting | 5✓ | Describe the setting, locations, and relevant dates, including periods of recruitment, exposure, follow-up, and data collection |
| Participants | 6✓ | ✓*page 5* - (*a*) *Cohort study*—Give the eligibility criteria, and the sources and methods of selection of participants. Describe methods of follow-up<br>*Case-control study*—Give the eligibility criteria, and the sources and methods of case ascertainment and control selection. Give the rationale for the choice of cases and controls<br>*Cross-sectional study*—Give the eligibility criteria, and the sources and methods of selection of participants |
| | | (*b*) *Cohort study*—For matched studies, give matching criteria and number of exposed and unexposed<br>*Case-control study*—For matched studies, give matching criteria and the number of controls per case |
| Variables | 7✓ | ✓*page 5* - Clearly define all outcomes, exposures, predictors, potential confounders, and effect modifiers. Give diagnostic criteria, if applicable |
| Data sources/ measurement | 8✓* | ✓*page 5* - For each variable of interest, give sources of data and details of methods of assessment (measurement). Describe comparability of assessment methods if there is more than one group |
| Bias | 9✓ | ✓*page 6* - Describe any efforts to address potential sources of bias |
| Study size | 10✓ | ✓*page 7 and figure 1*- Explain how the study size was arrived at |
| Quantitative variables | 11✓ | ✓*page 6* - Explain how quantitative variables were handled in the analyses. If applicable, describe which groupings were chosen and why |
| Statistical methods | 12✓ | (*a*) *NA* Describe all statistical methods, including those used to control for confounding |
| | | (*b*) *NA* Describe any methods used to examine subgroups and interactions |
| | | (*c*) ✓*page 6* - Explain how missing data were addressed |
| | | (*d*) ✓*page 6* - *Cohort study*—If applicable, explain how loss to follow-up was addressed<br>*Case-control study*—If applicable, explain how matching of cases and controls was addressed<br>*Cross-sectional study*—If applicable, describe analytical methods taking account of sampling strategy |
| | | (*e*) ✓Describe any sensitivity analyses |

For peer review only


For peer review only - http://bmjopen.bmj.com/site/about/guidelines.xhtml

Continued on next page

For peer review only



For peer review only - http://bmjopen.bmj.com/site/about/guidelines.xhtml

| | | |
|---|---|---|
| **Results** | | |
| Participants | 13* | ✓*page 6* - (a) Report numbers of individuals at each stage of study—eg numbers potentially eligible, examined for eligibility, confirmed eligible, included in the study, completing follow-up, and analysed |
| | | ✓*page 6 and figure 1* - (b) Give reasons for non-participation at each stage |
| | | ✓*figure 1* - (c) Consider use of a flow diagram |
| Descriptive data | 14*✓ | (a) ✓*table 1* - Give characteristics of study participants (eg demographic, clinical, social) and information on exposures and potential confounders |
| | | (b) ✓*table 1* - Indicate number of participants with missing data for each variable of interest |
| | | (c) ✓*table 1*- *Cohort study*—Summarise follow-up time (eg, average and total amount) |
| Outcome data | 15* | ✓*table 1, figures 2-3 and pages 7-8 Cohort study*—Report numbers of outcome events or summary measures over time |
| | | *Case-control study*—Report numbers in each exposure category, or summary measures of exposure |
| | | *Cross-sectional study*—Report numbers of outcome events or summary measures |
| Main results | 16 | ✓*table 2, appendix tables* and pages 7-8 - (*a*) Give unadjusted estimates and, if applicable, confounder-adjusted estimates and their precision (eg, 95% confidence interval). Make clear which confounders were adjusted for and why they were included |
| | | *NA* (*b*) Report category boundaries when continuous variables were categorized |
| | | ✓ absolute rates reported in pages 7-8 - (*c*) If relevant, consider translating estimates of relative risk into absolute risk for a meaningful time period |
| Other analyses | 17 | ✓*pages 7-8* - Report other analyses done—eg analyses of subgroups and interactions, and sensitivity analyses |
| **Discussion** | | |
| Key results | 18 | ✓*page 8* Summarise key results with reference to study objectives |
| Limitations | 19 | ✓*pages 9-10* - Discuss limitations of the study, taking into account sources of potential bias or imprecision. Discuss both direction and magnitude of any potential bias |
| Interpretation | 20 | ✓*pages 8-10* Give a cautious overall interpretation of results considering objectives, limitations, multiplicity of analyses, results from similar studies, and other relevant evidence |
| Generalisability | 21 | ✓ *page 9* Discuss the generalisability (external validity) of the study results |
| **Other information** | | |
| Funding | 22 | ✓*page 11* Give the source of funding and the role of the funders for the present study and, if applicable, for the original study on which the present article is based |

*Give information separately for cases and controls in case-control studies and, if applicable, for exposed and unexposed groups in cohort and cross-sectional studies.

**Note:** An Explanation and Elaboration article discusses each checklist item and gives methodological background and published examples of transparent reporting. The STROBE checklist is best used in conjunction with this article (freely available on the Web sites of PLoS Medicine at http://www.plosmedicine.org/, Annals of Internal Medicine at http://www.annals.org/, and Epidemiology at http://www.epidem.com/). Information on the STROBE Initiative is available at www.strobe-statement.org.

For peer review only

# An Electronic Health Records cohort study on Heart Failure Following Myocardial Infarction in England: Incidence and Predictors



| | |
|---|---|
| Journal: | *BMJ Open* |
| Manuscript ID | bmjopen-2017-018331.R3 |
| Article Type: | Research |
| Date Submitted by the Author: | 17-Oct-2017 |
| Complete List of Authors: | Gho, Johannes; Universitair Medisch Centrum Utrecht, Cardiology; Farr Institute of Health Informatics Research, University College London, Epidemiology and Public Health<br>Schmidt, Amand; Faculty of Science and Engineering, Groningen Research Institute of Pharmacy; University College London, Institute of Cardiovascular Science<br>Pasea, Laura; Farr Institute of Health Informatics Research, University College London, Epidemiology and Public Health<br>Koudstaal, Stefan; Farr Institute of Health Informatics Research, University College London, Epidemiology and Public Health; University Medical Centre Utrecht, Department of Cardiology<br>Pujades Rodriguez, Mar; Farr Institute of Health Informatics Research, University College London, Epidemiology and Public Health; University of Leeds, Medical Research Council Bioinformatics Centre, Leeds Institute of Biomedical and Clinical Sciences<br>Denaxas, S; Farr Institute of Health Informatics Research, University College London, Epidemiology and Public Health<br>Shah, Anoop; UCL, Farr Institute of Health Informatics research<br>Patel, Riyaz; Farr Institute of Health Informatics Research, University College London, Epidemiology and Public Health<br>Gale, Chris; University of Leeds, Medical Research Council Bioinformatics Centre, Leeds Institute for Cardiovascular and Metabolic Medicine<br>Hoes, Arno; Julius Center for Health Sciences and Primary Care, University Medical Center, Utrecht<br>Cleland, J; Imperial College London, National Heart & Lung Institute, Royal Brompton & Harefield Hospitals<br>Hemingway, Harry; Farr Institute of Health Informatics Research, University College London, Epidemiology and Public Health<br>Asselbergs, Folkert; University Medical Centre Utrecht, Department of Cardiology; ICIN-Netherlands Heart Institute, Durrer Center for Cardiogenetic Research |
| <b>Primary Subject Heading</b>: | General practice / Family practice |
| Secondary Subject Heading: | Cardiovascular medicine, Epidemiology, General practice / Family practice, Health informatics, Public health |
| Keywords: | Heart failure < CARDIOLOGY, Myocardial infarction < CARDIOLOGY, electronic health records |

SCHOLARONE™
Manuscripts

For peer review only

# An Electronic Health Records cohort study on Heart Failure Following Myocardial Infarction in England: Incidence and Predictors

Johannes M.I.H. Gho, MD, PhD,[1,2,*] Amand F. Schmidt, PhD,[2,3,4*] Laura Pasea, MSc,[1] Stefan Koudstaal, MD, MSc, PhD,[1,2] Mar Pujades-Rodriguez, PhD,[1,5] Spiros Denaxas, PhD,[1] Anoop D. Shah, MRCP,[1] Riyaz S. Patel, MD, PhD,[1,3] Chris P. Gale, PhD, FRCP,[6] Arno W. Hoes, MD, PhD,[7] John G. Cleland, MD, FRCP,[8] Harry Hemingway, FFPH, FRCP,[1] Folkert W. Asselbergs, MD, PhD,[1,2,3,9]

[1]Farr Institute of Health Informatics Research, UCL Institute of Health Informatics, University College London, London, UK
[2]Department of Cardiology, Division Heart and Lungs, University Medical Center Utrecht, Utrecht, the Netherlands
[3]Institute of Cardiovascular Science, Faculty of Population Health Sciences, University College London, London, UK
[4]Groningen Research Institute of Pharmacy, University of Groningen, Groningen, the Netherlands
[5]Medical Research Council Bioinformatics Centre, Leeds Institute of Biomedical and Clinical Sciences, University of Leeds, Leeds, UK
[6]Medical Research Council Bioinformatics Centre, Leeds Institute for Cardiovascular and Metabolic Medicine, University of Leeds, Leeds, UK
[7]Julius Center for Health Sciences and Primary Care, University Medical Center Utrecht and Utrecht University, Utrecht, the Netherlands
[8]National Heart & Lung Institute, Royal Brompton & Harefield Hospitals, Imperial College London, London, UK
[9]Durrer Center for Cardiogenetic Research, ICIN-Netherlands Heart Institute, Utrecht, the Netherlands
* Shared first author

**Corresponding author:**
A Floriaan Schmidt
Institute of Cardiovascular Science, University College London
222 Euston Road, London NW1 2DA, United Kingdom
E-mail: amand.schmidt@ucl.ac.uk; a.f.schmidt@rug.nl
Tel: 0044 (0)20 3549 5625

**Word count text**: 3427
**Word count abstract**: 265
**Number of references**: 32
**Number of tables**: 2
**Number of figures**: 3
**(Web)appendix**: 1

For peer review only

**Abstract**

**Objectives** To investigate the incidence and determinants of heart failure (HF) following a myocardial infarction (MI) in a contemporary cohort of MI patients using routinely collected primary and hospital care electronic health records.

**Methods** Data were used from the CALIBER programme, linking electronic health records in England from primary care, hospital admissions, an MI registry and mortality data. Subjects were eligible if they were 18 years or older, did not have a history of HF, and survived a first MI. Factors associated with time to HF were examined using Cox proportional hazard models.

**Results** Of the 24,479 MI patients 5,775 (23.6%) developed HF during a median follow-up of 3.7 years (incidence rate per 1000 person-years 63.8 (95% CI: 62.2 – 65.5). Baseline characteristics significantly associated with developing HF were: atrial fibrillation [HR 1.62, 95% CI 1.51 – 1.75], age [per 10 years increase: 1.45, 1.41 – 1.49], diabetes [1.45, 1.35 – 1.56], peripheral arterial disease [1.38, 1.26 – 1.51], COPD [1.28, 1.17 – 1.40], greater socioeconomic deprivation [$5^{th}$ vs. $1^{st}$ quintile 1.27, 1.13 – 1.41], ST-segment elevation MI at presentation [1.19, 1.11 – 1.27], and hypertension [1.16, 1.09 – 1.23]. Results were robust to various sensitivity analyses such as competing risk analysis and multiple imputation.

**Conclusion** In England, one in four survivors of a first MI develop HF within four years. This contemporary study demonstrates MI patients are at considerable risk of HF. Baseline patient characteristics associated with time till HF were identified, which may be used to target preventive strategies.


**Keywords:** heart failure; myocardial infarction; incidence; risk factors; electronic health records



For peer review only - http://bmjopen.bmj.com/site/about/guidelines.xhtml

**Strengths and limitations of this study**

- ❖ This study based on the use of linked electronic health records from general practitioners and hospital records, describes the current burden of heart failure in a representative sample of patients with a first myocardial infarction.
- ❖ The linkage of data from three sources (disease registry, primary care and hospital records) improved diagnostic ascertainment and accuracy in timing of events.
- ❖ Misclassification of drug exposure was likely to be minimal, as prescriptions issued during consultation are automatically recorded.
- ❖ Risk factor adjustment might have been incomplete given that information regarding baseline BMI, smoking and blood pressure was missing for 34 to up to 70% of patients.
- ❖ Stratified methods were used to account for potential calendar and centre effects, and competing risk models were used to adjust for potential competing effects on heart failure and mortality.

**Abbreviations list:** heart failure (HF); myocardial infarction (MI); ST-segment elevation MI (STEMI); hazard ratio (HR); confidence interval (CI); chronic obstructive pulmonary disease (COPD); body mass index (BMI).

For peer review only

## Introduction

Research describing the incidence of heart failure (HF) following myocardial infarction (MI) is limited, mainly originating from the thrombolytic era, often using small sample sizes with contradictory findings, making it difficult to provide evidence based medicine. For example, a previous UK study among almost 900 patients hospitalized with MI in 1998, found that one fifth developed HF during their hospital stay and a further third following hospital discharge[1]. More recently, a Swedish study found a 5-year cumulative risk of HF after MI of 21.8% in the calendar period 2004-2013[2]. Further, a Danish nationwide cohort study reported an incidence of HF at 90-days following MI of 19.6% in 2009-10[3]. Differences between these studies could be related to a number of factors, for example change in treatment, national policies, or definitions of heart failure, all of which potentially limit the generalizability of results. We used a large contemporary and representative sample of patients with MI based on electronic health records (EHR) to 1) describe the current incidence of HF after MI in England, using both primary and hospital care data, and 2) explore patient characteristics predictive of post MI HF.

## Methods

### Study design

Data were used from the CALIBER dataset, which included linked data from (i) primary care electronic health records, with diagnoses coded using the Read terminology (Clinical Practice Research Datalink, CPRD) from 226 consenting general practices[4], (ii) hospital care administrative records (Hospital Episode Statistics, HES) and disease registry/audit records (Myocardial Ischaemia National Audit Project, MINAP), and (iii) the mortality register (from UK Office for National Statistics, ONS) [5].

### Setting and participants

Patients were eligible if they were aged ≥18 years, registered in CPRD practices in England consenting to data linkage, with at least one year of up-to-standard pre-study follow-up (meeting CPRD practice quality standards), and experiencing a first MI between 1st January 1998 and 25th March 2010. The first record of



For peer review only - http://bmjopen.bmj.com/site/about/guidelines.xhtml

MI across the linked data sources was considered as the index event and subsequent MI records within 30 days from the same or other sources were considered as representing the same event [6]. Patients with a fatal index MI or a history of HF before their index MI were excluded. Patients were censored if they ceased to be registered at a participating general practice, at the date of death or the administrative censoring of the dataset (25th March 2010).

Variables and data sources

Study variables were derived from diagnoses recorded across several controlled clinical terminology and statistical classification systems: Read, ICD-9 or ICD-10, medication prescription information or the Office of Population Censuses and Surveys Classification of Interventions and Procedures (OPCS-4) codes (see https://rdrr.io/rforge/CALIBERcodelists/man/PRODDICT.html) with the electronic health record phenotyping algorithms published at https://caliberresearch.org/portal [5].

For continuous variables, the most recent measurement recorded in CPRD in the year before study entry was used as the baseline value. Data before study entry were used to determine the prognostic potential of age (years, using the date of birth), sex, ethnicity, social deprivation (Index of Multiple Deprivation, IMD score as recorded in ONS in quintiles), smoking, alcohol use, history of cardiovascular disease, previous coronary revascularization, history of diabetes, history of thyroid disease, history of chronic obstructive pulmonary disease (COPD), history of depression and history of non-metastatic cancer. (see the CALIBER portal https://caliberresearch.org/portal for details). Diabetes and hypertension diagnosis were based on Read codes from primary care or ICD-10 codes from Hospital Episode Statistics, both of which were classified using primary care consultation records and hospitals diagnosis records. Socioeconomic status was based on the Index of Multiple Deprivation which includes seven domains of deprivation (income deprivation, employment deprivation, health deprivation and disability, education, skills and training deprivation, barriers to housing and services, living environment deprivation and crime) [7]. The index MI event was characterized using ECG findings (e.g. ST-segment-elevation myocardial infarction (STEMI), or non-STEMI (NSTEMI)), site of infarction, mode and timing of reperfusion and peak cardiac biomarkers (troponin I, troponin T and CK). MI was defined by linking all of the CALIBER data sources

For peer review only

(e.g., Hospital Episode Statistics and CPRD), with the type of MI recorded in MINAP and CPRD, see for more detail https://www.caliberresearch.org and the appendix. Characteristics regarding the index MI (e.g., type of revascularization and delay from symptom onset to delivery of reperfusion therapy) were derived from MINAP. This nationwide registry is part of an annual audit which records over 20 key MI variables [8]. Herrett and colleagues [6] validated the current approach and showed the necessity of linking multiple sources to ascertain MI (see appendix for codes used). The primary study outcome was the first HF event following MI, which similar to MI, was recorded in multiple CALIBER data sources; echocardiographic findings and NYHA class were unavailable. All phenotypes were created and validated using a robust methodology described elsewhere [9] and have been used in previously published studies[10,11].

Statistical methods

Incidence rates (cases per 1000 person-years) and Kaplan-Meier cumulative incidence rates of HF were estimated, with Kaplan-Meier curves stratified by age (<50, 50-65, ≥65 years) and type of MI. Association between baseline variables with the onset of HF following MI were explored using Cox proportional hazard models. The proportional hazards assumption was checked using Schoenfeld residuals, and by nonparametric correlation coefficients between survival time and the parameter specific residuals. All models were stratified on general practice and calendar year periods of enrolment (1998-2001, 2001-2004, 2004-2007 and 2007-2010). Stratified models were used instead of frailty models because the former does not make any distributional assumption. Models were sequentially adjusted for: 1) age and sex, 2) cardiovascular risk factors, 3) type of MI, and 4) co-morbidities and prescribed medication. Associations are presented as hazard ratios (HRs) with 95% confidence intervals (CI). Analyses were performed using R (version 3.3.2)[12].

Sensitivity analyses

Due to the availability of EHR from multiple sources, clinical diagnoses and prescriptions were completely recorded. Biomarker and lifestyle measurements such as smoking, BMI, and blood pressure were however, incompletely recorded (see Table 1). It is probable that these data were preferentially recorded

For peer review only

in subjects perceived to be at a higher risk for early progression of CVD. Pairwise analyses regressing a complete case indicator on observed variables indeed showed considerable dependency between recorded data and missingness (results not shown), violating the “missing completely at random” assumption. This dependency was utilized by multiply imputing missing data using the mice package, which was implemented using 20 imputed datasets and pooled based on Rubin’s rules (Supplementary)[13].

To account for the fact that patients may have died before the onset of HF (e.g., competing risk by mortality), analyses were repeated using Fine and Gray models[14,15]. As a hypothetical example of competing risk, let us assume that out of 100 MI subjects, one develops HF and 99 die. In a Cox’s regression analysis all 99 dead subjects would be censored (implicitly, and incorrectly, assuming they may develop HF later in time), and the hazard of HF would equal 1/1. Instead a Fine and Gray model recognizes the 99 died subjects could never develop HF and hence calculates the hazard as 1/100.

In addition to these sensitivity analyses, we performed a cancer- and revascularization-stratified analysis. Due to discrepancies in sample size between the subgroups we decided against formal interaction testing, which may suffer from inflated false positive rates and lower power in such settings[16].

Ethical approval

CALIBER has received ethics approval (Supplementary). This study is in compliance with the Declaration of Helsinki and was approved by the ISAC (Independent Scientific Advisory Committee) for MHRA Database Research (protocol no. 14_198R) and the MINAP Academic Group and is registered with ClinicalTrials.gov (NCT02384213).

## Results

Subjects and baseline characteristics



For peer review only - http://bmjopen.bmj.com/site/about/guidelines.xhtml

In total there were 52,770 patients with an index MI during the study period (Figure 1). Excluding patients with a fatal index MI (n = 15,104), a prior history of HF (n = 3,876), missing information on the type of MI (n = 97) and subjects with less than 1 year of follow-up prior to the indexing MI event (n = 9,214), resulted in an analytical cohort of 24,479 patients. Patients were recruited from 226 GP practices, with the median practice enrolling 101 patients (quartile (Q1) 65 and Q3 150). Of the included subjects 4,657 were STEMI and 19,822 NSTEMI patients (0% missing), 15,969 (65.2%) were men (0% missing), 4,538 (42.1%) currently smoked (56% missing), 12,258 (50.1%) had hypertension (0% missing), and 3,014 (12.3%) had a history of diabetes at baseline (0% missing). In total, 6,129 (25.0%) were prescribed beta-blockers (0% missing) and 5,039 (20.6%) angiotensin converting enzyme (ACE) inhibitors (0% missing) prior to their indexing MI (Table 1). The number of patients identified across the previously defined calendar periods was reasonably stable, with a minimum of 5219 subjects in the period 1998-2001 compared to a maximum of 6838 subjects identified in the period 2004-2007.

Incidence

Patients contributed 90,482 person years of follow-up, during a median follow-up time of 3.7 years (IQR: 1.5; 6.7), 5,775 patients (23.6%) developed HF. The crude incidence rate of HF following a first MI was 63.8 (95% CI: 62.2 – 65.5) per 1000 person-years. Within the first 30 days post MI follow-up 2,438 (10.0%) patients developed HF (Figure 2). From day 30 onwards 3,337 (15.8%) of MI patients (event free during the first 30 days) developed HF, with 6.8% experiencing an HF event within the first year (Figure 2). The incidence of HF during the first 30-days of follow-up was 4.3% (102) in patients younger than 50 years, 6.0% (459) in 50-65 year olds and 12.9% (1877) in those 65 years and older, with HF incidence increasing proportionally as time progressed towards 10 years (Figure 3 top row). The 30-day incidence of HF was 9.5% (1892 events) for NSTEMI subjects and 11.7% (546 events) for STEMI subjects. Excluding patients who experienced a HF within the first 30 days showed that STEMI patients had a lower incidence of HF than NSTEMI subjects (Figure 3 lower panels). At 57 days the crude cumulative risk of HF in the NSTEMI subjects surpassed that of the STEMI subjects for the first time (0.0151 versus 0.0147), with the curves further diverging at 73 days of follow-up (since indexing MI).

For peer review only

Over the entire follow-up 5,921 subjects died, of which 3,538 were free of HF at the time. During the first 30 days of follow-up only 1 patient died, limiting the potential for competing risk by all-cause mortality. In patients who did not have HF after 30 days accounting for competing risk by all-cause mortality attenuated the cumulative risk to be about 15% after 10 years of follow-up (Appendix Figure). Furthermore, the cumulative risks of all-cause mortality and HF converged as follow-up time progressed towards 10 years.

Predictors of HF after multivariable adjustments

Table 2 describes the results of the previously described nested Cox's models, showing little change in estimates between the increasingly complex models. Focusing on the final model, the hazard of HF increased with atrial fibrillation [HR 1.62, 95%CI 1.51 – 1.75], age [per 10 years increase: 1.45, 1.41 – 1.49], diabetes [1.45, 1.35 – 1.56], peripheral arterial disease [1.38, 1.26 – 1.51], COPD [1.28, 1.17 – 1.40], hypertension [1.16, 1.09 – 1.23], higher socioeconomic deprivation (p-value for the 5 groups < 0.001), and STEMI at presentation [1.19, 1.11 – 1.27)]. Accounting for competing risk by all-cause mortality had little impact on the presented results (Appendix Table S1). After multiply imputing the data models were extended to include smoking, BMI, and systolic and diastolic blood pressure variables which showed BMI, male sex and smoking to be conditionally independent prognostic factors (Supplementary Table S2). The extended model 5 was implemented using imputed data, and non-imputed (complete case) data, resulting in similar HR estimates (magnitude and direction). Stratifying the sample on cancer diagnosis or history of revascularization showed broadly similar results between subgroups (See appendix table S3 and S4, focusing on the confidence intervals), however precision was low due to the limited number of patients with a history of revascularization or cancer.

## Discussion

In this large population-based study using linked electronic health records, 23.6% of patients who survived a first MI developed HF during a median follow-up of 3.7 years, resulting in an incidence rate of 64 cases per 1000 person-years. Incident HF was associated with increasing age, higher socioeconomic

For peer review only

deprivation, a history of diabetes, atrial fibrillation, peripheral arterial disease, COPD, STEMI at presentation, BMI and smoking.

A previous Canadian study, using data from the period 1994-2000[17], found that 71% of elderly patients without HF at index admission developed HF within 5 years' time after an MI, whereas the mortality due to MI decreased in the same period. The Framingham Heart Study[18], using data from 1990 to 1999, found a 5-years post-MI HF incidence of 31.9% after MI; lower than found in the Canadian study. Importantly, both studies showed a higher incidence of post-MI HF than our more contemporary English cohort. This lower HF incidence is likely due to continued improvements of MI treatment strategies, which are reflected in a decreased HF incidence over calendar time. For example, a 20,812 sample of MI patients hospitalized in Western Australia showed that the overall 1-year incidence of HF after MI decreased from 28.1% 1996 in to 16.5% in 2007[19]. This decline is confirmed further by a national Swedish hospital discharge and death registry study reporting a one third decline in incidence between 1993-2004[20].The same Swedish group recently reported that this trend persists for the period 2004-2013[2] and showed improved pharmacological treatment and early revascularization in this period. During a median follow-up of 4 years, 19% of the patients were re-hospitalized because of HF. An explanation for the higher percentage in our study is probably the inclusion of HF diagnosis made in the GP setting next to hospital records decreasing misclassification of HF events. Future efforts are needed to harmonize these national data sources to compare daily care and HF epidemiology across different countries.

Prognostic factors

Similar to the current study, Torabi et al[21] reported that HF after MI increased steeply with age. Socioeconomic deprivation has been shown to be an independent predictor of HF development and associated with an increased incidence of HF in MI-free subjects [22,23]. Socioeconomic deprivation has also been associated with more frequent hospital admission and higher mortality in HF patients[24–26]. The current study extends these observations to HF incidence after an MI. Furthermore, we showed that a history of diabetes, atrial fibrillation, peripheral arterial disease, COPD, STEMI at presentation, BMI and smoking are all independently prognostic of HF after MI. Interestingly, despite the size of the collected

For peer review only



For peer review only - http://bmjopen.bmj.com/site/about/guidelines.xhtml

sample, sex only became significantly associated with HF after accounting for differences in BMI and smoking. Furthermore, the impact of male sex (HR of 1.07 95%CI 1.01 – 1.13) was modest indicating relative sex equality. It is of note that blood pressure was not significantly associated with HF. However, blood pressure measurements were frequently missing, which (even after multiple imputation) may be the cause of the observed lack of association hence this deserves independent exploration. In this light it is important to note that the diagnosis of hypertension was associated with HF. Potentially this discrepancies between the association of blood pressure measurement and hypertension diagnosis can be explained by noting that a recorded diagnosis is indicative of long-term hypertensions, which may be different than a single blood pressure measurement in terms of prognosis. Further, given that both variables were included in the same model, the observed difference in association may suggest that conditional on hypertension, blood pressure itself is only modestly associated to HF, if at all.

Strengths and limitations

The linkage of multiple electronic health record sources from primary and hospital care allowed for the collection of a representative sample[27], which enabled us to explore the prognostic value of routinely collected data in primary care records and to detect non-hospitalized HF cases. The population of CPRD practices has been shown broadly representative of the UK population[4,28]. In total 226 GP practices consented to data linkage with Hospital Episode Statistics, MINAP, and the Office for National Statistics (containing 3.9% of the population of England in 2006). A potential limitation is that the ascertainment of cardiovascular outcomes was not based on clinical criteria (e.g., validated questionnaires and properly conducted physical examinations), practices of medical coding will have changed over time and there could be subgroups of patients with left ventricular dysfunction without clinical symptoms. Calendar dependent changes over time were accounted for by using time-period stratified Cox models. We further wish to highlight the infrequent use of PCI in STEMI patients, which was shown to be representative of the slower uptake in England[29]. Similarly, 32% of the patients used antiplatelet drugs at baseline which may be a reflection of non-MI CVD burden. The Kaplan-Meier plots for subtype of MI indicate a possible violation of the proportional hazard assumption for this variable. However, these plots represent the pairwise associations between MI subtype and time to HF, and as such assume that MI subtype is


For peer review only - http://bmjopen.bmj.com/site/about/guidelines.xhtml

independent of other prognostic factors, which is known to be false. The importance of conditioning on covariates is underlined by noting the Kaplan-Meier plots indicating a protective effect of STEMI versus NSTEMI, which was significantly reversed after correcting for covariates (models 3-4). Similarly, instead of using Kaplan-Meier plots to assess the proportional hazard assumption of the *crude* associations, we used Schoenfeld residuals to explore this assumption for the *conditional* associations (multivariable model 4); which did not show any violations. Based on model 4 the absolute correlation between the Schoenfeld residuals and time was < 0.10 (e.g., for MI subtype this was 0.06) indicating an absence of relevant interaction by time (e.g., non-proportionality of hazard). Residual confounding due to medication use (or other missing/mis-specified variables) might be another potential source of bias, however our intention was not to perform a causal analysis between drug prescriptions and time to HF. As such it is interesting to note that despite the large sample size we did not observe a significant association of ACE, ARB or beta-blocker prescribed prior to the indexing MI event and time to HF. We adhered to CPRD recommendations to obtain up-to-standard baseline data by excluding patients with less than 1 year at CPRD practice prior to index MI. Previously Lewis and colleagues showed that 3 months after registration with a new practice most patient characteristics were updated correctly, which approximated 100% after 1 year of follow-up[30]. Assuming that duration of the CPRD follow-up is independent of the relations assessed here, excluding such patients should not hamper generalizability of results. Using the first MI recorded in the database without a prior history of HF, might have introduced bias due to left truncation (e.g., some subjects may already have experienced an MI before enrolment)[31]. However CALIBER holds longitudinal records from primary and hospital care, making it unlikely a large part of the patients were misclassified as MI free and the one year of follow-up prior to entry further decreases the likelihood of misclassifying patients[30]. While the current data is adequate to identify subjects with a first MI, the sub-classification of MI patients into STEMI and NSTEMI, despite recent improvements, is known to be error prone [32]. As such, results for MI type need replication using higher quality data, in perhaps purposely designed studies. Due to our interest in HF occurrence after a first MI selection bias (e.g., index event bias) was introduced[33]. This index event bias does not impact the descriptive or prognostic value of the association presented, and is mostly relevant if one wants to develop an intervention based on the associations presented; which was not the aim of this study. Additionally, we note that we reduced the

For peer review only

influence of selection bias by accounting for dependencies between predictors. An important caveat of electronic health care records is that these data are predominantly focussed on recording diagnoses and prospections but not on their complement (i.e., who is **not** diseased or who **did** not receive a drug). As such we have assumed that subjects without a recorded drug prescription or diagnoses was unexposed or free of (that specific) disease. Provided that electronic registration is required for a patient to fill a prescription we can be fairly confident that we did not miss many prescribed treatments. However, it is likely that some subjects were misclassified as free of disease while in fact they were not. We've attempted to minimize this misclassification by linking data across multiple healthcare settings and data sources (MINAP, HES and CPRD, ONS).

A final limitation is that there is a possible delay between primary and hospital care records. Previous research has shown that MI events tend to be recorded in primary care after the HES or MINAP record [6]. The lower 30-day HF incidence in unclassified MI patients primarily derived from primary care could be partly explained because of a delay in coding. Therefore, we showed cumulative incidence rates in patients (alive and HF event free within the first 30 days) from 30 days after index MI to account for a delay in recording of MI in primary care. We were unable to differentiate between HF with preserved ejection fraction (HFpEF) and HF with reduced ejection fraction (HFrEF) as we had no access to detailed (echocardiographic) parameters to assess diastolic dysfunction. It is likely, however that the majority of our HF patients had developed systolic dysfunction after MI.

Conclusion

In this large cohort study using linked electronic health records in England from primary and hospital care, about one in four people developed HF within a median of four years after surviving a first MI. Increasing age, higher socioeconomic deprivation, a history of hypertension, diabetes, atrial fibrillation, peripheral arterial disease, COPD, smoking and STEMI at presentation were independent determinants new onset HF following MI.

**Authors' contributions**

For peer review only

JMIHG, AFS, LP, SK, MPR, SD, ADS, RSP, CPG, AWH, JGC, HH, FWA contributed to the idea and design of the study. JMIHG extracted and prepared the data for analysis. AFS and JMIHG performed the analysis. JMIHG drafted the manuscript, with revisions from AFS, LP, SK, MPR, SD, ADS, RSP, CPG, AWH, JGC, HH, FWA. JMIHG guaranties the quality and accuracy of the results presented.

**Competing interests**

We have read and understood BMJ policy on declaration of interests and declare that we have no competing interests

**Data Sharing Statement**

Access to raw data can be requested from the CPRD http://cprd.com

**Funding**

This study was supported by the National Institute for Health Research (RP-PG-0407–10314), Wellcome Trust (086091/Z/08/Z), the Medical Research Council Prognosis Research Strategy Partnership (G0902393/99558) and the Farr Institute of Health Informatics Research, funded by The Medical Research Council (MR/K006584/1), in partnership with Arthritis Research UK, the British Heart Foundation, Cancer Research UK, the Economic and Social Research Council, the Engineering and Physical Sciences Research Council, the National Institute of Health Research, the National Institute for Social Care and Health Research (Welsh Assembly Government), the Chief Scientist Office (Scottish Government Health Directorates) and the Wellcome Trust. Part of this work is funded through the Innovative Medicines Initiative 2 Joint Undertaking under grant agreement No 116074, BigData@Heart. AFS is funded by UCLH NIHR Biomedical Research Centre and is a UCL Springboard Population Health Sciences Fellow. HH is an NIHR Senior Investigator, and receives support from the NIHR University College Hospitals / University College London Biomedical Research Centre. FWA is supported by a Dekker scholarship-Junior Staff Member 2014T001 – Netherlands Heart Foundation and UCL Hospitals NIHR Biomedical Research Centre.

For peer review only



For peer review only - http://bmjopen.bmj.com/site/about/guidelines.xhtml

## References

1 Torabi A, Cleland JGF, Khan NK, *et al.* The timing of development and subsequent clinical course of heart failure after a myocardial infarction. *Eur Heart J* 2008;**29**:859–70.

2 Desta L, Jernberg T, Spaak J, *et al.* Risk and predictors of readmission for heart failure following a myocardial infarction between 2004 and 2013: A Swedish nationwide observational study. *Int J Cardiol* Published Online First: May 2017.http://linkinghub.elsevier.com/retrieve/pii/S0167527316348677 (accessed 22 Aug 2017).

3 Gjesing A, Gislason GH, Kober L, *et al.* Nationwide trends in development of heart failure and mortality after first-time myocardial infarction 1997-2010: A Danish cohort study. *Eur J Intern Med* 2014;**25**:731–8.

4 Herrett E, Gallagher AM, Bhaskaran K, *et al.* Data Resource Profile: Clinical Practice Research Datalink (CPRD). *Int J Epidemiol* 2015;**44**:827–36.

5 Denaxas SC, George J, Herrett E, *et al.* Data resource profile: Cardiovascular disease research using linked bespoke studies and electronic health records (CALIBER). *Int J Epidemiol* 2012;**41**:1625–38.

6 Herrett E, Shah AD, Boggon R, *et al.* Completeness and diagnostic validity of recording acute myocardial infarction events in primary care, hospital care, disease registry, and national mortality records: cohort study. *BMJ* 2013;**346**:f2350.

7 DCLG. The English Indices of Deprivation 2010. *Neighb Stat Release* 2011;:1–20.http://www.communities.gov.uk/publications/corporate/statistics/indices2010technicalreport

8 Herrett E, Smeeth L, Walker L, *et al.* The Myocardial Ischaemia National Audit Project (MINAP). *Heart* 2010;**96**:1264–7.http://www.ncbi.nlm.nih.gov/pubmed/23177996

9 Morley KI, Wallace J, Denaxas SC, *et al.* Defining Disease Phenotypes Using National Linked Electronic Health Records: A Case Study of Atrial Fibrillation. *PLoS One* 2014;**9**:e110900.

10 Rapsomaniki E, Timmis A, George J, *et al.* Blood pressure and incidence of twelve cardiovascular diseases: Lifetime risks, healthy life-years lost, and age-specific associations in 1·25 million people. *Lancet* 2014;**383**:1899–911. doi:10.1016/S0140-6736(14)60685-1

11 Shah AD, Langenberg C, Rapsomaniki E, *et al.* Type 2 diabetes and incidence of cardiovascular

For peer review only



For peer review only - http://bmjopen.bmj.com/site/about/guidelines.xhtml

diseases: a cohort study in 1·9 million people. *Lancet Diabetes Endocrinol* 2015;**3**:105–13. doi:10.1016/S2213-8587(14)70219-0

12 R Core Team. R: A language and environment for statistical computing. 2017.

13 Marshall A, Altman DG, Holder RL, *et al.* Combining estimates of interest in prognostic modelling studies after multiple imputation: current practice and guidelines. BMC Med. Res. Methodol. 2009;**9**:57-.

14 Satagopan JM, Ben-Porat L, Berwick M, *et al.* A note on competing risks in survival data analysis. *Br J Cancer* 2004;**91**:1229–35.http://www.nature.com/doifinder/10.1038/sj.bjc.6602102

15 Gray B. cmprsk: Subdistribution Analysis of Competing Risks. 2014.http://cran.r-project.org/package=cmprsk

16 Schmidt AF, Groenwold RHH, Knol MJ, *et al.* Exploring interaction effects in small samples increases rates of false-positive and false-negative findings: Results from a systematic review and simulation study. *J Clin Epidemiol* Published Online First: 2014. doi:10.1016/j.jclinepi.2014.02.008

17 Ezekowitz JA, Kaul P, Bakal JA, *et al.* Declining in-hospital mortality and increasing heart failure incidence in elderly patients with first myocardial infarction. *J Am Coll Cardiol* 2009;**53**:13–20.

18 Velagaleti RS, Pencina MJ, Murabito JM, *et al.* Long-term trends in the incidence of heart failure after myocardial infarction. *Circulation* 2008;**118**:2057–62.

19 Hung J, Teng T-HK, Finn J, *et al.* Trends from 1996 to 2007 in incidence and mortality outcomes of heart failure after acute myocardial infarction: a population-based study of 20,812 patients with first acute myocardial infarction in Western Australia. *J Am Heart Assoc* 2013;**2**:e000172.

20 Shafazand M, Rosengren A, Lappas G, *et al.* Decreasing trends in the incidence of heart failure after acute myocardial infarction from 1993-2004: a study of 175,216 patients with a first acute myocardial infarction in Sweden. *Eur J Heart Fail* 2011;**13**:135–41.

21 Torabi A, Cleland JG, Rigby AS, *et al.* Development and course of heart failure after a myocardial infarction in younger and older people. *J Geriatr Cardiol* 2014;**11**:1–12.http://www.pubmedcentral.nih.gov/articlerender.fcgi?artid=3981977&tool=pmcentrez&rendertype=abstract

22 McAlister FA, Murphy NF, Simpson CR, *et al.* Influence of socioeconomic deprivation on the

For peer review only



For peer review only - http://bmjopen.bmj.com/site/about/guidelines.xhtml

primary care burden and treatment of patients with a diagnosis of heart failure in general practice in Scotland: population based study. *BMJ* 2004;**328**:1110.

23 Hawkins NM, Jhund PS, McMurray JJ V, *et al.* Heart failure and socioeconomic status: Accumulating evidence of inequality. *Eur J Heart Fail* 2012;**14**:138–46.

24 Philbin EF, Dec GW, Jenkins PL, *et al.* Socioeconomic status as an independent risk factor for hospital readmission for heart failure. *Am J Cardiol* 2001;**87**:1367–71.

25 Struthers AD, Anderson G, Donnan PT, *et al.* Social deprivation increases cardiac hospitalisations in chronic heart failure independent of disease severity and diuretic non-adherence. *Heart* 2000;**83**:12–6.

26 Pujades-Rodriguez M, Timmis A, Stogiannis D, *et al.* Socioeconomic deprivation and the incidence of 12 cardiovascular diseases in 1.9 million women and men: implications for risk prediction and prevention. *PLoS One* 2014;**9**:e104671.

27 Mann RD, Andrews EB. *Pharmacovigilance: Second Edition*. 2007.

28 Walley T, Mantgani A. The UK General Practice Research Database. *Lancet (London, England)* 1997;**350**:1097–9.

29 Chung SC, Gedeborg R, Nicholas O, *et al.* Acute myocardial infarction: A comparison of short-term survival in national outcome registries in Sweden and the UK. *Lancet* 2014;**383**:1305–12.

30 Lewis JD, Bilker WB, Weinstein RB, *et al.* The relationship between time since registration and measured incidence rates in the General Practice Research Database. *Pharmacoepidemiol Drug Saf* 2005;**14**:443–51.

31 Hazelbag CM, Klungel OH, van Staa TP, *et al.* Left truncation results in substantial bias of the relation between time-dependent exposures and adverse events. *Ann Epidemiol* 2015;**25**:590–6.

32 Alexandrescu R, Bottle A, Jarman B, *et al.* Current ICD10 codes are insufficient to clearly distinguish acute myocardial infarction type: a descriptive study. *BMC Health Serv Res* 2013;**13**:468.http://www.ncbi.nlm.nih.gov/pubmed/24195773

33 Dahabreh IJ, Kent DM. Index event bias as an explanation for the paradoxes of recurrence risk research. *Jama* 2011;**305**:822–3.

For peer review only

## Figure titles and legends

**Figure 1 – Flowchart of study population**

CALIBER=Cardiovascular disease research using Linked Bespoke studies and electronic health Records; CPRD=Clinical Practice Research Datalink; HF=heart failure; MI=myocardial infarction.

**Figure 2 – Incidence of HF after index MI**

Kaplan-Meier curves for crude incidence of HF after MI. **Left panel** 30-day follow-up after index MI. **Right panel** 10 year follow-up (median follow-up time was 3.7 years) in patients who survived the first 30 days and did not develop HF during the first 30 days (30-days event free). HF=heart failure; MI=myocardial infarction. “N at risk” represent the number of subjects at risk at a certain time point. “Cum events” represents the cumulative number of events since the previous time points. A spike histogram is provided (in red) at the top of the graphs providing information on the number of event across time.

**Figure 3 – Incidence of HF after index MI by group**

Kaplan-Meier curves for crude incidence of HF after MI stratified by group. **Top left panel:** 30-day follow-up after index MI stratified by age group. **Top right panel:** 10-year follow-up (median follow-up time was 3.7 years) in 30-day event free patients stratified by age group. Log-rank test of ≥65 year old patients compared to patients aged 50-65 years ($p<0.001$). **Bottom left panel:** 30-day follow-up after index MI stratified by MI type. **Bottom right panel:** 10-year follow-up (median follow-up time was 3.7 years) in patients who survived the first 30 days and did not develop HF during the first 30 days stratified by MI type. Log-rank test of NSTEMI compared to STEMI ($p<0.001$). HF=heart failure; MI=myocardial infarction. N at risk” represent the number of subjects at risk at a certain time point, with the cumulative number of events since the previous time points presented between brackets.


For peer review only - http://bmjopen.bmj.com/site/about/guidelines.xhtml

Table 1 - Baseline characteristics at index myocardial infarction

| | STEMI patients n = 4,657 | NSTEMI patients n = 19,822 | Total MI patients n = 24,479 | Unknown (%) |
|---|---|---|---|---|
| Follow-up time (years), median (IQR) | 3.3 (1.4–5.7) | 3.9 (1.5–7.1) | 3.7 (1.5–6.7) | 0% |
| Mean age, years (SD) | 65.7 (13.2) | 68.7 (13.2) | 68.1 (13.2) | 0% |
| Male sex | 3311 (71.1%) | 12658 (63.9%) | 15969 (65.2%) | 0% |
| Ethnicity | | | | |
| White | 3466 (76.4%) | 14288 (76.1%) | 17754 (76.2%) | 4.8% |
| Asian | 83 (1.8%) | 327 (1.7%) | 410 (1.8%) | |
| Black | 20 (0.4%) | 63 (0.3%) | 83 (0.4%) | |
| Other | 968 (21.3%) | 4085 (21.7%) | 5053 (21.7%) | |
| Body mass index (BMI in $kg/m^2$) | | | | |
| Underweight (18.5 <) | 22 (1.7%) | 120 (2.0%) | 142 (1.9%) | 70.0% |
| Normal (18.5; 25) | 335 (25.5%) | 1661 (27.4%) | 1996 (27.2%) | |
| Overweight (25, 30) | 557 (43.0%) | 2431 (40.2%) | 2988 (40.7%) | |
| Obese (>30) | 381 (29.4%) | 1842 (30.4%) | 2223 (30.2%) | |
| Index of multiple deprivation | | | | |
| Most deprived quintile | 892 (19.2%) | 3985 (20.2%) | 4877 (20%) | 0.4% |
| | | | | |
| **Risk factors before index MI** | | | | |
| Current smoker | 1235 (51.1%) | 3303 (39.5%) | 4538 (42.1%) | 55.9% |
| Excess alcohol consumption | 55 (9.0%) | 219 (8.1%) | 274 (8.3%) | 86.4% |
| History of atrial fibrillation | 358 (7.7%) | 2210 (11.1%) | 2568 (10.5%) | 0% |
| History of hypertension | 2117 (45.5%) | 10141 (51.2%) | 12258 (50.1%) | 0% |
| History of peripheral arterial disease | 236 (5.1%) | 1475 (7.4%) | 1711 (7%) | 0% |
| Previous revascularization | | | | |
| PCI | 700 (15.0%) | 2044 (10.3%) | 2744 (11.2%) | 0% |
| CABG | 126 (2.7%) | 843 (4.3%) | 969 (4.0%) | 0% |
| Previous TIA | 154 (3.3%) | 998 (5.0%) | 1152 (4.7%) | 0% |
| Previous stroke | 80 (1.7%) | 410 (2.1%) | 490 (2.0%) | 0% |
| History of diabetes | 538 (11.6%) | 2476 (12.6%) | 3014 (12.3%) | 0% |
| History of thyroid disease | 237 (5.1%) | 1374 (6.9%) | 1661 (6.6%) | 0% |
| History of COPD | 304 (6.5%) | 1634 (8.2%) | 1938 (7.9%) | 0% |
| History of non-metastatic cancer | 478 (10.3%) | 2282 (11.5%) | 2760 (11.3%) | 0% |
| | | | | |
| **Vital signs before admission** median (IQR) | | | | |
| Systolic blood pressure, mmHg | 140 (130–153) | 140 (130–154) | 140 (130–154) | 34.2% |
| Diastolic blood pressure, mmHg | 80 (74–88) | 80 (71–88) | 80 (72–88) | 34.2% |
| | | | | |
| **Biomarkers,** median (IQR) | | | | |
| Troponin I (maximum) | 18.4 (3.6–50.0) | 2.2 (0.3–10.0) | 3.88 (0.62–21.4) | 88.3% |
| Troponin T (maximum) | 1.8 (0.60–4.8) | 0.4 (0.14–1.1) | 0.65 (0.18–2) | 90.8% |
| CK (maximum) | 721 (222–1631) | 219 (97–648) | 331 (123–1068) | 79.8% |
| | | | | |
| **Biomarkers before index MI** mean (SD) | | | | |
| Haemoglobin, g/dL, | 14.1 (1.72) | 13.6 (1.84) | 13.7 (1.83) | 67.1% |
| White blood cell count | 8.0 (2.94) | 7.9 (2.92) | 7.9 (2.92) | 69.1% |
| Neutrophil count | 5.0 (2.12) | 5.0 (2.40) | 5.0 (2.35) | 71.9% |
| Platelets | 270 (85.2) | 266 (88.0) | 267 (87.5) | 69.1% |
| Erythrocyte sedimentation rate | 19.5 (18.8) | 22.2 (21.6) | 21.8 (21.2) | 89.4% |
| Creatinine, μmol/L | 98.5 (39.1) | 102.5 (47.3) | 102 (46.0) | 58.7% |
| eGFR - CKD-EPI | 70.4 (19.9) | 66.2 (19.9) | 67.0 (20.0) | 60.4% |
| Random glucose concentration, mmol/L | 7.09 (3.53) | 7.26 (3.89) | 7.23 (3.82) | 76.5% |
| Total cholesterol | 5.39 (1.45) | 5.24 (1.28) | 5.27 (1.31) | 63.4% |
| LDL cholesterol | 3.24 (1.12) | 3.08 (1.10) | 3.11 (1.11) | 81.3% |
| | | | | |
| **Revascularization characteristics** | | | | |
| Site of infarction | | | | |
| Anterior | 393 (40.2%) | 255 (30.7%) | 648 (35.9%) | 92.6% |
| Primary PCI | 258 (19.3%) | 73 (3.2%) | 331 (9.2%) | 85.3% |
| Prehospital fibrinolysis | 324 (7.0%) | 94 (0.5%) | 418 (1.7%) | 0% |
| Median (IQR) delay from symptom to reperfusion (min) | 150 (98 – 280) | 160 (101 – 296) | 153 (99 – 285) | 88.3% |
| | | | | |


For peer review only - http://bmjopen.bmj.com/site/about/guidelines.xhtml

| **Prescribed medication before index MI** | | | | |
|---|---|---|---|---|
| Antiplatelet | 867 (18.6%) | 6979 (35.2%) | 7846 (32.1%) | 0% |
| Oral anticoagulant | 67 (1.4%) | 624 (3.1%) | 691 (2.8%) | 0% |
| Statin | 825 (17.7%) | 5297 (26.7%) | 6122 (25.0%) | 0% |
| ACE inhibitor | 676 (14.5%) | 4363 (22.0%) | 5039 (20.6%) | 0% |
| Angiotensin receptor blocker (ARB) | 220 (4.7%) | 1208 (6.1%) | 1428 (5.8%) | 0% |
| Beta-blocker | 766 (16.4%) | 5363 (27.1%) | 6129 (25.0%) | 0% |
| Calcium channel blocker | 804 (17.3%) | 4621 (23.3%) | 5425 (22.2%) | 0% |
| Loop diuretic | 255 (5.5%) | 2188 (11.0%) | 2443 (10.0%) | 0% |
| Aldosterone antagonist | 24 (0.5%) | 198 (1.0%) | 222 (0.9%) | 0% |
| Digoxin | 53 (1.1%) | 515 (2.6%) | 568 (2.3%) | 0% |

IQR=inter-quartile range; SD=standard deviation; MI=myocardial infarction; STEMI=ST-segment-elevation myocardial infarction; PCI=Percutaneous Coronary Intervention; CABG=Coronary Artery Bypass Grafting; TIA=Transient Ischaemic Attack; COPD=Chronic Obstructive Pulmonary Disease; CK=Creatine kinase; ACE=Angiotensin-converting-enzyme. Prehospital and in-hospital fibrinolysis are not mutually exclusive.

For peer review only



For peer review only - http://bmjopen.bmj.com/site/about/guidelines.xhtml

**Table 2 - Hazard ratio for heart failure in patients following a first myocardial infarction using multivariable Cox regression**

| | Model 1<br>n = 24,479<br>n events = 5,775 | Model 2<br>n = 24,479<br>n events = 5,775 | Model 3<br>n = 24,479<br>n events = 5,775 | Model 4<br>n = 24,479<br>n events = 5,775 |
|---|---|---|---|---|
| | HR (95% CI) | HR (95% CI) | HR (95% CI) | HR (95% CI) |
| Age, per 10 years | 1.51 (1.48 – 1.55) | 1.49 (1.46 – 1.53) | 1.46 (1.43 – 1.50) | 1.45 (1.41 – 1.49) |
| Men | 1.06 (1.00 – 1.12) | 1.08 (1.02 – 1.14) | 1.07 (1.01 – 1.13) | 1.06 (1.00 – 1.12) |
| Index of multiple deprivation | | overall p = <0.001 | overall p = <0.001 | overall p = <0.001 |
| Q1 (least deprived) | | Reference | Reference | Reference |
| Q2 | | 1.09 (0.99 – 1.20) | 1.09 (0.99 – 1.20) | 1.08 (0.99 – 1.19) |
| Q3 | | 1.20 (1.09 – 1.33) | 1.20 (1.08 – 1.32) | 1.18 (1.07 – 1.31) |
| Q4 | | 1.20 (1.08 – 1.33) | 1.20 (1.08 – 1.33) | 1.17 (1.06 – 1.30) |
| Q5 (most deprived) | | 1.30 (1.16 – 1.45) | 1.30 (1.16 – 1.45) | 1.27 (1.13 – 1.41) |
| History of hypertension | | 1.19 (1.12 – 1.26) | 1.17 (1.10 – 1.24) | 1.16 (1.09 – 1.23) |
| History of diabetes | | 1.48 (1.38 – 1.60) | 1.49 (1.38 – 1.60) | 1.45 (1.35 – 1.56) |
| History of atrial fibrillation | | | 1.65 (1.53 – 1.77) | 1.62 (1.51 – 1.75) |
| Type of MI<br>STEMI | | | 1.17 (1.10 – 1.26) | 1.19 (1.11 – 1.27) |
| History of peripheral arterial disease | | | | 1.38 (1.26 – 1.51) |
| History of COPD | | | | 1.28 (1.17 – 1.40) |
| Prescribed ACE inhibitor before MI | | | | 1.06 (0.99 – 1.13) |
| Prescribed Angiotensin receptor blocker (ARB) before MI | | | | 1.00 (0.89 – 1.11) |
| Prescribed Beta-blocker before MI | | | | 0.93 (0.88 – 1.00) |

All analyses presented in table 2 were performed on a complete case dataset of 24,479 subjects. HR=hazard ratio; CI=confidence interval; Q=quintile; MI=myocardial infarction; NSTEMI=non-ST-segment-elevation myocardial infarction; STEMI=ST-segment-elevation myocardial infarction; COPD=chronic obstructive pulmonary disease; ACE=Angiotensin-converting enzyme.

For peer review only

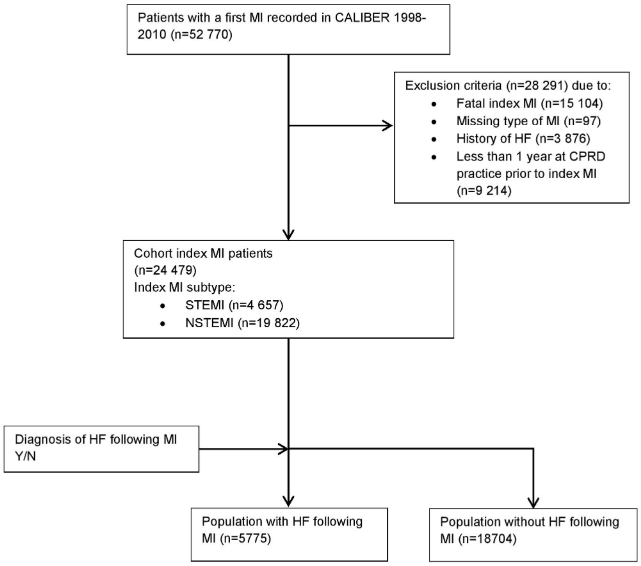


209x297mm (300 x 300 DPI)

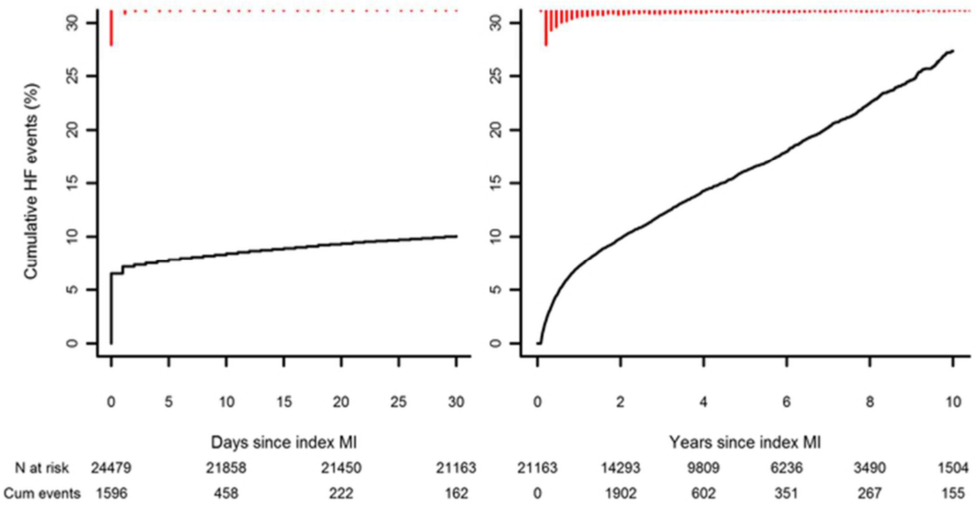


59x35mm (300 x 300 DPI)

review only

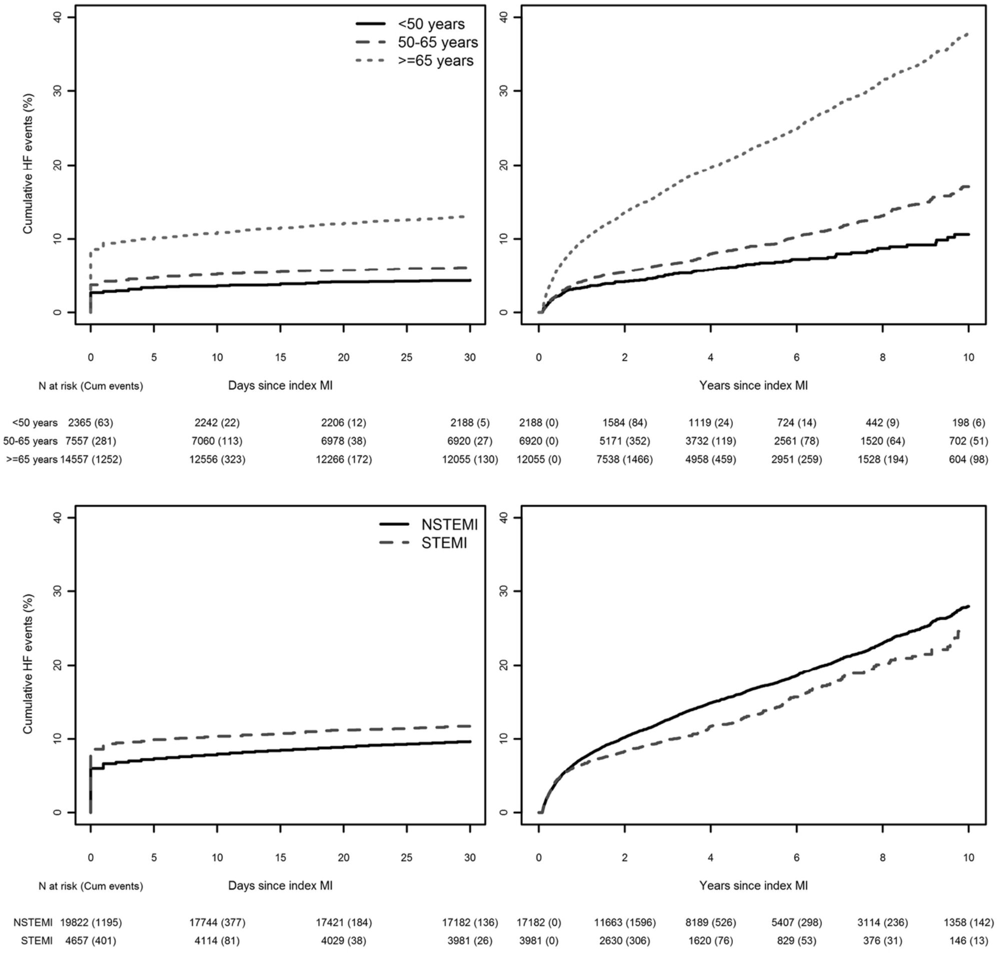


139x139mm (300 x 300 DPI)

**Supplementary Data**

## An Electronic Health Records cohort study on Heart Failure Following Myocardial Infarction in England: Incidence and Predictors

Johannes M.I.H. Gho, Amand F. Schmidt, Laura Pasea, Stefan Koudstaal, Mar Pujades-Rodriguez, Spiros Denaxas, Anoop D. Shah, Riyaz S. Patel, Chris P. Gale, Arno W. Hoes, John G. Cleland, Harry Hemingway, Folkert W. Asselbergs

For peer review only

Phenotype coding lists

**CALIBER myocardial infarction phenotype**
Fatal or non-fatal myocardial infarction (MI) as recorded in any of the constituent CALIBER data sources. The type of myocardial infarction (ST elevation MI, STEMI; or non ST elevation MI, NSTEMI) is recorded only in MINAP and CPRD.

Read codes used to identify myocardial infarction in primary care records (Clinical Research Practice Datalink)

| Category | Clinical code | Clinical term | Datasource lookup |
|---|---|---|---|
| **STEMI (3)** | G30X000 | Acute ST segment elevation myocardial infarction | 12229 |
| **NSTEMI (4)** | G307100 | Acute non-ST segment elevation myocardial infarction | 10562 |
| **Acute MI not further specified (5)** | 323..00 | ECG: myocardial infarction | 7783 |
| **Acute MI not further specified (5)** | 3233.00 | ECG: antero-septal infarct. | 26975 |
| **Acute MI not further specified (5)** | 3234.00 | ECG:posterior/inferior infarct | 26972 |
| **Acute MI not further specified (5)** | 3235.00 | ECG: subendocardial infarct | 55401 |
| **Acute MI not further specified (5)** | 3236.00 | ECG: lateral infarction | 52705 |
| **Acute MI not further specified (5)** | 323Z.00 | ECG: myocardial infarct NOS | 59032 |
| **Acute MI not further specified (5)** | 889A.00 | Diab mellit insulin-glucose infus acute myocardial infarct | 61670 |
| **Acute MI not further specified (5)** | G30..00 | Acute myocardial infarction | 241 |
| **Acute MI not further specified (5)** | G30..12 | Coronary thrombosis | 2491 |
| **Acute MI not further specified (5)** | G30..13 | Cardiac rupture following myocardial infarction (MI) | 30421 |
| **Acute MI not further specified (5)** | G30..15 | MI - acute myocardial infarction | 1677 |
| **Acute MI not further specified (5)** | G30..16 | Thrombosis - coronary | 13571 |
| **Acute MI not further specified (5)** | G300.00 | Acute anterolateral infarction | 12139 |
| **Acute MI not further specified (5)** | G301.00 | Other specified anterior myocardial infarction | 5387 |
| **Acute MI not further specified (5)** | G301000 | Acute anteroapical infarction | 40429 |
| **Acute MI not further specified (5)** | G301100 | Acute anteroseptal infarction | 17872 |
| **Acute MI not further specified (5)** | G301z00 | Anterior myocardial infarction NOS | 14897 |
| **Acute MI not further specified (5)** | G302.00 | Acute inferolateral infarction | 8935 |
| **Acute MI not further specified (5)** | G303.00 | Acute inferoposterior infarction | 29643 |
| **Acute MI not further specified (5)** | G304.00 | Posterior myocardial infarction NOS | 23892 |
| **Acute MI not further specified (5)** | G305.00 | Lateral myocardial infarction NOS | 14898 |
| **Acute MI not further specified (5)** | G306.00 | True posterior myocardial infarction | 63467 |
| **Acute MI not further specified (5)** | G307.00 | Acute subendocardial infarction | 3704 |
| **Acute MI not further specified (5)** | G307000 | Acute non-Q wave infarction | 9507 |

For peer review only

| | | | |
|---|---|---|---|
| **Acute MI not further specified (5)** | G308.00 | Inferior myocardial infarction NOS | 1678 |
| **Acute MI not further specified (5)** | G309.00 | Acute Q-wave infarct | 30330 |
| **Acute MI not further specified (5)** | G30B.00 | Acute posterolateral myocardial infarction | 32854 |
| **Acute MI not further specified (5)** | G30X.00 | Acute transmural myocardial infarction of unspecif site | 29758 |
| **Acute MI not further specified (5)** | G30y.00 | Other acute myocardial infarction | 34803 |
| **Acute MI not further specified (5)** | G30y000 | Acute atrial infarction | 28736 |
| **Acute MI not further specified (5)** | G30y100 | Acute papillary muscle infarction | 62626 |
| **Acute MI not further specified (5)** | G30y200 | Acute septal infarction | 41221 |
| **Acute MI not further specified (5)** | G30yz00 | Other acute myocardial infarction NOS | 46017 |
| **Acute MI not further specified (5)** | G30z.00 | Acute myocardial infarction NOS | 14658 |
| **Acute MI not further specified (5)** | G31y100 | Microinfarction of heart | 68357 |
| **Acute MI not further specified (5)** | G38..00 | Postoperative myocardial infarction | 32272 |
| **Acute MI not further specified (5)** | G380.00 | Postoperative transmural myocardial infarction anterior wall | 46112 |
| **Acute MI not further specified (5)** | G381.00 | Postoperative transmural myocardial infarction inferior wall | 46276 |
| **Acute MI not further specified (5)** | G384.00 | Postoperative subendocardial myocardial infarction | 41835 |
| **Acute MI not further specified (5)** | G38z.00 | Postoperative myocardial infarction, unspecified | 68748 |
| **Acute MI not further specified (5)** | Gyu3400 | [X]Acute transmural myocardial infarction of unspecif site | 96838 |

Hospital Episode Statistics

| **Category (code)** | ICD10 code | ICD10 term |
|---|---|---|
| **Acute MI not further specified (5)** | I21 | Acute myocardial infarction |

OPCS

| **Category (code)** | OPCS 4 | OPCS 4 term |
|---|---|---|
| **Transluminal coronary thrombolysis (2)** | K50.2 | Percutaneous transluminal coronary thrombolysis using streptokinase |
| **Transluminal coronary thrombolysis (2)** | K50.3 | Percutaneous transluminal injection of therapeutic substance into coronary artery NEC |

Office for National Statistics (ONS)

| **Category (code)** | ICD10 code | ICD10 term |
|---|---|---|
| **Death from MI (3)** | I21 | Acute myocardial infarction |
| **Death from MI (3)** | I22 | Subsequent myocardial infarction |
| **Death from MI (3)** | I23 | Certain current complications following acute myocardial infarction |

Office for National Statistics (ONS) prior to 2000 (ICD9)

| **Category (code)** | ICD9 code | ICD9 term |
|---|---|---|
| **Death from myocardial infarction (3)** | 410 | Acute myocardial infarction |
| **Death from myocardial infarction (3)** | 4110 | Other acute and subacute forms of ischemic heart disease ; Postmyocardial infarction syndrome |

For peer review only

| | | |
|---|---|---|
| **Death from myocardial infarction (3)** | 4297 | Ill-defined descriptions and complications of heart disease ; Certain sequelae of myocardial infarction, not elsewhere classified |

Myocardial Ischaemia National Audit Project (MINAP)

1 Acute STEMI

2 Acute NSTEMI

## Phenotype coding lists

**CALIBER myocardial infarction phenotype**

Fatal or non-fatal myocardial infarction (MI) as recorded in any of the constituent CALIBER data sources. The type of myocardial infarction (ST elevation MI, STEMI; or non ST elevation MI, NSTEMI) is recorded only in MINAP and CPRD.

Read codes used to identify myocardial infarction in primary care records (Clinical Research Practice Datalink)

| Category | Clinical code | Clinical term | Datasource lookup |
|---|---|---|---|
| **STEMI (3)** | G30X000 | Acute ST segment elevation myocardial infarction | 12229 |
| **NSTEMI (4)** | G307100 | Acute non-ST segment elevation myocardial infarction | 10562 |
| **Acute MI not further specified (5)** | 323..00 | ECG: myocardial infarction | 7783 |
| **Acute MI not further specified (5)** | 3233.00 | ECG: antero-septal infarct. | 26975 |
| **Acute MI not further specified (5)** | 3234.00 | ECG:posterior/inferior infarct | 26972 |
| **Acute MI not further specified (5)** | 3235.00 | ECG: subendocardial infarct | 55401 |
| **Acute MI not further specified (5)** | 3236.00 | ECG: lateral infarction | 52705 |
| **Acute MI not further specified (5)** | 323Z.00 | ECG: myocardial infarct NOS | 59032 |
| **Acute MI not further specified (5)** | 889A.00 | Diab mellit insulin-glucose infus acute myocardial infarct | 61670 |
| **Acute MI not further specified (5)** | G30..00 | Acute myocardial infarction | 241 |
| **Acute MI not further specified (5)** | G30..12 | Coronary thrombosis | 2491 |
| **Acute MI not further specified (5)** | G30..13 | Cardiac rupture following myocardial infarction (MI) | 30421 |
| **Acute MI not further specified (5)** | G30..15 | MI - acute myocardial infarction | 1677 |
| **Acute MI not further specified (5)** | G30..16 | Thrombosis - coronary | 13571 |
| **Acute MI not further specified (5)** | G300.00 | Acute anterolateral infarction | 12139 |
| **Acute MI not further specified (5)** | G301.00 | Other specified anterior myocardial infarction | 5387 |
| **Acute MI not further specified (5)** | G301000 | Acute anteroapical infarction | 40429 |
| **Acute MI not further specified (5)** | G301100 | Acute anteroseptal infarction | 17872 |
| **Acute MI not further specified (5)** | G301z00 | Anterior myocardial infarction NOS | 14897 |
| **Acute MI not further specified (5)** | G302.00 | Acute inferolateral infarction | 8935 |
| **Acute MI not further specified (5)** | G303.00 | Acute inferoposterior infarction | 29643 |
| **Acute MI not further specified (5)** | G304.00 | Posterior myocardial infarction NOS | 23892 |
| **Acute MI not further specified (5)** | G305.00 | Lateral myocardial infarction NOS | 14898 |

For peer review only

| | | | |
|---|---|---|---|
| **Acute MI not further specified (5)** | G306.00 | True posterior myocardial infarction | 63467 |
| **Acute MI not further specified (5)** | G307.00 | Acute subendocardial infarction | 3704 |
| **Acute MI not further specified (5)** | G307000 | Acute non-Q wave infarction | 9507 |
| **Acute MI not further specified (5)** | G308.00 | Inferior myocardial infarction NOS | 1678 |
| **Acute MI not further specified (5)** | G309.00 | Acute Q-wave infarct | 30330 |
| **Acute MI not further specified (5)** | G30B.00 | Acute posterolateral myocardial infarction | 32854 |
| **Acute MI not further specified (5)** | G30X.00 | Acute transmural myocardial infarction of unspecif site | 29758 |
| **Acute MI not further specified (5)** | G30y.00 | Other acute myocardial infarction | 34803 |
| **Acute MI not further specified (5)** | G30y000 | Acute atrial infarction | 28736 |
| **Acute MI not further specified (5)** | G30y100 | Acute papillary muscle infarction | 62626 |
| **Acute MI not further specified (5)** | G30y200 | Acute septal infarction | 41221 |
| **Acute MI not further specified (5)** | G30yz00 | Other acute myocardial infarction NOS | 46017 |
| **Acute MI not further specified (5)** | G30z.00 | Acute myocardial infarction NOS | 14658 |
| **Acute MI not further specified (5)** | G31y100 | Microinfarction of heart | 68357 |
| **Acute MI not further specified (5)** | G38..00 | Postoperative myocardial infarction | 32272 |
| **Acute MI not further specified (5)** | G380.00 | Postoperative transmural myocardial infarction anterior wall | 46112 |
| **Acute MI not further specified (5)** | G381.00 | Postoperative transmural myocardial infarction inferior wall | 46276 |
| **Acute MI not further specified (5)** | G384.00 | Postoperative subendocardial myocardial infarction | 41835 |
| **Acute MI not further specified (5)** | G38z.00 | Postoperative myocardial infarction, unspecified | 68748 |
| **Acute MI not further specified (5)** | Gyu3400 | [X]Acute transmural myocardial infarction of unspecif site | 96838 |

Hospital Episode Statistics

| **Category (code)** | ICD10 code | ICD10 term |
|---|---|---|
| **Acute MI not further specified (5)** | I21 | Acute myocardial infarction |

OPCS

| **Category (code)** | OPCS 4 | OPCS 4 term |
|---|---|---|
| **Transluminal coronary thrombolysis (2)** | K50.2 | Percutaneous transluminal coronary thrombolysis using streptokinase |
| **Transluminal coronary thrombolysis (2)** | K50.3 | Percutaneous transluminal injection of therapeutic substance into coronary artery NEC |

Office for National Statistics (ONS)

| **Category (code)** | ICD10 code | ICD10 term |
|---|---|---|
| **Death from MI (3)** | I21 | Acute myocardial infarction |
| **Death from MI (3)** | I22 | Subsequent myocardial infarction |
| **Death from MI (3)** | I23 | Certain current complications following acute myocardial infarction |

Office for National Statistics (ONS) prior to 2000 (ICD9)

For peer review only

| Category (code) | ICD9 code | ICD9 term |
|---|---|---|
| **Death from myocardial infarction (3)** | 410 | Acute myocardial infarction |
| **Death from myocardial infarction (3)** | 4110 | Other acute and subacute forms of ischemic heart disease ; Postmyocardial infarction syndrome |
| **Death from myocardial infarction (3)** | 4297 | Ill-defined descriptions and complications of heart disease ; Certain sequelae of myocardial infarction, not elsewhere classified |

Myocardial Ischaemia National Audit Project (MINAP)

| | |
|---|---|
| 1 | Acute STEMI |
| 2 | Acute NSTEMI |

**CALIBER heart failure phenotype**

Primary care
1. Diagnosis of left ventricular dysfunction during echocardiogram during a consultation
2. Diagnosis of heart failure of valvular cause during a consultation
3. Diagnosis of heart failure due to hypertension during a consultation
4. Diagnosis of heart failure due to unspecified cause during a consultation
5. Diagnosis of heart failure not otherwise specified during a consultation

Secondary care
1. Diagnosis of heart failure due to hypertension as the primary diagnosis during a hospitalization
2. Diagnosis of heart failure not otherwise specified as the primary diagnosis during a hospitalization

Mortality
1. Hypertensive heart failure as the underlying cause of death
2. Heart failure not otherwise specified as the underlying cause of death

Read codes used to identify myocardial infarction in primary care records (Clinical Research Practice Datalink)

| Category (code) | Clinical code | Clinical term | Datasource lookup |
|---|---|---|---|
| **Heart failure due to valvular disease (3)** | G580400 | Congestive heart failure due to valvular disease | 94870 |
| **Heart failure due to hypertension (4)** | G210.00 | Malignant hypertensive heart disease | 50157 |
| **Heart failure due to hypertension (4)** | G210000 | Malignant hypertensive heart disease without CCF | 95334 |
| **Heart failure due to hypertension (4)** | G210100 | Malignant hypertensive heart disease with CCF | 72668 |
| **Heart failure due to hypertension (4)** | G211100 | Benign hypertensive heart disease with CCF | 52127 |
| **Heart failure due to hypertension (4)** | G21z100 | Hypertensive heart disease NOS with CCF | 62718 |
| **Heart failure due to hypertension (4)** | G230.00 | Malignant hypertensive heart and renal disease | 67232 |
| **Heart failure due to hypertension (4)** | G232.00 | Hypertensive heart&renal dis wth (congestive) heart failure | 21837 |
| **Heart failure due to hypertension (4)** | G234.00 | Hyperten heart&renal dis+both(congestv)heart and renal fail | 57987 |
| **Heart failure due to other cause (5)** | G1yz100 | Rheumatic left ventricular failure | 22262 |
| **Heart failure NOS (6)** | 1O1..00 | Heart failure confirmed | 9913 |
| **Heart failure NOS (6)** | 662W.00 | Heart failure annual review | 30779 |
| **Heart failure NOS (6)** | 662p.00 | Heart failure 6 month review | 83502 |
| **Heart failure NOS (6)** | 8B29.00 | Cardiac failure therapy | 24503 |
| **Heart failure NOS (6)** | 8H2S.00 | Admit heart failure emergency | 32898 |
| **Heart failure NOS (6)** | 9Or0.00 | Heart failure review completed | 19380 |
| **Heart failure NOS (6)** | G400.00 | Acute cor pulmonale | 8464 |
| **Heart failure NOS (6)** | G41z.11 | Chronic cor pulmonale | 5695 |
| **Heart failure NOS (6)** | G554000 | Congestive cardiomyopathy | 5141 |
| **Heart failure NOS (6)** | G554011 | Congestive obstructive cardiomyopathy | 68766 |
| **Heart failure NOS (6)** | G58..00 | Heart failure | 2062 |
| **Heart failure NOS (6)** | G58..11 | Cardiac failure | 1223 |
| **Heart failure NOS (6)** | G580.00 | Congestive heart failure | 398 |

For peer review only

| | | | |
|---|---|---|---|
| **Heart failure NOS (6)** | G580.11 | Congestive cardiac failure | 2906 |
| **Heart failure NOS (6)** | G580.12 | Right heart failure | 10079 |
| **Heart failure NOS (6)** | G580.13 | Right ventricular failure | 10154 |
| **Heart failure NOS (6)** | G580.14 | Biventricular failure | 9524 |
| **Heart failure NOS (6)** | G580000 | Acute congestive heart failure | 23707 |
| **Heart failure NOS (6)** | G580100 | Chronic congestive heart failure | 32671 |
| **Heart failure NOS (6)** | G580200 | Decompensated cardiac failure | 27884 |
| **Heart failure NOS (6)** | G580300 | Compensated cardiac failure | 11424 |
| **Heart failure NOS (6)** | G581.00 | Left ventricular failure | 884 |
| **Heart failure NOS (6)** | G581.11 | Asthma - cardiac | 23481 |
| **Heart failure NOS (6)** | G581.13 | Impaired left ventricular function | 5942 |
| **Heart failure NOS (6)** | G581000 | Acute left ventricular failure | 5255 |
| **Heart failure NOS (6)** | G582.00 | Acute heart failure | 27964 |
| **Heart failure NOS (6)** | G58z.00 | Heart failure NOS | 4024 |
| **Heart failure NOS (6)** | G58z.12 | Cardiac failure NOS | 17278 |
| **Heart failure NOS (6)** | G5yy900 | Left ventricular systolic dysfunction | 8966 |
| **Heart failure NOS (6)** | G5yyA00 | Left ventricular diastolic dysfunction | 12550 |
| **Heart failure NOS (6)** | R2y1000 | [D]Cardiorespiratory failure | 20324 |
| **Left ventricular dysfunction (3)** | 585f.00 | Echocardiogram shows left ventricular systolic dysfunction | 11284 |
| **Left ventricular dysfunction (3)** | 585g.00 | Echocardiogram shows left ventricular diastolic dysfunction | 11351 |

Hospital Episode Statistics

| Category (code) | ICD10 code | ICD10 term |
|---|---|---|
| **HF - hypertension cause (4)** | I110 | Hypertensive heart disease with (congestive) heart failure |
| **HF - hypertension cause (4)** | I130 | Hypertensive heart and renal disease with (congestive) heart failure |
| **HF - hypertension cause (4)** | I132 | Hypertensive heart and renal disease with both (congestive) heart failure and renal failure |
| **HF - other unspecified cause (5)** | I260 | Pulmonary embolism with mention of acute cor pulmonale |
| **HF - not otherwise specified (6)** | I50 | Heart failure |

Office for National Statistics (ONS)

| Category (code) | ICD10 code | ICD10 term |
|---|---|---|
| **Hypertensive heart failure (4)** | I110 | Hypertensive heart disease with (congestive) heart failure |
| **Hypertensive heart failure (4)** | I130 | Hypertensive heart and renal disease with (congestive) heart failure |
| **Hypertensive heart failure (4)** | I132 | Hypertensive heart and renal disease with both (congestive) heart failure and renal failure |
| **Heart failure with other specified cause (5)** | I260 | Pulmonary embolism with mention of acute cor pulmonale |
| **Heart failure with unspecified cause (6)** | I50 | Heart failure |

Office for National Statistics (ONS) prior to 2000 (ICD9)

| Category (code) | ICD9 code | ICD9 term |
|---|---|---|
| **Heart failure with other specified cause (5)** | 4151 | Acute pulmonary heart disease ; Pulmonary embolism and infarction |
| **Heart failure with unspecified cause (6)** | 428 | Heart failure |

Myocardial Ischaemia National Audit Project (MINAP)

1 CCF

**CALIBER baseline definitions**

See also: https://www.caliberresearch.org

For peer review only

Definitions used for smoking at baseline
CPRD:
**smoking_cessation_gprd**
Prescription of smoking cessation medication in primary care. Currently lookup is performed from using BNF chapter 4.10.2.
**smoking_status_gprd**
An individual record of smoking status in primary care. A patient may have multiple records of smoking status.
HES:
**smoking_status_hes**
Smoking status recorded in HES. Currently there are only ICD-10 codes for smoking, no codes for ex-smoking or non-smoking.

Definitions used for hypertension at baseline
CPRD:
**ht_gprd**
Diagnosis of hypertension
HES:
**ht_hes**
Hypertension diagnosis recorded in secondary care.

Definitions used for diabetes at baseline
CPRD:
**dm_gprd**
Type of diabetes diagnosis at a particular point in time.
**diabdiag_gprd**
GP diagnosis of diabetes mellitus using a specific type I or type II diagnosis in the Read diagnosis chapter (i.e. codes starting with C08, C09, C10E, C10F). Vague terms such as `juvenile onset diabetes` or `insulin dependent diabetes` are not included.
HES:
**dm_hes**
Diagnosis of diabetes in secondary care (HES).

For peer review only

Supplementary Methods

**Ethical approval**

CALIBER was approved by the Lewisham Local Research Ethics Committee (ref: 09/H0810/16 date: 08/04/2009) and the Ethics and Confidentiality Committee (ECC) (ref: ECC 2-06(b)/2009 CALIBER dataset). CALIBER has been registered with the University College London Data Protection Officer (ref: Z6364106/2009/2/26). CALIBER EHR data are anonymised; individual informed consent was not sought from study participants.

**Multiple imputation algorithm**

Multiple imputation was implemented using the 'mice' package for R, to replace missing values in prognostic covariates (body mass index, smoking, systolic and diastolic blood pressure).[1] Imputation models included general practice and 3-year periods from 1998 to 2010 of index myocardial infarction (MI) and:

1. All the baseline covariates as used in the multivariable analysis (age, sex, index of multiple deprivation, hypertension, diabetes, type of MI, body mass index, smoking, systolic blood pressure, diastolic blood pressure);
2. Baseline measurements of covariates not considered in the multivariable analysis (alcohol intake, haemoglobin, white blood cell count, creatinine, alanine aminotransferase (ALAT), total cholesterol, HDL cholesterol);
3. Baseline medications prior to index MI (antiplatelet, oral anticoagulant, statin, ACE inhibitor, angiotensin receptor blocker (ARB), beta-blocker, calcium channel blocker, loop diuretic, aldosterone antagonist, digoxin);
4. Coexisting medical conditions (history of atrial fibrillation, peripheral arterial disease, depression, cancer, chronic obstructive pulmonary disease);
5. The Nelson-Aalen hazard and the event status for the outcomes analysed.

Missing data was multiply imputed and across 20 datasets a (stratified) multivariable Cox regression model was fit and results were combined using Rubin's rules.[2]

References

1. van Buuren S. Multiple imputation of discrete and continuous data by fully conditional specification. Stat Methods Med Res 2007;16:219-42.
2. Carpenter JR, Kenward MG. *Multiple Imputation and its Application*: Wiley; 2013.

For peer review only

**Table S1 - Hazard ratios for heart failure in patients following a first myocardial infarction using the competing risks regression based on Fine and Gray's proportional subhazards model to account for the competing risk of all cause mortality**

| | Model 1 | Model 2 | Model 3 | Model 4 |
|---|---|---|---|---|
| | HR (95% CI) | HR (95% CI) | HR (95% CI) | HR (95% CI) |
| Age, per 10 years increase | 1.41 (1.38 – 1.44) | 1.39 (1.36 – 1.42) | 1.36 (1.33 – 1.39) | 1.35 (1.32 – 1.38) |
| Men | 1.04 (0.99 – 1.10) | 1.07 (1.01 – 1.13) | 1.06 (1.00 – 1.12) | 1.05 (1.00 – 1.11) |
| Index of multiple deprivation | | overall p = <0.001 | overall p = <0.001 | overall p = <0.001 |
| Q1 | | Reference | Reference | Reference |
| Q2 | | 1.11 (1.03 – 1.21) | 1.11 (1.02 – 1.20) | 1.10 (1.01 – 1.20) |
| Q3 | | 1.21 (1.12 – 1.32) | 1.20 (1.11 – 1.31) | 1.19 (1.09 – 1.29) |
| Q4 | | 1.24 (1.14 – 1.35) | 1.24 (1.14 – 1.34) | 1.22 (1.12 – 1.32) |
| Q5 | | 1.36 (1.25 – 1.47) | 1.35 (1.25 – 1.47) | 1.32 (1.21 – 1.43) |
| History of hypertension | | 1.15 (1.09 – 1.22) | 1.13 (1.07 – 1.19) | 1.14 (1.08 – 1.21) |
| History of diabetes | | 1.39 (1.29 – 1.49) | 1.38 (1.29 – 1.48) | 1.36 (1.27 – 1.47) |
| History of atrial fibrillation | | | 1.50 (1.40 – 1.61) | 1.49 (1.39 – 1.60) |
| Type of MI | | | | |
| STEMI | | | 1.10 (1.03 – 1.18) | 1.11 (1.03 – 1.18) |
| History of peripheral arterial disease | | | | 1.29 (1.19 – 1.40) |
| History of COPD | | | | 1.18 (1.09 – 1.29) |
| ACE inhibitor use before MI | | | | 1.01 (0.95 – 1.08) |
| Angiotensin receptor blocker (ARB) use before MI | | | | 0.94 (0.85 – 1.05) |
| Beta-blocker use before MI | | | | 0.94 (0.88 – 1.00) |

Complete case analysis with the addition of clinically relevant variables per model (n = 24,479, n events = 5,775). Overall p-values are shown for categorical covariates. ACE=Angiotensin-converting enzyme; CI=confidence interval; COPD=chronic obstructive pulmonary disease; MI=myocardial infarction; NSTEMI=non-ST-segment-elevation myocardial infarction; STEMI=ST-segment-elevation myocardial infarction; Q=quintile; HR=ratio of the subdistribution hazards.

For peer review only

**Table S2 - Hazard ratios for heart failure in patients following a first myocardial infarction in sensitivity analyses**

| | Model 5<br>Cox regression<br>complete case without multiple imputation<br>n = 4,898<br>n events = 1,159 | Model 5<br>Cox regression<br>with multiple imputation<br>n = 24,479<br>n events = 5,775 | Model 5<br>Competing risk analysis<br>with complete case<br>n = 4,898<br>n events = 1,159 |
|---|---|---|---|
| | HR (95% CI) | HR (95% CI) | HR (95% CI) |
| Age, per 10 years increase | 1.48 (1.38 – 1.60) | 1.47 (1.43 – 1.51) | 1.44 (1.35 – 1.54) |
| Men | 1.03 (0.89 – 1.19) | 1.07 (1.01 – 1.13) | 1.07 (0.95 – 1.22) |
| Body mass index (BMI in kg/m$^2$) | p = 0.005 | p = <0.001 | p = < 0.001 |
| Underweight (18.5 <) | 0.78 (0.48 – 1.32) | 0.76 (0.58 – 0.99) | 0.71 (0.45 – 1.12) |
| Normal (18.5; 25) | Reference | Reference | Reference |
| Overweight (25, 30) | 0.84 (0.71 – 0.99) | 0.84 (0.76 – 0.92) | 0.84 (0.73 – 0.97) |
| Obese (>30) | 1.12 (0.94 – 1.34) | 0.98 (0.90 – 1.08) | 1.17 (1.00 – 1.36) |
| Index of multiple deprivation | p = 0.040 | p < 0.001 | p = < 0.001 |
| Q1 | Reference | Reference | Reference |
| Q2 | 1.22 (0.94 – 1.58) | 1.08 (0.98 – 1.19) | 1.35 (1.10 – 1.66) |
| Q3 | 1.34 (1.03 – 1.75) | 1.17 (1.06 – 1.30) | 1.40 (1.14 – 1.71) |
| Q4 | 1.23 (0.93 – 1.63) | 1.16 (1.05 – 1.29) | 1.31 (1.07 – 1.61) |
| Q5 | 1.45 (1.09 – 1.92) | 1.25 (1.12 – 1.40) | 1.61 (1.33 – 1.96) |
| Smoking status | p = 0.792 | p = 0.012 | p = 0.952 |
| Non-smoker | Reference | Reference | Reference |
| Current smoker | 0.95 (0.77 – 1.14) | 1.14 (1.04 – 1.25) | 1.02 (0.86 – 1. 20) |
| Ex-smoker | 0.98 (0.83 – 1.15) | 1.03 (0.94 – 1.12) | 0.99 (0.87 – 1.14) |
| History of hypertension | 1.14 (0.96 – 1.35) | 1.16 (1.09 – 1.24) | 1.18 (1.02 – 1.37) |
| History of diabetes | 1.28 (1.09 – 1.49) | 1.46 (1.36 – 1.58) | 1.23 (1.08 – 1.39) |
| History of atrial fibrillation | 1.49 (1.25 – 1.76) | 1.63 (1.51 – 1.75) | 1.37 (1.18 – 1.59) |
| Type of MI | | | |
| STEMI | 1.15 (0.97 – 1.37) | 1.18 (1.10 – 1.27) | 1.10 (0.95 – 1.28) |
| History of peripheral arterial disease | 1.53 (1.25 – 1.87) | 1.37 (1.25 – 1.50) | 1.33 (1.13 – 1.57) |
| History of COPD | 1.21 (0.99 – 1.48) | 1.24 (1.15 – 1.38) | 1.22 (1.03 – 1.44) |
| Systolic blood pressure, per 10 mmHg increase | 0.99 (0.95 – 1.03) | 1.00 (0.98 – 1.02) | 1.01 (0.97 – 1.04) |
| Diastolic blood pressure, per 10 mmHg increase | 1.01 (0.94 – 1.09) | 0.99 (0.96 – 1.03) | 0.99 (0.93 – 1.06) |
| ACE inhibitor use before MI | 1.01 (0.87 – 1.17) | 1.06 (0.99 – 1.14) | 1.07 (0.94 – 1.21) |
| Angiotensin receptor blocker (ARB) use before MI | 0.99 (0.95 – 1.03) | 1.00 (0.90 – 1.12) | 0.93 (0.78 – 1.12) |
| Beta-blocker use before MI | 1.01 (0.94 – 1.09) | 0.94 (0.88 – 1.00) | 0.90 (0.79 – 1.02) |

Using multivariable Cox regression without and with multiple imputation and the competing risks regression based on Fine and Gray's proportional subhazards model to account for the competing risk of all cause mortality (n = 4898, n events = 1159). Additional prognostic covariates: body mass index, smoking, systolic and diastolic blood pressure. Overall p-values are shown for categorical covariates. HR=hazard ratio; CI=confidence interval; MI=myocardial infarction; NSTEMI=non-ST-segment-elevation myocardial infarction; STEMI=ST-segment-elevation myocardial infarction; COPD=chronic obstructive pulmonary disease; ACE=Angiotensin-converting enzyme; Q=quintile.

For peer review only

**Table S3 - Hazard ratios for heart failure in patients following a first myocardial infarction stratified on having a previous cancer diagnosis or not.**

| | Model 4<br>**Subjects without a history of cancer**<br>n = 21,719<br>n events = 5,029 | Model 4<br>**Subjects with a history of cancer**<br>n = 2,760<br>n events = 746 |
|---|---|---|
| | HR (95% CI) | HR (95% CI) |
| Age, per 10 years increase | 1.49 (1.45 – 1.53) | 1.45 (1.30 – 1.61) |
| Men | 1.08 (1.01 – 1.15) | 1.13 (0.93 – 1.37) |
| Index of multiple deprivation | overall p = <0.001 | overall p = <0.001 |
| Q1 | Reference | Reference |
| Q2 | 1.08 (0.97 – 1.20) | 1.07 (0.77 – 1.48) |
| Q3 | 1.17 (1.05 – 1.31) | 1.27 (0.91 – 1.78) |
| Q4 | 1.17 (1.04 – 1.31) | 1.14 (0.79 – 1.66) |
| Q5 | 1.25 (1.10 – 1.41) | 1.43 (0.97 – 2.11) |
| History of hypertension | 1.11 (1.03 – 1.18) | 1.45 (1.16 – 1.81) |
| History of diabetes | 1.53 (1.41 – 1.66) | 1.19 (0.91 – 1.55) |
| History of atrial fibrillation | 1.62 (1.49 – 1.76) | 1.67 (1.33 – 2.09) |
| Type of MI | | |
| STEMI | 1.18 (1.09 – 1.28) | 1.01 (0.77 – 1.31) |
| History of peripheral arterial disease | 1.39 (1.26 – 1.54) | 1.19 (0.88 – 1.62) |
| History of COPD | 1.30 (1.18 – 1.43) | 1.36 (1.03 – 1.81) |
| ACE inhibitor use before MI | 1.11 (1.03 – 1.20) | 1.00 (0.79 – 1.26) |
| Angiotensin receptor blocker (ARB) use before MI | 1.06 (0.93 – 1.20) | 0.92 (0.65 – 1.31) |
| Beta-blocker use before MI | 0.97 (0.90 – 1.04) | 0.99 (0.79 – 1.24) |

All analyses are based on a complete case analysis. Overall p-values are shown for categorical covariates. ACE=Angiotensin-converting enzyme; CI=confidence interval; COPD=chronic obstructive pulmonary disease; MI=myocardial infarction; NSTEMI=non-ST-segment-elevation myocardial infarction; STEMI=ST-segment-elevation myocardial infarction; Q=quintile.

For peer review only

**Table S4 - Hazard ratios for heart failure in patients following a first myocardial infarction stratified on a history of revascularization.**

| | Model 4<br>**Subjects without a history of revascularization**<br>n = 21,160<br>n events = 5,222 | Model 4<br>**Subjects with a history of revascularization**<br>n = 3.319<br>n events = 553 |
|---|---|---|
| | HR (95% CI) | HR (95% CI) |
| Age, per 10 years increase | 1.48 (1.44 – 1.52) | 1.30 (1.17 – 1.44) |
| Men | 1.07 (1.01 – 1.14) | 0.96 (0.75 – 1.23) |
| Index of multiple deprivation | overall p = <0.001 | overall p = <0.001 |
| Q1 | Reference | Reference |
| Q2 | 1.06 (0.95 – 1.17) | 1.13 (0.80 – 1.61) |
| Q3 | 1.14 (1.02 – 1.27) | 1.39 (0.95 – 2.03) |
| Q4 | 1.15 (1.02 – 1.28) | 1.34 (0.85 – 2.02) |
| Q5 | 1.22 (1.08 – 1.38) | 1.29 (0.84 – 1.99) |
| History of hypertension | 1.14 (1.07 – 1.22 | 1.18 (0.92 – 1.50) |
| History of diabetes | 1.49 (1.37 – 1.61) | 1.32 (1.00 – 1.75) |
| History of atrial fibrillation | 1.61 (1.48 – 1.74) | 1.90 (1.39 – 2.58) |
| Type of MI | | |
| STEMI | 1.14 (1.05 – 1.23) | 1.39 (1.07 – 1.79) |
| History of peripheral arterial disease | 1.40 (1.27 – 1.55) | 1.22 (0.88 – 1.62) |
| History of COPD | 1.30 (1.18 – 1.43) | 1.36 (0.92 – 2.03) |
| ACE inhibitor use before MI | 1.11 (1.03 – 1.19) | 1.15 (0.89 – 1.49) |
| Angiotensin receptor blocker (ARB) use before MI | 1.06 (0.94 – 1.19) | 0.79 (0.54 – 1.17) |
| Beta-blocker use before MI | 1.00 (0.94 – 1.07) | 0.84 (0.65 – 1.08) |

All analyses are based on a complete case analysis. Overall p-values are shown for categorical covariates. ACE=Angiotensin-converting enzyme; CI=confidence interval; COPD=chronic obstructive pulmonary disease; MI=myocardial infarction; NSTEMI=non-ST-segment-elevation myocardial infarction; STEMI=ST-segment-elevation myocardial infarction; Q=quintile.

For peer review only

**Supplementary Figure 1 – Cumulative incidence curves for HF accounting for mortality as first event after index MI**

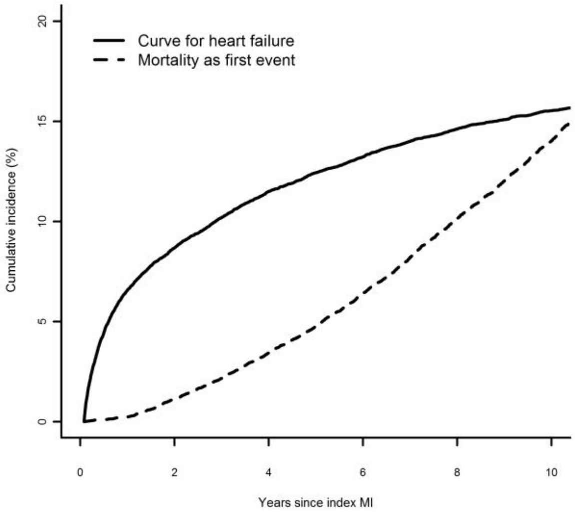


| | | | | | | |
|---|---|---|---|---|---|---|
| N at risk | 21663 | 14293 | 9809 | 6236 | 3489 | 1504 |
| Cum HF events | 0 | 1902 | 602 | 351 | 267 | 155 |
| Cum ACM events | 0 | 292 | 635 | 814 | 990 | 971 |

Cumulative incidence curves adjusted for competing risk of mortality as first event. 10-year follow-up in patients who survived the first 30 days and did not develop HF during the first 30 days (30 days event free).

For peer review only

STROBE Statement—checklist of items that should be included in reports of observational studies

| | Item No | Recommendation |
|---|---|---|
| **Title and abstract** | 1 ✓ | (*a*) ✓*page 2* - Indicate the study's design with a commonly used term in the title or the abstract |
| | | (*b*) ✓*page 2* - Provide in the abstract an informative and balanced summary of what was done and what was found |
| **Introduction** | | |
| Background/rationale | 2✓ | ✓*page 4* - Explain the scientific background and rationale for the investigation being reported |
| Objectives | 3✓ | ✓ *page 4* - State specific objectives, including any prespecified hypotheses |
| **Methods** | | |
| Study design | 4✓ | ✓*pages 4 through 6* - Present key elements of study design early in the paper |
| Setting | 5✓ | Describe the setting, locations, and relevant dates, including periods of recruitment, exposure, follow-up, and data collection |
| Participants | 6✓ | ✓*page 5* - (*a*) *Cohort study*—Give the eligibility criteria, and the sources and methods of selection of participants. Describe methods of follow-up<br>*Case-control study*—Give the eligibility criteria, and the sources and methods of case ascertainment and control selection. Give the rationale for the choice of cases and controls<br>*Cross-sectional study*—Give the eligibility criteria, and the sources and methods of selection of participants |
| | | (*b*) *Cohort study*—For matched studies, give matching criteria and number of exposed and unexposed<br>*Case-control study*—For matched studies, give matching criteria and the number of controls per case |
| Variables | 7✓ | ✓*page 5* - Clearly define all outcomes, exposures, predictors, potential confounders, and effect modifiers. Give diagnostic criteria, if applicable |
| Data sources/ measurement | 8✓* | ✓*page 5* - For each variable of interest, give sources of data and details of methods of assessment (measurement). Describe comparability of assessment methods if there is more than one group |
| Bias | 9✓ | ✓*page 6* - Describe any efforts to address potential sources of bias |
| Study size | 10✓ | ✓*page 7 and figure 1*- Explain how the study size was arrived at |
| Quantitative variables | 11✓ | ✓*page 6* - Explain how quantitative variables were handled in the analyses. If applicable, describe which groupings were chosen and why |
| Statistical methods | 12✓ | (*a*) *NA* Describe all statistical methods, including those used to control for confounding |
| | | (*b*) *NA* Describe any methods used to examine subgroups and interactions |
| | | (*c*) ✓*page 6* - Explain how missing data were addressed |
| | | (*d*) ✓*page 6* - *Cohort study*—If applicable, explain how loss to follow-up was addressed<br>*Case-control study*—If applicable, explain how matching of cases and controls was addressed<br>*Cross-sectional study*—If applicable, describe analytical methods taking account of sampling strategy |
| | | (*e*) ✓Describe any sensitivity analyses |


For peer review only - http://bmjopen.bmj.com/site/about/guidelines.xhtml

Continued on next page

For peer review only


For peer review only - http://bmjopen.bmj.com/site/about/guidelines.xhtml

| | | |
|---|---|---|
| **Results** | | |
| Participants | 13* | ✓*page 6* - (a) Report numbers of individuals at each stage of study—eg numbers potentially eligible, examined for eligibility, confirmed eligible, included in the study, completing follow-up, and analysed |
| | | ✓*page 6 and figure 1* - (b) Give reasons for non-participation at each stage |
| | | ✓*figure 1* - (c) Consider use of a flow diagram |
| Descriptive data | 14*✓ | (a) ✓*table 1* - Give characteristics of study participants (eg demographic, clinical, social) and information on exposures and potential confounders |
| | | (b) ✓*table 1* - Indicate number of participants with missing data for each variable of interest |
| | | (c) ✓*table 1- Cohort study*—Summarise follow-up time (eg, average and total amount) |
| Outcome data | 15* | ✓*table 1, figures 2-3 and pages 7-8 Cohort study*—Report numbers of outcome events or summary measures over time |
| | | *Case-control study*—Report numbers in each exposure category, or summary measures of exposure |
| | | *Cross-sectional study*—Report numbers of outcome events or summary measures |
| Main results | 16 | ✓*table 2, appendix tables* and pages 7-8 - (*a*) Give unadjusted estimates and, if applicable, confounder-adjusted estimates and their precision (eg, 95% confidence interval). Make clear which confounders were adjusted for and why they were included |
| | | *NA* (*b*) Report category boundaries when continuous variables were categorized |
| | | ✓ absolute rates reported in pages 7-8 - (*c*) If relevant, consider translating estimates of relative risk into absolute risk for a meaningful time period |
| Other analyses | 17 | ✓*pages 7-8* - Report other analyses done—eg analyses of subgroups and interactions, and sensitivity analyses |
| **Discussion** | | |
| Key results | 18 | ✓*page 8* Summarise key results with reference to study objectives |
| Limitations | 19 | ✓*pages 9-10* - Discuss limitations of the study, taking into account sources of potential bias or imprecision. Discuss both direction and magnitude of any potential bias |
| Interpretation | 20 | ✓*pages 8-10* Give a cautious overall interpretation of results considering objectives, limitations, multiplicity of analyses, results from similar studies, and other relevant evidence |
| Generalisability | 21 | ✓ *page 9* Discuss the generalisability (external validity) of the study results |
| **Other information** | | |
| Funding | 22 | ✓*page 11* Give the source of funding and the role of the funders for the present study and, if applicable, for the original study on which the present article is based |

*Give information separately for cases and controls in case-control studies and, if applicable, for exposed and unexposed groups in cohort and cross-sectional studies.

**Note:** An Explanation and Elaboration article discusses each checklist item and gives methodological background and published examples of transparent reporting. The STROBE checklist is best used in conjunction with this article (freely available on the Web sites of PLoS Medicine at http://www.plosmedicine.org/, Annals of Internal Medicine at http://www.annals.org/, and Epidemiology at http://www.epidem.com/). Information on the STROBE Initiative is available at www.strobe-statement.org.

For peer review only

BMJ Open

# An Electronic Health Records cohort study on Heart Failure Following Myocardial Infarction in England: Incidence and Predictors



| | |
|---|---|
| Journal: | *BMJ Open* |
| Manuscript ID | bmjopen-2017-018331.R4 |
| Article Type: | Research |
| Date Submitted by the Author: | 03-Nov-2017 |
| Complete List of Authors: | Gho, Johannes; Universitair Medisch Centrum Utrecht, Cardiology; Farr Institute of Health Informatics Research, University College London, Epidemiology and Public Health<br>Schmidt, Amand; Faculty of Science and Engineering, Groningen Research Institute of Pharmacy; University College London, Institute of Cardiovascular Science<br>Pasea, Laura; Farr Institute of Health Informatics Research, University College London, Epidemiology and Public Health<br>Koudstaal, Stefan; Farr Institute of Health Informatics Research, University College London, Epidemiology and Public Health; University Medical Centre Utrecht, Department of Cardiology<br>Pujades Rodriguez, Mar; Farr Institute of Health Informatics Research, University College London, Epidemiology and Public Health; University of Leeds, Medical Research Council Bioinformatics Centre, Leeds Institute of Biomedical and Clinical Sciences<br>Denaxas, S; Farr Institute of Health Informatics Research, University College London, Epidemiology and Public Health<br>Shah, Anoop; UCL, Farr Institute of Health Informatics research<br>Patel, Riyaz; Farr Institute of Health Informatics Research, University College London, Epidemiology and Public Health<br>Gale, Chris; University of Leeds, Medical Research Council Bioinformatics Centre, Leeds Institute for Cardiovascular and Metabolic Medicine<br>Hoes, Arno; Julius Center for Health Sciences and Primary Care, University Medical Center, Utrecht<br>Cleland, J; Imperial College London, National Heart & Lung Institute, Royal Brompton & Harefield Hospitals<br>Hemingway, Harry; Farr Institute of Health Informatics Research, University College London, Epidemiology and Public Health<br>Asselbergs, Folkert; University Medical Centre Utrecht, Department of Cardiology; ICIN-Netherlands Heart Institute, Durrer Center for Cardiogenetic Research |
| <b>Primary Subject Heading</b>: | General practice / Family practice |
| Secondary Subject Heading: | Cardiovascular medicine, Epidemiology, General practice / Family practice, Health informatics, Public health |
| Keywords: | Heart failure < CARDIOLOGY, Myocardial infarction < CARDIOLOGY, electronic health records |

SCHOLARONE™
Manuscripts

For peer review only

# An Electronic Health Records cohort study on Heart Failure Following Myocardial Infarction in England: Incidence and Predictors

Johannes M.I.H. Gho, MD, PhD,[1,2,*] Amand F. Schmidt, PhD,[2,3,4*] Laura Pasea, MSc,[1] Stefan Koudstaal, MD, MSc, PhD,[1,2] Mar Pujades-Rodriguez, PhD,[1,5] Spiros Denaxas, PhD,[1] Anoop D. Shah, MRCP,[1] Riyaz S. Patel, MD, PhD,[1,3] Chris P. Gale, PhD, FRCP,[6] Arno W. Hoes, MD, PhD,[7] John G. Cleland, MD, FRCP,[8] Harry Hemingway, FFPH, FRCP,[1] Folkert W. Asselbergs, MD, PhD,[1,2,3,9]

[1]Farr Institute of Health Informatics Research, UCL Institute of Health Informatics, University College London, London, UK
[2]Department of Cardiology, Division Heart and Lungs, University Medical Center Utrecht, Utrecht, the Netherlands
[3]Institute of Cardiovascular Science, Faculty of Population Health Sciences, University College London, London, UK
[4]Groningen Research Institute of Pharmacy, University of Groningen, Groningen, the Netherlands
[5]Medical Research Council Bioinformatics Centre, Leeds Institute of Biomedical and Clinical Sciences, University of Leeds, Leeds, UK
[6]Medical Research Council Bioinformatics Centre, Leeds Institute for Cardiovascular and Metabolic Medicine, University of Leeds, Leeds, UK
[7]Julius Center for Health Sciences and Primary Care, University Medical Center Utrecht and Utrecht University, Utrecht, the Netherlands
[8]National Heart & Lung Institute, Royal Brompton & Harefield Hospitals, Imperial College London, London, UK
[9]Durrer Center for Cardiogenetic Research, ICIN-Netherlands Heart Institute, Utrecht, the Netherlands
* Shared first author

**Corresponding author:**
A Floriaan Schmidt
Institute of Cardiovascular Science, University College London
222 Euston Road, London NW1 2DA, United Kingdom
E-mail: amand.schmidt@ucl.ac.uk
Tel: 0044 (0)20 3549 5625

**Word count text**: 3627
**Word count abstract**: 242
**Number of references**: 34
**Number of tables**: 2
**Number of figures**: 3
**(Web)appendix**: 1

For peer review only

**Abstract**

**Objectives** To investigate the incidence and determinants of heart failure (HF) following a myocardial infarction (MI) in a contemporary cohort of MI patients using routinely collected primary and hospital care electronic health records.

**Methods** Data were used from the CALIBER programme, linking electronic health records in England from primary care, hospital admissions, an MI registry and mortality data. Subjects were eligible if they were 18 years or older, did not have a history of HF, and survived a first MI. Factors associated with time to HF were examined using Cox proportional hazard models.

**Results** Of the 24,479 MI patients 5,775 (23.6%) developed HF during a median follow-up of 3.7 years (incidence rate per 1000 person-years 63.8 (95% CI: 62.2 – 65.5). Baseline characteristics significantly associated with developing HF were: atrial fibrillation [HR 1.62, 95% CI 1.51 – 1.75], age [per 10 years increase: 1.45, 1.41 – 1.49], diabetes [1.45, 1.35 – 1.56], peripheral arterial disease [1.38, 1.26 – 1.51], COPD [1.28, 1.17 – 1.40], greater socioeconomic deprivation [$5^{th}$ vs. $1^{st}$ quintile 1.27, 1.13 – 1.41], ST-segment elevation MI at presentation [1.19, 1.11 – 1.27], and hypertension [1.16, 1.09 – 1.23]. Results were robust to various sensitivity analyses such as competing risk analysis and multiple imputation.

**Conclusion** In England, one in four survivors of a first MI develop HF within four years. This contemporary study demonstrates MI patients are at considerable risk of HF. Baseline patient characteristics associated with time till HF were identified, which may be used to target preventive strategies.


**Keywords:** heart failure; myocardial infarction; incidence; risk factors; electronic health records

For peer review only



For peer review only - http://bmjopen.bmj.com/site/about/guidelines.xhtml

**Strengths and limitations of this study**

- ❖ This study based on the use of linked electronic health records from general practitioners and hospital records, describes the current burden of heart failure in a representative sample of patients with a first myocardial infarction.
- ❖ The linkage of data from three sources (disease registry, primary care and hospital records) improved diagnostic ascertainment and accuracy in timing of events.
- ❖ Misclassification of drug exposure was likely to be minimal, as prescriptions issued during consultation are automatically recorded.
- ❖ Risk factor adjustment might have been incomplete given that information regarding baseline BMI, smoking and blood pressure was missing for 34 to up to 70% of patients.
- ❖ Stratified methods were used to account for potential calendar and centre effects, and competing risk models were used to adjust for potential competing effects on heart failure and mortality.
- ❖ Due to the high degree of missing data on time to revascularization (88.3%%) we did not explore its relation with HF incidence.

**Abbreviations list:** heart failure (HF); myocardial infarction (MI); ST-segment elevation MI (STEMI); hazard ratio (HR); confidence interval (CI); chronic obstructive pulmonary disease (COPD); body mass index (BMI).



For peer review only - http://bmjopen.bmj.com/site/about/guidelines.xhtml

## Introduction

Research describing the incidence of heart failure (HF) following myocardial infarction (MI) is limited, mainly originating from the thrombolytic era, often using small sample sizes with contradictory findings, making it difficult to provide evidence based medicine. For example, a previous UK study among almost 900 patients hospitalized with MI in 1998, found that one fifth developed HF during their hospital stay and a further third following hospital discharge[1]. More recently, a Swedish study found a 5-year cumulative risk of HF after MI of 21.8% in the calendar period 2004-2013[2]. Further, a Danish nationwide cohort study reported an incidence of HF at 90-days following MI of 19.6% in 2009-10[3]. Differences between these studies could be related to a number of factors, for example change in treatment, national policies, or definitions of heart failure, all of which potentially limit the generalizability of results. We used a large contemporary and representative sample of patients with MI based on electronic health records (EHR) to 1) describe the current incidence of HF after MI in England, using both primary and hospital care data, and 2) explore patient characteristics predictive of post MI HF.

## Methods

### Study design

Data were used from the CALIBER dataset, which included linked data from (i) primary care electronic health records, with diagnoses coded using the Read terminology (Clinical Practice Research Datalink, CPRD) from 226 consenting general practices[4], (ii) hospital care administrative records (Hospital Episode Statistics, HES) and disease registry/audit records (Myocardial Ischaemia National Audit Project, MINAP), and (iii) the mortality register (from UK Office for National Statistics, ONS) [5].

### Setting and participants

Patients were eligible if they were aged ≥18 years, registered in CPRD practices in England consenting to data linkage, with at least one year of up-to-standard pre-study follow-up (meeting CPRD practice quality standards), and experiencing a first MI between 1st January 1998 and 25th March 2010. The first record of



For peer review only - http://bmjopen.bmj.com/site/about/guidelines.xhtml

MI across the linked data sources was considered as the index event and subsequent MI records within 30 days from the same or other sources were considered as representing the same event [6]. Patients with a fatal index MI or a history of HF before their index MI were excluded. Patients were censored if they ceased to be registered at a participating general practice, at the date of death or the administrative censoring of the dataset (25th March 2010).

Variables and data sources

Study variables were derived from diagnoses recorded across several controlled clinical terminology and statistical classification systems: Read, ICD-9 or ICD-10, medication prescription information or the Office of Population Censuses and Surveys Classification of Interventions and Procedures (OPCS-4) codes (see https://rdrr.io/rforge/CALIBERcodelists/man/PRODDICT.html) with the electronic health record phenotyping algorithms published at https://caliberresearch.org/portal [5].

For continuous variables, the most recent measurement recorded in CPRD in the year before study entry was used as the baseline value. Data before study entry were used to determine the prognostic potential of age (years, using the date of birth), sex, ethnicity, social deprivation (Index of Multiple Deprivation, IMD score as recorded in ONS in quintiles), smoking, alcohol use, history of cardiovascular disease, previous coronary revascularization, history of diabetes, history of thyroid disease, history of chronic obstructive pulmonary disease (COPD), history of depression and history of non-metastatic cancer. (see the CALIBER portal https://caliberresearch.org/portal for details). Diabetes and hypertension diagnosis were based on Read codes from primary care or ICD-10 codes from Hospital Episode Statistics, both of which were classified using primary care consultation records and hospitals diagnosis records. Socioeconomic status was based on the Index of Multiple Deprivation which includes seven domains of deprivation (income deprivation, employment deprivation, health deprivation and disability, education, skills and training deprivation, barriers to housing and services, living environment deprivation and crime) [7]. The index MI event was characterized using ECG findings (e.g. ST-segment-elevation myocardial infarction (STEMI), or non-STEMI (NSTEMI)), site of infarction, mode and timing of reperfusion and peak cardiac biomarkers (troponin I, troponin T and CK). MI was defined by linking all of the CALIBER data sources



For peer review only - http://bmjopen.bmj.com/site/about/guidelines.xhtml

(e.g., Hospital Episode Statistics and CPRD), with the type of MI recorded in MINAP and CPRD, see for more detail https://www.caliberresearch.org and the appendix. Characteristics regarding the index MI (e.g., type of revascularization and delay from symptom onset to delivery of reperfusion therapy) were derived from MINAP. This nationwide registry is part of an annual audit which records over 20 key MI variables [8]. Herrett and colleagues [6] validated the current approach and showed the necessity of linking multiple sources to ascertain MI (see appendix for codes used). The primary study outcome was the first HF event following MI, which similar to MI, was recorded in multiple CALIBER data sources; echocardiographic findings and NYHA class were unavailable. All phenotypes were created and validated using a robust methodology described elsewhere [9] and have been used in previously published studies[10,11].

Statistical methods

Incidence rates (cases per 1000 person-years) and Kaplan-Meier cumulative incidence rates of HF were estimated, with Kaplan-Meier curves stratified by age (<50, 50-65, ≥65 years) and type of MI. Association between baseline variables with the onset of HF following MI were explored using Cox proportional hazard models. The proportional hazards assumption was checked using Schoenfeld residuals, and by nonparametric correlation coefficients between survival time and the parameter specific residuals. All models were stratified on general practice and calendar year periods of enrolment (1998-2001, 2001-2004, 2004-2007 and 2007-2010). Stratified models were used instead of frailty models because the former does not make any distributional assumption. Models were sequentially adjusted for: 1) age and sex, 2) cardiovascular risk factors, 3) type of MI, and 4) co-morbidities and prescribed medication. Associations are presented as hazard ratios (HRs) with 95% confidence intervals (CI). Analyses were performed using R (version 3.3.2)[12].

Sensitivity analyses

Due to the availability of EHR from multiple sources, clinical diagnoses and prescriptions were completely recorded. Biomarker and lifestyle measurements such as smoking, BMI, and blood pressure were however, incompletely recorded (see Table 1). It is probable that these data were preferentially recorded


For peer review only - http://bmjopen.bmj.com/site/about/guidelines.xhtml

in subjects perceived to be at a higher risk for early progression of CVD. Pairwise analyses regressing a complete case indicator on observed variables indeed showed considerable dependency between recorded data and missingness (results not shown), violating the “missing completely at random” assumption. This dependency was utilized by multiply imputing missing data using the mice package, which was implemented using 20 imputed datasets and pooled based on Rubin’s rules (Supplementary)[13].

To account for the fact that patients may have died before the onset of HF (e.g., competing risk by mortality), analyses were repeated using Fine and Gray models[14,15]. As a hypothetical example of competing risk, let us assume that out of 100 MI subjects, one develops HF and 99 die. In a Cox’s regression analysis all 99 dead subjects would be censored (implicitly, and incorrectly, assuming they may develop HF later in time), and the hazard of HF would equal 1/1. Instead a Fine and Gray model recognizes the 99 died subjects could never develop HF and hence calculates the hazard as 1/100.

In addition to these sensitivity analyses, we performed a cancer- and revascularization-stratified analysis. Due to discrepancies in sample size between the subgroups we decided against formal interaction testing, which may suffer from inflated false positive rates and lower power in such settings[16].

Ethical approval

CALIBER has received ethics approval (Supplementary). This study is in compliance with the Declaration of Helsinki and was approved by the ISAC (Independent Scientific Advisory Committee) for MHRA Database Research (protocol no. 14_198R) and the MINAP Academic Group and is registered with ClinicalTrials.gov (NCT02384213).

## Results

Subjects and baseline characteristics

For peer review only

In total there were 52,770 patients with an index MI during the study period (Figure 1). Excluding patients with a fatal index MI (n = 15,104), a prior history of HF (n = 3,876), missing information on the type of MI (n = 97) and subjects with less than 1 year of follow-up prior to the indexing MI event (n = 9,214), resulted in an analytical cohort of 24,479 patients. Patients were recruited from 226 GP practices, with the median practice enrolling 101 patients (quartile (Q1) 65 and Q3 150). Of the included subjects 4,657 were STEMI and 19,822 NSTEMI patients (0% missing), 15,969 (65.2%) were men (0% missing), 4,538 (42.1%) currently smoked (56% missing), 12,258 (50.1%) had hypertension (0% missing), and 3,014 (12.3%) had a history of diabetes at baseline (0% missing). In total, 6,129 (25.0%) were prescribed beta-blockers (0% missing) and 5,039 (20.6%) angiotensin converting enzyme (ACE) inhibitors (0% missing) prior to their indexing MI (Table 1). The number of patients identified across the previously defined calendar periods was reasonably stable, with a minimum of 5219 subjects in the period 1998-2001 compared to a maximum of 6838 subjects identified in the period 2004-2007.

Incidence

Patients contributed 90,482 person years of follow-up, during a median follow-up time of 3.7 years (IQR: 1.5; 6.7), 5,775 patients (23.6%) developed HF. The crude incidence rate of HF following a first MI was 63.8 (95% CI: 62.2 – 65.5) per 1000 person-years. Within the first 30 days post MI follow-up 2,438 (10.0%) patients developed HF (Figure 2). From day 30 onwards 3,337 (15.8%) of MI patients (event free during the first 30 days) developed HF, with 6.8% experiencing an HF event within the first year (Figure 2). The incidence of HF during the first 30-days of follow-up was 4.3% (102) in patients younger than 50 years, 6.0% (459) in 50-65 year olds and 12.9% (1877) in those 65 years and older, with HF incidence increasing proportionally as time progressed towards 10 years (Figure 3 top row). The 30-day incidence of HF was 9.5% (1892 events) for NSTEMI subjects and 11.7% (546 events) for STEMI subjects. Excluding patients who experienced a HF within the first 30 days showed that STEMI patients had a lower incidence of HF than NSTEMI subjects (Figure 3 lower panels). At 57 days the crude cumulative risk of HF in the NSTEMI subjects surpassed that of the STEMI subjects for the first time (0.0151 versus 0.0147), with the curves further diverging at 73 days of follow-up (since indexing MI).

For peer review only

Over the entire follow-up 5,921 subjects died, of which 3,538 were free of HF at the time. During the first 30 days of follow-up only 1 patient died, limiting the potential for competing risk by all-cause mortality. In patients who did not have HF after 30 days accounting for competing risk by all-cause mortality attenuated the cumulative risk to be about 15% after 10 years of follow-up (Appendix Figure). Furthermore, the cumulative risks of all-cause mortality and HF converged as follow-up time progressed towards 10 years.

Predictors of HF after multivariable adjustments

Table 2 describes the results of the previously described nested Cox's models, showing little change in estimates between the increasingly complex models. Focusing on the final model, the hazard of HF increased with atrial fibrillation [HR 1.62, 95%CI 1.51 – 1.75], age [per 10 years increase: 1.45, 1.41 – 1.49], diabetes [1.45, 1.35 – 1.56], peripheral arterial disease [1.38, 1.26 – 1.51], COPD [1.28, 1.17 – 1.40], hypertension [1.16, 1.09 – 1.23], higher socioeconomic deprivation (p-value for the 5 groups < 0.001), and STEMI at presentation [1.19, 1.11 – 1.27)]. Accounting for competing risk by all-cause mortality had little impact on the presented results (Appendix Table S1). After multiply imputing the data models were extended to include smoking, BMI, and systolic and diastolic blood pressure variables which showed BMI, male sex and smoking to be conditionally independent prognostic factors (Appendix Table S2). The extended model 5 was implemented using imputed data, and non-imputed (complete case) data, resulting in similar HR estimates (magnitude and direction). Stratifying the sample on cancer diagnosis or history of revascularization showed broadly similar results between subgroups (See appendix table S3 and S4, focusing on the confidence intervals), however precision was low due to the limited number of patients with a history of revascularization or cancer.

**Discussion**

In this large population-based study using linked electronic health records, 23.6% of patients who survived a first MI developed HF during a median follow-up of 3.7 years, resulting in an incidence rate of 64 cases per 1000 person-years. Incident HF was associated with increasing age, higher socioeconomic


For peer review only - http://bmjopen.bmj.com/site/about/guidelines.xhtml

deprivation, a history of diabetes, atrial fibrillation, peripheral arterial disease, COPD, STEMI at presentation, BMI and smoking.

A previous Canadian study, using data from the period 1994-2000[17], found that 71% of elderly patients without HF at index admission developed HF within 5 years' time after an MI, whereas the mortality due to MI decreased in the same period. The Framingham Heart Study[18], using data from 1990 to 1999, found a 5-years post-MI HF incidence of 31.9% after MI; lower than found in the Canadian study. Importantly, both studies showed a higher incidence of post-MI HF than our more contemporary English cohort. This lower HF incidence is likely due to continued improvements of MI treatment strategies, which are reflected in a decreased HF incidence over calendar time. For example, a 20,812 sample of MI patients hospitalized in Western Australia showed that the overall 1-year incidence of HF after MI decreased from 28.1% 1996 in to 16.5% in 2007[19]. This decline is confirmed further by a national Swedish hospital discharge and death registry study reporting a one third decline in incidence between 1993-2004[20].The same Swedish group recently reported that this trend persists for the period 2004-2013[2] and showed improved pharmacological treatment and early revascularization in this period. During a median follow-up of 4 years, 19% of the patients were re-hospitalized because of HF. An explanation for the higher percentage in our study is probably the inclusion of HF diagnosis made in the GP setting next to hospital records decreasing misclassification of HF events. Future efforts are needed to harmonize these national data sources to compare daily care and HF epidemiology across different countries.

Prognostic factors

Similar to the current study, Torabi et al[21] reported that HF after MI increased steeply with age. Socioeconomic deprivation has been shown to be an independent predictor of HF development and associated with an increased incidence of HF in MI-free subjects [22,23]. Socioeconomic deprivation has also been associated with more frequent hospital admission and higher mortality in HF patients[24–26]. The current study extends these observations to HF incidence after an MI. Furthermore, we showed that a history of diabetes, atrial fibrillation, peripheral arterial disease, COPD, STEMI at presentation, BMI and smoking are all independently prognostic of HF after MI. Interestingly, despite the size of the collected

For peer review only

sample, sex only became significantly associated with HF after accounting for differences in BMI and smoking. Furthermore, the impact of male sex (HR of 1.07 95%CI 1.01 – 1.13) was modest indicating relative sex equality. It is of note that blood pressure was not significantly associated with HF. However, blood pressure measurements were frequently missing, which (even after multiple imputation) may be the cause of the observed lack of association hence this deserves independent exploration. In this light it is important to note that the diagnosis of hypertension was associated with HF. Potentially this discrepancies between the association of blood pressure measurement and hypertension diagnosis can be explained by noting that a recorded diagnosis is indicative of long-term hypertensions, which may be different than a single blood pressure measurement in terms of prognosis. Further, given that both variables were included in the same model, the observed difference in association may suggest that conditional on hypertension, blood pressure itself is only modestly associated to HF, if at all.

Strengths and limitations

The linkage of multiple electronic health record sources from primary and hospital care allowed for the collection of a representative sample[27], which enabled us to explore the prognostic value of routinely collected data in primary care records and to detect non-hospitalized HF cases. The population of CPRD practices has been shown broadly representative of the UK population[4,28]. In total 226 GP practices consented to data linkage with Hospital Episode Statistics, MINAP, and the Office for National Statistics (containing 3.9% of the population of England in 2006). A potential limitation is that the ascertainment of cardiovascular outcomes was not based on clinical criteria (e.g., validated questionnaires and properly conducted physical examinations), practices of medical coding will have changed over time and there could be subgroups of patients with left ventricular dysfunction without clinical symptoms. Calendar dependent changes over time were accounted for by using time-period stratified Cox models. We further wish to highlight the infrequent use of PCI in STEMI patients, which was shown to be representative of the slower uptake in England[29]. Similarly, 32% of the patients used antiplatelet drugs at baseline which may be a reflection of non-MI CVD burden. The Kaplan-Meier plots for subtype of MI indicate a possible violation of the proportional hazard assumption for this variable. However, these plots represent the pairwise associations between MI subtype and time to HF, and as such assume that MI subtype is



For peer review only - http://bmjopen.bmj.com/site/about/guidelines.xhtml

For peer review only

independent of other prognostic factors, which is known to be false. The importance of conditioning on covariates is underlined by noting the Kaplan-Meier plots indicating a protective effect of STEMI versus NSTEMI, which was significantly reversed after correcting for covariates (models 3-4). Similarly, instead of using Kaplan-Meier plots to assess the proportional hazard assumption of the *crude* associations, we used Schoenfeld residuals to explore this assumption for the *conditional* associations (multivariable model 4); which did not show any violations. Based on model 4 the absolute correlation between the Schoenfeld residuals and time was < 0.10 (e.g., for MI subtype this was 0.06) indicating an absence of relevant interaction by time (e.g., non-proportionality of hazard). Residual confounding due to medication use (or other missing/mis-specified variables) might be another potential source of bias, however our intention was not to perform a causal analysis between drug prescriptions and time to HF. As such it is interesting to note that despite the large sample size we did not observe a significant association of ACE, ARB or beta-blocker prescribed prior to the indexing MI event and time to HF. Related, we acknowledge we did not assess **all** potential predictors of post MI HF, for example due to the high degree of missingness we did not explore the prognostic potential of time to revascularization[30].

We adhered to CPRD recommendations to obtain up-to-standard baseline data by excluding patients with less than 1 year at CPRD practice prior to index MI. Previously Lewis and colleagues showed that 3 months after registration with a new practice most patient characteristics were updated correctly, which approximated 100% after 1 year of follow-up[31]. Assuming that duration of the CPRD follow-up is independent of the relations assessed here, excluding such patients should not hamper generalizability of results. Using the first MI recorded in the database without a prior history of HF, might have introduced bias due to left truncation (e.g., some subjects may already have experienced an MI before enrolment)[32]. However CALIBER holds longitudinal records from primary and hospital care, making it unlikely a large part of the patients were misclassified as MI free and the one year of follow-up prior to entry further decreases the likelihood of misclassifying patients[31]. While the current data is adequate to identify subjects with a first MI, the sub-classification of MI patients into STEMI and NSTEMI, despite recent improvements, is known to be error prone [33]. As such, results for MI type need replication using higher quality data, in perhaps purposely designed studies. Due to our interest in HF occurrence after a

For peer review only


For peer review only - http://bmjopen.bmj.com/site/about/guidelines.xhtml

first MI selection bias (e.g., index event bias) was introduced[34]. This index event bias does not impact the descriptive or prognostic value of the association presented, and is mostly relevant if one wants to develop an intervention based on the associations presented; which was not the aim of this study. Additionally, we note that we reduced the influence of selection bias by accounting for dependencies between predictors. An important caveat of electronic health care records is that these data are predominantly focussed on recording diagnoses and prospections but not on their complement (i.e., who is **not** diseased or who **did** not receive a drug). As such we have assumed that subjects without a recorded drug prescription or diagnoses was unexposed or free of (that specific) disease. Provided that electronic registration is required for a patient to fill a prescription we can be fairly confident that we did not miss many prescribed treatments. However, it is likely that some subjects were misclassified as free of disease while in fact they were not. We've attempted to minimize this misclassification by linking data across multiple healthcare settings and data sources (MINAP, HES and CPRD, ONS).

A final limitation is that there is a possible delay between primary and hospital care records. Previous research has shown that MI events tend to be recorded in primary care after the HES or MINAP record [6]. The lower 30-day HF incidence in unclassified MI patients primarily derived from primary care could be partly explained because of a delay in coding. Therefore, we showed cumulative incidence rates in patients (alive and HF event free within the first 30 days) from 30 days after index MI to account for a delay in recording of MI in primary care. We were unable to differentiate between HF with preserved ejection fraction (HFpEF) and HF with reduced ejection fraction (HFrEF) as we had no access to detailed (echocardiographic) parameters to assess diastolic dysfunction. It is likely, however that the majority of our HF patients had developed systolic dysfunction after MI.

Conclusion

In this large cohort study using linked electronic health records in England from primary and hospital care, about one in four people developed HF within a median of four years after surviving a first MI. Increasing age, higher socioeconomic deprivation, a history of hypertension, diabetes, atrial fibrillation, peripheral

For peer review only



For peer review only - http://bmjopen.bmj.com/site/about/guidelines.xhtml

arterial disease, COPD, smoking and STEMI at presentation were independent determinants new onset HF following MI.

## Authors' contributions

JMIHG, AFS, LP, SK, MPR, SD, ADS, RSP, CPG, AWH, JGC, HH, FWA contributed to the idea and design of the study. JMIHG extracted and prepared the data for analysis. AFS and JMIHG performed the analysis. JMIHG drafted the manuscript, with revisions from AFS, LP, SK, MPR, SD, ADS, RSP, CPG, AWH, JGC, HH, FWA. JMIHG guarantees the quality and accuracy of the results presented.

## Competing interests

We have read and understood BMJ policy on declaration of interests and declare that we have no competing interests

## Data Sharing Statement

Access to raw data can be requested from the CPRD http://cprd.com

## Funding

This study was supported by the National Institute for Health Research (RP-PG-0407–10314), Wellcome Trust (086091/Z/08/Z), the Medical Research Council Prognosis Research Strategy Partnership (G0902393/99558) and the Farr Institute of Health Informatics Research, funded by The Medical Research Council (MR/K006584/1), in partnership with Arthritis Research UK, the British Heart Foundation, Cancer Research UK, the Economic and Social Research Council, the Engineering and Physical Sciences Research Council, the National Institute of Health Research, the National Institute for Social Care and Health Research (Welsh Assembly Government), the Chief Scientist Office (Scottish Government Health Directorates) and the Wellcome Trust. Part of this work is funded through the Innovative Medicines Initiative 2 Joint Undertaking under grant agreement No 116074, BigData@Heart. AFS is funded by UCLH NIHR Biomedical Research Centre and is a UCL Springboard Population Health Sciences Fellow. HH is an NIHR Senior Investigator, and receives support from the NIHR University College Hospitals / University College London Biomedical Research Centre. FWA is supported by a Dekker scholarship-Junior Staff Member 2014T001 – Netherlands Heart Foundation and UCL Hospitals NIHR Biomedical Research Centre.

For peer review only

## References


1 Torabi A, Cleland JGF, Khan NK, *et al.* The timing of development and subsequent clinical course of heart failure after a myocardial infarction. *Eur Heart J* 2008;**29**:859–70.

2 Desta L, Jernberg T, Spaak J, *et al.* Risk and predictors of readmission for heart failure following a myocardial infarction between 2004 and 2013: A Swedish nationwide observational study. *Int J Cardiol* Published Online First: May 2017.http://linkinghub.elsevier.com/retrieve/pii/S0167527316348677 (accessed 22 Aug 2017).

3 Gjesing A, Gislason GH, Kober L, *et al.* Nationwide trends in development of heart failure and mortality after first-time myocardial infarction 1997-2010: A Danish cohort study. *Eur J Intern Med* 2014;**25**:731–8.

4 Herrett E, Gallagher AM, Bhaskaran K, *et al.* Data Resource Profile: Clinical Practice Research Datalink (CPRD). *Int J Epidemiol* 2015;**44**:827–36.

5 Denaxas SC, George J, Herrett E, *et al.* Data resource profile: Cardiovascular disease research using linked bespoke studies and electronic health records (CALIBER). *Int J Epidemiol* 2012;**41**:1625–38.

6 Herrett E, Shah AD, Boggon R, *et al.* Completeness and diagnostic validity of recording acute myocardial infarction events in primary care, hospital care, disease registry, and national mortality records: cohort study. *BMJ* 2013;**346**:f2350.

7 DCLG. The English Indices of Deprivation 2010. *Neighb Stat Release* 2011;:1–20.http://www.communities.gov.uk/publications/corporate/statistics/indices2010technicalreport

8 Herrett E, Smeeth L, Walker L, *et al.* The Myocardial Ischaemia National Audit Project (MINAP). *Heart* 2010;**96**:1264–7.http://www.ncbi.nlm.nih.gov/pubmed/23177996

9 Morley KI, Wallace J, Denaxas SC, *et al.* Defining Disease Phenotypes Using National Linked Electronic Health Records: A Case Study of Atrial Fibrillation. *PLoS One* 2014;**9**:e110900.

10 Rapsomaniki E, Timmis A, George J, *et al.* Blood pressure and incidence of twelve cardiovascular diseases: Lifetime risks, healthy life-years lost, and age-specific associations in 1·25 million people. *Lancet* 2014;**383**:1899–911. doi:10.1016/S0140-6736(14)60685-1

11 Shah AD, Langenberg C, Rapsomaniki E, *et al.* Type 2 diabetes and incidence of cardiovascular


For peer review only



For peer review only - http://bmjopen.bmj.com/site/about/guidelines.xhtml

diseases: a cohort study in 1·9 million people. *Lancet Diabetes Endocrinol* 2015;**3**:105–13. doi:10.1016/S2213-8587(14)70219-0

12 R Core Team. R: A language and environment for statistical computing. 2017.

13 Marshall A, Altman DG, Holder RL, *et al.* Combining estimates of interest in prognostic modelling studies after multiple imputation: current practice and guidelines. BMC Med. Res. Methodol. 2009;**9**:57-.

14 Satagopan JM, Ben-Porat L, Berwick M, *et al.* A note on competing risks in survival data analysis. *Br J Cancer* 2004;**91**:1229–35.http://www.nature.com/doifinder/10.1038/sj.bjc.6602102

15 Gray B. cmprsk: Subdistribution Analysis of Competing Risks. 2014.http://cran.r-project.org/package=cmprsk

16 Schmidt AF, Groenwold RHH, Knol MJ, *et al.* Exploring interaction effects in small samples increases rates of false-positive and false-negative findings: Results from a systematic review and simulation study. *J Clin Epidemiol* Published Online First: 2014. doi:10.1016/j.jclinepi.2014.02.008

17 Ezekowitz JA, Kaul P, Bakal JA, *et al.* Declining in-hospital mortality and increasing heart failure incidence in elderly patients with first myocardial infarction. *J Am Coll Cardiol* 2009;**53**:13–20.

18 Velagaleti RS, Pencina MJ, Murabito JM, *et al.* Long-term trends in the incidence of heart failure after myocardial infarction. *Circulation* 2008;**118**:2057–62.

19 Hung J, Teng T-HK, Finn J, *et al.* Trends from 1996 to 2007 in incidence and mortality outcomes of heart failure after acute myocardial infarction: a population-based study of 20,812 patients with first acute myocardial infarction in Western Australia. *J Am Heart Assoc* 2013;**2**:e000172.

20 Shafazand M, Rosengren A, Lappas G, *et al.* Decreasing trends in the incidence of heart failure after acute myocardial infarction from 1993-2004: a study of 175,216 patients with a first acute myocardial infarction in Sweden. *Eur J Heart Fail* 2011;**13**:135–41.

21 Torabi A, Cleland JG, Rigby AS, *et al.* Development and course of heart failure after a myocardial infarction in younger and older people. *J Geriatr Cardiol* 2014;**11**:1–12.http://www.pubmedcentral.nih.gov/articlerender.fcgi?artid=3981977&tool=pmcentrez&rendertype=abstract

22 McAlister FA, Murphy NF, Simpson CR, *et al.* Influence of socioeconomic deprivation on the

For peer review only



For peer review only - http://bmjopen.bmj.com/site/about/guidelines.xhtml

primary care burden and treatment of patients with a diagnosis of heart failure in general practice in Scotland: population based study. *BMJ* 2004;**328**:1110.

23 Hawkins NM, Jhund PS, McMurray JJ V, *et al.* Heart failure and socioeconomic status: Accumulating evidence of inequality. *Eur J Heart Fail* 2012;**14**:138–46.

24 Philbin EF, Dec GW, Jenkins PL, *et al.* Socioeconomic status as an independent risk factor for hospital readmission for heart failure. *Am J Cardiol* 2001;**87**:1367–71.

25 Struthers AD, Anderson G, Donnan PT, *et al.* Social deprivation increases cardiac hospitalisations in chronic heart failure independent of disease severity and diuretic non-adherence. *Heart* 2000;**83**:12–6.

26 Pujades-Rodriguez M, Timmis A, Stogiannis D, *et al.* Socioeconomic deprivation and the incidence of 12 cardiovascular diseases in 1.9 million women and men: implications for risk prediction and prevention. *PLoS One* 2014;**9**:e104671.

27 Mann RD, Andrews EB. *Pharmacovigilance: Second Edition*. 2007.

28 Walley T, Mantgani A. The UK General Practice Research Database. *Lancet (London, England)* 1997;**350**:1097–9.

29 Chung SC, Gedeborg R, Nicholas O, *et al.* Acute myocardial infarction: A comparison of short-term survival in national outcome registries in Sweden and the UK. *Lancet* 2014;**383**:1305–12.

30 Stone GW, Selker HP, Thiele H, *et al.* Relationship between Infarct Size and Outcomes Following Primary PCI Patient-Level Analysis from 10 Randomized Trials. *J Am Coll Cardiol* 2016;**67**:1674–83. doi:10.1016/j.jacc.2016.01.069

31 Lewis JD, Bilker WB, Weinstein RB, *et al.* The relationship between time since registration and measured incidence rates in the General Practice Research Database. *Pharmacoepidemiol Drug Saf* 2005;**14**:443–51.

32 Hazelbag CM, Klungel OH, van Staa TP, *et al.* Left truncation results in substantial bias of the relation between time-dependent exposures and adverse events. *Ann Epidemiol* 2015;**25**:590–6.

33 Alexandrescu R, Bottle A, Jarman B, *et al.* Current ICD10 codes are insufficient to clearly distinguish acute myocardial infarction type: a descriptive study. *BMC Health Serv Res* 2013;**13**:468.http://www.ncbi.nlm.nih.gov/pubmed/24195773

For peer review only

479 34 Dahabreh IJ, Kent DM. Index event bias as an explanation for the paradoxes of recurrence risk
480 research. *Jama* 2011;**305**:822–3.

481

For peer review only

**Figure titles and legends**

**Figure 1 – Flowchart of study population**

CALIBER=Cardiovascular disease research using Linked Bespoke studies and electronic health Records; CPRD=Clinical Practice Research Datalink; HF=heart failure; MI=myocardial infarction.

**Figure 2 – Incidence of HF after index MI**

Kaplan-Meier curves for crude incidence of HF after MI. **Left panel** 30-day follow-up after index MI. **Right panel** 10 year follow-up (median follow-up time was 3.7 years) in patients who survived the first 30 days and did not develop HF during the first 30 days (30-days event free). HF=heart failure; MI=myocardial infarction. “N at risk” represent the number of subjects at risk at a certain time point. “Cum events” represents the cumulative number of events since the previous time points. A spike histogram is provided (in red) at the top of the graphs providing information on the number of event across time.

**Figure 3 – Incidence of HF after index MI by group**

Kaplan-Meier curves for crude incidence of HF after MI stratified by group. **Top left panel:** 30-day follow-up after index MI stratified by age group. **Top right panel:** 10-year follow-up (median follow-up time was 3.7 years) in 30-day event free patients stratified by age group. Log-rank test of ≥65 year old patients compared to patients aged 50-65 years ($p<0.001$). **Bottom left panel:** 30-day follow-up after index MI stratified by MI type. **Bottom right panel:** 10-year follow-up (median follow-up time was 3.7 years) in patients who survived the first 30 days and did not develop HF during the first 30 days stratified by MI type. Log-rank test of NSTEMI compared to STEMI ($p<0.001$). HF=heart failure; MI=myocardial infarction. N at risk” represent the number of subjects at risk at a certain time point, with the cumulative number of events since the previous time points presented between brackets.

For peer review only

Table 1 - Baseline characteristics at index myocardial infarction

| | STEMI patients n = 4,657 | NSTEMI patients n = 19,822 | Total MI patients n = 24,479 | Unknown (%) |
|---|---|---|---|---|
| Follow-up time (years), median (IQR) | 3.3 (1.4–5.7) | 3.9 (1.5–7.1) | 3.7 (1.5–6.7) | 0% |
| Mean age, years (SD) | 65.7 (13.2) | 68.7 (13.2) | 68.1 (13.2) | 0% |
| Male sex | 3311 (71.1%) | 12658 (63.9%) | 15969 (65.2%) | 0% |
| Ethnicity | | | | |
| White | 3466 (76.4%) | 14288 (76.1%) | 17754 (76.2%) | 4.8% |
| Asian | 83 (1.8%) | 327 (1.7%) | 410 (1.8%) | |
| Black | 20 (0.4%) | 63 (0.3%) | 83 (0.4%) | |
| Other | 968 (21.3%) | 4085 (21.7%) | 5053 (21.7%) | |
| Body mass index (BMI in kg/m$^2$) | | | | |
| Underweight (18.5 <) | 22 (1.7%) | 120 (2.0%) | 142 (1.9%) | 70.0% |
| Normal (18.5; 25) | 335 (25.5%) | 1661 (27.4%) | 1996 (27.2%) | |
| Overweight (25, 30) | 557 (43.0%) | 2431 (40.2%) | 2988 (40.7%) | |
| Obese (>30) | 381 (29.4%) | 1842 (30.4%) | 2223 (30.2%) | |
| Index of multiple deprivation | | | | |
| Most deprived quintile | 892 (19.2%) | 3985 (20.2%) | 4877 (20%) | 0.4% |
| | | | | |
| **Risk factors before index MI** | | | | |
| Current smoker | 1235 (51.1%) | 3303 (39.5%) | 4538 (42.1%) | 55.9% |
| Excess alcohol consumption | 55 (9.0%) | 219 (8.1%) | 274 (8.3%) | 86.4% |
| History of atrial fibrillation | 358 (7.7%) | 2210 (11.1%) | 2568 (10.5%) | 0% |
| History of hypertension | 2117 (45.5%) | 10141 (51.2%) | 12258 (50.1%) | 0% |
| History of peripheral arterial disease | 236 (5.1%) | 1475 (7.4%) | 1711 (7%) | 0% |
| Previous revascularization | | | | |
| PCI | 700 (15.0%) | 2044 (10.3%) | 2744 (11.2%) | 0% |
| CABG | 126 (2.7%) | 843 (4.3%) | 969 (4.0%) | 0% |
| Previous TIA | 154 (3.3%) | 998 (5.0%) | 1152 (4.7%) | 0% |
| Previous stroke | 80 (1.7%) | 410 (2.1%) | 490 (2.0%) | 0% |
| History of diabetes | 538 (11.6%) | 2476 (12.6%) | 3014 (12.3%) | 0% |
| History of thyroid disease | 237 (5.1%) | 1374 (6.9%) | 1661 (6.6%) | 0% |
| History of COPD | 304 (6.5%) | 1634 (8.2%) | 1938 (7.9%) | 0% |
| History of non-metastatic cancer | 478 (10.3%) | 2282 (11.5%) | 2760 (11.3%) | 0% |
| | | | | |
| **Vital signs before admission** median (IQR) | | | | |
| Systolic blood pressure, mmHg | 140 (130–153) | 140 (130–154) | 140 (130–154) | 34.2% |
| Diastolic blood pressure, mmHg | 80 (74–88) | 80 (71–88) | 80 (72–88) | 34.2% |
| | | | | |
| **Biomarkers,** median (IQR) | | | | |
| Troponin I (maximum) | 18.4 (3.6–50.0) | 2.2 (0.3–10.0) | 3.88 (0.62–21.4) | 88.3% |
| Troponin T (maximum) | 1.8 (0.60–4.8) | 0.4 (0.14–1.1) | 0.65 (0.18–2) | 90.8% |
| CK (maximum) | 721 (222–1631) | 219 (97–648) | 331 (123–1068) | 79.8% |
| | | | | |
| **Biomarkers before index MI** mean (SD) | | | | |
| Haemoglobin, g/dL, | 14.1 (1.72) | 13.6 (1.84) | 13.7 (1.83) | 67.1% |
| White blood cell count | 8.0 (2.94) | 7.9 (2.92) | 7.9 (2.92) | 69.1% |
| Neutrophil count | 5.0 (2.12) | 5.0 (2.40) | 5.0 (2.35) | 71.9% |
| Platelets | 270 (85.2) | 266 (88.0) | 267 (87.5) | 69.1% |
| Erythrocyte sedimentation rate | 19.5 (18.8) | 22.2 (21.6) | 21.8 (21.2) | 89.4% |
| Creatinine, µmol/L | 98.5 (39.1) | 102.5 (47.3) | 102 (46.0) | 58.7% |
| eGFR - CKD-EPI | 70.4 (19.9) | 66.2 (19.9) | 67.0 (20.0) | 60.4% |
| Random glucose concentration, mmol/L | 7.09 (3.53) | 7.26 (3.89) | 7.23 (3.82) | 76.5% |
| Total cholesterol | 5.39 (1.45) | 5.24 (1.28) | 5.27 (1.31) | 63.4% |
| LDL cholesterol | 3.24 (1.12) | 3.08 (1.10) | 3.11 (1.11) | 81.3% |
| | | | | |
| **Revascularization characteristics** | | | | |
| Site of infarction | | | | |
| Anterior | 393 (40.2%) | 255 (30.7%) | 648 (35.9%) | 92.6% |
| Primary PCI | 258 (19.3%) | 73 (3.2%) | 331 (9.2%) | 85.3% |
| Prehospital fibrinolysis | 324 (7.0%) | 94 (0.5%) | 418 (1.7%) | 0% |
| Median (IQR) delay from symptom to reperfusion (min) | 150 (98 – 280) | 160 (101 – 296) | 153 (99 – 285) | 88.3% |
| | | | | |

For peer review only



For peer review only - http://bmjopen.bmj.com/site/about/guidelines.xhtml

| | | | | |
|---|---|---|---|---|
| **Prescribed medication before index MI** | | | | |
| Antiplatelet | 867 (18.6%) | 6979 (35.2%) | 7846 (32.1%) | 0% |
| Oral anticoagulant | 67 (1.4%) | 624 (3.1%) | 691 (2.8%) | 0% |
| Statin | 825 (17.7%) | 5297 (26.7%) | 6122 (25.0%) | 0% |
| ACE inhibitor | 676 (14.5%) | 4363 (22.0%) | 5039 (20.6%) | 0% |
| Angiotensin receptor blocker (ARB) | 220 (4.7%) | 1208 (6.1%) | 1428 (5.8%) | 0% |
| Beta-blocker | 766 (16.4%) | 5363 (27.1%) | 6129 (25.0%) | 0% |
| Calcium channel blocker | 804 (17.3%) | 4621 (23.3%) | 5425 (22.2%) | 0% |
| Loop diuretic | 255 (5.5%) | 2188 (11.0%) | 2443 (10.0%) | 0% |
| Aldosterone antagonist | 24 (0.5%) | 198 (1.0%) | 222 (0.9%) | 0% |
| Digoxin | 53 (1.1%) | 515 (2.6%) | 568 (2.3%) | 0% |

IQR=inter-quartile range; SD=standard deviation; MI=myocardial infarction; STEMI=ST-segment-elevation myocardial infarction; PCI=Percutaneous Coronary Intervention; CABG=Coronary Artery Bypass Grafting; TIA=Transient Ischaemic Attack; COPD=Chronic Obstructive Pulmonary Disease; CK=Creatine kinase; ACE=Angiotensin-converting-enzyme. Prehospital and in-hospital fibrinolysis are not mutually exclusive.

For peer review only

**Table 2 - Hazard ratio for heart failure in patients following a first myocardial infarction using multivariable Cox regression**

| | Model 1<br>n = 24,479<br>n events = 5,775 | Model 2<br>n = 24,479<br>n events = 5,775 | Model 3<br>n = 24,479<br>n events = 5,775 | Model 4<br>n = 24,479<br>n events = 5,775 |
|---|---|---|---|---|
| | HR (95% CI) | HR (95% CI) | HR (95% CI) | HR (95% CI) |
| Age, per 10 years | 1.51 (1.48 – 1.55) | 1.49 (1.46 – 1.53) | 1.46 (1.43 – 1.50) | 1.45 (1.41 – 1.49) |
| Men | 1.06 (1.00 – 1.12) | 1.08 (1.02 – 1.14) | 1.07 (1.01 – 1.13) | 1.06 (1.00 – 1.12) |
| Index of multiple deprivation | | overall p = <0.001 | overall p = <0.001 | overall p = <0.001 |
| Q1 (least deprived) | | Reference | Reference | Reference |
| Q2 | | 1.09 (0.99 – 1.20) | 1.09 (0.99 – 1.20) | 1.08 (0.99 – 1.19) |
| Q3 | | 1.20 (1.09 – 1.33) | 1.20 (1.08 – 1.32) | 1.18 (1.07 – 1.31) |
| Q4 | | 1.20 (1.08 – 1.33) | 1.20 (1.08 – 1.33) | 1.17 (1.06 – 1.30) |
| Q5 (most deprived) | | 1.30 (1.16 – 1.45) | 1.30 (1.16 – 1.45) | 1.27 (1.13 – 1.41) |
| History of hypertension | | 1.19 (1.12 – 1.26) | 1.17 (1.10 – 1.24) | 1.16 (1.09 – 1.23) |
| History of diabetes | | 1.48 (1.38 – 1.60) | 1.49 (1.38 – 1.60) | 1.45 (1.35 – 1.56) |
| History of atrial fibrillation | | | 1.65 (1.53 – 1.77) | 1.62 (1.51 – 1.75) |
| Type of MI<br>STEMI | | | 1.17 (1.10 – 1.26) | 1.19 (1.11 – 1.27) |
| History of peripheral arterial disease | | | | 1.38 (1.26 – 1.51) |
| History of COPD | | | | 1.28 (1.17 – 1.40) |
| Prescribed ACE inhibitor before MI | | | | 1.06 (0.99 – 1.13) |
| Prescribed Angiotensin receptor blocker (ARB) before MI | | | | 1.00 (0.89 – 1.11) |
| Prescribed Beta-blocker before MI | | | | 0.93 (0.88 – 1.00) |

All analyses presented in table 2 were performed on a complete case dataset of 24,479 subjects. HR=hazard ratio; CI=confidence interval; Q=quintile; MI=myocardial infarction; NSTEMI=non-ST-segment-elevation myocardial infarction; STEMI=ST-segment-elevation myocardial infarction; COPD=chronic obstructive pulmonary disease; ACE=Angiotensin-converting enzyme.

For peer review only

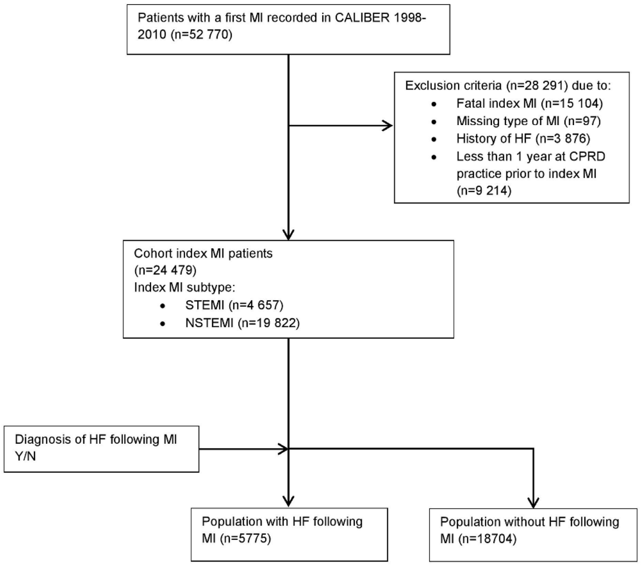


209x297mm (300 x 300 DPI)

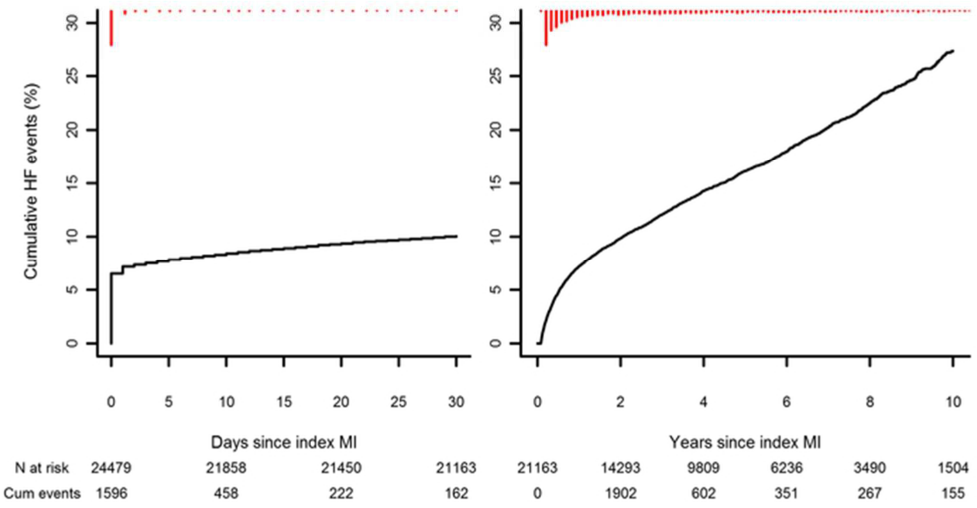


59x35mm (300 x 300 DPI)

review only

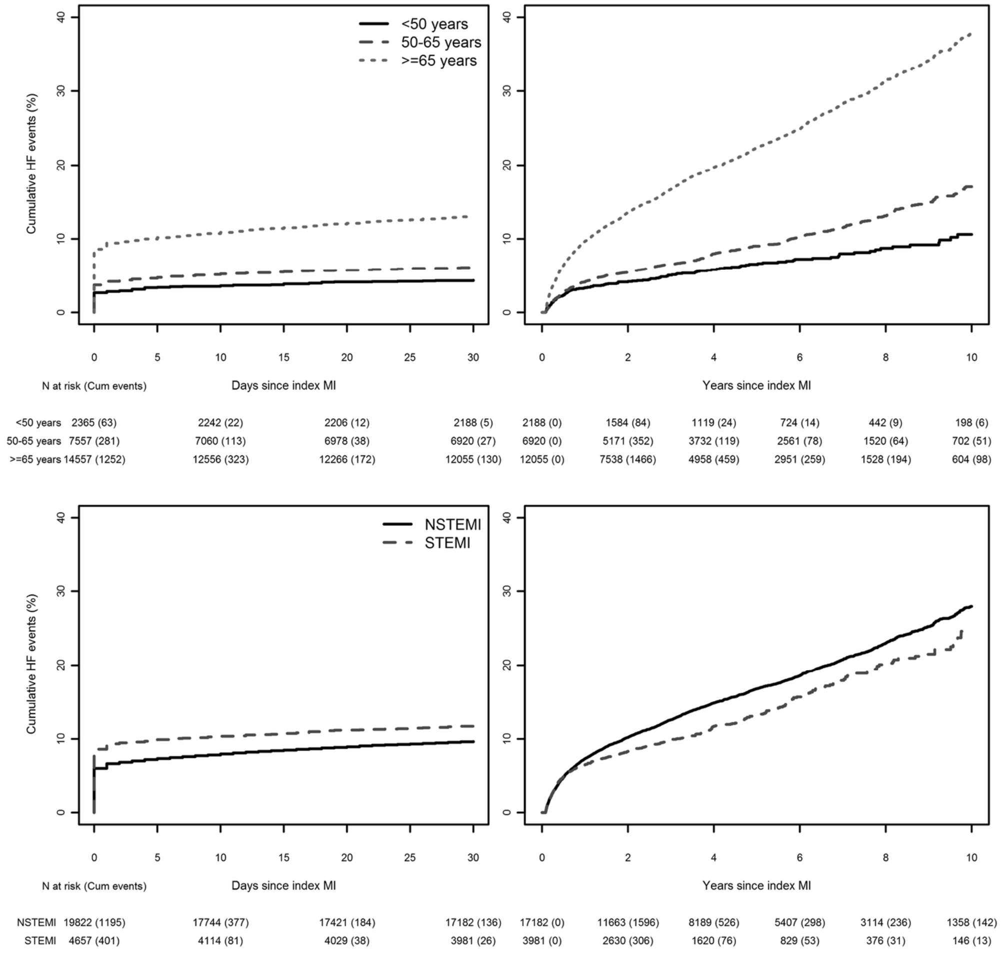


139x139mm (300 x 300 DPI)

**Supplementary Data**

# An Electronic Health Records cohort study on Heart Failure Following Myocardial Infarction in England: Incidence and Predictors

Johannes M.I.H. Gho, Amand F. Schmidt, Laura Pasea, Stefan Koudstaal, Mar Pujades-Rodriguez, Spiros Denaxas, Anoop D. Shah, Riyaz S. Patel, Chris P. Gale, Arno W. Hoes, John G. Cleland, Harry Hemingway, Folkert W. Asselbergs

For peer review only

## Phenotype coding lists

**CALIBER myocardial infarction phenotype**
Fatal or non-fatal myocardial infarction (MI) as recorded in any of the constituent CALIBER data sources. The type of myocardial infarction (ST elevation MI, STEMI; or non ST elevation MI, NSTEMI) is recorded only in MINAP and CPRD.

Read codes used to identify myocardial infarction in primary care records (Clinical Research Practice Datalink)

| Category | Clinical code | Clinical term | Datasource lookup |
|---|---|---|---|
| **STEMI (3)** | G30X000 | Acute ST segment elevation myocardial infarction | 12229 |
| **NSTEMI (4)** | G307100 | Acute non-ST segment elevation myocardial infarction | 10562 |
| **Acute MI not further specified (5)** | 323..00 | ECG: myocardial infarction | 7783 |
| **Acute MI not further specified (5)** | 3233.00 | ECG: antero-septal infarct. | 26975 |
| **Acute MI not further specified (5)** | 3234.00 | ECG:posterior/inferior infarct | 26972 |
| **Acute MI not further specified (5)** | 3235.00 | ECG: subendocardial infarct | 55401 |
| **Acute MI not further specified (5)** | 3236.00 | ECG: lateral infarction | 52705 |
| **Acute MI not further specified (5)** | 323Z.00 | ECG: myocardial infarct NOS | 59032 |
| **Acute MI not further specified (5)** | 889A.00 | Diab mellit insulin-glucose infus acute myocardial infarct | 61670 |
| **Acute MI not further specified (5)** | G30..00 | Acute myocardial infarction | 241 |
| **Acute MI not further specified (5)** | G30..12 | Coronary thrombosis | 2491 |
| **Acute MI not further specified (5)** | G30..13 | Cardiac rupture following myocardial infarction (MI) | 30421 |
| **Acute MI not further specified (5)** | G30..15 | MI - acute myocardial infarction | 1677 |
| **Acute MI not further specified (5)** | G30..16 | Thrombosis - coronary | 13571 |
| **Acute MI not further specified (5)** | G300.00 | Acute anterolateral infarction | 12139 |
| **Acute MI not further specified (5)** | G301.00 | Other specified anterior myocardial infarction | 5387 |
| **Acute MI not further specified (5)** | G301000 | Acute anteroapical infarction | 40429 |
| **Acute MI not further specified (5)** | G301100 | Acute anteroseptal infarction | 17872 |
| **Acute MI not further specified (5)** | G301z00 | Anterior myocardial infarction NOS | 14897 |
| **Acute MI not further specified (5)** | G302.00 | Acute inferolateral infarction | 8935 |
| **Acute MI not further specified (5)** | G303.00 | Acute inferoposterior infarction | 29643 |
| **Acute MI not further specified (5)** | G304.00 | Posterior myocardial infarction NOS | 23892 |
| **Acute MI not further specified (5)** | G305.00 | Lateral myocardial infarction NOS | 14898 |
| **Acute MI not further specified (5)** | G306.00 | True posterior myocardial infarction | 63467 |
| **Acute MI not further specified (5)** | G307.00 | Acute subendocardial infarction | 3704 |
| **Acute MI not further specified (5)** | G307000 | Acute non-Q wave infarction | 9507 |

For peer review only

| | | | |
|---|---|---|---|
| **Acute MI not further specified (5)** | G308.00 | Inferior myocardial infarction NOS | 1678 |
| **Acute MI not further specified (5)** | G309.00 | Acute Q-wave infarct | 30330 |
| **Acute MI not further specified (5)** | G30B.00 | Acute posterolateral myocardial infarction | 32854 |
| **Acute MI not further specified (5)** | G30X.00 | Acute transmural myocardial infarction of unspecif site | 29758 |
| **Acute MI not further specified (5)** | G30y.00 | Other acute myocardial infarction | 34803 |
| **Acute MI not further specified (5)** | G30y000 | Acute atrial infarction | 28736 |
| **Acute MI not further specified (5)** | G30y100 | Acute papillary muscle infarction | 62626 |
| **Acute MI not further specified (5)** | G30y200 | Acute septal infarction | 41221 |
| **Acute MI not further specified (5)** | G30yz00 | Other acute myocardial infarction NOS | 46017 |
| **Acute MI not further specified (5)** | G30z.00 | Acute myocardial infarction NOS | 14658 |
| **Acute MI not further specified (5)** | G31y100 | Microinfarction of heart | 68357 |
| **Acute MI not further specified (5)** | G38..00 | Postoperative myocardial infarction | 32272 |
| **Acute MI not further specified (5)** | G380.00 | Postoperative transmural myocardial infarction anterior wall | 46112 |
| **Acute MI not further specified (5)** | G381.00 | Postoperative transmural myocardial infarction inferior wall | 46276 |
| **Acute MI not further specified (5)** | G384.00 | Postoperative subendocardial myocardial infarction | 41835 |
| **Acute MI not further specified (5)** | G38z.00 | Postoperative myocardial infarction, unspecified | 68748 |
| **Acute MI not further specified (5)** | Gyu3400 | [X]Acute transmural myocardial infarction of unspecif site | 96838 |

Hospital Episode Statistics

| **Category (code)** | ICD10 code | ICD10 term |
|---|---|---|
| **Acute MI not further specified (5)** | I21 | Acute myocardial infarction |

OPCS

| **Category (code)** | OPCS 4 | OPCS 4 term |
|---|---|---|
| **Transluminal coronary thrombolysis (2)** | K50.2 | Percutaneous transluminal coronary thrombolysis using streptokinase |
| **Transluminal coronary thrombolysis (2)** | K50.3 | Percutaneous transluminal injection of therapeutic substance into coronary artery NEC |

Office for National Statistics (ONS)

| **Category (code)** | ICD10 code | ICD10 term |
|---|---|---|
| **Death from MI (3)** | I21 | Acute myocardial infarction |
| **Death from MI (3)** | I22 | Subsequent myocardial infarction |
| **Death from MI (3)** | I23 | Certain current complications following acute myocardial infarction |

Office for National Statistics (ONS) prior to 2000 (ICD9)

| **Category (code)** | ICD9 code | ICD9 term |
|---|---|---|
| **Death from myocardial infarction (3)** | 410 | Acute myocardial infarction |
| **Death from myocardial infarction (3)** | 4110 | Other acute and subacute forms of ischemic heart disease ; Postmyocardial infarction syndrome |

For peer review only

| | | |
|---|---|---|
| **Death from myocardial infarction (3)** | 4297 | Ill-defined descriptions and complications of heart disease ; Certain sequelae of myocardial infarction, not elsewhere classified |

Myocardial Ischaemia National Audit Project (MINAP)

1 Acute STEMI

2 Acute NSTEMI

## Phenotype coding lists

**CALIBER myocardial infarction phenotype**
Fatal or non-fatal myocardial infarction (MI) as recorded in any of the constituent CALIBER data sources. The type of myocardial infarction (ST elevation MI, STEMI; or non ST elevation MI, NSTEMI) is recorded only in MINAP and CPRD.

Read codes used to identify myocardial infarction in primary care records (Clinical Research Practice Datalink)

| Category | Clinical code | Clinical term | Datasource lookup |
|---|---|---|---|
| **STEMI (3)** | G30X000 | Acute ST segment elevation myocardial infarction | 12229 |
| **NSTEMI (4)** | G307100 | Acute non-ST segment elevation myocardial infarction | 10562 |
| **Acute MI not further specified (5)** | 323..00 | ECG: myocardial infarction | 7783 |
| **Acute MI not further specified (5)** | 3233.00 | ECG: antero-septal infarct. | 26975 |
| **Acute MI not further specified (5)** | 3234.00 | ECG:posterior/inferior infarct | 26972 |
| **Acute MI not further specified (5)** | 3235.00 | ECG: subendocardial infarct | 55401 |
| **Acute MI not further specified (5)** | 3236.00 | ECG: lateral infarction | 52705 |
| **Acute MI not further specified (5)** | 323Z.00 | ECG: myocardial infarct NOS | 59032 |
| **Acute MI not further specified (5)** | 889A.00 | Diab mellit insulin-glucose infus acute myocardial infarct | 61670 |
| **Acute MI not further specified (5)** | G30..00 | Acute myocardial infarction | 241 |
| **Acute MI not further specified (5)** | G30..12 | Coronary thrombosis | 2491 |
| **Acute MI not further specified (5)** | G30..13 | Cardiac rupture following myocardial infarction (MI) | 30421 |
| **Acute MI not further specified (5)** | G30..15 | MI - acute myocardial infarction | 1677 |
| **Acute MI not further specified (5)** | G30..16 | Thrombosis - coronary | 13571 |
| **Acute MI not further specified (5)** | G300.00 | Acute anterolateral infarction | 12139 |
| **Acute MI not further specified (5)** | G301.00 | Other specified anterior myocardial infarction | 5387 |
| **Acute MI not further specified (5)** | G301000 | Acute anteroapical infarction | 40429 |
| **Acute MI not further specified (5)** | G301100 | Acute anteroseptal infarction | 17872 |
| **Acute MI not further specified (5)** | G301z00 | Anterior myocardial infarction NOS | 14897 |
| **Acute MI not further specified (5)** | G302.00 | Acute inferolateral infarction | 8935 |
| **Acute MI not further specified (5)** | G303.00 | Acute inferoposterior infarction | 29643 |
| **Acute MI not further specified (5)** | G304.00 | Posterior myocardial infarction NOS | 23892 |
| **Acute MI not further specified (5)** | G305.00 | Lateral myocardial infarction NOS | 14898 |

For peer review only

| | | | |
|---|---|---|---|
| **Acute MI not further specified (5)** | G306.00 | True posterior myocardial infarction | 63467 |
| **Acute MI not further specified (5)** | G307.00 | Acute subendocardial infarction | 3704 |
| **Acute MI not further specified (5)** | G307000 | Acute non-Q wave infarction | 9507 |
| **Acute MI not further specified (5)** | G308.00 | Inferior myocardial infarction NOS | 1678 |
| **Acute MI not further specified (5)** | G309.00 | Acute Q-wave infarct | 30330 |
| **Acute MI not further specified (5)** | G30B.00 | Acute posterolateral myocardial infarction | 32854 |
| **Acute MI not further specified (5)** | G30X.00 | Acute transmural myocardial infarction of unspecif site | 29758 |
| **Acute MI not further specified (5)** | G30y.00 | Other acute myocardial infarction | 34803 |
| **Acute MI not further specified (5)** | G30y000 | Acute atrial infarction | 28736 |
| **Acute MI not further specified (5)** | G30y100 | Acute papillary muscle infarction | 62626 |
| **Acute MI not further specified (5)** | G30y200 | Acute septal infarction | 41221 |
| **Acute MI not further specified (5)** | G30yz00 | Other acute myocardial infarction NOS | 46017 |
| **Acute MI not further specified (5)** | G30z.00 | Acute myocardial infarction NOS | 14658 |
| **Acute MI not further specified (5)** | G31y100 | Microinfarction of heart | 68357 |
| **Acute MI not further specified (5)** | G38..00 | Postoperative myocardial infarction | 32272 |
| **Acute MI not further specified (5)** | G380.00 | Postoperative transmural myocardial infarction anterior wall | 46112 |
| **Acute MI not further specified (5)** | G381.00 | Postoperative transmural myocardial infarction inferior wall | 46276 |
| **Acute MI not further specified (5)** | G384.00 | Postoperative subendocardial myocardial infarction | 41835 |
| **Acute MI not further specified (5)** | G38z.00 | Postoperative myocardial infarction, unspecified | 68748 |
| **Acute MI not further specified (5)** | Gyu3400 | [X]Acute transmural myocardial infarction of unspecif site | 96838 |

Hospital Episode Statistics

| **Category (code)** | ICD10 code | ICD10 term |
|---|---|---|
| **Acute MI not further specified (5)** | I21 | Acute myocardial infarction |

OPCS

| **Category (code)** | OPCS 4 | OPCS 4 term |
|---|---|---|
| **Transluminal coronary thrombolysis (2)** | K50.2 | Percutaneous transluminal coronary thrombolysis using streptokinase |
| **Transluminal coronary thrombolysis (2)** | K50.3 | Percutaneous transluminal injection of therapeutic substance into coronary artery NEC |

Office for National Statistics (ONS)

| **Category (code)** | ICD10 code | ICD10 term |
|---|---|---|
| **Death from MI (3)** | I21 | Acute myocardial infarction |
| **Death from MI (3)** | I22 | Subsequent myocardial infarction |
| **Death from MI (3)** | I23 | Certain current complications following acute myocardial infarction |

Office for National Statistics (ONS) prior to 2000 (ICD9)

For peer review only

| Category (code) | ICD9 code | ICD9 term |
|---|---|---|
| **Death from myocardial infarction (3)** | 410 | Acute myocardial infarction |
| **Death from myocardial infarction (3)** | 4110 | Other acute and subacute forms of ischemic heart disease ; Postmyocardial infarction syndrome |
| **Death from myocardial infarction (3)** | 4297 | Ill-defined descriptions and complications of heart disease ; Certain sequelae of myocardial infarction, not elsewhere classified |

Myocardial Ischaemia National Audit Project (MINAP)

1 Acute STEMI

2 Acute NSTEMI

**CALIBER heart failure phenotype**

Primary care
1. Diagnosis of left ventricular dysfunction during echocardiogram during a consultation
2. Diagnosis of heart failure of valvular cause during a consultation
3. Diagnosis of heart failure due to hypertension during a consultation
4. Diagnosis of heart failure due to unspecified cause during a consultation
5. Diagnosis of heart failure not otherwise specified during a consultation

Secondary care
1. Diagnosis of heart failure due to hypertension as the primary diagnosis during a hospitalization
2. Diagnosis of heart failure not otherwise specified as the primary diagnosis during a hospitalization

Mortality
1. Hypertensive heart failure as the underlying cause of death
2. Heart failure not otherwise specified as the underlying cause of death

Read codes used to identify myocardial infarction in primary care records (Clinical Research Practice Datalink)

| Category (code) | Clinical code | Clinical term | Datasource lookup |
|---|---|---|---|
| **Heart failure due to valvular disease (3)** | G580400 | Congestive heart failure due to valvular disease | 94870 |
| **Heart failure due to hypertension (4)** | G210.00 | Malignant hypertensive heart disease | 50157 |
| **Heart failure due to hypertension (4)** | G210000 | Malignant hypertensive heart disease without CCF | 95334 |
| **Heart failure due to hypertension (4)** | G210100 | Malignant hypertensive heart disease with CCF | 72668 |
| **Heart failure due to hypertension (4)** | G211100 | Benign hypertensive heart disease with CCF | 52127 |
| **Heart failure due to hypertension (4)** | G21z100 | Hypertensive heart disease NOS with CCF | 62718 |
| **Heart failure due to hypertension (4)** | G230.00 | Malignant hypertensive heart and renal disease | 67232 |
| **Heart failure due to hypertension (4)** | G232.00 | Hypertensive heart&renal dis wth (congestive) heart failure | 21837 |
| **Heart failure due to hypertension (4)** | G234.00 | Hyperten heart&renal dis+both(congestv)heart and renal fail | 57987 |
| **Heart failure due to other cause (5)** | G1yz100 | Rheumatic left ventricular failure | 22262 |
| **Heart failure NOS (6)** | 1O1..00 | Heart failure confirmed | 9913 |
| **Heart failure NOS (6)** | 662W.00 | Heart failure annual review | 30779 |
| **Heart failure NOS (6)** | 662p.00 | Heart failure 6 month review | 83502 |
| **Heart failure NOS (6)** | 8B29.00 | Cardiac failure therapy | 24503 |
| **Heart failure NOS (6)** | 8H2S.00 | Admit heart failure emergency | 32898 |
| **Heart failure NOS (6)** | 9Or0.00 | Heart failure review completed | 19380 |
| **Heart failure NOS (6)** | G400.00 | Acute cor pulmonale | 8464 |
| **Heart failure NOS (6)** | G41z.11 | Chronic cor pulmonale | 5695 |
| **Heart failure NOS (6)** | G554000 | Congestive cardiomyopathy | 5141 |
| **Heart failure NOS (6)** | G554011 | Congestive obstructive cardiomyopathy | 68766 |
| **Heart failure NOS (6)** | G58..00 | Heart failure | 2062 |
| **Heart failure NOS (6)** | G58..11 | Cardiac failure | 1223 |
| **Heart failure NOS (6)** | G580.00 | Congestive heart failure | 398 |

For peer review only

| | | | |
|---|---|---|---|
| **Heart failure NOS (6)** | G580.11 | Congestive cardiac failure | 2906 |
| **Heart failure NOS (6)** | G580.12 | Right heart failure | 10079 |
| **Heart failure NOS (6)** | G580.13 | Right ventricular failure | 10154 |
| **Heart failure NOS (6)** | G580.14 | Biventricular failure | 9524 |
| **Heart failure NOS (6)** | G580000 | Acute congestive heart failure | 23707 |
| **Heart failure NOS (6)** | G580100 | Chronic congestive heart failure | 32671 |
| **Heart failure NOS (6)** | G580200 | Decompensated cardiac failure | 27884 |
| **Heart failure NOS (6)** | G580300 | Compensated cardiac failure | 11424 |
| **Heart failure NOS (6)** | G581.00 | Left ventricular failure | 884 |
| **Heart failure NOS (6)** | G581.11 | Asthma - cardiac | 23481 |
| **Heart failure NOS (6)** | G581.13 | Impaired left ventricular function | 5942 |
| **Heart failure NOS (6)** | G581000 | Acute left ventricular failure | 5255 |
| **Heart failure NOS (6)** | G582.00 | Acute heart failure | 27964 |
| **Heart failure NOS (6)** | G58z.00 | Heart failure NOS | 4024 |
| **Heart failure NOS (6)** | G58z.12 | Cardiac failure NOS | 17278 |
| **Heart failure NOS (6)** | G5yy900 | Left ventricular systolic dysfunction | 8966 |
| **Heart failure NOS (6)** | G5yyA00 | Left ventricular diastolic dysfunction | 12550 |
| **Heart failure NOS (6)** | R2y1000 | [D]Cardiorespiratory failure | 20324 |
| **Left ventricular dysfunction (3)** | 585f.00 | Echocardiogram shows left ventricular systolic dysfunction | 11284 |
| **Left ventricular dysfunction (3)** | 585g.00 | Echocardiogram shows left ventricular diastolic dysfunction | 11351 |

Hospital Episode Statistics

| Category (code) | ICD10 code | ICD10 term |
|---|---|---|
| **HF - hypertension cause (4)** | I110 | Hypertensive heart disease with (congestive) heart failure |
| **HF - hypertension cause (4)** | I130 | Hypertensive heart and renal disease with (congestive) heart failure |
| **HF - hypertension cause (4)** | I132 | Hypertensive heart and renal disease with both (congestive) heart failure and renal failure |
| **HF - other unspecified cause (5)** | I260 | Pulmonary embolism with mention of acute cor pulmonale |
| **HF - not otherwise specified (6)** | I50 | Heart failure |

Office for National Statistics (ONS)

| Category (code) | ICD10 code | ICD10 term |
|---|---|---|
| **Hypertensive heart failure (4)** | I110 | Hypertensive heart disease with (congestive) heart failure |
| **Hypertensive heart failure (4)** | I130 | Hypertensive heart and renal disease with (congestive) heart failure |
| **Hypertensive heart failure (4)** | I132 | Hypertensive heart and renal disease with both (congestive) heart failure and renal failure |
| **Heart failure with other specified cause (5)** | I260 | Pulmonary embolism with mention of acute cor pulmonale |
| **Heart failure with unspecified cause (6)** | I50 | Heart failure |

Office for National Statistics (ONS) prior to 2000 (ICD9)

| Category (code) | ICD9 code | ICD9 term |
|---|---|---|
| **Heart failure with other specified cause (5)** | 4151 | Acute pulmonary heart disease ; Pulmonary embolism and infarction |
| **Heart failure with unspecified cause (6)** | 428 | Heart failure |

Myocardial Ischaemia National Audit Project (MINAP)

| | |
|---|---|
| 1 | CCF |

**CALIBER baseline definitions**
See also: https://www.caliberresearch.org

For peer review only

Definitions used for smoking at baseline
CPRD:
**smoking_cessation_gprd**
Prescription of smoking cessation medication in primary care. Currently lookup is performed from using BNF chapter 4.10.2.
**smoking_status_gprd**
An individual record of smoking status in primary care. A patient may have multiple records of smoking status.
HES:
**smoking_status_hes**
Smoking status recorded in HES. Currently there are only ICD-10 codes for smoking, no codes for ex-smoking or non-smoking.

Definitions used for hypertension at baseline
CPRD:
**ht_gprd**
Diagnosis of hypertension
HES:
**ht_hes**
Hypertension diagnosis recorded in secondary care.

Definitions used for diabetes at baseline
CPRD:
**dm_gprd**
Type of diabetes diagnosis at a particular point in time.
**diabdiag_gprd**
GP diagnosis of diabetes mellitus using a specific type I or type II diagnosis in the Read diagnosis chapter (i.e. codes starting with C08, C09, C10E, C10F). Vague terms such as `juvenile onset diabetes` or `insulin dependent diabetes` are not included.
HES:
**dm_hes**
Diagnosis of diabetes in secondary care (HES).

For peer review only

Supplementary Methods

**Ethical approval**
CALIBER was approved by the Lewisham Local Research Ethics Committee (ref: 09/H0810/16 date: 08/04/2009) and the Ethics and Confidentiality Committee (ECC) (ref: ECC 2-06(b)/2009 CALIBER dataset). CALIBER has been registered with the University College London Data Protection Officer (ref: Z6364106/2009/2/26). CALIBER EHR data are anonymised; individual informed consent was not sought from study participants.

**Multiple imputation algorithm**
Multiple imputation was implemented using the 'mice' package for R, to replace missing values in prognostic covariates (body mass index, smoking, systolic and diastolic blood pressure).[1] Imputation models included general practice and 3-year periods from 1998 to 2010 of index myocardial infarction (MI) and:

1. All the baseline covariates as used in the multivariable analysis (age, sex, index of multiple deprivation, hypertension, diabetes, type of MI, body mass index, smoking, systolic blood pressure, diastolic blood pressure);
2. Baseline measurements of covariates not considered in the multivariable analysis (alcohol intake, haemoglobin, white blood cell count, creatinine, alanine aminotransferase (ALAT), total cholesterol, HDL cholesterol);
3. Baseline medications prior to index MI (antiplatelet, oral anticoagulant, statin, ACE inhibitor, angiotensin receptor blocker (ARB), beta-blocker, calcium channel blocker, loop diuretic, aldosterone antagonist, digoxin);
4. Coexisting medical conditions (history of atrial fibrillation, peripheral arterial disease, depression, cancer, chronic obstructive pulmonary disease);
5. The Nelson-Aalen hazard and the event status for the outcomes analysed.

Missing data was multiply imputed and across 20 datasets a (stratified) multivariable Cox regression model was fit and results were combined using Rubin's rules.[2]

References

1. van Buuren S. Multiple imputation of discrete and continuous data by fully conditional specification. Stat Methods Med Res 2007;16:219-42.
2. Carpenter JR, Kenward MG. *Multiple Imputation and its Application*: Wiley; 2013.

For peer review only

**Table S1 - Hazard ratios for heart failure in patients following a first myocardial infarction using the competing risks regression based on Fine and Gray's proportional subhazards model to account for the competing risk of all cause mortality**

| | Model 1 | Model 2 | Model 3 | Model 4 |
|---|---|---|---|---|
| | HR (95% CI) | HR (95% CI) | HR (95% CI) | HR (95% CI) |
| Age, per 10 years increase | 1.41 (1.38 – 1.44) | 1.39 (1.36 – 1.42) | 1.36 (1.33 – 1.39) | 1.35 (1.32 – 1.38) |
| Men | 1.04 (0.99 – 1.10) | 1.07 (1.01 – 1.13) | 1.06 (1.00 – 1.12) | 1.05 (1.00 – 1.11) |
| Index of multiple deprivation | | overall p = <0.001 | overall p = <0.001 | overall p = <0.001 |
| Q1 | | Reference | Reference | Reference |
| Q2 | | 1.11 (1.03 – 1.21) | 1.11 (1.02 – 1.20) | 1.10 (1.01 – 1.20) |
| Q3 | | 1.21 (1.12 – 1.32) | 1.20 (1.11 – 1.31) | 1.19 (1.09 – 1.29) |
| Q4 | | 1.24 (1.14 – 1.35) | 1.24 (1.14 – 1.34) | 1.22 (1.12 – 1.32) |
| Q5 | | 1.36 (1.25 – 1.47) | 1.35 (1.25 – 1.47) | 1.32 (1.21 – 1.43) |
| History of hypertension | | 1.15 (1.09 – 1.22) | 1.13 (1.07 – 1.19) | 1.14 (1.08 – 1.21) |
| History of diabetes | | 1.39 (1.29 – 1.49) | 1.38 (1.29 – 1.48) | 1.36 (1.27 – 1.47) |
| History of atrial fibrillation | | | 1.50 (1.40 – 1.61) | 1.49 (1.39 – 1.60) |
| Type of MI | | | | |
| STEMI | | | 1.10 (1.03 – 1.18) | 1.11 (1.03 – 1.18) |
| History of peripheral arterial disease | | | | 1.29 (1.19 – 1.40) |
| History of COPD | | | | 1.18 (1.09 – 1.29) |
| ACE inhibitor use before MI | | | | 1.01 (0.95 – 1.08) |
| Angiotensin receptor blocker (ARB) use before MI | | | | 0.94 (0.85 – 1.05) |
| Beta-blocker use before MI | | | | 0.94 (0.88 – 1.00) |

Complete case analysis with the addition of clinically relevant variables per model (n = 24,479, n events = 5,775). Overall p-values are shown for categorical covariates. ACE=Angiotensin-converting enzyme; CI=confidence interval; COPD=chronic obstructive pulmonary disease; MI=myocardial infarction; NSTEMI=non-ST-segment-elevation myocardial infarction; STEMI=ST-segment-elevation myocardial infarction; Q=quintile; HR=ratio of the subdistribution hazards.

For peer review only

**Table S2 - Hazard ratios for heart failure in patients following a first myocardial infarction in sensitivity analyses**

| | Model 5<br>Cox regression<br>complete case without<br>multiple imputation<br>n = 4,898<br>n events = 1,159 | Model 5<br>Cox regression<br>with multiple imputation<br>n = 24,479<br>n events = 5,775 | Model 5<br>Competing risk analysis<br>with complete case<br>n = 4,898<br>n events = 1,159 |
|---|---|---|---|
| | HR (95% CI) | HR (95% CI) | HR (95% CI) |
| Age, per 10 years increase | 1.48 (1.38 – 1.60) | 1.47 (1.43 – 1.51) | 1.44 (1.35 – 1.54) |
| Men | 1.03 (0.89 – 1.19) | 1.07 (1.01 – 1.13) | 1.07 (0.95 – 1.22) |
| Body mass index (BMI in kg/m$^2$) | p = 0.005 | p = <0.001 | p = < 0.001 |
| Underweight (18.5 <) | 0.78 (0.48 – 1.32) | 0.76 (0.58 – 0.99) | 0.71 (0.45 – 1.12) |
| Normal (18.5; 25) | Reference | Reference | Reference |
| Overweight (25, 30) | 0.84 (0.71 – 0.99) | 0.84 (0.76 – 0.92) | 0.84 (0.73 – 0.97) |
| Obese (>30) | 1.12 (0.94 – 1.34) | 0.98 (0.90 – 1.08) | 1.17 (1.00 – 1.36) |
| Index of multiple deprivation | p = 0.040 | p < 0.001 | p = < 0.001 |
| Q1 | Reference | Reference | Reference |
| Q2 | 1.22 (0.94 – 1.58) | 1.08 (0.98 – 1.19) | 1.35 (1.10 – 1.66) |
| Q3 | 1.34 (1.03 – 1.75) | 1.17 (1.06 – 1.30) | 1.40 (1.14 – 1.71) |
| Q4 | 1.23 (0.93 – 1.63) | 1.16 (1.05 – 1.29) | 1.31 (1.07 – 1.61) |
| Q5 | 1.45 (1.09 – 1.92) | 1.25 (1.12 – 1.40) | 1.61 (1.33 – 1.96) |
| Smoking status | p = 0.792 | p = 0.012 | p = 0.952 |
| Non-smoker | Reference | Reference | Reference |
| Current smoker | 0.95 (0.77 – 1.14) | 1.14 (1.04 – 1.25) | 1.02 (0.86 – 1. 20) |
| Ex-smoker | 0.98 (0.83 – 1.15) | 1.03 (0.94 – 1.12) | 0.99 (0.87 – 1.14) |
| History of hypertension | 1.14 (0.96 – 1.35) | 1.16 (1.09 – 1.24) | 1.18 (1.02 – 1.37) |
| History of diabetes | 1.28 (1.09 – 1.49) | 1.46 (1.36 – 1.58) | 1.23 (1.08 – 1.39) |
| History of atrial fibrillation | 1.49 (1.25 – 1.76) | 1.63 (1.51 – 1.75) | 1.37 (1.18 – 1.59) |
| Type of MI<br>STEMI | 1.15 (0.97 – 1.37) | 1.18 (1.10 – 1.27) | 1.10 (0.95 – 1.28) |
| History of peripheral arterial disease | 1.53 (1.25 – 1.87) | 1.37 (1.25 – 1.50) | 1.33 (1.13 – 1.57) |
| History of COPD | 1.21 (0.99 – 1.48) | 1.24 (1.15 – 1.38) | 1.22 (1.03 – 1.44) |
| Systolic blood pressure, per 10 mmHg increase | 0.99 (0.95 – 1.03) | 1.00 (0.98 – 1.02) | 1.01 (0.97 – 1.04) |
| Diastolic blood pressure, per 10 mmHg increase | 1.01 (0.94 – 1.09) | 0.99 (0.96 – 1.03) | 0.99 (0.93 – 1.06) |
| ACE inhibitor use before MI | 1.01 (0.87 – 1.17) | 1.06 (0.99 – 1.14) | 1.07 (0.94 – 1.21) |
| Angiotensin receptor blocker (ARB) use before MI | 0.99 (0.95 – 1.03) | 1.00 (0.90 – 1.12) | 0.93 (0.78 – 1.12) |
| Beta-blocker use before MI | 1.01 (0.94 – 1.09) | 0.94 (0.88 – 1.00) | 0.90 (0.79 – 1.02) |

Using multivariable Cox regression without and with multiple imputation and the competing risks regression based on Fine and Gray's proportional subhazards model to account for the competing risk of all cause mortality (n = 4898, n events = 1159). Additional prognostic covariates: body mass index, smoking, systolic and diastolic blood pressure. Overall p-values are shown for categorical covariates. HR=hazard ratio; CI=confidence interval; MI=myocardial infarction; NSTEMI=non-ST-segment-elevation myocardial infarction; STEMI=ST-segment-elevation myocardial infarction; COPD=chronic obstructive pulmonary disease; ACE=Angiotensin-converting enzyme; Q=quintile.

For peer review only

**Table S3 - Hazard ratios for heart failure in patients following a first myocardial infarction stratified on having a previous cancer diagnosis or not.**

| | Model 4 **Subjects without a history of cancer** n = 21,719 n events = 5,029 | Model 4 **Subjects with a history of cancer** n = 2,760 n events = 746 |
|---|---|---|
| | HR (95% CI) | HR (95% CI) |
| Age, per 10 years increase | 1.49 (1.45 – 1.53) | 1.45 (1.30 – 1.61) |
| Men | 1.08 (1.01 – 1.15) | 1.13 (0.93 – 1.37) |
| Index of multiple deprivation | overall p = <0.001 | overall p = <0.001 |
| Q1 | Reference | Reference |
| Q2 | 1.08 (0.97 – 1.20) | 1.07 (0.77 – 1.48) |
| Q3 | 1.17 (1.05 – 1.31) | 1.27 (0.91 – 1.78) |
| Q4 | 1.17 (1.04 – 1.31) | 1.14 (0.79 – 1.66) |
| Q5 | 1.25 (1.10 – 1.41) | 1.43 (0.97 – 2.11) |
| History of hypertension | 1.11 (1.03 – 1.18) | 1.45 (1.16 – 1.81) |
| History of diabetes | 1.53 (1.41 – 1.66) | 1.19 (0.91 – 1.55) |
| History of atrial fibrillation | 1.62 (1.49 – 1.76) | 1.67 (1.33 – 2.09) |
| Type of MI | | |
| STEMI | 1.18 (1.09 – 1.28) | 1.01 (0.77 – 1.31) |
| History of peripheral arterial disease | 1.39 (1.26 – 1.54) | 1.19 (0.88 – 1.62) |
| History of COPD | 1.30 (1.18 – 1.43) | 1.36 (1.03 – 1.81) |
| ACE inhibitor use before MI | 1.11 (1.03 – 1.20) | 1.00 (0.79 – 1.26) |
| Angiotensin receptor blocker (ARB) use before MI | 1.06 (0.93 – 1.20) | 0.92 (0.65 – 1.31) |
| Beta-blocker use before MI | 0.97 (0.90 – 1.04) | 0.99 (0.79 – 1.24) |

All analyses are based on a complete case analysis. Overall p-values are shown for categorical covariates. ACE=Angiotensin-converting enzyme; CI=confidence interval; COPD=chronic obstructive pulmonary disease; MI=myocardial infarction; NSTEMI=non-ST-segment-elevation myocardial infarction; STEMI=ST-segment-elevation myocardial infarction; Q=quintile.

For peer review only

**Table S4 - Hazard ratios for heart failure in patients following a first myocardial infarction stratified on a history of revascularization.**

| | Model 4<br>**Subjects without a history of revascularization**<br>n = 21,160<br>n events = 5,222 | Model 4<br>**Subjects with a history of revascularization**<br>n = 3.319<br>n events = 553 |
|---|---|---|
| | HR (95% CI) | HR (95% CI) |
| Age, per 10 years increase | 1.48 (1.44 – 1.52) | 1.30 (1.17 – 1.44) |
| Men | 1.07 (1.01 – 1.14) | 0.96 (0.75 – 1.23) |
| Index of multiple deprivation | overall p = <0.001 | overall p = <0.001 |
| Q1 | Reference | Reference |
| Q2 | 1.06 (0.95 – 1.17) | 1.13 (0.80 – 1.61) |
| Q3 | 1.14 (1.02 – 1.27) | 1.39 (0.95 – 2.03) |
| Q4 | 1.15 (1.02 – 1.28) | 1.34 (0.85 – 2.02) |
| Q5 | 1.22 (1.08 – 1.38) | 1.29 (0.84 – 1.99) |
| History of hypertension | 1.14 (1.07 – 1.22 | 1.18 (0.92 – 1.50) |
| History of diabetes | 1.49 (1.37 – 1.61) | 1.32 (1.00 – 1.75) |
| History of atrial fibrillation | 1.61 (1.48 – 1.74) | 1.90 (1.39 – 2.58) |
| Type of MI | | |
| STEMI | 1.14 (1.05 – 1.23) | 1.39 (1.07 – 1.79) |
| History of peripheral arterial disease | 1.40 (1.27 – 1.55) | 1.22 (0.88 – 1.62) |
| History of COPD | 1.30 (1.18 – 1.43) | 1.36 (0.92 – 2.03) |
| ACE inhibitor use before MI | 1.11 (1.03 – 1.19) | 1.15 (0.89 – 1.49) |
| Angiotensin receptor blocker (ARB) use before MI | 1.06 (0.94 – 1.19) | 0.79 (0.54 – 1.17) |
| Beta-blocker use before MI | 1.00 (0.94 – 1.07) | 0.84 (0.65 – 1.08) |

All analyses are based on a complete case analysis. Overall p-values are shown for categorical covariates. ACE=Angiotensin-converting enzyme; CI=confidence interval; COPD=chronic obstructive pulmonary disease; MI=myocardial infarction; NSTEMI=non-ST-segment-elevation myocardial infarction; STEMI=ST-segment-elevation myocardial infarction; Q=quintile.

For peer review only

**Supplementary Figure 1 – Cumulative incidence curves for HF accounting for mortality as first event after index MI**

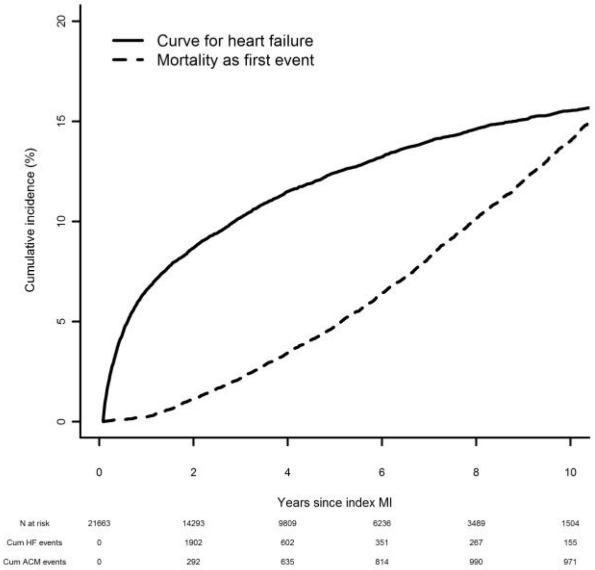


Cumulative incidence curves adjusted for competing risk of mortality as first event. 10-year follow-up in patients who survived the first 30 days and did not develop HF during the first 30 days (30 days event free).

For peer review only

STROBE Statement—checklist of items that should be included in reports of observational studies

| | Item No | Recommendation |
|---|---|---|
| **Title and abstract** | 1 ✓ | (*a*) ✓*page 2* - Indicate the study's design with a commonly used term in the title or the abstract |
| | | (*b*) ✓*page 2* - Provide in the abstract an informative and balanced summary of what was done and what was found |
| **Introduction** | | |
| Background/rationale | 2✓ | ✓*page 4* - Explain the scientific background and rationale for the investigation being reported |
| Objectives | 3✓ | ✓ *page 4* - State specific objectives, including any prespecified hypotheses |
| **Methods** | | |
| Study design | 4✓ | ✓*pages 4 through 6* - Present key elements of study design early in the paper |
| Setting | 5✓ | Describe the setting, locations, and relevant dates, including periods of recruitment, exposure, follow-up, and data collection |
| Participants | 6✓ | ✓*page 5* - (*a*) *Cohort study*—Give the eligibility criteria, and the sources and methods of selection of participants. Describe methods of follow-up<br>*Case-control study*—Give the eligibility criteria, and the sources and methods of case ascertainment and control selection. Give the rationale for the choice of cases and controls<br>*Cross-sectional study*—Give the eligibility criteria, and the sources and methods of selection of participants |
| | | (*b*) *Cohort study*—For matched studies, give matching criteria and number of exposed and unexposed<br>*Case-control study*—For matched studies, give matching criteria and the number of controls per case |
| Variables | 7✓ | ✓*page 5* - Clearly define all outcomes, exposures, predictors, potential confounders, and effect modifiers. Give diagnostic criteria, if applicable |
| Data sources/ measurement | 8✓* | ✓*page 5* - For each variable of interest, give sources of data and details of methods of assessment (measurement). Describe comparability of assessment methods if there is more than one group |
| Bias | 9✓ | ✓*page 6* - Describe any efforts to address potential sources of bias |
| Study size | 10✓ | ✓*page 7 and figure 1*- Explain how the study size was arrived at |
| Quantitative variables | 11✓ | ✓*page 6* - Explain how quantitative variables were handled in the analyses. If applicable, describe which groupings were chosen and why |
| Statistical methods | 12✓ | (*a*) *NA* Describe all statistical methods, including those used to control for confounding |
| | | (*b*) *NA* Describe any methods used to examine subgroups and interactions |
| | | (*c*) ✓*page 6* - Explain how missing data were addressed |
| | | (*d*) ✓*page 6* - *Cohort study*—If applicable, explain how loss to follow-up was addressed<br>*Case-control study*—If applicable, explain how matching of cases and controls was addressed<br>*Cross-sectional study*—If applicable, describe analytical methods taking account of sampling strategy |
| | | (*e*) ✓Describe any sensitivity analyses |

For peer review only

Continued on next page

For peer review only


For peer review only - http://bmjopen.bmj.com/site/about/guidelines.xhtml

| | | |
|---|---|---|
| **Results** | | |
| Participants | 13* | ✓*page 6* - (a) Report numbers of individuals at each stage of study—eg numbers potentially eligible, examined for eligibility, confirmed eligible, included in the study, completing follow-up, and analysed |
| | | ✓*page 6 and figure 1* - (b) Give reasons for non-participation at each stage |
| | | ✓*figure 1* - (c) Consider use of a flow diagram |
| Descriptive data | 14*✓ | (a) ✓*table 1* - Give characteristics of study participants (eg demographic, clinical, social) and information on exposures and potential confounders |
| | | (b) ✓*table 1* - Indicate number of participants with missing data for each variable of interest |
| | | (c) ✓*table 1- Cohort study*—Summarise follow-up time (eg, average and total amount) |
| Outcome data | 15* | ✓*table 1, figures 2-3 and pages 7-8 Cohort study*—Report numbers of outcome events or summary measures over time |
| | | *Case-control study*—Report numbers in each exposure category, or summary measures of exposure |
| | | *Cross-sectional study*—Report numbers of outcome events or summary measures |
| Main results | 16 | ✓*table 2, appendix tables* and pages 7-8 - (*a*) Give unadjusted estimates and, if applicable, confounder-adjusted estimates and their precision (eg, 95% confidence interval). Make clear which confounders were adjusted for and why they were included |
| | | *NA* (*b*) Report category boundaries when continuous variables were categorized |
| | | ✓ absolute rates reported in pages 7-8 - (*c*) If relevant, consider translating estimates of relative risk into absolute risk for a meaningful time period |
| Other analyses | 17 | ✓*pages 7-8* - Report other analyses done—eg analyses of subgroups and interactions, and sensitivity analyses |
| **Discussion** | | |
| Key results | 18 | ✓*page 8* Summarise key results with reference to study objectives |
| Limitations | 19 | ✓*pages 9-10* - Discuss limitations of the study, taking into account sources of potential bias or imprecision. Discuss both direction and magnitude of any potential bias |
| Interpretation | 20 | ✓*pages 8-10* Give a cautious overall interpretation of results considering objectives, limitations, multiplicity of analyses, results from similar studies, and other relevant evidence |
| Generalisability | 21 | ✓ *page 9* Discuss the generalisability (external validity) of the study results |
| **Other information** | | |
| Funding | 22 | ✓*page 11* Give the source of funding and the role of the funders for the present study and, if applicable, for the original study on which the present article is based |

*Give information separately for cases and controls in case-control studies and, if applicable, for exposed and unexposed groups in cohort and cross-sectional studies.

**Note:** An Explanation and Elaboration article discusses each checklist item and gives methodological background and published examples of transparent reporting. The STROBE checklist is best used in conjunction with this article (freely available on the Web sites of PLoS Medicine at http://www.plosmedicine.org/, Annals of Internal Medicine at http://www.annals.org/, and Epidemiology at http://www.epidem.com/). Information on the STROBE Initiative is available at www.strobe-statement.org.

For peer review only

BMJ Open

# An Electronic Health Records cohort study on Heart Failure Following Myocardial Infarction in England: Incidence and Predictors

| | |
|---:|---|
| Journal: | *BMJ Open* |
| Manuscript ID | bmjopen-2017-018331.R5 |
| Article Type: | Research |
| Date Submitted by the Author: | 29-Nov-2017 |
| Complete List of Authors: | Gho, Johannes; Universitair Medisch Centrum Utrecht, Cardiology; Farr Institute of Health Informatics Research, University College London, Epidemiology and Public Health<br>Schmidt, Amand; Faculty of Science and Engineering, Groningen Research Institute of Pharmacy; University College London, Institute of Cardiovascular Science<br>Pasea, Laura; Farr Institute of Health Informatics Research, University College London, Epidemiology and Public Health<br>Koudstaal, Stefan; Farr Institute of Health Informatics Research, University College London, Epidemiology and Public Health; University Medical Centre Utrecht, Department of Cardiology<br>Pujades Rodriguez, Mar; Farr Institute of Health Informatics Research, University College London, Epidemiology and Public Health; University of Leeds, Medical Research Council Bioinformatics Centre, Leeds Institute of Biomedical and Clinical Sciences<br>Denaxas, S; Farr Institute of Health Informatics Research, University College London, Epidemiology and Public Health<br>Shah, Anoop; UCL, Farr Institute of Health Informatics research<br>Patel, Riyaz; Farr Institute of Health Informatics Research, University College London, Epidemiology and Public Health<br>Gale, Chris; University of Leeds, Medical Research Council Bioinformatics Centre, Leeds Institute for Cardiovascular and Metabolic Medicine<br>Hoes, Arno; Julius Center for Health Sciences and Primary Care, University Medical Center, Utrecht<br>Cleland, J; Imperial College London, National Heart & Lung Institute, Royal Brompton & Harefield Hospitals<br>Hemingway, Harry; Farr Institute of Health Informatics Research, University College London, Epidemiology and Public Health<br>Asselbergs, Folkert; University Medical Centre Utrecht, Department of Cardiology; ICIN-Netherlands Heart Institute, Durrer Center for Cardiogenetic Research |
| <b>Primary Subject Heading</b>: | General practice / Family practice |
| Secondary Subject Heading: | Cardiovascular medicine, Epidemiology, General practice / Family practice, Health informatics, Public health |
| Keywords: | Heart failure < CARDIOLOGY, Myocardial infarction < CARDIOLOGY, electronic health records |

SCHOLARONE™
Manuscripts

For peer review only

# An Electronic Health Records cohort study on Heart Failure Following Myocardial Infarction in England: Incidence and Predictors

Johannes M.I.H. Gho, MD, PhD,[1,2,*] Amand F. Schmidt, PhD,[2,3,4*] Laura Pasea, MSc,[1] Stefan Koudstaal, MD, MSc, PhD,[1,2] Mar Pujades-Rodriguez, PhD,[1,5] Spiros Denaxas, PhD,[1] Anoop D. Shah, MRCP,[1] Riyaz S. Patel, MD, PhD,[1,3] Chris P. Gale, PhD, FRCP,[6] Arno W. Hoes, MD, PhD,[7] John G. Cleland, MD, FRCP,[8] Harry Hemingway, FFPH, FRCP,[1] Folkert W. Asselbergs, MD, PhD,[1,2,3,9]

[1]Farr Institute of Health Informatics Research, UCL Institute of Health Informatics, University College London, London, UK
[2]Department of Cardiology, Division Heart and Lungs, University Medical Center Utrecht, Utrecht, the Netherlands
[3]Institute of Cardiovascular Science, Faculty of Population Health Sciences, University College London, London, UK
[4]Groningen Research Institute of Pharmacy, University of Groningen, Groningen, the Netherlands
[5]Medical Research Council Bioinformatics Centre, Leeds Institute of Biomedical and Clinical Sciences, University of Leeds, Leeds, UK
[6]Medical Research Council Bioinformatics Centre, Leeds Institute for Cardiovascular and Metabolic Medicine, University of Leeds, Leeds, UK
[7]Julius Center for Health Sciences and Primary Care, University Medical Center Utrecht and Utrecht University, Utrecht, the Netherlands
[8]National Heart & Lung Institute, Royal Brompton & Harefield Hospitals, Imperial College London, London, UK
[9]Durrer Center for Cardiogenetic Research, ICIN-Netherlands Heart Institute, Utrecht, the Netherlands
* Shared first author

**Corresponding author:**
A Floriaan Schmidt
Institute of Cardiovascular Science, University College London
222 Euston Road, London NW1 2DA, United Kingdom
E-mail: amand.schmidt@ucl.ac.uk; a.f.schmidt@rug.nl
Tel: 0044 (0)20 3549 5625

**Word count text**: 3627
**Word count abstract**: 242
**Number of references**: 34
**Number of tables**: 2
**Number of figures**: 3
**(Web)appendix**: 1

For peer review only


For peer review only - http://bmjopen.bmj.com/site/about/guidelines.xhtml

**Abstract**

**Objectives** To investigate the incidence and determinants of heart failure (HF) following a myocardial infarction (MI) in a contemporary cohort of MI patients using routinely collected primary and hospital care electronic health records.

**Methods** Data were used from the CALIBER programme, linking electronic health records in England from primary care, hospital admissions, an MI registry and mortality data. Subjects were eligible if they were 18 years or older, did not have a history of HF, and survived a first MI. Factors associated with time to HF were examined using Cox proportional hazard models.

**Results** Of the 24,479 MI patients 5,775 (23.6%) developed HF during a median follow-up of 3.7 years (incidence rate per 1000 person-years 63.8 (95% CI: 62.2 – 65.5). Baseline characteristics significantly associated with developing HF were: atrial fibrillation [HR 1.62, 95% CI 1.51 – 1.75], age [per 10 years increase: 1.45, 1.41 – 1.49], diabetes [1.45, 1.35 – 1.56], peripheral arterial disease [1.38, 1.26 – 1.51], COPD [1.28, 1.17 – 1.40], greater socioeconomic deprivation [5$^{th}$ vs. 1$^{st}$ quintile 1.27, 1.13 – 1.41], ST-segment elevation MI at presentation [1.19, 1.11 – 1.27], and hypertension [1.16, 1.09 – 1.23]. Results were robust to various sensitivity analyses such as competing risk analysis and multiple imputation.

**Conclusion** In England, one in four survivors of a first MI develop HF within four years. This contemporary study demonstrates MI patients are at considerable risk of HF. Baseline patient characteristics associated with time till HF were identified, which may be used to target preventive strategies.

**Keywords:** heart failure; myocardial infarction; incidence; risk factors; electronic health records

For peer review only



For peer review only - http://bmjopen.bmj.com/site/about/guidelines.xhtml

**Strengths and limitations of this study**

- ❖ This study based on the use of linked electronic health records from general practitioners and hospital records, describes the current burden of heart failure in a representative sample of patients with a first myocardial infarction.
- ❖ The linkage of data from three sources (disease registry, primary care and hospital records) improved diagnostic ascertainment and accuracy in timing of events.
- ❖ Misclassification of drug exposure was likely to be minimal, as prescriptions issued during consultation are automatically recorded.
- ❖ Risk factor adjustment might have been incomplete given that information regarding baseline BMI, smoking and blood pressure was missing for 34 to up to 70% of patients.
- ❖ Stratified methods were used to account for potential calendar and centre effects, and competing risk models were used to adjust for potential competing effects on heart failure and mortality.
- ❖ Due to the high degree of missing data on time to revascularization (88.3%%) we did not explore its relation with HF incidence.

**Abbreviations list:** heart failure (HF); myocardial infarction (MI); ST-segment elevation MI (STEMI); hazard ratio (HR); confidence interval (CI); chronic obstructive pulmonary disease (COPD); body mass index (BMI).

For peer review only

## Introduction

Research describing the incidence of heart failure (HF) following myocardial infarction (MI) is limited, mainly originating from the thrombolytic era, often using small sample sizes with contradictory findings, making it difficult to provide evidence based medicine. For example, a previous UK study among almost 900 patients hospitalized with MI in 1998, found that one fifth developed HF during their hospital stay and a further third following hospital discharge[1]. More recently, a Swedish study found a 5-year cumulative risk of HF after MI of 21.8% in the calendar period 2004-2013[2]. Further, a Danish nationwide cohort study reported an incidence of HF at 90-days following MI of 19.6% in 2009-10[3]. Differences between these studies could be related to a number of factors, for example change in treatment, national policies, or definitions of heart failure, all of which potentially limit the generalizability of results. We used a large contemporary and representative sample of patients with MI based on electronic health records (EHR) to 1) describe the current incidence of HF after MI in England, using both primary and hospital care data, and 2) explore patient characteristics predictive of post MI HF.

## Methods

### Study design

Data were used from the CALIBER dataset, which included linked data from (i) primary care electronic health records, with diagnoses coded using the Read terminology (Clinical Practice Research Datalink, CPRD) from 226 consenting general practices[4], (ii) hospital care administrative records (Hospital Episode Statistics, HES) and disease registry/audit records (Myocardial Ischaemia National Audit Project, MINAP), and (iii) the mortality register (from UK Office for National Statistics, ONS) [5].

### Setting and participants

Patients were eligible if they were aged ≥18 years, registered in CPRD practices in England consenting to data linkage, with at least one year of up-to-standard pre-study follow-up (meeting CPRD practice quality standards), and experiencing a first MI between 1$^{st}$ January 1998 and 25$^{th}$ March 2010. The first record of



For peer review only - http://bmjopen.bmj.com/site/about/guidelines.xhtml

MI across the linked data sources was considered as the index event and subsequent MI records within 30 days from the same or other sources were considered as representing the same event [6]. Patients with a fatal index MI or a history of HF before their index MI were excluded. Patients were censored if they ceased to be registered at a participating general practice, at the date of death or the administrative censoring of the dataset (25th March 2010).

Variables and data sources

Study variables were derived from diagnoses recorded across several controlled clinical terminology and statistical classification systems: Read, ICD-9 or ICD-10, medication prescription information or the Office of Population Censuses and Surveys Classification of Interventions and Procedures (OPCS-4) codes (see https://rdrr.io/rforge/CALIBERcodelists/man/PRODDICT.html) with the electronic health record phenotyping algorithms published at https://caliberresearch.org/portal [5].

For continuous variables, the most recent measurement recorded in CPRD in the year before study entry was used as the baseline value. Data before study entry were used to determine the prognostic potential of age (years, using the date of birth), sex, ethnicity, social deprivation (Index of Multiple Deprivation, IMD score as recorded in ONS in quintiles), smoking, alcohol use, history of cardiovascular disease, previous coronary revascularization, history of diabetes, history of thyroid disease, history of chronic obstructive pulmonary disease (COPD), history of depression and history of non-metastatic cancer. (see the CALIBER portal https://caliberresearch.org/portal for details). Diabetes and hypertension diagnosis were based on Read codes from primary care or ICD-10 codes from Hospital Episode Statistics, both of which were classified using primary care consultation records and hospitals diagnosis records. Socioeconomic status was based on the Index of Multiple Deprivation which includes seven domains of deprivation (income deprivation, employment deprivation, health deprivation and disability, education, skills and training deprivation, barriers to housing and services, living environment deprivation and crime) [7]. The index MI event was characterized using ECG findings (e.g. ST-segment-elevation myocardial infarction (STEMI), or non-STEMI (NSTEMI)), site of infarction, mode and timing of reperfusion and peak cardiac biomarkers (troponin I, troponin T and CK). MI was defined by linking all of the CALIBER data sources



For peer review only - http://bmjopen.bmj.com/site/about/guidelines.xhtml

(e.g., Hospital Episode Statistics and CPRD), with the type of MI recorded in MINAP and CPRD, see for more detail https://www.caliberresearch.org and the appendix. Characteristics regarding the index MI (e.g., type of revascularization and delay from symptom onset to delivery of reperfusion therapy) were derived from MINAP. This nationwide registry is part of an annual audit which records over 20 key MI variables [8]. Herrett and colleagues [6] validated the current approach and showed the necessity of linking multiple sources to ascertain MI (see appendix for codes used). The primary study outcome was the first HF event following MI, which similar to MI, was recorded in multiple CALIBER data sources; echocardiographic findings and NYHA class were unavailable. All phenotypes were created and validated using a robust methodology described elsewhere [9] and have been used in previously published studies[10,11].

Statistical methods

Incidence rates (cases per 1000 person-years) and Kaplan-Meier cumulative incidence rates of HF were estimated, with Kaplan-Meier curves stratified by age (<50, 50-65, ≥65 years) and type of MI. Association between baseline variables with the onset of HF following MI were explored using Cox proportional hazard models. The proportional hazards assumption was checked using Schoenfeld residuals, and by nonparametric correlation coefficients between survival time and the parameter specific residuals. All models were stratified on general practice and calendar year periods of enrolment (1998-2001, 2001-2004, 2004-2007 and 2007-2010). Stratified models were used instead of frailty models because the former does not make any distributional assumption. Models were sequentially adjusted for: 1) age and sex, 2) cardiovascular risk factors, 3) type of MI, and 4) co-morbidities and prescribed medication. Associations are presented as hazard ratios (HRs) with 95% confidence intervals (CI). Analyses were performed using R (version 3.3.2)[12].

Sensitivity analyses

Due to the availability of EHR from multiple sources, clinical diagnoses and prescriptions were completely recorded. Biomarker and lifestyle measurements such as smoking, BMI, and blood pressure were however, incompletely recorded (see Table 1). It is probable that these data were preferentially recorded

For peer review only


For peer review only - http://bmjopen.bmj.com/site/about/guidelines.xhtml

in subjects perceived to be at a higher risk for early progression of CVD. Pairwise analyses regressing a complete case indicator on observed variables indeed showed considerable dependency between recorded data and missingness (results not shown), violating the “missing completely at random” assumption. This dependency was utilized by multiply imputing missing data using the mice package, which was implemented using 20 imputed datasets and pooled based on Rubin’s rules (Supplementary methods)[13].

To account for the fact that patients may have died before the onset of HF (e.g., competing risk by mortality), analyses were repeated using Fine and Gray models[14,15]. As a hypothetical example of competing risk, let us assume that out of 100 MI subjects, one develops HF and 99 die. In a Cox’s regression analysis all 99 dead subjects would be censored (implicitly, and incorrectly, assuming they may develop HF later in time), and the hazard of HF would equal 1/1. Instead a Fine and Gray model recognizes the 99 died subjects could never develop HF and hence calculates the hazard as 1/100.

In addition to these sensitivity analyses, we performed a cancer- and revascularization-stratified analysis. Due to discrepancies in sample size between the subgroups we decided against formal interaction testing, which may suffer from inflated false positive rates and lower power in such settings[16].

Ethical approval

CALIBER has received ethics approval (Supplementary methods). This study is in compliance with the Declaration of Helsinki and was approved by the ISAC (Independent Scientific Advisory Committee) for MHRA Database Research (protocol no. 14_198R) and the MINAP Academic Group and is registered with ClinicalTrials.gov (NCT02384213).

## Results

Subjects and baseline characteristics

For peer review only

In total there were 52,770 patients with an index MI during the study period (Figure 1). Excluding patients with a fatal index MI (n = 15,104), a prior history of HF (n = 3,876), missing information on the type of MI (n = 97) and subjects with less than 1 year of follow-up prior to the indexing MI event (n = 9,214), resulted in an analytical cohort of 24,479 patients. Patients were recruited from 226 GP practices, with the median practice enrolling 101 patients (quartile (Q1) 65 and Q3 150). Of the included subjects 4,657 were STEMI and 19,822 NSTEMI patients (0% missing), 15,969 (65.2%) were men (0% missing), 4,538 (42.1%) currently smoked (56% missing), 12,258 (50.1%) had hypertension (0% missing), and 3,014 (12.3%) had a history of diabetes at baseline (0% missing). In total, 6,129 (25.0%) were prescribed beta-blockers (0% missing) and 5,039 (20.6%) angiotensin converting enzyme (ACE) inhibitors (0% missing) prior to their indexing MI (Table 1). The number of patients identified across the previously defined calendar periods was reasonably stable, with a minimum of 5219 subjects in the period 1998-2001 compared to a maximum of 6838 subjects identified in the period 2004-2007.

Incidence

Patients contributed 90,482 person years of follow-up, during a median follow-up time of 3.7 years (IQR: 1.5; 6.7), 5,775 patients (23.6%) developed HF. The crude incidence rate of HF following a first MI was 63.8 (95% CI: 62.2 – 65.5) per 1000 person-years. Within the first 30 days post MI follow-up 2,438 (10.0%) patients developed HF (Figure 2). From day 30 onwards 3,337 (15.8%) of MI patients (event free during the first 30 days) developed HF, with 6.8% experiencing an HF event within the first year (Figure 2). The incidence of HF during the first 30-days of follow-up was 4.3% (102) in patients younger than 50 years, 6.0% (459) in 50-65 year olds and 12.9% (1877) in those 65 years and older, with HF incidence increasing proportionally as time progressed towards 10 years (Figure 3 top row). The 30-day incidence of HF was 9.5% (1892 events) for NSTEMI subjects and 11.7% (546 events) for STEMI subjects. Excluding patients who experienced a HF within the first 30 days showed that STEMI patients had a lower incidence of HF than NSTEMI subjects (Figure 3 lower panels). At 57 days the crude cumulative risk of HF in the NSTEMI subjects surpassed that of the STEMI subjects for the first time (0.0151 versus 0.0147), with the curves further diverging at 73 days of follow-up (since indexing MI).

For peer review only

Over the entire follow-up 5,921 subjects died, of which 3,538 were free of HF at the time. During the first 30 days of follow-up only 1 patient died, limiting the potential for competing risk by all-cause mortality. In patients who did not have HF after 30 days accounting for competing risk by all-cause mortality attenuated the cumulative risk to be about 15% after 10 years of follow-up (Supplementary data). Furthermore, the cumulative risks of all-cause mortality and HF converged as follow-up time progressed towards 10 years.

Predictors of HF after multivariable adjustments

Table 2 describes the results of the previously described nested Cox's models, showing little change in estimates between the increasingly complex models. Focusing on the final model, the hazard of HF increased with atrial fibrillation [HR 1.62, 95%CI 1.51 – 1.75], age [per 10 years increase: 1.45, 1.41 – 1.49], diabetes [1.45, 1.35 – 1.56], peripheral arterial disease [1.38, 1.26 – 1.51], COPD [1.28, 1.17 – 1.40], hypertension [1.16, 1.09 – 1.23], higher socioeconomic deprivation (p-value for the 5 groups < 0.001), and STEMI at presentation [1.19, 1.11 – 1.27)]. Accounting for competing risk by all-cause mortality had little impact on the presented results (Supplementary data). After multiply imputing the data models were extended to include smoking, BMI, and systolic and diastolic blood pressure variables which showed BMI, male sex and smoking to be conditionally independent prognostic factors (Supplementary data). The extended model 5 was implemented using imputed data, and non-imputed (complete case) data, resulting in similar HR estimates (magnitude and direction). Stratifying the sample on cancer diagnosis or history of revascularization showed broadly similar results between subgroups (See Supplementary data, focusing on the confidence intervals), however precision was low due to the limited number of patients with a history of revascularization or cancer.

**Discussion**

In this large population-based study using linked electronic health records, 23.6% of patients who survived a first MI developed HF during a median follow-up of 3.7 years, resulting in an incidence rate of 64 cases per 1000 person-years. Incident HF was associated with increasing age, higher socioeconomic


For peer review only - http://bmjopen.bmj.com/site/about/guidelines.xhtml

deprivation, a history of diabetes, atrial fibrillation, peripheral arterial disease, COPD, STEMI at presentation, BMI and smoking.

A previous Canadian study, using data from the period 1994-2000[17], found that 71% of elderly patients without HF at index admission developed HF within 5 years' time after an MI, whereas the mortality due to MI decreased in the same period. The Framingham Heart Study[18], using data from 1990 to 1999, found a 5-years post-MI HF incidence of 31.9% after MI; lower than found in the Canadian study. Importantly, both studies showed a higher incidence of post-MI HF than our more contemporary English cohort. This lower HF incidence is likely due to continued improvements of MI treatment strategies, which are reflected in a decreased HF incidence over calendar time. For example, a 20,812 sample of MI patients hospitalized in Western Australia showed that the overall 1-year incidence of HF after MI decreased from 28.1% 1996 in to 16.5% in 2007[19]. This decline is confirmed further by a national Swedish hospital discharge and death registry study reporting a one third decline in incidence between 1993-2004[20].The same Swedish group recently reported that this trend persists for the period 2004-2013[2] and showed improved pharmacological treatment and early revascularization in this period. During a median follow-up of 4 years, 19% of the patients were re-hospitalized because of HF. An explanation for the higher percentage in our study is probably the inclusion of HF diagnosis made in the GP setting next to hospital records decreasing misclassification of HF events. Future efforts are needed to harmonize these national data sources to compare daily care and HF epidemiology across different countries.

Prognostic factors

Similar to the current study, Torabi et al[21] reported that HF after MI increased steeply with age. Socioeconomic deprivation has been shown to be an independent predictor of HF development and associated with an increased incidence of HF in MI-free subjects [22,23]. Socioeconomic deprivation has also been associated with more frequent hospital admission and higher mortality in HF patients[24–26]. The current study extends these observations to HF incidence after an MI. Furthermore, we showed that a history of diabetes, atrial fibrillation, peripheral arterial disease, COPD, STEMI at presentation, BMI and smoking are all independently prognostic of HF after MI. Interestingly, despite the size of the collected

For peer review



For peer review only - http://bmjopen.bmj.com/site/about/guidelines.xhtml

sample, sex only became significantly associated with HF after accounting for differences in BMI and smoking. Furthermore, the impact of male sex (HR of 1.07 95%CI 1.01 – 1.13) was modest indicating relative sex equality. It is of note that blood pressure was not significantly associated with HF. However, blood pressure measurements were frequently missing, which (even after multiple imputation) may be the cause of the observed lack of association hence this deserves independent exploration. In this light it is important to note that the diagnosis of hypertension was associated with HF. Potentially this discrepancies between the association of blood pressure measurement and hypertension diagnosis can be explained by noting that a recorded diagnosis is indicative of long-term hypertensions, which may be different than a single blood pressure measurement in terms of prognosis. Further, given that both variables were included in the same model, the observed difference in association may suggest that conditional on hypertension, blood pressure itself is only modestly associated to HF, if at all.

Strengths and limitations

The linkage of multiple electronic health record sources from primary and hospital care allowed for the collection of a representative sample[27], which enabled us to explore the prognostic value of routinely collected data in primary care records and to detect non-hospitalized HF cases. The population of CPRD practices has been shown broadly representative of the UK population[4,28]. In total 226 GP practices consented to data linkage with Hospital Episode Statistics, MINAP, and the Office for National Statistics (containing 3.9% of the population of England in 2006). A potential limitation is that the ascertainment of cardiovascular outcomes was not based on clinical criteria (e.g., validated questionnaires and properly conducted physical examinations), practices of medical coding will have changed over time and there could be subgroups of patients with left ventricular dysfunction without clinical symptoms. Calendar dependent changes over time were accounted for by using time-period stratified Cox models. We further wish to highlight the infrequent use of PCI in STEMI patients, which was shown to be representative of the slower uptake in England[29]. Similarly, 32% of the patients used antiplatelet drugs at baseline which may be a reflection of non-MI CVD burden. The Kaplan-Meier plots for subtype of MI indicate a possible violation of the proportional hazard assumption for this variable. However, these plots represent the pairwise associations between MI subtype and time to HF, and as such assume that MI subtype is

For peer review only

independent of other prognostic factors, which is known to be false. The importance of conditioning on covariates is underlined by noting the Kaplan-Meier plots indicating a protective effect of STEMI versus NSTEMI, which was significantly reversed after correcting for covariates (models 3-4). Similarly, instead of using Kaplan-Meier plots to assess the proportional hazard assumption of the *crude* associations, we used Schoenfeld residuals to explore this assumption for the *conditional* associations (multivariable model 4); which did not show any violations. Based on model 4 the absolute correlation between the Schoenfeld residuals and time was < 0.10 (e.g., for MI subtype this was 0.06) indicating an absence of relevant interaction by time (e.g., non-proportionality of hazard). Residual confounding due to medication use (or other missing/mis-specified variables) might be another potential source of bias, however our intention was not to perform a causal analysis between drug prescriptions and time to HF. As such it is interesting to note that despite the large sample size we did not observe a significant association of ACE, ARB or beta-blocker prescribed prior to the indexing MI event and time to HF. Related, we acknowledge we did not assess **all** potential predictors of post MI HF, for example due to the high degree of missingness we did not explore the prognostic potential of time to revascularization[30].

We adhered to CPRD recommendations to obtain up-to-standard baseline data by excluding patients with less than 1 year at CPRD practice prior to index MI. Previously Lewis and colleagues showed that 3 months after registration with a new practice most patient characteristics were updated correctly, which approximated 100% after 1 year of follow-up[31]. Assuming that duration of the CPRD follow-up is independent of the relations assessed here, excluding such patients should not hamper generalizability of results. Using the first MI recorded in the database without a prior history of HF, might have introduced bias due to left truncation (e.g., some subjects may already have experienced an MI before enrolment)[32]. However CALIBER holds longitudinal records from primary and hospital care, making it unlikely a large part of the patients were misclassified as MI free and the one year of follow-up prior to entry further decreases the likelihood of misclassifying patients[31]. While the current data is adequate to identify subjects with a first MI, the sub-classification of MI patients into STEMI and NSTEMI, despite recent improvements, is known to be error prone [33]. As such, results for MI type need replication using higher quality data, in perhaps purposely designed studies. Due to our interest in HF occurrence after a

For peer review only


For peer review only - http://bmjopen.bmj.com/site/about/guidelines.xhtml

first MI selection bias (e.g., index event bias) was introduced[34]. This index event bias does not impact the descriptive or prognostic value of the association presented, and is mostly relevant if one wants to develop an intervention based on the associations presented; which was not the aim of this study. Additionally, we note that we reduced the influence of selection bias by accounting for dependencies between predictors. An important caveat of electronic health care records is that these data are predominantly focussed on recording diagnoses and prospections but not on their complement (i.e., who is **not** diseased or who **did** not receive a drug). As such we have assumed that subjects without a recorded drug prescription or diagnoses was unexposed or free of (that specific) disease. Provided that electronic registration is required for a patient to fill a prescription we can be fairly confident that we did not miss many prescribed treatments. However, it is likely that some subjects were misclassified as free of disease while in fact they were not. We've attempted to minimize this misclassification by linking data across multiple healthcare settings and data sources (MINAP, HES and CPRD, ONS).

A final limitation is that there is a possible delay between primary and hospital care records. Previous research has shown that MI events tend to be recorded in primary care after the HES or MINAP record [6]. The lower 30-day HF incidence in unclassified MI patients primarily derived from primary care could be partly explained because of a delay in coding. Therefore, we showed cumulative incidence rates in patients (alive and HF event free within the first 30 days) from 30 days after index MI to account for a delay in recording of MI in primary care. We were unable to differentiate between HF with preserved ejection fraction (HFpEF) and HF with reduced ejection fraction (HFrEF) as we had no access to detailed (echocardiographic) parameters to assess diastolic dysfunction. It is likely, however that the majority of our HF patients had developed systolic dysfunction after MI.

## Conclusion

In this large cohort study using linked electronic health records in England from primary and hospital care, about one in four people developed HF within a median of four years after surviving a first MI. Increasing age, higher socioeconomic deprivation, a history of hypertension, diabetes, atrial fibrillation, peripheral

For peer review only

arterial disease, COPD, smoking and STEMI at presentation were independent determinants new onset HF following MI.

**Authors' contributions**

JMIHG, AFS, LP, SK, MPR, SD, ADS, RSP, CPG, AWH, JGC, HH, FWA contributed to the idea and design of the study. JMIHG extracted and prepared the data for analysis. AFS and JMIHG performed the analysis. JMIHG drafted the manuscript, with revisions from AFS, LP, SK, MPR, SD, ADS, RSP, CPG, AWH, JGC, HH, FWA. JMIHG guaranties the quality and accuracy of the results presented.

**Competing interests**

We have read and understood BMJ policy on declaration of interests and declare that we have no competing interests

**Data Sharing Statement**

Access to raw data can be requested from the CPRD http://cprd.com

**Funding**

This study was supported by the National Institute for Health Research (RP-PG-0407–10314), Wellcome Trust (086091/Z/08/Z), the Medical Research Council Prognosis Research Strategy Partnership (G0902393/99558) and the Farr Institute of Health Informatics Research, funded by The Medical Research Council (MR/K006584/1), in partnership with Arthritis Research UK, the British Heart Foundation, Cancer Research UK, the Economic and Social Research Council, the Engineering and Physical Sciences Research Council, the National Institute of Health Research, the National Institute for Social Care and Health Research (Welsh Assembly Government), the Chief Scientist Office (Scottish Government Health Directorates) and the Wellcome Trust. Part of this work is funded through the Innovative Medicines Initiative 2 Joint Undertaking under grant agreement No 116074, BigData@Heart. AFS is funded by UCLH NIHR Biomedical Research Centre and is a UCL Springboard Population Health Sciences Fellow. HH is an NIHR Senior Investigator, and receives support from the NIHR University College Hospitals / University College London Biomedical Research Centre. FWA is supported by a Dekker scholarship-Junior Staff Member 2014T001 – Netherlands Heart Foundation and UCL Hospitals NIHR Biomedical Research Centre.

For peer review only

## References


1 Torabi A, Cleland JGF, Khan NK, *et al.* The timing of development and subsequent clinical course of heart failure after a myocardial infarction. *Eur Heart J* 2008;**29**:859–70.

2 Desta L, Jernberg T, Spaak J, *et al.* Risk and predictors of readmission for heart failure following a myocardial infarction between 2004 and 2013: A Swedish nationwide observational study. *Int J Cardiol* Published Online First: May 2017.http://linkinghub.elsevier.com/retrieve/pii/S0167527316348677 (accessed 22 Aug 2017).

3 Gjesing A, Gislason GH, Kober L, *et al.* Nationwide trends in development of heart failure and mortality after first-time myocardial infarction 1997-2010: A Danish cohort study. *Eur J Intern Med* 2014;**25**:731–8.

4 Herrett E, Gallagher AM, Bhaskaran K, *et al.* Data Resource Profile: Clinical Practice Research Datalink (CPRD). *Int J Epidemiol* 2015;**44**:827–36.

5 Denaxas SC, George J, Herrett E, *et al.* Data resource profile: Cardiovascular disease research using linked bespoke studies and electronic health records (CALIBER). *Int J Epidemiol* 2012;**41**:1625–38.

6 Herrett E, Shah AD, Boggon R, *et al.* Completeness and diagnostic validity of recording acute myocardial infarction events in primary care, hospital care, disease registry, and national mortality records: cohort study. *BMJ* 2013;**346**:f2350.

7 DCLG. The English Indices of Deprivation 2010. *Neighb Stat Release* 2011;:1–20.http://www.communities.gov.uk/publications/corporate/statistics/indices2010technicalreport

8 Herrett E, Smeeth L, Walker L, *et al.* The Myocardial Ischaemia National Audit Project (MINAP). *Heart* 2010;**96**:1264–7.http://www.ncbi.nlm.nih.gov/pubmed/23177996

9 Morley KI, Wallace J, Denaxas SC, *et al.* Defining Disease Phenotypes Using National Linked Electronic Health Records: A Case Study of Atrial Fibrillation. *PLoS One* 2014;**9**:e110900.

10 Rapsomaniki E, Timmis A, George J, *et al.* Blood pressure and incidence of twelve cardiovascular diseases: Lifetime risks, healthy life-years lost, and age-specific associations in 1·25 million people. *Lancet* 2014;**383**:1899–911. doi:10.1016/S0140-6736(14)60685-1

11 Shah AD, Langenberg C, Rapsomaniki E, *et al.* Type 2 diabetes and incidence of cardiovascular


For peer review only



For peer review only - http://bmjopen.bmj.com/site/about/guidelines.xhtml

diseases: a cohort study in 1·9 million people. *Lancet Diabetes Endocrinol* 2015;**3**:105–13. doi:10.1016/S2213-8587(14)70219-0

12 R Core Team. R: A language and environment for statistical computing. 2017.

13 Marshall A, Altman DG, Holder RL, *et al.* Combining estimates of interest in prognostic modelling studies after multiple imputation: current practice and guidelines. BMC Med. Res. Methodol. 2009;**9**:57-.

14 Satagopan JM, Ben-Porat L, Berwick M, *et al.* A note on competing risks in survival data analysis. *Br J Cancer* 2004;**91**:1229–35.http://www.nature.com/doifinder/10.1038/sj.bjc.6602102

15 Gray B. cmprsk: Subdistribution Analysis of Competing Risks. 2014.http://cran.r-project.org/package=cmprsk

16 Schmidt AF, Groenwold RHH, Knol MJ, *et al.* Exploring interaction effects in small samples increases rates of false-positive and false-negative findings: Results from a systematic review and simulation study. *J Clin Epidemiol* Published Online First: 2014. doi:10.1016/j.jclinepi.2014.02.008

17 Ezekowitz JA, Kaul P, Bakal JA, *et al.* Declining in-hospital mortality and increasing heart failure incidence in elderly patients with first myocardial infarction. *J Am Coll Cardiol* 2009;**53**:13–20.

18 Velagaleti RS, Pencina MJ, Murabito JM, *et al.* Long-term trends in the incidence of heart failure after myocardial infarction. *Circulation* 2008;**118**:2057–62.

19 Hung J, Teng T-HK, Finn J, *et al.* Trends from 1996 to 2007 in incidence and mortality outcomes of heart failure after acute myocardial infarction: a population-based study of 20,812 patients with first acute myocardial infarction in Western Australia. *J Am Heart Assoc* 2013;**2**:e000172.

20 Shafazand M, Rosengren A, Lappas G, *et al.* Decreasing trends in the incidence of heart failure after acute myocardial infarction from 1993-2004: a study of 175,216 patients with a first acute myocardial infarction in Sweden. *Eur J Heart Fail* 2011;**13**:135–41.

21 Torabi A, Cleland JG, Rigby AS, *et al.* Development and course of heart failure after a myocardial infarction in younger and older people. *J Geriatr Cardiol* 2014;**11**:1–12.http://www.pubmedcentral.nih.gov/articlerender.fcgi?artid=3981977&tool=pmcentrez&rendertype=abstract

22 McAlister FA, Murphy NF, Simpson CR, *et al.* Influence of socioeconomic deprivation on the

For peer review only



For peer review only - http://bmjopen.bmj.com/site/about/guidelines.xhtml

451 primary care burden and treatment of patients with a diagnosis of heart failure in general practice in Scotland: population based study. *BMJ* 2004;**328**:1110.

453 23 Hawkins NM, Jhund PS, McMurray JJ V, *et al.* Heart failure and socioeconomic status: Accumulating evidence of inequality. *Eur J Heart Fail* 2012;**14**:138–46.

455 24 Philbin EF, Dec GW, Jenkins PL, *et al.* Socioeconomic status as an independent risk factor for hospital readmission for heart failure. *Am J Cardiol* 2001;**87**:1367–71.

457 25 Struthers AD, Anderson G, Donnan PT, *et al.* Social deprivation increases cardiac hospitalisations in chronic heart failure independent of disease severity and diuretic non-adherence. *Heart* 2000;**83**:12–6.

460 26 Pujades-Rodriguez M, Timmis A, Stogiannis D, *et al.* Socioeconomic deprivation and the incidence of 12 cardiovascular diseases in 1.9 million women and men: implications for risk prediction and prevention. *PLoS One* 2014;**9**:e104671.

463 27 Mann RD, Andrews EB. *Pharmacovigilance: Second Edition*. 2007.

464 28 Walley T, Mantgani A. The UK General Practice Research Database. *Lancet (London, England)* 1997;**350**:1097–9.

466 29 Chung SC, Gedeborg R, Nicholas O, *et al.* Acute myocardial infarction: A comparison of short-term survival in national outcome registries in Sweden and the UK. *Lancet* 2014;**383**:1305–12.

468 30 Stone GW, Selker HP, Thiele H, *et al.* Relationship between Infarct Size and Outcomes Following Primary PCI Patient-Level Analysis from 10 Randomized Trials. *J Am Coll Cardiol* 2016;**67**:1674–83. doi:10.1016/j.jacc.2016.01.069

471 31 Lewis JD, Bilker WB, Weinstein RB, *et al.* The relationship between time since registration and measured incidence rates in the General Practice Research Database. *Pharmacoepidemiol Drug Saf* 2005;**14**:443–51.

474 32 Hazelbag CM, Klungel OH, van Staa TP, *et al.* Left truncation results in substantial bias of the relation between time-dependent exposures and adverse events. *Ann Epidemiol* 2015;**25**:590–6.

476 33 Alexandrescu R, Bottle A, Jarman B, *et al.* Current ICD10 codes are insufficient to clearly distinguish acute myocardial infarction type: a descriptive study. *BMC Health Serv Res* 2013;**13**:468.http://www.ncbi.nlm.nih.gov/pubmed/24195773



For peer review only - http://bmjopen.bmj.com/site/about/guidelines.xhtml

34 Dahabreh IJ, Kent DM. Index event bias as an explanation for the paradoxes of recurrence risk research. *Jama* 2011;**305**:822–3.

For peer review only

**Figure titles and legends**

**Figure 1 – Flowchart of study population**

CALIBER=Cardiovascular disease research using Linked Bespoke studies and electronic health Records; CPRD=Clinical Practice Research Datalink; HF=heart failure; MI=myocardial infarction.

**Figure 2 – Incidence of HF after index MI**

Kaplan-Meier curves for crude incidence of HF after MI. **Left panel** 30-day follow-up after index MI. **Right panel** 10 year follow-up (median follow-up time was 3.7 years) in patients who survived the first 30 days and did not develop HF during the first 30 days (30-days event free). HF=heart failure; MI=myocardial infarction. “N at risk” represent the number of subjects at risk at a certain time point. “Cum events” represents the cumulative number of events since the previous time points. A spike histogram is provided (in red) at the top of the graphs providing information on the number of event across time.

**Figure 3 – Incidence of HF after index MI by group**

Kaplan-Meier curves for crude incidence of HF after MI stratified by group. **Top left panel:** 30-day follow-up after index MI stratified by age group. **Top right panel:** 10-year follow-up (median follow-up time was 3.7 years) in 30-day event free patients stratified by age group. Log-rank test of ≥65 year old patients compared to patients aged 50-65 years ($p<0.001$). **Bottom left panel:** 30-day follow-up after index MI stratified by MI type. **Bottom right panel:** 10-year follow-up (median follow-up time was 3.7 years) in patients who survived the first 30 days and did not develop HF during the first 30 days stratified by MI type. Log-rank test of NSTEMI compared to STEMI ($p<0.001$). HF=heart failure; MI=myocardial infarction. N at risk” represent the number of subjects at risk at a certain time point, with the cumulative number of events since the previous time points presented between brackets.

For peer review only

Table 1 - Baseline characteristics at index myocardial infarction

| | STEMI patients n = 4,657 | NSTEMI patients n = 19,822 | Total MI patients n = 24,479 | Unknown (%) |
|---|---|---|---|---|
| Follow-up time (years), median (IQR) | 3.3 (1.4–5.7) | 3.9 (1.5–7.1) | 3.7 (1.5–6.7) | 0% |
| Mean age, years (SD) | 65.7 (13.2) | 68.7 (13.2) | 68.1 (13.2) | 0% |
| Male sex | 3311 (71.1%) | 12658 (63.9%) | 15969 (65.2%) | 0% |
| Ethnicity | | | | |
| White | 3466 (76.4%) | 14288 (76.1%) | 17754 (76.2%) | 4.8% |
| Asian | 83 (1.8%) | 327 (1.7%) | 410 (1.8%) | |
| Black | 20 (0.4%) | 63 (0.3%) | 83 (0.4%) | |
| Other | 968 (21.3%) | 4085 (21.7%) | 5053 (21.7%) | |
| Body mass index (BMI in kg/m$^2$) | | | | |
| Underweight (18.5 <) | 22 (1.7%) | 120 (2.0%) | 142 (1.9%) | 70.0% |
| Normal (18.5; 25) | 335 (25.5%) | 1661 (27.4%) | 1996 (27.2%) | |
| Overweight (25, 30) | 557 (43.0%) | 2431 (40.2%) | 2988 (40.7%) | |
| Obese (>30) | 381 (29.4%) | 1842 (30.4%) | 2223 (30.2%) | |
| Index of multiple deprivation | | | | |
| Most deprived quintile | 892 (19.2%) | 3985 (20.2%) | 4877 (20%) | 0.4% |
| | | | | |
| **Risk factors before index MI** | | | | |
| Current smoker | 1235 (51.1%) | 3303 (39.5%) | 4538 (42.1%) | 55.9% |
| Excess alcohol consumption | 55 (9.0%) | 219 (8.1%) | 274 (8.3%) | 86.4% |
| History of atrial fibrillation | 358 (7.7%) | 2210 (11.1%) | 2568 (10.5%) | 0% |
| History of hypertension | 2117 (45.5%) | 10141 (51.2%) | 12258 (50.1%) | 0% |
| History of peripheral arterial disease | 236 (5.1%) | 1475 (7.4%) | 1711 (7%) | 0% |
| Previous revascularization | | | | |
| PCI | 700 (15.0%) | 2044 (10.3%) | 2744 (11.2%) | 0% |
| CABG | 126 (2.7%) | 843 (4.3%) | 969 (4.0%) | 0% |
| Previous TIA | 154 (3.3%) | 998 (5.0%) | 1152 (4.7%) | 0% |
| Previous stroke | 80 (1.7%) | 410 (2.1%) | 490 (2.0%) | 0% |
| History of diabetes | 538 (11.6%) | 2476 (12.6%) | 3014 (12.3%) | 0% |
| History of thyroid disease | 237 (5.1%) | 1374 (6.9%) | 1661 (6.6%) | 0% |
| History of COPD | 304 (6.5%) | 1634 (8.2%) | 1938 (7.9%) | 0% |
| History of non-metastatic cancer | 478 (10.3%) | 2282 (11.5%) | 2760 (11.3%) | 0% |
| | | | | |
| **Vital signs before admission** median (IQR) | | | | |
| Systolic blood pressure, mmHg | 140 (130–153) | 140 (130–154) | 140 (130–154) | 34.2% |
| Diastolic blood pressure, mmHg | 80 (74–88) | 80 (71–88) | 80 (72–88) | 34.2% |
| | | | | |
| **Biomarkers,** median (IQR) | | | | |
| Troponin I (maximum) | 18.4 (3.6–50.0) | 2.2 (0.3–10.0) | 3.88 (0.62–21.4) | 88.3% |
| Troponin T (maximum) | 1.8 (0.60–4.8) | 0.4 (0.14–1.1) | 0.65 (0.18–2) | 90.8% |
| CK (maximum) | 721 (222–1631) | 219 (97–648) | 331 (123–1068) | 79.8% |
| | | | | |
| **Biomarkers before index MI** mean (SD) | | | | |
| Haemoglobin, g/dL, | 14.1 (1.72) | 13.6 (1.84) | 13.7 (1.83) | 67.1% |
| White blood cell count | 8.0 (2.94) | 7.9 (2.92) | 7.9 (2.92) | 69.1% |
| Neutrophil count | 5.0 (2.12) | 5.0 (2.40) | 5.0 (2.35) | 71.9% |
| Platelets | 270 (85.2) | 266 (88.0) | 267 (87.5) | 69.1% |
| Erythrocyte sedimentation rate | 19.5 (18.8) | 22.2 (21.6) | 21.8 (21.2) | 89.4% |
| Creatinine, µmol/L | 98.5 (39.1) | 102.5 (47.3) | 102 (46.0) | 58.7% |
| eGFR - CKD-EPI | 70.4 (19.9) | 66.2 (19.9) | 67.0 (20.0) | 60.4% |
| Random glucose concentration, mmol/L | 7.09 (3.53) | 7.26 (3.89) | 7.23 (3.82) | 76.5% |
| Total cholesterol | 5.39 (1.45) | 5.24 (1.28) | 5.27 (1.31) | 63.4% |
| LDL cholesterol | 3.24 (1.12) | 3.08 (1.10) | 3.11 (1.11) | 81.3% |
| | | | | |
| **Revascularization characteristics** | | | | |
| Site of infarction | | | | |
| Anterior | 393 (40.2%) | 255 (30.7%) | 648 (35.9%) | 92.6% |
| Primary PCI | 258 (19.3%) | 73 (3.2%) | 331 (9.2%) | 85.3% |
| Prehospital fibrinolysis | 324 (7.0%) | 94 (0.5%) | 418 (1.7%) | 0% |
| Median (IQR) delay from symptom to reperfusion (min) | 150 (98 – 280) | 160 (101 – 296) | 153 (99 – 285) | 88.3% |
| | | | | |


For peer review only - http://bmjopen.bmj.com/site/about/guidelines.xhtml

| | | | | |
|---|---|---|---|---|
| **Prescribed medication before index MI** | | | | |
| Antiplatelet | 867 (18.6%) | 6979 (35.2%) | 7846 (32.1%) | 0% |
| Oral anticoagulant | 67 (1.4%) | 624 (3.1%) | 691 (2.8%) | 0% |
| Statin | 825 (17.7%) | 5297 (26.7%) | 6122 (25.0%) | 0% |
| ACE inhibitor | 676 (14.5%) | 4363 (22.0%) | 5039 (20.6%) | 0% |
| Angiotensin receptor blocker (ARB) | 220 (4.7%) | 1208 (6.1%) | 1428 (5.8%) | 0% |
| Beta-blocker | 766 (16.4%) | 5363 (27.1%) | 6129 (25.0%) | 0% |
| Calcium channel blocker | 804 (17.3%) | 4621 (23.3%) | 5425 (22.2%) | 0% |
| Loop diuretic | 255 (5.5%) | 2188 (11.0%) | 2443 (10.0%) | 0% |
| Aldosterone antagonist | 24 (0.5%) | 198 (1.0%) | 222 (0.9%) | 0% |
| Digoxin | 53 (1.1%) | 515 (2.6%) | 568 (2.3%) | 0% |

IQR=inter-quartile range; SD=standard deviation; MI=myocardial infarction; STEMI=ST-segment-elevation myocardial infarction; PCI=Percutaneous Coronary Intervention; CABG=Coronary Artery Bypass Grafting; TIA=Transient Ischaemic Attack; COPD=Chronic Obstructive Pulmonary Disease; CK=Creatine kinase; ACE=Angiotensin-converting-enzyme. Prehospital and in-hospital fibrinolysis are not mutually exclusive.

For peer review only

**Table 2 - Hazard ratio for heart failure in patients following a first myocardial infarction using multivariable Cox regression**

| | Model 1<br>n = 24,479<br>n events = 5,775 | Model 2<br>n = 24,479<br>n events = 5,775 | Model 3<br>n = 24,479<br>n events = 5,775 | Model 4<br>n = 24,479<br>n events = 5,775 |
|---|---|---|---|---|
| | HR (95% CI) | HR (95% CI) | HR (95% CI) | HR (95% CI) |
| Age, per 10 years | 1.51 (1.48 – 1.55) | 1.49 (1.46 – 1.53) | 1.46 (1.43 – 1.50) | 1.45 (1.41 – 1.49) |
| Men | 1.06 (1.00 – 1.12) | 1.08 (1.02 – 1.14) | 1.07 (1.01 – 1.13) | 1.06 (1.00 – 1.12) |
| Index of multiple deprivation | | overall p = <0.001 | overall p = <0.001 | overall p = <0.001 |
| Q1 (least deprived) | | Reference | Reference | Reference |
| Q2 | | 1.09 (0.99 – 1.20) | 1.09 (0.99 – 1.20) | 1.08 (0.99 – 1.19) |
| Q3 | | 1.20 (1.09 – 1.33) | 1.20 (1.08 – 1.32) | 1.18 (1.07 – 1.31) |
| Q4 | | 1.20 (1.08 – 1.33) | 1.20 (1.08 – 1.33) | 1.17 (1.06 – 1.30) |
| Q5 (most deprived) | | 1.30 (1.16 – 1.45) | 1.30 (1.16 – 1.45) | 1.27 (1.13 – 1.41) |
| History of hypertension | | 1.19 (1.12 – 1.26) | 1.17 (1.10 – 1.24) | 1.16 (1.09 – 1.23) |
| History of diabetes | | 1.48 (1.38 – 1.60) | 1.49 (1.38 – 1.60) | 1.45 (1.35 – 1.56) |
| History of atrial fibrillation | | | 1.65 (1.53 – 1.77) | 1.62 (1.51 – 1.75) |
| Type of MI<br>STEMI | | | 1.17 (1.10 – 1.26) | 1.19 (1.11 – 1.27) |
| History of peripheral arterial disease | | | | 1.38 (1.26 – 1.51) |
| History of COPD | | | | 1.28 (1.17 – 1.40) |
| Prescribed ACE inhibitor before MI | | | | 1.06 (0.99 – 1.13) |
| Prescribed Angiotensin receptor blocker (ARB) before MI | | | | 1.00 (0.89 – 1.11) |
| Prescribed Beta-blocker before MI | | | | 0.93 (0.88 – 1.00) |

All analyses presented in table 2 were performed on a complete case dataset of 24,479 subjects. HR=hazard ratio; CI=confidence interval; Q=quintile; MI=myocardial infarction; NSTEMI=non-ST-segment-elevation myocardial infarction; STEMI=ST-segment-elevation myocardial infarction; COPD=chronic obstructive pulmonary disease; ACE=Angiotensin-converting enzyme.

For peer review only



For peer review only - http://bmjopen.bmj.com/site/about/guidelines.xhtml

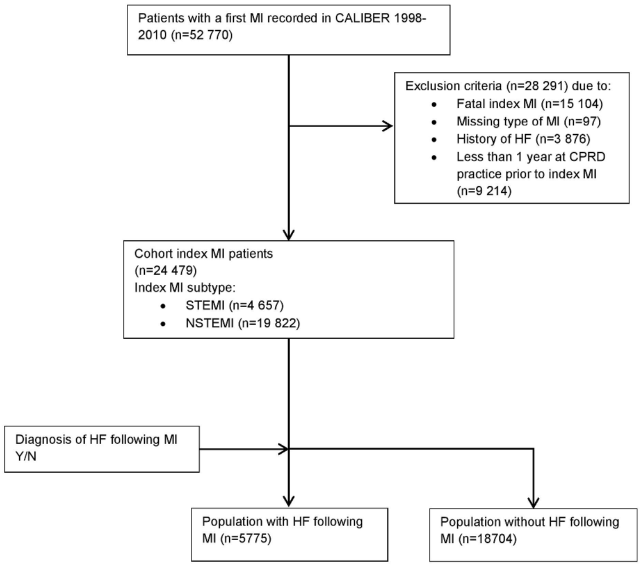


209x297mm (300 x 300 DPI)

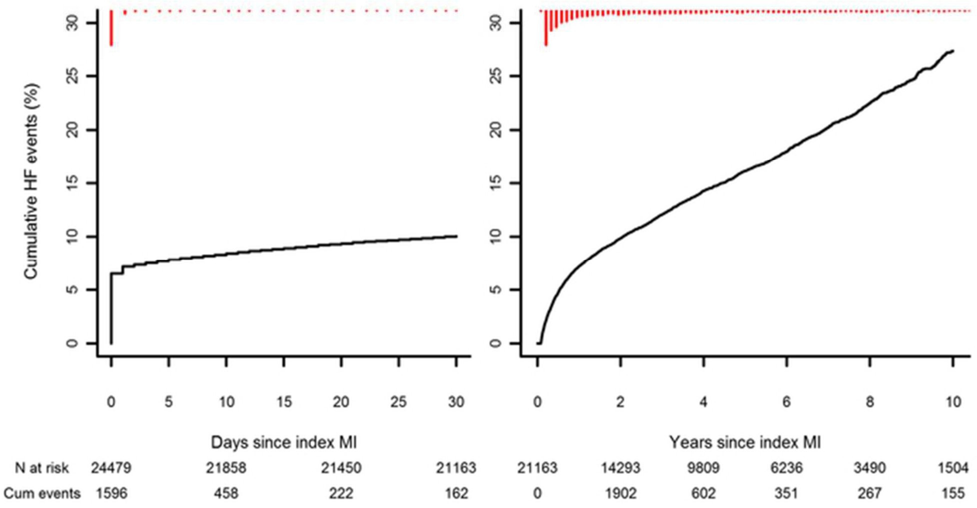


59x35mm (300 x 300 DPI)

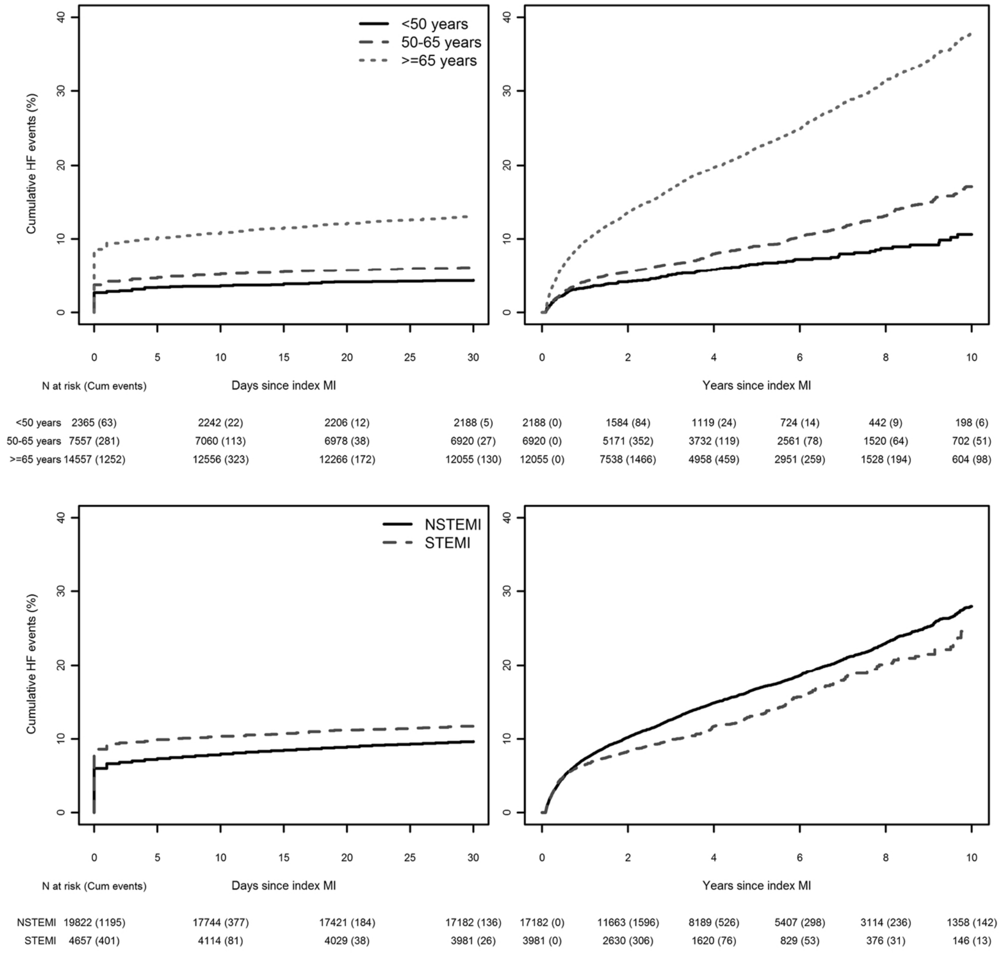


139x139mm (300 x 300 DPI)

**Supplementary Data**

# An Electronic Health Records cohort study on Heart Failure Following Myocardial Infarction in England: Incidence and Predictors

Johannes M.I.H. Gho, Amand F. Schmidt, Laura Pasea, Stefan Koudstaal, Mar Pujades-Rodriguez, Spiros Denaxas, Anoop D. Shah, Riyaz S. Patel, Chris P. Gale, Arno W. Hoes, John G. Cleland, Harry Hemingway, Folkert W. Asselbergs

For peer review only

Phenotype coding lists

**CALIBER myocardial infarction phenotype**
Fatal or non-fatal myocardial infarction (MI) as recorded in any of the constituent CALIBER data sources. The type of myocardial infarction (ST elevation MI, STEMI; or non ST elevation MI, NSTEMI) is recorded only in MINAP and CPRD.

Read codes used to identify myocardial infarction in primary care records (Clinical Research Practice Datalink)

| Category | Clinical code | Clinical term | Datasource lookup |
|---|---|---|---|
| **STEMI (3)** | G30X000 | Acute ST segment elevation myocardial infarction | 12229 |
| **NSTEMI (4)** | G307100 | Acute non-ST segment elevation myocardial infarction | 10562 |
| **Acute MI not further specified (5)** | 323..00 | ECG: myocardial infarction | 7783 |
| **Acute MI not further specified (5)** | 3233.00 | ECG: antero-septal infarct. | 26975 |
| **Acute MI not further specified (5)** | 3234.00 | ECG:posterior/inferior infarct | 26972 |
| **Acute MI not further specified (5)** | 3235.00 | ECG: subendocardial infarct | 55401 |
| **Acute MI not further specified (5)** | 3236.00 | ECG: lateral infarction | 52705 |
| **Acute MI not further specified (5)** | 323Z.00 | ECG: myocardial infarct NOS | 59032 |
| **Acute MI not further specified (5)** | 889A.00 | Diab mellit insulin-glucose infus acute myocardial infarct | 61670 |
| **Acute MI not further specified (5)** | G30..00 | Acute myocardial infarction | 241 |
| **Acute MI not further specified (5)** | G30..12 | Coronary thrombosis | 2491 |
| **Acute MI not further specified (5)** | G30..13 | Cardiac rupture following myocardial infarction (MI) | 30421 |
| **Acute MI not further specified (5)** | G30..15 | MI - acute myocardial infarction | 1677 |
| **Acute MI not further specified (5)** | G30..16 | Thrombosis - coronary | 13571 |
| **Acute MI not further specified (5)** | G300.00 | Acute anterolateral infarction | 12139 |
| **Acute MI not further specified (5)** | G301.00 | Other specified anterior myocardial infarction | 5387 |
| **Acute MI not further specified (5)** | G301000 | Acute anteroapical infarction | 40429 |
| **Acute MI not further specified (5)** | G301100 | Acute anteroseptal infarction | 17872 |
| **Acute MI not further specified (5)** | G301z00 | Anterior myocardial infarction NOS | 14897 |
| **Acute MI not further specified (5)** | G302.00 | Acute inferolateral infarction | 8935 |
| **Acute MI not further specified (5)** | G303.00 | Acute inferoposterior infarction | 29643 |
| **Acute MI not further specified (5)** | G304.00 | Posterior myocardial infarction NOS | 23892 |
| **Acute MI not further specified (5)** | G305.00 | Lateral myocardial infarction NOS | 14898 |
| **Acute MI not further specified (5)** | G306.00 | True posterior myocardial infarction | 63467 |
| **Acute MI not further specified (5)** | G307.00 | Acute subendocardial infarction | 3704 |
| **Acute MI not further specified (5)** | G307000 | Acute non-Q wave infarction | 9507 |

For peer review only

| | | | |
|---|---|---|---|
| **Acute MI not further specified (5)** | G308.00 | Inferior myocardial infarction NOS | 1678 |
| **Acute MI not further specified (5)** | G309.00 | Acute Q-wave infarct | 30330 |
| **Acute MI not further specified (5)** | G30B.00 | Acute posterolateral myocardial infarction | 32854 |
| **Acute MI not further specified (5)** | G30X.00 | Acute transmural myocardial infarction of unspecif site | 29758 |
| **Acute MI not further specified (5)** | G30y.00 | Other acute myocardial infarction | 34803 |
| **Acute MI not further specified (5)** | G30y000 | Acute atrial infarction | 28736 |
| **Acute MI not further specified (5)** | G30y100 | Acute papillary muscle infarction | 62626 |
| **Acute MI not further specified (5)** | G30y200 | Acute septal infarction | 41221 |
| **Acute MI not further specified (5)** | G30yz00 | Other acute myocardial infarction NOS | 46017 |
| **Acute MI not further specified (5)** | G30z.00 | Acute myocardial infarction NOS | 14658 |
| **Acute MI not further specified (5)** | G31y100 | Microinfarction of heart | 68357 |
| **Acute MI not further specified (5)** | G38..00 | Postoperative myocardial infarction | 32272 |
| **Acute MI not further specified (5)** | G380.00 | Postoperative transmural myocardial infarction anterior wall | 46112 |
| **Acute MI not further specified (5)** | G381.00 | Postoperative transmural myocardial infarction inferior wall | 46276 |
| **Acute MI not further specified (5)** | G384.00 | Postoperative subendocardial myocardial infarction | 41835 |
| **Acute MI not further specified (5)** | G38z.00 | Postoperative myocardial infarction, unspecified | 68748 |
| **Acute MI not further specified (5)** | Gyu3400 | [X]Acute transmural myocardial infarction of unspecif site | 96838 |

Hospital Episode Statistics

| **Category (code)** | ICD10 code | ICD10 term |
|---|---|---|
| **Acute MI not further specified (5)** | I21 | Acute myocardial infarction |

OPCS

| **Category (code)** | OPCS 4 | OPCS 4 term |
|---|---|---|
| **Transluminal coronary thrombolysis (2)** | K50.2 | Percutaneous transluminal coronary thrombolysis using streptokinase |
| **Transluminal coronary thrombolysis (2)** | K50.3 | Percutaneous transluminal injection of therapeutic substance into coronary artery NEC |

Office for National Statistics (ONS)

| **Category (code)** | ICD10 code | ICD10 term |
|---|---|---|
| **Death from MI (3)** | I21 | Acute myocardial infarction |
| **Death from MI (3)** | I22 | Subsequent myocardial infarction |
| **Death from MI (3)** | I23 | Certain current complications following acute myocardial infarction |

Office for National Statistics (ONS) prior to 2000 (ICD9)

| **Category (code)** | ICD9 code | ICD9 term |
|---|---|---|
| **Death from myocardial infarction (3)** | 410 | Acute myocardial infarction |
| **Death from myocardial infarction (3)** | 4110 | Other acute and subacute forms of ischemic heart disease ; Postmyocardial infarction syndrome |

For peer review only

| | | |
|---|---|---|
| **Death from myocardial infarction (3)** | 4297 | Ill-defined descriptions and complications of heart disease ; Certain sequelae of myocardial infarction, not elsewhere classified |

Myocardial Ischaemia National Audit Project (MINAP)

1 Acute STEMI

2 Acute NSTEMI

## Phenotype coding lists

**CALIBER myocardial infarction phenotype**
Fatal or non-fatal myocardial infarction (MI) as recorded in any of the constituent CALIBER data sources. The type of myocardial infarction (ST elevation MI, STEMI; or non ST elevation MI, NSTEMI) is recorded only in MINAP and CPRD.

Read codes used to identify myocardial infarction in primary care records (Clinical Research Practice Datalink)

| Category | Clinical code | Clinical term | Datasource lookup |
|---|---|---|---|
| **STEMI (3)** | G30X000 | Acute ST segment elevation myocardial infarction | 12229 |
| **NSTEMI (4)** | G307100 | Acute non-ST segment elevation myocardial infarction | 10562 |
| **Acute MI not further specified (5)** | 323..00 | ECG: myocardial infarction | 7783 |
| **Acute MI not further specified (5)** | 3233.00 | ECG: antero-septal infarct. | 26975 |
| **Acute MI not further specified (5)** | 3234.00 | ECG:posterior/inferior infarct | 26972 |
| **Acute MI not further specified (5)** | 3235.00 | ECG: subendocardial infarct | 55401 |
| **Acute MI not further specified (5)** | 3236.00 | ECG: lateral infarction | 52705 |
| **Acute MI not further specified (5)** | 323Z.00 | ECG: myocardial infarct NOS | 59032 |
| **Acute MI not further specified (5)** | 889A.00 | Diab mellit insulin-glucose infus acute myocardial infarct | 61670 |
| **Acute MI not further specified (5)** | G30..00 | Acute myocardial infarction | 241 |
| **Acute MI not further specified (5)** | G30..12 | Coronary thrombosis | 2491 |
| **Acute MI not further specified (5)** | G30..13 | Cardiac rupture following myocardial infarction (MI) | 30421 |
| **Acute MI not further specified (5)** | G30..15 | MI - acute myocardial infarction | 1677 |
| **Acute MI not further specified (5)** | G30..16 | Thrombosis - coronary | 13571 |
| **Acute MI not further specified (5)** | G300.00 | Acute anterolateral infarction | 12139 |
| **Acute MI not further specified (5)** | G301.00 | Other specified anterior myocardial infarction | 5387 |
| **Acute MI not further specified (5)** | G301000 | Acute anteroapical infarction | 40429 |
| **Acute MI not further specified (5)** | G301100 | Acute anteroseptal infarction | 17872 |
| **Acute MI not further specified (5)** | G301z00 | Anterior myocardial infarction NOS | 14897 |
| **Acute MI not further specified (5)** | G302.00 | Acute inferolateral infarction | 8935 |
| **Acute MI not further specified (5)** | G303.00 | Acute inferoposterior infarction | 29643 |
| **Acute MI not further specified (5)** | G304.00 | Posterior myocardial infarction NOS | 23892 |
| **Acute MI not further specified (5)** | G305.00 | Lateral myocardial infarction NOS | 14898 |

For peer review only

| | | | |
|---|---|---|---|
| **Acute MI not further specified (5)** | G306.00 | True posterior myocardial infarction | 63467 |
| **Acute MI not further specified (5)** | G307.00 | Acute subendocardial infarction | 3704 |
| **Acute MI not further specified (5)** | G307000 | Acute non-Q wave infarction | 9507 |
| **Acute MI not further specified (5)** | G308.00 | Inferior myocardial infarction NOS | 1678 |
| **Acute MI not further specified (5)** | G309.00 | Acute Q-wave infarct | 30330 |
| **Acute MI not further specified (5)** | G30B.00 | Acute posterolateral myocardial infarction | 32854 |
| **Acute MI not further specified (5)** | G30X.00 | Acute transmural myocardial infarction of unspecif site | 29758 |
| **Acute MI not further specified (5)** | G30y.00 | Other acute myocardial infarction | 34803 |
| **Acute MI not further specified (5)** | G30y000 | Acute atrial infarction | 28736 |
| **Acute MI not further specified (5)** | G30y100 | Acute papillary muscle infarction | 62626 |
| **Acute MI not further specified (5)** | G30y200 | Acute septal infarction | 41221 |
| **Acute MI not further specified (5)** | G30yz00 | Other acute myocardial infarction NOS | 46017 |
| **Acute MI not further specified (5)** | G30z.00 | Acute myocardial infarction NOS | 14658 |
| **Acute MI not further specified (5)** | G31y100 | Microinfarction of heart | 68357 |
| **Acute MI not further specified (5)** | G38..00 | Postoperative myocardial infarction | 32272 |
| **Acute MI not further specified (5)** | G380.00 | Postoperative transmural myocardial infarction anterior wall | 46112 |
| **Acute MI not further specified (5)** | G381.00 | Postoperative transmural myocardial infarction inferior wall | 46276 |
| **Acute MI not further specified (5)** | G384.00 | Postoperative subendocardial myocardial infarction | 41835 |
| **Acute MI not further specified (5)** | G38z.00 | Postoperative myocardial infarction, unspecified | 68748 |
| **Acute MI not further specified (5)** | Gyu3400 | [X]Acute transmural myocardial infarction of unspecif site | 96838 |

Hospital Episode Statistics

| **Category (code)** | ICD10 code | ICD10 term |
|---|---|---|
| **Acute MI not further specified (5)** | I21 | Acute myocardial infarction |

OPCS

| **Category (code)** | OPCS 4 | OPCS 4 term |
|---|---|---|
| **Transluminal coronary thrombolysis (2)** | K50.2 | Percutaneous transluminal coronary thrombolysis using streptokinase |
| **Transluminal coronary thrombolysis (2)** | K50.3 | Percutaneous transluminal injection of therapeutic substance into coronary artery NEC |

Office for National Statistics (ONS)

| **Category (code)** | ICD10 code | ICD10 term |
|---|---|---|
| **Death from MI (3)** | I21 | Acute myocardial infarction |
| **Death from MI (3)** | I22 | Subsequent myocardial infarction |
| **Death from MI (3)** | I23 | Certain current complications following acute myocardial infarction |

Office for National Statistics (ONS) prior to 2000 (ICD9)

For peer review only

| Category (code) | ICD9 code | ICD9 term |
|---|---|---|
| **Death from myocardial infarction (3)** | 410 | Acute myocardial infarction |
| **Death from myocardial infarction (3)** | 4110 | Other acute and subacute forms of ischemic heart disease ; Postmyocardial infarction syndrome |
| **Death from myocardial infarction (3)** | 4297 | Ill-defined descriptions and complications of heart disease ; Certain sequelae of myocardial infarction, not elsewhere classified |

Myocardial Ischaemia National Audit Project (MINAP)

| | |
|---|---|
| 1 | Acute STEMI |
| 2 | Acute NSTEMI |

**CALIBER heart failure phenotype**

Primary care
1. Diagnosis of left ventricular dysfunction during echocardiogram during a consultation
2. Diagnosis of heart failure of valvular cause during a consultation
3. Diagnosis of heart failure due to hypertension during a consultation
4. Diagnosis of heart failure due to unspecified cause during a consultation
5. Diagnosis of heart failure not otherwise specified during a consultation

Secondary care
1. Diagnosis of heart failure due to hypertension as the primary diagnosis during a hospitalization
2. Diagnosis of heart failure not otherwise specified as the primary diagnosis during a hospitalization

Mortality
1. Hypertensive heart failure as the underlying cause of death
2. Heart failure not otherwise specified as the underlying cause of death

Read codes used to identify myocardial infarction in primary care records (Clinical Research Practice Datalink)

| Category (code) | Clinical code | Clinical term | Datasource lookup |
|---|---|---|---|
| **Heart failure due to valvular disease (3)** | G580400 | Congestive heart failure due to valvular disease | 94870 |
| **Heart failure due to hypertension (4)** | G210.00 | Malignant hypertensive heart disease | 50157 |
| **Heart failure due to hypertension (4)** | G210000 | Malignant hypertensive heart disease without CCF | 95334 |
| **Heart failure due to hypertension (4)** | G210100 | Malignant hypertensive heart disease with CCF | 72668 |
| **Heart failure due to hypertension (4)** | G211100 | Benign hypertensive heart disease with CCF | 52127 |
| **Heart failure due to hypertension (4)** | G21z100 | Hypertensive heart disease NOS with CCF | 62718 |
| **Heart failure due to hypertension (4)** | G230.00 | Malignant hypertensive heart and renal disease | 67232 |
| **Heart failure due to hypertension (4)** | G232.00 | Hypertensive heart&renal dis wth (congestive) heart failure | 21837 |
| **Heart failure due to hypertension (4)** | G234.00 | Hyperten heart&renal dis+both(congestv)heart and renal fail | 57987 |
| **Heart failure due to other cause (5)** | G1yz100 | Rheumatic left ventricular failure | 22262 |
| **Heart failure NOS (6)** | 1O1..00 | Heart failure confirmed | 9913 |
| **Heart failure NOS (6)** | 662W.00 | Heart failure annual review | 30779 |
| **Heart failure NOS (6)** | 662p.00 | Heart failure 6 month review | 83502 |
| **Heart failure NOS (6)** | 8B29.00 | Cardiac failure therapy | 24503 |
| **Heart failure NOS (6)** | 8H2S.00 | Admit heart failure emergency | 32898 |
| **Heart failure NOS (6)** | 9Or0.00 | Heart failure review completed | 19380 |
| **Heart failure NOS (6)** | G400.00 | Acute cor pulmonale | 8464 |
| **Heart failure NOS (6)** | G41z.11 | Chronic cor pulmonale | 5695 |
| **Heart failure NOS (6)** | G554000 | Congestive cardiomyopathy | 5141 |
| **Heart failure NOS (6)** | G554011 | Congestive obstructive cardiomyopathy | 68766 |
| **Heart failure NOS (6)** | G58..00 | Heart failure | 2062 |
| **Heart failure NOS (6)** | G58..11 | Cardiac failure | 1223 |
| **Heart failure NOS (6)** | G580.00 | Congestive heart failure | 398 |

For peer review only

| | | | |
|---|---|---|---|
| **Heart failure NOS (6)** | G580.11 | Congestive cardiac failure | 2906 |
| **Heart failure NOS (6)** | G580.12 | Right heart failure | 10079 |
| **Heart failure NOS (6)** | G580.13 | Right ventricular failure | 10154 |
| **Heart failure NOS (6)** | G580.14 | Biventricular failure | 9524 |
| **Heart failure NOS (6)** | G580000 | Acute congestive heart failure | 23707 |
| **Heart failure NOS (6)** | G580100 | Chronic congestive heart failure | 32671 |
| **Heart failure NOS (6)** | G580200 | Decompensated cardiac failure | 27884 |
| **Heart failure NOS (6)** | G580300 | Compensated cardiac failure | 11424 |
| **Heart failure NOS (6)** | G581.00 | Left ventricular failure | 884 |
| **Heart failure NOS (6)** | G581.11 | Asthma - cardiac | 23481 |
| **Heart failure NOS (6)** | G581.13 | Impaired left ventricular function | 5942 |
| **Heart failure NOS (6)** | G581000 | Acute left ventricular failure | 5255 |
| **Heart failure NOS (6)** | G582.00 | Acute heart failure | 27964 |
| **Heart failure NOS (6)** | G58z.00 | Heart failure NOS | 4024 |
| **Heart failure NOS (6)** | G58z.12 | Cardiac failure NOS | 17278 |
| **Heart failure NOS (6)** | G5yy900 | Left ventricular systolic dysfunction | 8966 |
| **Heart failure NOS (6)** | G5yyA00 | Left ventricular diastolic dysfunction | 12550 |
| **Heart failure NOS (6)** | R2y1000 | [D]Cardiorespiratory failure | 20324 |
| **Left ventricular dysfunction (3)** | 585f.00 | Echocardiogram shows left ventricular systolic dysfunction | 11284 |
| **Left ventricular dysfunction (3)** | 585g.00 | Echocardiogram shows left ventricular diastolic dysfunction | 11351 |

Hospital Episode Statistics

| Category (code) | ICD10 code | ICD10 term |
|---|---|---|
| **HF - hypertension cause (4)** | I110 | Hypertensive heart disease with (congestive) heart failure |
| **HF - hypertension cause (4)** | I130 | Hypertensive heart and renal disease with (congestive) heart failure |
| **HF - hypertension cause (4)** | I132 | Hypertensive heart and renal disease with both (congestive) heart failure and renal failure |
| **HF - other unspecified cause (5)** | I260 | Pulmonary embolism with mention of acute cor pulmonale |
| **HF - not otherwise specified (6)** | I50 | Heart failure |

Office for National Statistics (ONS)

| Category (code) | ICD10 code | ICD10 term |
|---|---|---|
| **Hypertensive heart failure (4)** | I110 | Hypertensive heart disease with (congestive) heart failure |
| **Hypertensive heart failure (4)** | I130 | Hypertensive heart and renal disease with (congestive) heart failure |
| **Hypertensive heart failure (4)** | I132 | Hypertensive heart and renal disease with both (congestive) heart failure and renal failure |
| **Heart failure with other specified cause (5)** | I260 | Pulmonary embolism with mention of acute cor pulmonale |
| **Heart failure with unspecified cause (6)** | I50 | Heart failure |

Office for National Statistics (ONS) prior to 2000 (ICD9)

| Category (code) | ICD9 code | ICD9 term |
|---|---|---|
| **Heart failure with other specified cause (5)** | 4151 | Acute pulmonary heart disease ; Pulmonary embolism and infarction |
| **Heart failure with unspecified cause (6)** | 428 | Heart failure |

Myocardial Ischaemia National Audit Project (MINAP)

1 CCF

**CALIBER baseline definitions**
See also: https://www.caliberresearch.org

For peer review only

Definitions used for smoking at baseline
CPRD:
**smoking_cessation_gprd**
Prescription of smoking cessation medication in primary care. Currently lookup is performed from using BNF chapter 4.10.2.
**smoking_status_gprd**
An individual record of smoking status in primary care. A patient may have multiple records of smoking status.
HES:
**smoking_status_hes**
Smoking status recorded in HES. Currently there are only ICD-10 codes for smoking, no codes for ex-smoking or non-smoking.

Definitions used for hypertension at baseline
CPRD:
**ht_gprd**
Diagnosis of hypertension
HES:
**ht_hes**
Hypertension diagnosis recorded in secondary care.

Definitions used for diabetes at baseline
CPRD:
**dm_gprd**
Type of diabetes diagnosis at a particular point in time.
**diabdiag_gprd**
GP diagnosis of diabetes mellitus using a specific type I or type II diagnosis in the Read diagnosis chapter (i.e. codes starting with C08, C09, C10E, C10F). Vague terms such as `juvenile onset diabetes` or `insulin dependent diabetes` are not included.
HES:
**dm_hes**
Diagnosis of diabetes in secondary care (HES).

For peer review only

Supplementary Methods

**Ethical approval**
CALIBER was approved by the Lewisham Local Research Ethics Committee (ref: 09/H0810/16 date: 08/04/2009) and the Ethics and Confidentiality Committee (ECC) (ref: ECC 2-06(b)/2009 CALIBER dataset). CALIBER has been registered with the University College London Data Protection Officer (ref: Z6364106/2009/2/26). CALIBER EHR data are anonymised; individual informed consent was not sought from study participants.

**Multiple imputation algorithm**
Multiple imputation was implemented using the 'mice' package for R, to replace missing values in prognostic covariates (body mass index, smoking, systolic and diastolic blood pressure).[1] Imputation models included general practice and 3-year periods from 1998 to 2010 of index myocardial infarction (MI) and:

1. All the baseline covariates as used in the multivariable analysis (age, sex, index of multiple deprivation, hypertension, diabetes, type of MI, body mass index, smoking, systolic blood pressure, diastolic blood pressure);
2. Baseline measurements of covariates not considered in the multivariable analysis (alcohol intake, haemoglobin, white blood cell count, creatinine, alanine aminotransferase (ALAT), total cholesterol, HDL cholesterol);
3. Baseline medications prior to index MI (antiplatelet, oral anticoagulant, statin, ACE inhibitor, angiotensin receptor blocker (ARB), beta-blocker, calcium channel blocker, loop diuretic, aldosterone antagonist, digoxin);
4. Coexisting medical conditions (history of atrial fibrillation, peripheral arterial disease, depression, cancer, chronic obstructive pulmonary disease);
5. The Nelson-Aalen hazard and the event status for the outcomes analysed.

Missing data was multiply imputed and across 20 datasets a (stratified) multivariable Cox regression model was fit and results were combined using Rubin's rules.[2]

References

1. van Buuren S. Multiple imputation of discrete and continuous data by fully conditional specification. Stat Methods Med Res 2007;16:219-42.
2. Carpenter JR, Kenward MG. *Multiple Imputation and its Application*: Wiley; 2013.

For peer review only

**Table S1 - Hazard ratios for heart failure in patients following a first myocardial infarction using the competing risks regression based on Fine and Gray's proportional subhazards model to account for the competing risk of all cause mortality**

| | Model 1 | Model 2 | Model 3 | Model 4 |
|---|---|---|---|---|
| | HR (95% CI) | HR (95% CI) | HR (95% CI) | HR (95% CI) |
| Age, per 10 years increase | 1.41 (1.38 – 1.44) | 1.39 (1.36 – 1.42) | 1.36 (1.33 – 1.39) | 1.35 (1.32 – 1.38) |
| Men | 1.04 (0.99 – 1.10) | 1.07 (1.01 – 1.13) | 1.06 (1.00 – 1.12) | 1.05 (1.00 – 1.11) |
| Index of multiple deprivation | | overall p = <0.001 | overall p = <0.001 | overall p = <0.001 |
| Q1 | | Reference | Reference | Reference |
| Q2 | | 1.11 (1.03 – 1.21) | 1.11 (1.02 – 1.20) | 1.10 (1.01 – 1.20) |
| Q3 | | 1.21 (1.12 – 1.32) | 1.20 (1.11 – 1.31) | 1.19 (1.09 – 1.29) |
| Q4 | | 1.24 (1.14 – 1.35) | 1.24 (1.14 – 1.34) | 1.22 (1.12 – 1.32) |
| Q5 | | 1.36 (1.25 – 1.47) | 1.35 (1.25 – 1.47) | 1.32 (1.21 – 1.43) |
| History of hypertension | | 1.15 (1.09 – 1.22) | 1.13 (1.07 – 1.19) | 1.14 (1.08 – 1.21) |
| History of diabetes | | 1.39 (1.29 – 1.49) | 1.38 (1.29 – 1.48) | 1.36 (1.27 – 1.47) |
| History of atrial fibrillation | | | 1.50 (1.40 – 1.61) | 1.49 (1.39 – 1.60) |
| Type of MI | | | | |
| STEMI | | | 1.10 (1.03 – 1.18) | 1.11 (1.03 – 1.18) |
| History of peripheral arterial disease | | | | 1.29 (1.19 – 1.40) |
| History of COPD | | | | 1.18 (1.09 – 1.29) |
| ACE inhibitor use before MI | | | | 1.01 (0.95 – 1.08) |
| Angiotensin receptor blocker (ARB) use before MI | | | | 0.94 (0.85 – 1.05) |
| Beta-blocker use before MI | | | | 0.94 (0.88 – 1.00) |

Complete case analysis with the addition of clinically relevant variables per model (n = 24,479, n events = 5,775). Overall p-values are shown for categorical covariates. ACE=Angiotensin-converting enzyme; CI=confidence interval; COPD=chronic obstructive pulmonary disease; MI=myocardial infarction; NSTEMI=non-ST-segment-elevation myocardial infarction; STEMI=ST-segment-elevation myocardial infarction; Q=quintile; HR=ratio of the subdistribution hazards.

For peer review only

**Table S2 - Hazard ratios for heart failure in patients following a first myocardial infarction in sensitivity analyses**

| | Model 5<br>Cox regression<br>complete case without multiple imputation<br>n = 4,898<br>n events = 1,159 | Model 5<br>Cox regression<br>with multiple imputation<br>n = 24,479<br>n events = 5,775 | Model 5<br>Competing risk analysis<br>with complete case<br>n = 4,898<br>n events = 1,159 |
|---|---|---|---|
| | HR (95% CI) | HR (95% CI) | HR (95% CI) |
| Age, per 10 years increase | 1.48 (1.38 – 1.60) | 1.47 (1.43 – 1.51) | 1.44 (1.35 – 1.54) |
| Men | 1.03 (0.89 – 1.19) | 1.07 (1.01 – 1.13) | 1.07 (0.95 – 1.22) |
| Body mass index (BMI in kg/m$^2$) | p = 0.005 | p = <0.001 | p = < 0.001 |
| Underweight (18.5 <) | 0.78 (0.48 – 1.32) | 0.76 (0.58 – 0.99) | 0.71 (0.45 – 1.12) |
| Normal (18.5; 25) | Reference | Reference | Reference |
| Overweight (25, 30) | 0.84 (0.71 – 0.99) | 0.84 (0.76 – 0.92) | 0.84 (0.73 – 0.97) |
| Obese (>30) | 1.12 (0.94 – 1.34) | 0.98 (0.90 – 1.08) | 1.17 (1.00 – 1.36) |
| Index of multiple deprivation | p = 0.040 | p < 0.001 | p = < 0.001 |
| Q1 | Reference | Reference | Reference |
| Q2 | 1.22 (0.94 – 1.58) | 1.08 (0.98 – 1.19) | 1.35 (1.10 – 1.66) |
| Q3 | 1.34 (1.03 – 1.75) | 1.17 (1.06 – 1.30) | 1.40 (1.14 – 1.71) |
| Q4 | 1.23 (0.93 – 1.63) | 1.16 (1.05 – 1.29) | 1.31 (1.07 – 1.61) |
| Q5 | 1.45 (1.09 – 1.92) | 1.25 (1.12 – 1.40) | 1.61 (1.33 – 1.96) |
| Smoking status | p = 0.792 | p = 0.012 | p = 0.952 |
| Non-smoker | Reference | Reference | Reference |
| Current smoker | 0.95 (0.77 – 1.14) | 1.14 (1.04 – 1.25) | 1.02 (0.86 – 1. 20) |
| Ex-smoker | 0.98 (0.83 – 1.15) | 1.03 (0.94 – 1.12) | 0.99 (0.87 – 1.14) |
| History of hypertension | 1.14 (0.96 – 1.35) | 1.16 (1.09 – 1.24) | 1.18 (1.02 – 1.37) |
| History of diabetes | 1.28 (1.09 – 1.49) | 1.46 (1.36 – 1.58) | 1.23 (1.08 – 1.39) |
| History of atrial fibrillation | 1.49 (1.25 – 1.76) | 1.63 (1.51 – 1.75) | 1.37 (1.18 – 1.59) |
| Type of MI | | | |
| STEMI | 1.15 (0.97 – 1.37) | 1.18 (1.10 – 1.27) | 1.10 (0.95 – 1.28) |
| History of peripheral arterial disease | 1.53 (1.25 – 1.87) | 1.37 (1.25 – 1.50) | 1.33 (1.13 – 1.57) |
| History of COPD | 1.21 (0.99 – 1.48) | 1.24 (1.15 – 1.38) | 1.22 (1.03 – 1.44) |
| Systolic blood pressure, per 10 mmHg increase | 0.99 (0.95 – 1.03) | 1.00 (0.98 – 1.02) | 1.01 (0.97 – 1.04) |
| Diastolic blood pressure, per 10 mmHg increase | 1.01 (0.94 – 1.09) | 0.99 (0.96 – 1.03) | 0.99 (0.93 – 1.06) |
| ACE inhibitor use before MI | 1.01 (0.87 – 1.17) | 1.06 (0.99 – 1.14) | 1.07 (0.94 – 1.21) |
| Angiotensin receptor blocker (ARB) use before MI | 0.99 (0.95 – 1.03) | 1.00 (0.90 – 1.12) | 0.93 (0.78 – 1.12) |
| Beta-blocker use before MI | 1.01 (0.94 – 1.09) | 0.94 (0.88 – 1.00) | 0.90 (0.79 – 1.02) |

Using multivariable Cox regression without and with multiple imputation and the competing risks regression based on Fine and Gray's proportional subhazards model to account for the competing risk of all cause mortality (n = 4898, n events = 1159). Additional prognostic covariates: body mass index, smoking, systolic and diastolic blood pressure. Overall p-values are shown for categorical covariates. HR=hazard ratio; CI=confidence interval; MI=myocardial infarction; NSTEMI=non-ST-segment-elevation myocardial infarction; STEMI=ST-segment-elevation myocardial infarction; COPD=chronic obstructive pulmonary disease; ACE=Angiotensin-converting enzyme; Q=quintile.

For peer review only

**Table S3 - Hazard ratios for heart failure in patients following a first myocardial infarction stratified on having a previous cancer diagnosis or not.**

| | Model 4<br>**Subjects without a history of cancer**<br>n = 21,719<br>n events = 5,029 | Model 4<br>**Subjects with a history of cancer**<br>n = 2,760<br>n events = 746 |
|---|---|---|
| | HR (95% CI) | HR (95% CI) |
| Age, per 10 years increase | 1.49 (1.45 – 1.53) | 1.45 (1.30 – 1.61) |
| Men | 1.08 (1.01 – 1.15) | 1.13 (0.93 – 1.37) |
| Index of multiple deprivation | overall p = <0.001 | overall p = <0.001 |
| Q1 | Reference | Reference |
| Q2 | 1.08 (0.97 – 1.20) | 1.07 (0.77 – 1.48) |
| Q3 | 1.17 (1.05 – 1.31) | 1.27 (0.91 – 1.78) |
| Q4 | 1.17 (1.04 – 1.31) | 1.14 (0.79 – 1.66) |
| Q5 | 1.25 (1.10 – 1.41) | 1.43 (0.97 – 2.11) |
| History of hypertension | 1.11 (1.03 – 1.18) | 1.45 (1.16 – 1.81) |
| History of diabetes | 1.53 (1.41 – 1.66) | 1.19 (0.91 – 1.55) |
| History of atrial fibrillation | 1.62 (1.49 – 1.76) | 1.67 (1.33 – 2.09) |
| Type of MI | | |
| STEMI | 1.18 (1.09 – 1.28) | 1.01 (0.77 – 1.31) |
| History of peripheral arterial disease | 1.39 (1.26 – 1.54) | 1.19 (0.88 – 1.62) |
| History of COPD | 1.30 (1.18 – 1.43) | 1.36 (1.03 – 1.81) |
| ACE inhibitor use before MI | 1.11 (1.03 – 1.20) | 1.00 (0.79 – 1.26) |
| Angiotensin receptor blocker (ARB) use before MI | 1.06 (0.93 – 1.20) | 0.92 (0.65 – 1.31) |
| Beta-blocker use before MI | 0.97 (0.90 – 1.04) | 0.99 (0.79 – 1.24) |

All analyses are based on a complete case analysis. Overall p-values are shown for categorical covariates. ACE=Angiotensin-converting enzyme; CI=confidence interval; COPD=chronic obstructive pulmonary disease; MI=myocardial infarction; NSTEMI=non-ST-segment-elevation myocardial infarction; STEMI=ST-segment-elevation myocardial infarction; Q=quintile.

For peer review only

**Table S4 - Hazard ratios for heart failure in patients following a first myocardial infarction stratified on a history of revascularization.**

| | Model 4 **Subjects without a history of revascularization** n = 21,160 n events = 5,222 | Model 4 **Subjects with a history of revascularization** n = 3.319 n events = 553 |
|---|---|---|
| | HR (95% CI) | HR (95% CI) |
| Age, per 10 years increase | 1.48 (1.44 – 1.52) | 1.30 (1.17 – 1.44) |
| Men | 1.07 (1.01 – 1.14) | 0.96 (0.75 – 1.23) |
| Index of multiple deprivation | overall p = <0.001 | overall p = <0.001 |
| Q1 | Reference | Reference |
| Q2 | 1.06 (0.95 – 1.17) | 1.13 (0.80 – 1.61) |
| Q3 | 1.14 (1.02 – 1.27) | 1.39 (0.95 – 2.03) |
| Q4 | 1.15 (1.02 – 1.28) | 1.34 (0.85 – 2.02) |
| Q5 | 1.22 (1.08 – 1.38) | 1.29 (0.84 – 1.99) |
| History of hypertension | 1.14 (1.07 – 1.22 | 1.18 (0.92 – 1.50) |
| History of diabetes | 1.49 (1.37 – 1.61) | 1.32 (1.00 – 1.75) |
| History of atrial fibrillation | 1.61 (1.48 – 1.74) | 1.90 (1.39 – 2.58) |
| Type of MI | | |
| STEMI | 1.14 (1.05 – 1.23) | 1.39 (1.07 – 1.79) |
| History of peripheral arterial disease | 1.40 (1.27 – 1.55) | 1.22 (0.88 – 1.62) |
| History of COPD | 1.30 (1.18 – 1.43) | 1.36 (0.92 – 2.03) |
| ACE inhibitor use before MI | 1.11 (1.03 – 1.19) | 1.15 (0.89 – 1.49) |
| Angiotensin receptor blocker (ARB) use before MI | 1.06 (0.94 – 1.19) | 0.79 (0.54 – 1.17) |
| Beta-blocker use before MI | 1.00 (0.94 – 1.07) | 0.84 (0.65 – 1.08) |

All analyses are based on a complete case analysis. Overall p-values are shown for categorical covariates. ACE=Angiotensin-converting enzyme; CI=confidence interval; COPD=chronic obstructive pulmonary disease; MI=myocardial infarction; NSTEMI=non-ST-segment-elevation myocardial infarction; STEMI=ST-segment-elevation myocardial infarction; Q=quintile.

For peer review only

**Supplementary Figure 1 – Cumulative incidence curves for HF accounting for mortality as first event after index MI**

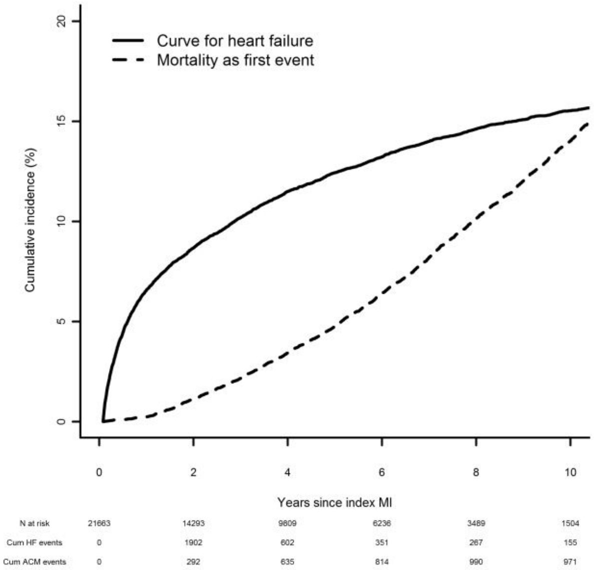


Cumulative incidence curves adjusted for competing risk of mortality as first event. 10-year follow-up in patients who survived the first 30 days and did not develop HF during the first 30 days (30 days event free).

For peer review only

STROBE Statement—checklist of items that should be included in reports of observational studies

| | Item No | Recommendation |
|---|---|---|
| **Title and abstract** | 1 ✓ | (*a*) ✓*page 2* - Indicate the study's design with a commonly used term in the title or the abstract |
| | | (*b*) ✓*page 2* - Provide in the abstract an informative and balanced summary of what was done and what was found |
| **Introduction** | | |
| Background/rationale | 2✓ | ✓*page 4* - Explain the scientific background and rationale for the investigation being reported |
| Objectives | 3✓ | ✓ *page 4* - State specific objectives, including any prespecified hypotheses |
| **Methods** | | |
| Study design | 4✓ | ✓*pages 4 through 6* - Present key elements of study design early in the paper |
| Setting | 5✓ | Describe the setting, locations, and relevant dates, including periods of recruitment, exposure, follow-up, and data collection |
| Participants | 6✓ | ✓*page 5* - (*a*) *Cohort study*—Give the eligibility criteria, and the sources and methods of selection of participants. Describe methods of follow-up<br>*Case-control study*—Give the eligibility criteria, and the sources and methods of case ascertainment and control selection. Give the rationale for the choice of cases and controls<br>*Cross-sectional study*—Give the eligibility criteria, and the sources and methods of selection of participants |
| | | (*b*) *Cohort study*—For matched studies, give matching criteria and number of exposed and unexposed<br>*Case-control study*—For matched studies, give matching criteria and the number of controls per case |
| Variables | 7✓ | ✓*page 5* - Clearly define all outcomes, exposures, predictors, potential confounders, and effect modifiers. Give diagnostic criteria, if applicable |
| Data sources/ measurement | 8✓* | ✓*page 5* - For each variable of interest, give sources of data and details of methods of assessment (measurement). Describe comparability of assessment methods if there is more than one group |
| Bias | 9✓ | ✓*page 6* - Describe any efforts to address potential sources of bias |
| Study size | 10✓ | ✓*page 7 and figure 1*- Explain how the study size was arrived at |
| Quantitative variables | 11✓ | ✓*page 6* - Explain how quantitative variables were handled in the analyses. If applicable, describe which groupings were chosen and why |
| Statistical methods | 12✓ | (*a*) *NA* Describe all statistical methods, including those used to control for confounding |
| | | (*b*) *NA* Describe any methods used to examine subgroups and interactions |
| | | (*c*) ✓*page 6* - Explain how missing data were addressed |
| | | (*d*) ✓*page 6* - *Cohort study*—If applicable, explain how loss to follow-up was addressed<br>*Case-control study*—If applicable, explain how matching of cases and controls was addressed<br>*Cross-sectional study*—If applicable, describe analytical methods taking account of sampling strategy |
| | | (*e*) ✓Describe any sensitivity analyses |


For peer review only - http://bmjopen.bmj.com/site/about/guidelines.xhtml

Continued on next page

For peer review only


For peer review only - http://bmjopen.bmj.com/site/about/guidelines.xhtml

| | | |
|---|---|---|
| **Results** | | |
| Participants | 13* | ✓*page 6* - (a) Report numbers of individuals at each stage of study—eg numbers potentially eligible, examined for eligibility, confirmed eligible, included in the study, completing follow-up, and analysed |
| | | ✓*page 6 and figure 1* - (b) Give reasons for non-participation at each stage |
| | | ✓*figure 1* - (c) Consider use of a flow diagram |
| Descriptive data | 14*✓ | (a) ✓*table 1* - Give characteristics of study participants (eg demographic, clinical, social) and information on exposures and potential confounders |
| | | (b) ✓*table 1* - Indicate number of participants with missing data for each variable of interest |
| | | (c) ✓*table 1- Cohort study*—Summarise follow-up time (eg, average and total amount) |
| Outcome data | 15* | ✓*table 1, figures 2-3 and pages 7-8 Cohort study*—Report numbers of outcome events or summary measures over time |
| | | *Case-control study*—Report numbers in each exposure category, or summary measures of exposure |
| | | *Cross-sectional study*—Report numbers of outcome events or summary measures |
| Main results | 16 | ✓*table 2, appendix tables* and pages 7-8 - (*a*) Give unadjusted estimates and, if applicable, confounder-adjusted estimates and their precision (eg, 95% confidence interval). Make clear which confounders were adjusted for and why they were included |
| | | *NA* (*b*) Report category boundaries when continuous variables were categorized |
| | | ✓ absolute rates reported in pages 7-8 - (*c*) If relevant, consider translating estimates of relative risk into absolute risk for a meaningful time period |
| Other analyses | 17 | ✓*pages 7-8* - Report other analyses done—eg analyses of subgroups and interactions, and sensitivity analyses |
| **Discussion** | | |
| Key results | 18 | ✓*page 8* Summarise key results with reference to study objectives |
| Limitations | 19 | ✓*pages 9-10* - Discuss limitations of the study, taking into account sources of potential bias or imprecision. Discuss both direction and magnitude of any potential bias |
| Interpretation | 20 | ✓*pages 8-10* Give a cautious overall interpretation of results considering objectives, limitations, multiplicity of analyses, results from similar studies, and other relevant evidence |
| Generalisability | 21 | ✓ *page 9* Discuss the generalisability (external validity) of the study results |
| **Other information** | | |
| Funding | 22 | ✓*page 11* Give the source of funding and the role of the funders for the present study and, if applicable, for the original study on which the present article is based |

*Give information separately for cases and controls in case-control studies and, if applicable, for exposed and unexposed groups in cohort and cross-sectional studies.

**Note:** An Explanation and Elaboration article discusses each checklist item and gives methodological background and published examples of transparent reporting. The STROBE checklist is best used in conjunction with this article (freely available on the Web sites of PLoS Medicine at http://www.plosmedicine.org/, Annals of Internal Medicine at http://www.annals.org/, and Epidemiology at http://www.epidem.com/). Information on the STROBE Initiative is available at www.strobe-statement.org.

For peer review only
